# Associations of Wellbeing Levels, Changes, and Within-Person Variability With Late-Life All-Cause Mortality Across 12 Years: Contrasting Hedonic vs. Eudaimonic Wellbeing Among Very Old Adults

**DOI:** 10.3389/fpsyg.2021.750891

**Published:** 2022-01-11

**Authors:** Oliver Karl Schilling, Markus Wettstein, Hans-Werner Wahl

**Affiliations:** ^1^Department of Psychological Aging Research, Psychological Institute, Heidelberg University, Heidelberg, Germany; ^2^Network Aging Research, Heidelberg University, Heidelberg, Germany; ^3^Department of Psychology, Humboldt University Berlin, Berlin, Germany

**Keywords:** very old age, late-life mortality, longevity, hedonic wellbeing, eudaimonic wellbeing, autonomy, oldest-old

## Abstract

Advanced old age has been characterized as a biologically highly vulnerable life phase. Biological, morbidity-, and cognitive impairment-related factors play an important role as mortality predictors among very old adults. However, it is largely unknown whether previous findings confirming the role of different wellbeing domains for mortality translate to survival among the oldest-old individuals. Moreover, the distinction established in the wellbeing literature between hedonic and eudaimonic wellbeing as well as the consideration of within-person variability of potentially relevant mortality predictors has not sufficiently been addressed in prior mortality research. In this study, we examined a broad set of hedonic and eudaimonic wellbeing indicators, including their levels, their changes, as well as their within-person variability, as predictors of all-cause mortality in a sample of very old individuals. We used data from the LateLine study, a 7-year longitudinal study based on a sample of *n* = 124 individuals who were living alone and who were aged 87–97 years (*M* = 90.6, *SD* = 2.9) at baseline. Study participants provided up to 16 measurement occasions (mean number of measurement occasions per individual = 5.50, *SD* = 4.79) between 2009 and 2016. Dates of death were available for 118 individuals (95.2%) who had deceased between 2009 and 2021. We ran longitudinal multilevel structural equation models and specified between-person level differences, within-person long-term linear change trends, as well as the “detrended” within-person variability in three indicators of hedonic (i.e., life satisfaction and positive and negative affect) and four indicators of eudaimonic wellbeing (i.e., purpose in life, autonomy, environmental mastery, and self-acceptance) as all-cause mortality predictors. Controlling for age, gender, education, and physical condition and testing our sets of hedonic and eudaimonic indictors separately in terms of their mortality impact, solely one eudaimonic wellbeing indicator, namely, autonomy, showed significant effects on survival. Surprisingly, autonomy appeared “paradoxically” related with mortality, with high individual levels and intraindividual highly stable perceptions of autonomy being associated with a shorter residual lifetime. Thus, it seems plausible that accepting dependency and changing perceptions of autonomy over time in accordance with objectively remaining capabilities might become adaptive for survival in very old age.

## Introduction

The fourth age has been described, both from a theoretical perspective as well as from an empirical perspective, as a biologically highly vulnerable phase in life (Baltes, 1997; Baltes and Smith, 2003; Wahl and Ehni, 2020), characterized by changing life circumstances and loss experiences across various domains, such as decreasing physical health and functional ability, declining cognitive and sensory resources, as well as increasing constraints in self-regulation (Gerstorf and Ram, 2009). At the same time, such a one-sided loss portrayal of very old age needs differentiation as individuals in their fourth age also represent a selective group of long-term survivors who may possess particular psychosocial resources and a considerable amount of resilience which promotes survival. In fact, whereas objective health indicators exhibit a substantial decline in advanced old age, subjective health measures remain, on average, rather stable in the oldest-old individuals, which has been termed as a “late-life health paradox” (Wettstein et al., 2016). A major mechanism arguably contributing to this paradox seems to be flexible adjustments (“response shift”; Sprangers and Schwartz, 1999) of health-related expectations and goals (Heckhausen et al., 2013; Wahl et al., 2021). Thus, keeping high subjective standards in health, functional autonomy, and self-efficacy expectation, a constellation that has been found to serve adaptation in earlier life periods, may become quite dysfunctional in the fourth age. Moreover, “risk factor paradoxes,” such as identified for medical risk factors in geriatric research (Ahmadi et al., 2015), might also be detectable in psychological adaptation to typical late-life conditions (Schilling, 2016). This might be particularly true with regard to psychological aspects of wellbeing and their adaptive – or maladaptive – role in very old age, with wellbeing representing a domain of psychological functioning that can be considered as both outcome and driver of successful aging.

Important for this study, adding to studies of generally high wellbeing in later life despite experiences of loss (e.g., with regard to health, sensory abilities, and cognitive functioning; Kunzmann et al., 2000; Schilling, 2006), a range of wellbeing indicators of very old adults have been found to remain quite high in very late life (Wettstein et al., 2015; Jopp et al., 2016). For instance, Wettstein et al. (2015) investigated wellbeing trajectories in very old age based on a broad set of indicators representing hedonic as well as eudaimonic wellbeing (Ryan and Deci, 2001) and observed 4-year mean-level stability in several indicators [negative affect (NA), autonomy, and self-acceptance].

### Conceptual Diversity of Wellbeing: Hedonic vs. Eudaimonic Facets

The term wellbeing denotes a broadly defined and multi-faceted construct, covering a multitude of indicators referring to different specific aspects of optimal experience and functioning (Ryan and Deci, 2001). A basic theoretical distinction established in current wellbeing research has been made between hedonic and eudaimonic approaches. Specifically, whereas the focus of hedonic wellbeing is on “happiness, generally defined as the presence of positive affect and the absence of negative affect” (Deci and Ryan, 2008, Abstract), eudaimonic wellbeing rather refers to living well, self-realization, and self-fulfillment of potentials of individuals (Ryan and Deci, 2001; Deci and Ryan, 2008; Keyes et al., 2002).

The hedonic view covers at least three distinctive “sub-constructs,” namely the so-called cognitive component of satisfaction with life, referring to individuals’ evaluations of their life circumstances, and the affective components of experiencing positive and negative emotions (Diener et al., 1999; Diener and Ryan, 2009).

Prominent theories of eudaimonic wellbeing include self-determination theory (SDT, Ryan and Deci, 2001; Deci and Ryan, 2002) and the concept of psychological wellbeing (PWB) proposed by Ryff and Keyes (1995) and Ryff and Singer (2006). While SDT supposes three basic components – autonomy, relatedness, and competence – viewed as basic and universal psychological needs and motivational forces of human life, PWB includes six dimensions, overlapping with regard to content with the SDT components, but also with hedonic facets (Keyes et al., 2002). Considering this diversity of hedonic and eudaimonic wellbeing is key to the examination of how wellbeing might be associated with mortality in very old age. That is, different facets and empirically measurable indicators differ by the underlying psychological theory regarding their role as motivators driving and/or as outcomes of individual engagement and adaptation across the life span. Thus, expecting differential pathways connecting certain wellbeing measures with mortality, it is a yet open key question whether or to what extent which aspects of wellbeing might be relevant for survival at the far end of the human life span and how they differ in such relevance.

We posit that the hedonic and eudaimonic wellbeing distinction may not only represent different “philosophical” ways to approach a “good life” across the life span and in old age but also possess a differential and in fact ambiguous resource quality to counteract biological forces and promote longevity. For example, regarding hedonic wellbeing, it could be that maximizing the experience of positive emotions serves to counteract depression and loneliness, both established mortality risk factors (Fernández-Ballesteros and Sánchez-Izquierdo, 2019). For instance, the broaden-and-built theory of positive emotions (Fredrickson, 2004) states that positive emotions broaden the thought-action repertoire of an individual, building personal resources which might be adaptive and beneficial for long-term survival. Moreover, positive affect (PA) and NA have been proposed to serve basic (bio-)behavioral activation vs. inhibition systems, respectively (Watson et al., 1999; Quilty and Oakman, 2004). According to this reasoning, PA serves to activate behavioral engagements toward rewarding and beneficial experiences, whereas NA serves an inhibitory function, preventing the organism from actions that may provoke unpleasant and harmful outcomes. Thus, considering hedonic wellbeing with regard to behavioral activation vs. inhibition, things might get complicated in very old age, when individuals have become more vulnerable due to increasing psycho-social loss and health constraints. Given such increased vulnerability, maintaining active could be adaptive in keeping the person vital and preventing the decline of functionality, but could also increase risks of an overburdening and harmful lifestyle. In contrast, behavioral inhibition might get more adaptive in preventing harm and depletion, therefore, prolonging survival. Thus, it could be considered that the activating vs. inhibitory functions of the affective system exert some counter-intuitive associations with survival in very old age, in which high levels of PA, as well as low NA, could be coupled with maladaptive behaviors that compromise – rather than promote – survival.

Bringing these considerations to a more general dimension, it may be the selective “orchestration” of active engagements vs. protective disengagements of an individual that is crucial for their mortality. In this orchestration, eudaimonic wellbeing may come into play. Eudaimonic wellbeing more strongly operates at the “meaning-making” level of life in very old age and, therefore, may guide selective engagements and disengagements of very old individuals according to basic needs implied in the facets of eudaimonic facets of wellbeing. Eudaimonic wellbeing might motivate the oldest-old individuals to prevent self-harming or self-depletive engagements, motivate them to cope with major health threats, and activate social resources, both eventually contributing to the prolongation of life (Ryff and Singer, 2009; Fernández-Ballesteros and Sánchez-Izquierdo, 2019). However, eudaimonic facets such as “feeling as an autonomous human being” that echo fundamental human needs (Ryan and Deci, 2001) may become dysfunctional in very old age, so that they could result in constant failure experiences and eventually strong feelings of learned helplessness, which have been shown to increase the mortality risk (Seligman, 2000). In conclusion, facets of both hedonic and eudaimonic wellbeing can be expected to differentially exert both protective and detrimental effects when it comes to mortality.

However, to the best of our knowledge, no empirical study so far has tested a broad range of hedonic as well as eudaimonic indicators in parallel within one analysis as predictors of all-cause mortality in advanced old age. Whereas the role of (self-rated and objective) health, cognition, disability, and other morbidity-related factors for longevity is established by various empirical findings (Parker et al., 1992; Idler and Benyamini, 1997; Ganguli et al., 2002; DeSalvo et al., 2006; Tiainen et al., 2013; Eekhoff et al., 2019; Aichele et al., 2021), less is known about the mortality-relevance of psychosocial factors such as wellbeing, particularly from a differential perspective of wellbeing domains and with regard to the life phase of very old age. Notably, wellbeing effects and health-related factors on mortality seem to be independent (Ambrasat et al., 2011), i.e., associations between wellbeing and mortality are not spurious and caused by morbidity-related factors. Moreover, a recent study by Puterman et al. (2020) demonstrated that from a large set of 57 factors, psychological factors (such as NA) belong to the strongest predictors of mortality, in addition to behavioral, economic, and social factors.

### Wellbeing and Mortality: The Evidence So Far

Numerous studies revealed that higher levels of wellbeing – mostly operationalized by hedonic indicators such as life satisfaction or PA/NA, but some also including eudaimonic components of wellbeing – are associated with longer survival (Lyubomirsky et al., 2005; Chida and Steptoe, 2008; Diener and Chan, 2011; Ong et al., 2011).

Higher wellbeing levels, particularly positive emotions, but also components of eudaimonic wellbeing (Steptoe et al., 2015; Martín-María et al., 2017), seem to affect longevity via various pathways, e.g., by influencing biological processes and immune functioning (Ryff and Singer, 2006; Steptoe et al., 2012), by buffering the detrimental role of stress on health outcomes (Pressman and Cohen, 2005; Pressman et al., 2019), by facilitating the accumulation of health-relevant resources (such as social networks), or by being associated with better, “longevity-promoting” health behaviors. As already mentioned, the broaden-and-built theory of positive emotions (Fredrickson, 2004) states that positive emotions broaden the thought-action repertoire of an individual and build personal resources, which might be adaptive and beneficial for long-term survival.

Regarding one of the very few empirical studies that compared the predictive effects of various hedonic wellbeing indicators on mortality in parallel, Wiest et al. (2011) found that when controlling for lifestyle, sociodemographic, and health factors, only higher PA was associated with lower mortality risk among older adults, whereas life satisfaction and NA had no significant effect on mortality risk. Among middle-aged individuals, none of these indicators was significantly related to mortality risk. However, other studies have found that life satisfaction is meaningfully related to mortality (Parker et al., 1992), also in the oldest-old individuals (Johansson and Thorvaldsson, 2021). In a large US panel study (Willroth et al., 2020), a joint model including several hedonic wellbeing indicators in parallel as mortality predictors was employed. Life satisfaction and NA were uniquely predictive of mortality, controlling for age, gender, and education. Thus, the existing evidence does not seem to be entirely consistent.

With regard to eudaimonic wellbeing, Hill and Turiano (2014) identified higher purpose in life as a protective factor against 14-year mortality. The effect of this eudaimonic indicator was independent of individuals’ age and remained significant when PA and NA, as well as positive relations with others as an additional eudaimonic wellbeing indicator, were controlled for (which were all non-significant predictors). Similarly, Boyle et al. (2009) reported that greater purpose in life is associated with reduced all-cause mortality, even when depressive symptoms, neuroticism, and other potential confounding factors are controlled for. Moreover, in a large 20-year prospective cohort study, higher self-acceptance was found to predict a lower mortality risk (Ng et al., 2020).

In conclusion, there is evidence in support of a beneficial effect of higher wellbeing – in terms of both hedonic and eudaimonic indicators – on longevity. However, the findings are not entirely consistent. Also, apart from a few exceptions (Wiest et al., 2011; Hill and Turiano, 2014; Ng et al., 2020), most studies did not contrast the role of different wellbeing indicators, such as hedonic in contrast to eudaimonic wellbeing indicators, and if so not in samples of oldest-old individuals, who represent a specific group with unique characteristics and risks. Martín-María et al. (2017) concluded from their meta-analysis regarding the impact of wellbeing on mortality that so far most empirical studies did only address one wellbeing aspect and that “even though most studies included older people, they reported results only for the total sample undifferentiated by age” (p. 573). Thus, integrative approaches investigating multiple facets of wellbeing from both domains in parallel and comparing their predictive strength are still rare, and little is known regarding the role of wellbeing for mortality specifically in the oldest-old individuals. In this study, we aimed to address these research gaps by investigating and contrasting the role of different well-being facets for mortality in very old adults.

### Wellbeing and Mortality From a Time-Varying Perspective

Notably, the findings outlined above were mostly derived from analyzing the “*status quo*” of wellbeing assessed at one point in time as a predictor of mortality risk. However, intraindividual dynamics of change and variability in wellbeing might also be related to mortality, by indicating accumulating dysfunction and approaching death. Hülür et al. (2017), for instance, used a large representative German longitudinal database to predict mortality hazards from the level and linear slopes of 20-year life satisfaction trajectories. Both components showed unique effects, in which higher levels, as well as less decline of life satisfaction, predicted longer survival. Apart from this predictive approach, evidence of time-to-death-related “terminal decline” in wellbeing indicators, including life satisfaction (Gerstorf et al., 2008a,b, 2010) and affective wellbeing (Schilling et al., 2013; Vogel et al., 2013; Windsor et al., 2015; Schilling et al., 2018), supports the notion that impending death is associated with a pronounced decline in wellbeing. With regard to very old age, oldest-old individuals are close to the end of their lives, thus in the stage of “tertiary aging” (Birren and Cunningham, 1985) which is characterized by “accelerated functional deteriorations that manifest shortly (months, maybe years) before death” (Ram et al., 2010, p. 28). Also, Ong and Steptoe (2020) found that higher instability of PA, in terms of more fluctuative short-term variability measured across the course of one day, was predictive of all-cause mortality among older adults.

The role of change and intraindividual variability in wellbeing for survival particularly in the oldest-old individuals has so far hardly been researched. Specifically, it may not only be wellbeing assessed at one point in time but also change and within-person variability in wellbeing that predict mortality in late life. This potentially mortality-predictive variability might not only be the average rate of change inherent in the slope component of an individual trajectory. Rather, the within-person variability in different wellbeing domains, which exists beyond a general change trend and which is pronounced in very old age (Wettstein et al., 2015), deserves consideration as a predictor of mortality, reflecting either increased vulnerability, general resource instability, or – alternatively – adaptive changeability in reaction to changing life circumstances. Change in wellbeing over time among the oldest-old individuals might even be better described in terms of within-person variability from one measurement to the next rather than as a systematic – linear or non-linear – trajectory of either increase or decline. Henceforth, we will refer to this intraindividual variability beyond the long-term linear trend as *detrended variability* (a term adopted from detrended fluctuation analysis; see for instance Hardstone et al., 2012)^1^.

The ambiguous role of intraindividual (detrended) variability – potentially not only in very old age but also across the life span in general – has been debated before. For instance, MacDonald and Stawski (2015) discussed that “as a predictor of various developmental processes, intraindividual variability can be either adaptive or maladaptive” and that “sometimes less [intraindividual variability] reflects vulnerability and sometimes more [intraindividual variability] does” (p. 249). Specifically, intraindividual variability in cognitive performances, such as speed, seems to indicate vulnerability as well as neurological dysfunction and is associated with impending death (MacDonald et al., 2008). Aslo, PA instability (i.e., higher within-person variability in PA) is associated with higher mortality risk (Ong and Steptoe, 2020). In contrast, intraindividual cognitive variability can also be adaptive in nature, e.g., for skill acquisition (Siegler, 1994). With regard to very old age, intraindividual variability could, in fact, be either adaptive or maladaptive. It might reflect instability or vulnerability and thus be dysfunctional with regard to outcomes such as longevity, but higher within-person variability could as well be indicative of adjustment processes to changing life circumstances and accumulating loss experiences in the oldest-old individuals.

Investigating such detrended variability requires a specific data set with multiple measurement occasions which are not too far apart. In this study, we used data provided by very old adults with an unusually high number of repeated observations, comprising up to 16 measurement occasions across a considerable time period spanning 7 years, assessed with 1-year measurement intervals between the first measurement occasions and 6-month and 4-month intervals thereafter. Thus, this study design allows for a thorough and systematic investigation of intraindividual variability of hedonic and eudaimonic wellbeing of very old adults without restrictions of previous typical longitudinal aging studies. Such typical studies usually provided a far smaller number of measurement occasions, and/or they are characterized by longer intervals between adjacent measurement occasions, thus potentially missing substantial amounts of within-person variability occurring over time periods of months, rather than years.

### This Study

Summarizing from above, this study examines predictive effects of wellbeing on mortality, aiming, in particular, to address three gaps in the evidence published so far: first, we focused on predictors of survival among *very old* individuals. Second, we addressed the diversity of wellbeing with an integrative examination of *multiple indicators of hedonic and eudaimonic wellbeing*. Third, we included *intraindividual changes* – in terms of long-term change trends, as well as within-person detrended variability – of wellbeing in the examination of mortality-predictive effects.

Given a lack of findings on these three specific aspects, we refrained from spelling-out hypotheses. Instead, we followed an exploratory approach in this study and examined whether multiple hedonic (life satisfaction, PA, and NA) and eudaimonic wellbeing indicators (autonomy, environmental mastery, purpose in life, and self-acceptance), i.e., their levels, changes, and their within-person variability, independently predict all-cause mortality in oldest-old individuals aged between 87 and 97 years at baseline. We purposefully stayed at the exploratory level of testing because, as outlined above, both hedonic and eudaimonic wellbeing indicators may come with risk and protective factors concerning mortality.

## Materials and Methods

### Sample and Procedure

We used data from the longitudinal project LateLine (Schilling et al., 2013; Neubauer et al., 2015; Wettstein et al., 2015, 2016), including 16 measurement occasions from 2009 to 2016 (T1–T16; see Table 1). The LateLine study followed up the German subsample of the multinational European ENABLE-AGE project (for detailed information on this parent sample and its recruitment, Iwarsson et al., 2007). ENABLE-AGE included originally 450 older individuals born between 1912 and 1922 and living alone in the Heidelberg-Mannheim area. The sample had been drawn randomly from the public registry data in 2002. All ENABLE-AGE participants from Germany were invited to participate in the LateLine follow-up study. The inclusion criterion of the parent sample of living alone was chosen because very old individuals who live alone might represent a particularly vulnerable group. Moreover, living alone is very common in advanced old age, e.g., due to widowhood (>50% of all individuals aged 80 years and older in Germany were living alone in 2002; Wahl and Heyl, 2015). The LateLine study sample consists of *n* = 124 very old individuals (see Table 1 for the numbers of participants at each measurement occasion; 11 “drop-ins” entered the study after T1). Each study participant provided, on average, 5.50 observations (*SD* = 4.79, range 1–16), amounting to *N* = 682 within-person level observations which should suffice for systematic in-depth analysis of the effects of within-person change and of detrended within-person variability on late-life mortality.

**TABLE 1:** LateLine study – measurement times, numbers of participants, and participants deceased between measurement occasions.

Measurement wave	Measurement period (month/year)	Number of participants (*N* = 124)	Participants excluded^a^	Participants deceased (%)^b^
T1	01/09–10/09	113	16	5 (4.0)
T2	01/10–09/10	92	12	14 (11.3)
T3	02/11–04/11	71	3	6 (4.8)
T4	07/11–10/11	61	1	7 (5.6)
T5	01/12–03/12	55	1	1 (0.8)
T6	06/12–08/12	51	2	12 (9.7)
T7	03/13–06/13	44	1	7 (5.7)
T8	07/13–10/13	39	1	4 (3.2)
T9	11/13–03/14	32	2	4 (3.2)
T10	03/14–06/14	30	0	3 (2.4)
T11	07/14–11/14	25	0	6 (4.8)
T12	12/14–03/15	19	0	4 (3.2)
T13	04/15–08/15	14	0	7 (5.7)
T14	09/15–12/15	15	1	4 (3.2)
T15	01/16–04/16	11	1	2 (1.6)
T16	05/16–08/16	10	1	32 (25.8)^c^
Sum		682	42	118 (95.2)

*^a^Criteria for exclusion: severe cognitive impairment, based in mini-mental state examination (MMSE) < 17 or interviewer face validity.*

*^b^Numbers of participants deceased until the beginning of the subsequent measurement period.*

*^c^Deceased until last mortality follow-up, 2 participants confirmed still alive in Oct 2021.*

Data collection was carried out during home visits by trained interviewers. Participants with severe cognitive impairment, i.e., with a score < 17 on the mini-mental state examination (MMSE; Folstein et al., 1975) were excluded from participation (see Table 1 for the number of excluded participants at each measurement occasion).

### Survival

On October 1, 2021, the date of our last mortality follow-up, 118 (94.4%) participants had died, with exact dates of death obtained from registration offices (see Table 1 for the exact numbers of deceased individuals per measurement occasion). Only 2 (1.6%) participants were still alive at that time (now centenarians), and for 4 (3.2%) participants, we could not get information about mortality status. *Time-to-death at baseline* was computed as distance in days from the “official” start of the LateLine project on January 1, 2009 to the day of death of individuals (for computational reasons, these time-to-death values were rescaled to distance in years). We included the 2 participants who were still alive in the survival analyses with time-to-death right-censored recorded as time distance between baseline and October 1, 2021. For the participants with unknown mortality status, time-to-death was set missing, implying that these were not included in the mortality analyses.

### Measures

#### Hedonic Wellbeing

As one indicator of hedonic wellbeing, *life satisfaction* was assessed by the Satisfaction with Life Scale (SWLS; Diener et al., 1985). This scale measures global life satisfaction with five items using a scale ranging from 1 (strongly disagree) to 5 (strongly agree). The internal consistency of the SWLS was α = 0.82 at baseline.

*Affective wellbeing* is another indicator of hedonic wellbeing and was assessed based on the positive and negative affect schedule (PANAS; Watson et al., 1988). The PANAS consists of 10 positive and 10 negative emotion adjectives. Participants have to report how often they have experienced each of the 20 emotions within a specified time using a scale ranging from 1 (never) to 5 (very often). We used the time since the last measurement occasion as a time frame to assess individual levels of affective wellbeing across broader time periods because such broader intervals are less affected by situational and contextual “noise” influences (such as the season of the respective assessment). PA and NA were computed by averaging all reported positive and negative emotion frequencies, respectively (internal consistency at baseline PA: α = 0.82, NA: α = 0.83).

#### Eudaimonic Wellbeing

Four subscales of the Ryff instrument of PWB (Ryff, 1989) were available and included. Each scale consists of nine items that were measured on a scale ranging from 1 (strongly disagree) to 5 (strongly agree). The PWB components are as follows: autonomy (e.g., “I have confidence in my opinions, even if they are contrary to the general consensus”; α = 0.72), environmental mastery (e.g., “In general, I feel I am in charge of the situation in which I live”; α = 0.74), purpose in life (e.g., “Some people wander aimlessly through life, but I am not one of them”; α = 0.69), and self-acceptance (e.g., “I like most aspects of my personality”; α = 0.80).

#### Covariates

We controlled for gender and education (years of schooling) as established social-demographic predictors of mortality in all analyses. We refrained from including self-report health measures due to their considerable overlap with wellbeing indicators. Therefore, we controlled for between-person differences in the objective health status of participants at baseline in which we used two measures of physical functioning in oldest-old age, handgrip strength, and chair-stand performance. Handgrip strength was measured with the Jamar hydraulic hand dynamometer, and the average score (in kg) of three successive trials was used in the analyses (Innes, 1999; Bohannon, 2019). The chair-stand performance test score, ranging from 0 to 4 (higher values indicating better performance), was measured via the chair-stand test from the short physical performance battery (Guralnik et al., 1994). Both tests are well-established measures used to assess physical functionality, overall fitness, and functional impairment of very old individuals.

### Statistical Analyses

To examine associations of wellbeing levels and trajectories with mortality of participants, we modeled individual linear growth curves of the wellbeing indicators and considered all three components (i.e., levels, slopes, and detrended within-person variability/individual residual variance) inherent in these growth curves as potential predictors of participants’ time-to-death. That is, first, the individual wellbeing level represents interindividual differences in wellbeing estimated for the study baseline date (January 1, 2009). Second, linear slopes represent systematic change trends in terms of an average rate of change per year (prior results using the same data did not suggest non-linear change trends among very old adults in the wellbeing indicators that are targeted in this study; see Wettstein et al., 2015). Third, we considered the residual variance of each individual representing the degree to which the wellbeing measures of individuals fluctuated around the slope trend, thus corresponding to detrended variability of individuals. We used multilevel structural equation models, including a within-person (level 1) location-scale model component to estimate the above-mentioned growth curve parameters as random effects (McNeish, 2020), and a between-person (level 2) regression of time-to-death on these random effects.

In formal terms, the model could be expressed as follows. First, for a given wellbeing indicator *W*, the within-person model equation is as follows:


(1) $$W_{t\,i}=L_{w\,i}+t\,S_{w\,i}+R_{w\,t\,i},$$

*W*_ti_ denotes the wellbeing score assessed from person *i* at time-in-study *t*, therefore, *L_wi_* and *S_wi_* denote the level and slope of the linear trajectory in *W* fitted to person *i*, *R_wti_* the residuals, i.e., the deviations of the scores from the linear trend, for which normality is assumed as usual for residuals, however, with person-specific residual variance $\sigma_{\text{wi}}^{2}$ modeled as random parameter, varying across persons and assumed to follow a lognormal distribution. Thus, with $\sigma_{\text{wi}}^{2}=\exp\left(\gamma_{\text{wv}}+\upsilon_{\text{wvi}}\right)$, the between-person part of the location-scale submodel may be specified as multivariate normal distribution:


(2) $$\left[\begin{array}{c} L_{w\,i}\\ S_{w\,i}\\ \ln\sigma_{w\,i}^{2} \end{array}\right]∼M\,V\,N\,\left(\left[\begin{array}{c} \gamma_{w\,l}\\ \gamma_{w\,s}\\ \gamma_{w\,v} \end{array}\right],\left[\begin{array}{ccc} \pi_{w\,l\,l} & & \\ \pi_{w\,l\,s} & \pi_{w\,s\,s} & \\ \pi_{w\,l\,v} & \pi_{w\,s\,v} & \pi_{w\,v\,v} \end{array}\right]\right),$$

where the πs denote the between-person random (co)variances, and γ*_wl_*, γ*_ws_*, and γ*_wv_*, denote the between-person means of the level, slope, and the logarithmized detrended variance of wellbeing indicator *W*, respectively.

Let the latter be $V_{w\,i}=\ln\sigma_{w\,i}^{2}$, then the between-person survival submodel employing wellbeing indicator *W* as a predictor is as follows:


(3) $$t\,t\,d_{i}=\beta_{0}+\beta_{1}\,L_{w\,i}+\beta_{2}\,S_{w\,i}+\beta_{3}\,V_{w\,i}+\beta_{4}\,S\,e\,x_{i}+\beta_{5}\,A\,g\,e_{i}+\beta_{6}\,E\,d\,u_{i}+\beta_{7}\,P\,h\,C_{i}+r_{i},\left(3\right)$$

where *ttd*_*i*_ denotes time-to-death at baseline, *Edu*_i_ denotes the education score and *PhC*_i_ denotes the physical condition included to control for between-person differences in objective health status at baseline, which was modeled as latent variable derived from the baseline measures of handgrip strength and chair-stand performance:


(4) $$H\,a\,n\,d\,g\,r\,i\,p_{i}=\mu_{1}+P\,h\,C_{i}+\varepsilon_{1\,i}\;\left(4\right)$$

(5) $$C\,h\,a\,i\,r\,S\,\tan\,d_{i}=\mu_{2}+\lambda_{2}\,P\,h\,C_{i}+\varepsilon_{2\,i}\;\left(5\right)$$

We ran this model in two ways, namely, for the single analysis involving each of the seven wellbeing indicators separately (as formulated in the above equations) and for a joint analysis involving all wellbeing indicators simultaneously. Thus, this simultaneous model includes seven within-person model equations, one for each indicator *W*, and an enlarged survival submodel equation, including the levels, slopes, and detrended variances of all seven indicators as predictors (summing up to altogether 25 predictors). The single model serves to reveal the overall associations of each indicator with time-to-death, whereas the simultaneous model targets an “integrative” analysis of the unique mortality effects of each facet of wellbeing, over and above the possibly confounding effects of the other wellbeing indicators.

The analyses were run with Mplus (Version 8.5; Muthén and Muthén, 1998-2017). To compute the multilevel SEM, Mplus requires to use the “Bayes estimator,” that is, Bayes estimation employing Markov Chain Monte Carlo (MCMC) algorithms (for details see Asparouhov and Muthén, 2010; McNeish, 2020). Note, thus, that we did not use Bayes estimation from a truly Bayesian perspective, aiming to utilize prior information about the model parameters, but rather took it “as a computational tool for getting estimates that are analogous to what would have been obtained by ML had it been feasible” (Muthén and Asparouhov, 2012, p. 314). Lacking solid prior information, we used the Mplus default diffuse priors for all parameters, except for the following: We specified prior distributions for the fixed levels and linear slopes of time-in-study-related trajectories of the wellbeing indicators by using the respective growth curve estimates reported in Wettstein et al. (2015). In that previous examination of LateLine data, which at that time included only the first 7 measurement occasions, we had already estimated time-in-study-related linear growth curve models for all wellbeing indicators used in this study, therefore, these estimates might be used as a reasonable guess about the distribution of the respective parameters in our current models. That is, for each wellbeing indicator, the prior distribution for the between-person means of the level and the slope was the normal distribution *N*(*M_p_, V_p_*), where *M*_p_ was the respective fixed level or slope and *V*_p_ the random variance estimated in that previous study (see Wettstein et al., 2015, p. 9: Table 4). However, using diffuse priors for the majority of the model parameters, it might be concerned whether our sample size is large enough for accurate estimation in particular regarding the simultaneous estimation of multiple parameters with the full model including all wellbeing indicators (Smid et al., 2020; Zitzmann et al., 2021). Zitzmann and Hecht (2019) showed that the accuracy of the Mplus Bayes estimation can be enhanced by increasing the precision of the MCMC method by adjustment of the effective sample size (ESS).^2^ Therefore, we followed their recommendation to set ESS = 400 to receive “estimates of good statistical quality,” which is applied in Mplus by setting the Potential Scale Reduction (PSR) cut-off for convergence to PSR = 0.00125.

It should also be noted that with regard to missing data treatment, the MCMC algorithm provides estimation unbiased under missing-at-random. Finally, it may also be noted, that in the multilevel SEM we took some marginal coarseness, in right-censoring time-to-death for two participants who still were alive (and centenarians) by the time of conducting these analyses^3^.

## Results

Descriptive information – means, SDs, and bivariate correlations – about the study variables used in the survival analyses are provided in Table 2. We computed between-person and within-person correlations for the time-varying variables by running random-intercept-only multilevel models to disentangle person means (i.e., the random intercepts) and person-mean-centered scores (i.e., the residuals). Between-person correlations thus denote correlations between person means, whereas within-person correlations denote correlations between the person-mean-centered scores.

**TABLE 2:** Means, SDs, and within-person and between-person correlations of study variables.

	M	SD	Correlations^a^									
			AUT	EMA	PIL	SAC	SWL	PA	NA	TTD	EDU	AGE
Autonomy (AUT)	3.946	0.501		**0.376**	–0.001	**0.343**	0.046	0.063	–0.007	–	–	–
Environmental Mastery (EMA)	3.916	0.539	**0.348**		**0.174**	**0.562**	**0.281**	**0.315**	**–0.152**	–	–	–
Purpose in Life (PIL)	3.044	0.532	0.015	**0.388**		**0.176**	**0.093**	**0.218**	–0.064	–	–	–
Self-Acceptance (SAC)	3.967	0.566	**0.261**	**0.746**	**0.313**		**0.336**	**0.261**	**–0.202**	–	–	–
Satisfaction with Life (SWL)	3.688	0.770	0.147	**0.692**	**0.388**	**0.797**		**0.261**	**–0.131**	–	–	–
Positive Affect (PA)	3.106	0.618	0.135	**0.533**	**0.552**	**0.496**	**0.574**		**–0.148**	–	–	–
Negative Affect (NA)	2.100	0.591	**–0.332**	**–0.480**	0.071	**–0.395**	**–0.397**	–0.065		–	–	–
Time-to-Death^b^ (TTD)	5.519	3.218	**–0.191**	0.015	–0.075	–0.028	0.079	0.074	–0.048		–	–
Education (EDU)	12.387	2.968	0.114	0.055	0.173^+^	0.031	**–0.189**	–0.031	–0.093	**–0.215**		–
Age at Baseline (AGE)	90.106	2.895	–0.096	–0.099	–0.153^+^	–0.038	–0.058	–0.018	0.046	**–0.198**	–0.130	
Sex female^c^	98	79%	0.010	–0.055	–0.046	0.015	0.053	0.010	0.026	0.097	0.108	0.058

*^a^Between-person correlations below diagonal, within-person correlations above diagonal, correlations in bold print significant with p < 0.05, ^+^ p < 0.10.*

*^b^Time-to-death at baseline right centered, including 2 study survivors.*

*^c^Number and percentage of female participants in M and SD (standard deviation) columns, respectively.*

Regarding the correlations, several aspects are remarkable: First, among the wellbeing indicators, autonomy seems to change within persons most independently from all other indicators, with substantive within-person correlations with environmental mastery and self-acceptance but only slight correlations with the hedonic indicators, and being virtually independent from purpose in life. In contrast, nearly all other within-person correlations (except purpose in life with life satisfaction and with NA) show at least substantive effect sizes (*r* > |0.10|). Second, again autonomy shows some specificity, compared to all other wellbeing indicators, with a significant negative correlation with time-to-death of substantive effect size, whereas none of the other wellbeing indicators showed any substantive correlation to time-to-death at baseline. Aside from wellbeing, a negative correlation of education with time-to-death seems notable, indicating that more years of education were associated with shorter survival in this oldest-old sample, apparently a “paradoxical” effect, which might be expected reversed in younger samples.

To examine the overall associations of each wellbeing indicator with time-to-death, we ran single analyses of the multilevel SEM, each including the level, slope, and variance components of each wellbeing indicator separately, as described above in the section “Materials and Methods”. The results relevant to this research question – i.e., the estimates of the regression of time-to-death on level, slope, and variance of wellbeing (and on the covariates age at baseline, sex, education, and physical condition, measured *via* baseline handgrip and chair stand performance) – are shown for the eudaimonic indicators in Table 3 and for hedonic indicators in Table 4. Note that in Bayes estimation with the MCMC method there are, in a strict sense, no null hypotheses, and therefore no *p*-values indicating “significance” – significance of a parameter refers to a 95% credible interval not including zero, which might be taken as two-sided test with *p* < 0.05. Only two components of one wellbeing indicator revealed significant effects on survival, namely, level and detrended variability of autonomy. The signs of these effects indicate that entering the study with higher autonomy levels predicted shorter time-to-death, whereas greater detrended variability of autonomy appears predictive of longer survival. In contrast, the linear trend (i.e., the average rate of change per time unit) in autonomy was not significantly associated with the distance to death. No other wellbeing component revealed any significant predictive effect on time-to-death. Moreover, the standardized regression coefficients of level (-0.315) and detrended variance (0.399) in autonomy show relatively strong effects in comparison with those of other wellbeing facets, which mostly appear considerably lower. Notable exceptions are the standardized coefficients of the detrended variances of environmental mastery (0.324) and NA (0.367), both indicating again that greater detrended variability predicts longer survival time, though these effects were not significant. Also, using the single *R*^2^s as a descriptive indicator of time-to-death variance “explained” by the respective model (note that *R*^2^ obtained from Bayes estimation should not be interpreted as a kind of population estimate, but specific to the model and sample under study), comparison among the values shown in Tables 3, 4 further points at the pronounced predictive effects of autonomy components on time-to-death (*R*^2^ = 0.52, followed by environmental mastery and NA, *R*^2^ = 0.47 and *R*^2^ = 0.46).

**TABLE 3:** Multilevel SEM between-person regressions of time-to-death at baseline on autonomy, environmental mastery, purpose in life, and self-acceptance.

		Estimate	Posterior SD	95% CI	Standard. estimate
**Autonomy**	Level	−2.178*	0.977	–4.275– –0.449	–0.315
	Slope	–4.847	16.634	–37.363–27.912	–0.034
	Variance	2.064*	0.955	0.450–4.199	0.399
Age		−0.254*	0.094	–0.436– –0.069	–0.129
Sex		1.693*	0.721	0.311–3.126	0.149
Education		−0.222*	0.090	–0.402– –0.045	–0.143
Physical Condition		0.574*	0.359	0.269–1.578	0.495
Intercept		18.646*	5.310	9.734–30.548	5.654
*R* ^2^		0.522*	0.125	0.269–0.756	

**Environ. Mastery**	Level	0.744	0.833	–0.866–2.432	0.118
	Slope	10.239	16.820	–22.607–43.323	0.077
	Variance	2.368	1.650	–0.489–5.748	0.324
Age		−0.224*	0.095	–0.411– –0.037	–0.130
Sex		1.643	0.732	0.237–3.124	0.146
Education		−0.290*	0.092	–0.470– –0.109	–0.187
Physical Condition		0.563*	0.446	0.223–1.796	0.459
Intercept		7.187	4.759	–0.936–17.386	2.187
*R* ^2^		0.465*	0.130	0.223–0.724	

**Purpose in Life**	Level	0.278	0.672	–1.043–1.601	0.054
	Slope	0.410	19.204	–37.719–37.669	0.003
	Variance	0.786	0.862	–0.977–2.423	0.156
Age		−0.230*	0.096	–0.419– –0.042	–0.133
Sex		1.688*	0.749	0.264–3.205	0.148
Education		−0.272*	0.094	–0.455– –0.088	–0.173
Physical Condition		0.690*	0.882	0.289–3.264	0.516
Intercept		6.250*	2.342	1.716–10.914	1.883
*R* ^2^		0.423*	0.153	0.191–0.787	

**Self Acceptance**	Level	0.473	0.772	–1.047–2.021	0.076
	Slope	4.965	17.908	–30.545–39.577	0.036
	Variance	0.732	1.022	–1.253–2.794	0.133
Age		−0.266*	0.096	–0.415– –0.038	–0.131
Sex		1.663*	0.747	0.237–3.176	0.146
Education		−0.256*	0.094	–0.440– –0.072	–0.163
Physical Condition		0.690*	0.743	0.295–2.622	0.522
Intercept		5.425	2.814	–0.085–11.036	1.638
*R* ^2^		0.417*	0.149	0.188–0.767	

Results from Mplus MCMC Bayes estimation (Asparouhov and Muthén, 2010). * indicates “significant” effect – i.e., the 95% credible interval does not include 0.

**TABLE 4:** Multilevel SEM between-person regressions of time-to-death at baseline on life satisfaction, positive affect, and negative affect.

		Estimate	Posterior SD	95% CI	Standard. estimate
**Life Satisfaction**	Level	0.304	0.527	–0.729–1.356	0.071
	Slope	–5.266	18.198	–40.111–31.313	–0.038
	Variance	0.196	0.965	–1.772–2.016	0.037
Age		−0.230*	0.095	–0.418– –0.043	–0.133
Sex		1.646*	0.742	0.211–3.147	0.145
Education		−0.278*	0.094	–0.461– –0.093	–0.177
Physical Condition		0.708*	0.579	0.295–2.447	0.513
Intercept		4.907*	1.986	1.022–8.864	1.579
*R* ^2^		0.403*	0.154	0.181–0.776	

**Positive Affect**	Level	0.431	0.662	–0.830–1.768	0.076
	Slope	13.729	16.935	–19.928–46.588	0.102
	Variance	–0.663	1.016	–2.807–1.249	0.121
Age		−0.238*	0.095	–0.425– –0.051	–0.140
Sex		1.559*	0.740	0.154–3.050	0.139
Education		−0.252*	0.092	–0.432– –0.073	–0.163
Physical Condition		0.639*	0.717	0.228–2.835	0.480
Intercept		2.827	3.705	–4.636–10.039	0.864
*R* ^2^		0.391*	0.149	0.178–0.752	

**Negative Affect**	Level	–1.330	1.042	–3.657–0.444	–0.238
	Slope	2.707	18.650	–33.928–39.217	0.018
	Variance	2.042	1.348	–0.319–4.992	0.367
Age		−0.244*	0.095	–0.430– –0.058	–0.142
Sex		1.529*	0.736	0.123–3.026	0.135
Education		−0.272*	0.092	–0.452– –0.092	–0.175
Physical Condition		0.627*	0.914	0.233–1.984	0.467
Intercept		13.781*	5.337	4.469–25.463	4.171
*R* ^2^		0.458*	0.130	0.220–0.721	

Results from Mplus MCMC Bayes estimation (Asparouhov and Muthén, 2010). * indicates “significant” effect – i.e., the 95% credible interval does not include 0.

Among the covariates included for statistical control, all appeared as significant predictors of time-to-death, with older age at baseline, female gender, less years of education, and worse physical condition at baseline predicting a shorter survival time in the oldest-old individuals. In terms of the standardized coefficients, the physical condition held the strongest effect on time-to-death among all predictors included in all models.

Table 5 shows the results obtained from the full simultaneous multilevel SEM, including all components (i.e., levels, slopes, and detrended within-person variability) of all wellbeing indicators as predictors of time-to-death at baseline. The full model serves to estimate the unique effects of the wellbeing indicators, controlling for their potential mutual confounding. With regard to the effects of the wellbeing indicators, only the coefficient of the level of autonomy was significant, again as in the single model analysis indicating that higher autonomy at baseline predicts shorter survival. Compared with the single analysis result, the standardized coefficient (–0.268) was reduced. The effect of detrended variance in autonomy was no longer significant. However, considering lowered power of testing the effects simultaneously with this extensive full model, it should be noticed that the standardized coefficient (0.227) outstands the unique effects of all other predictors, aside from level of autonomy and physical condition.

**TABLE 5:** Multilevel SEM between-person regression of time-to-death at baseline and full model including all predictors.

		Estimate	Posterior SD	95% CI	Standard. estimate
Autonomy	Level	−2.157*	1.136	–4.522– –0.042	–0.268
	Slope	–7.199	17.458	–41.746–26.814	–0.044
	Variance	1.425	1.090	–0.583–3.733	0.227
Environ. Mastery	Level	0.881	1.121	–1.321–3.092	0.122
	Slope	10.418	18.387	–25.817–46.391	0.067
	Variance	1.138	1.625	–2.069–4.395	0.127
Purpose in Life	Level	–0.097	0.878	–1.826–1.626	–0.016
	Slope	–1.854	19.188	–39.792–35.556	–0.012
	Variance	0.401	0.844	–1.297–2.037	0.069
Self Acceptance	Level	–0.257	1.102	–2.437–1.899	–0.036
	Slope	–0.070	19.287	–38.238–37.594	0.000
	Variance	0.184	1.006	–1.818–2.173	0.029
Life Satisfaction	Level	0.126	0.796	–1.408–1.717	0.026
	Slope	–1.397	18.526	–37.659–35.131	0.009
	Variance	0.026	0.912	–1.812–1.800	0.004
Positive Affect	Level	0.494	0.897	–1.231–2.290	0.075
	Slope	8.976	17.352	–25.181–43.072	0.058
	Variance	–0.691	0.943	–2.611–1.126	–0.106
Negative Affect	Level	–0.818	1.020	–2.983–1.040	–0.126
	Slope	1.903	18.670	–34.848–38.471	0.011
	Variance	0.958	1.257	–1.292–3.659	0.145
Age		−0.255*	0.108	–0.468– –0.043	–0.129
Sex		1.432	0.826	–0.144–3.109	0.110
Education		−0.248*	0.108	–0.461– –0.036	–0.138
Physical Condition		0.412*	0.390	0.076–1.313	0.316
Intercept		19.378*	8.657	2.922–37.202	5.093
*R* ^2^		0.623*	0.089	0.437–0.784	

Results from Mplus MCMC Bayes estimation (Asparouhov and Muthén, 2010). * indicates “significant” effect – i.e., the 95% credible interval does not include 0.

So far, we focused on the multilevel SEM results relevant with regard to our study aims (i.e., the estimates for the time-to-death regression submodel). For the sake of complete reporting, we showed the estimates for the within-person location-scale submodel – the fixed effects (between-person means) and random variances (between person-variances) – of the wellbeing indicators in Table 6. The estimates shown are from the full model, including all wellbeing indicators, the respective estimates from the single models did differ only marginally by decimals. As can be seen, all fixed linear slopes except for life satisfaction and NA were significant, indicating some general trend of decline of wellbeing over time. However, the random variances of the slopes obviously do only cover small proportions of interindividual differences in intraindividual wellbeing changes: “De-logarithmizing” the random variances of the detrended variance components to make these comparable with the slope variances shows that these are substantially larger [e.g., for autonomy: exp(0.357) = 1.429]. This may explain why all slope components were not predictive for time-to-death. The complete outcomes from running the SEM procedures can be found in detail in the Mplus outputs provided as Supplementary Material. Also, note that in another Supplementary Material, we presented results obtained from a two-step Cox regression procedure conducted just for comparison (but compromised by reliability concerns), which by and large confirm the effects of level and variability in autonomy on survival.

**TABLE 6:** Within-person location-scale model estimates of the fixed effects and random variances of level, linear slope, and detrended variance of the wellbeing indicators.

		Fixed Effect^a^		Random Variance^b^	
		Estimate	95% CI	Estimate	95% CI
Autonomy	Level	4.014*	3.914–4.116	0.224	0.147–0.332
	Slope	−0.005*	–0.008– –0.001	0.001	0.000–0.001
	LnVar	−2.019*	–2.266– –1.803	0.357	0.164–0.696
Environ. Mastery	Level	4.129*	4.015–4.244	0.275	0.183–0.403
	Slope	−0.011*	–0.017– –0.004	0.001	0.000–0.001
	LnVar	−1.913*	–2.131– –1.717	0.174	0.042–0.430
Purpose in Life	Level	3.242*	3.111–3.375	0.383	0.259–0.559
	Slope	−0.011*	–0.017– –0.005	0.001	0.000–0.001
	LnVar	−1.935*	–2.186– –1.704	0.425	0.206–0.818
Self Acceptance	Level	4.076*	3.960–4.187	0.283	0.189–0.416
	Slope	–0.004	–0.010–0.002	0.001	0.000–0.001
	LnVar	−2.234*	–2.452– –2.017	0.348	0.152–0.696
Life Satisfaction	Level	3.688*	3.525–3.848	0.595	0.417–0.852
	Slope	0.002	–0.005–0.008	0.001	0.000–0.001
	LnVar	−1.968*	–2.191– –1.751	0.390	0.181–0.744
Positive Affect	Level	3.314*	3.191–3.438	0.329	0.230–0.474
	Slope	−0.015*	–0.022– –0.010	0.001	0.000–0.001
	LnVar	−2.302*	–2.539– –2.074	0.340	0.134–0.687
Negative Affect	Level	2.110*	1.992–2.232	0.344	0.246–0.486
	Slope	–0.002	–0.008–0.004	0.000	0.000–0.001
	LnVar	−2.529*	–2.757– –2.324	0.326	0.131–0.683

*Full model (including all time-to-death predictors) results from Mplus MCMC Bayes estimation (Asparouhov and Muthén, 2010). LnVar, logarithm of the within-person detrended variance.*

*^a^Between-person mean.*

*^b^Between-person variance.*

* “significant” fixed effect – i.e., the 95% credible interval does not include 0 (credible intervals for variance estimates cannot include 0 with Bayes estimation).

## Discussion

Previous research provided evidence that hedonic and eudaimonic wellbeing adds to the prediction of all-cause mortality in midlife and old age (Martín-María et al., 2017). However, evidence on the role of both major wellbeing components for mortality in the specific life phase of fourth age is still scarce. As life circumstances and resources become qualitatively different in the final years of the life span (Baltes and Smith, 2003; Gerstorf and Ram, 2009; Wahl and Ehni, 2020), this may, for one, lead to reversed interrelations in which specific resource constellations being protective for health and survival in early old age may become dysfunctional under conditions of the fourth age (Schilling, 2016; Mueller et al., 2017). Also, it is possible that the role of wellbeing indicators established as predictors of mortality in the second half of life (Chida and Steptoe, 2008; Diener and Chan, 2011; Martín-María et al., 2017) may be weakened and overwritten in advanced old age by accumulated multi-domain resource loss. This loss might particularly be driven by biological and morbidity-driven processes, as – following the principle of the incomplete architecture of human ontogeny (Baltes, 1997) – “evolution and biology are not good friends of old age” (p. 368).

Our study comes with supporting evidence for both of these assumptions. First, although we included multiple indicators of both hedonic and eudaimonic wellbeing, which represent two theoretically grounded, major components of wellbeing (Ryan and Deci, 2001), most of them did not reveal a meaningful role as predictors of mortality. Specifically, no component of hedonic wellbeing (i.e., life satisfaction, PA, and NA), i.e., neither levels nor slopes nor detrended within-person variability in these indicators, was a significant predictor of time-to-death. Thus, it may be that, whereas hedonic wellbeing has been found to be significantly related to mortality in samples of middle-aged and older adults (Lyubomirsky et al., 2005; Martín-María et al., 2017; Fernández-Ballesteros and Sánchez-Izquierdo, 2019; Ong and Steptoe, 2020), this may no longer be the case when investigating exclusively the specific age group of very old adults as we did in this study.

The only predictor standing out among the wellbeing indicators was the eudaimonic indicator of autonomy. Importantly, these findings revealed an, at first glance, paradoxical role of autonomy, in which lower levels and higher within-person instability of perceived autonomy went along with longer survival time. According to prior findings, most of them not addressing specifically very old adults, harmful effects of wellbeing on longevity are the exception (Martín-María et al., 2017). However, this finding regarding the role of autonomy for late-life mortality did not come entirely unexpectedly. Conceptual and empirical research on the fourth age suggests that the role of resource constellations in this life period might change or reverse (Schilling, 2016; Mueller et al., 2017). Also, from a theoretical perspective, established theories, such as the motivational theory of life-span development (Heckhausen et al., 2010) or the dual-process model of coping (Brandtstädter, 2009), postulate that pronounced striving for agency may become dysfunctional in that life phase, whereas disengagement from goals (Heckhausen et al., 2010, 2013), accommodative processes (Brandtstädter, 2009; Brandtstädter et al., 2010), as well as “consciously accepted dependency” (Kruse, 2005) become increasingly adaptive. Adaptive processes are not limited to younger ages, and findings from resilience research suggest that “well into old age, the self is able to maintain relatively high levels of adaptation” (Staudinger and Fleeson, 1996, Abstract).

This theoretical background might to some extent explain why lower autonomy at baseline and higher detrended within-person variability in autonomy were associated with a longer survival time. According to SDT (Deci and Ryan, 2002), autonomy is one of the three fundamental and universal psychological needs. Ryff (1989) described an individual with high autonomy scores as “self-determining and independent; […]; regulates behavior from within” (p. 1072). Similarly, according to Deci and Ryan (2008), autonomy refers to “volition, to having the experience of choice, to endorsing actions of individuals at the highest level of reflection” (p. 6). With regard to very old age, accepting restrictions in the experience of choice, for instance, due to physical vulnerability, might be adaptive, whereas endorsing a high – potentially illusory – experience of choice might not reflect factual life circumstances and become dysfunctional over time (see also Neubauer et al., 2015).

Heightened intraindividual variability has often been found to indicate vulnerability and instability, with more fluctuations – e.g., in the domain of cognitive functioning (MacDonald et al., 2008), but also in affective wellbeing (Ong and Steptoe, 2020) – being associated with higher mortality risk. However, within-person variability is not necessarily always maladaptive (Siegler, 1994; MacDonald and Stawski, 2015), depending on the specific outcome and on the kind of variability. Our findings suggest that higher within-person changeability in autonomy might reflect adaptive processes of flexibly adjusting one’s perceived autonomy to changing life circumstances and changing individual resources in very old age. However, more studies are required regarding which factors drive within-person variability of wellbeing in very old age and under which circumstances such variability is adaptive or not.

Furthermore, although such an interpretation of an adaptive role of low autonomy as well as of high within-person variability of autonomy in very old age seems plausible and might be an important addition to the previous mortality research considering wellbeing, caution is certainly in place, and additional replication of our exploratory research in independent samples that are larger and more representative than our rather small study sample is definitely needed. Also, our findings should not be overgeneralized and interpreted as evidence suggesting that higher within-person variability and lower levels across *all* wellbeing domains are associated with longer survival time in the oldest-old individuals. Rather, these effects appeared to apply specifically to the autonomy component. Regarding the other wellbeing indicators, an effect of the within-person variability may be seen “tentatively” only in the results from the separate analyses of environmental mastery and NA (with standardized coefficients suggesting a substantially sized, though insignificant, effect), but neither so in the other wellbeing components studied nor in the unique effects estimated simultaneously in the full model analysis. The “counterintuitive” effect of the autonomy level was not even tentatively found for any other wellbeing indicator. Thus, our pattern of findings speaks against a generalized view of wellbeing in old age and rather reflects the multidimensionality of the wellbeing construct. Therefore, it might depend on the specific eudaimonic wellbeing domain considered whether higher wellbeing and higher intraindividual variability in wellbeing are related to a shorter or longer survival time in very old age.

More needs to be known with regard to how levels, changes, and within-person variations in autonomy – as well as in other wellbeing – are associated with time-to-death. Mediating mechanisms as well as factors triggering such within-person variations should be identified by future research. Also, in line with the debate that intraindividual variation can be either adaptive or maladaptive (MacDonald and Stawski, 2015), subgroups should be investigated which benefit from higher intraindividual wellbeing variability versus for whom such variations are rather harmful. For instance, very old individuals whose health remains stable over time and who do not have to face major changes in life conditions may not need to adjust their autonomy over time, so that for these persons, high intraindividual variability in autonomy over time might rather indicate vulnerability and be maladaptive. In contrast, individuals experiencing health declines or other occurrences of resource loss might benefit from flexibly adjusting their autonomy, and for these individuals, such flexible adjustments could be a means to promote survival (see, e.g., the Lines-of-Defense model proposed by Heckhausen et al., 2013). Finally, low levels of autonomy and greater intraindividual fluctuations in autonomy might be adaptive for survival among the oldest-old individuals, but not necessarily for other developmental outcomes (such as health or functional ability). These potential adaptive effects require further research.

## Limitations

Although we had a sample available that provided an impressive amount of up to 16 repeated measures over 7 years, our overall sample was limited in size and of restricted representativeness, comprising only very old adults living alone (although most very old adults live alone; Wahl and Heyl, 2015) and without (severe) cognitive impairment. Thus, regarding the sample we analyzed, the findings should not be generalized without caution and need replication and confirmation by future research.

Also, the sample size could be considered as a limitation of statistical power (from a frequentist perspective). However, on the one hand, taking into account the multilevel data structure, having an average number of 5.5 observations clustered within 124 individuals – i.e., more than 600 observations in total – can be considered as sufficient to detect effects, in particularly using Bayes estimation, which is not restricted by sample size requirements in the same way as frequentist maximum likelihood estimation. On the other hand, Bayes estimation may lack accuracy when conducted with small samples and diffuse priors (Smid et al., 2020; Zitzmann et al., 2021). To deal with this problem, we applied the recommendations to increase accuracy proposed by Zitzmann and Hecht (2019), as explained in the section “Materials and Methods”.

Some of the measures were of low psychometric quality. For instance, the internal consistency (Cronbach’s α) of purpose in life was rather low, although this might also be due to the content heterogeneity of the purpose items and not necessarily due to the low reliability of the scale.

Finally, given our already quite complex analyses comprising three components per wellbeing indicator (i.e., level, slope, and detrended within-person variability) and the sample size concerns as noted above, we refrained from further in-depth analyses, for instance of mediating effects linking wellbeing and survival, or of moderators with regard to the described predictor effects of autonomy. Also, the LateLine study on which we based our analysis only contained a subset of indicators of Ryff’s eudaimonic wellbeing concept (Ryff and Keyes, 1995) in order to reduce the burden for the very old study participants. Thus, the role of two additional eudaimonic wellbeing indicators (positive relations with others and personal growth) for late-life all-cause mortality could not be empirically addressed based on the available data.

## Conclusion

In this study, we investigated the role of multiple wellbeing indicators for survival in the oldest-old individuals. Lower autonomy at baseline and higher intraindividual variability in autonomy were associated with a longer survival time, controlling for age, gender, education, and the physical condition of participants at the study baseline. However, in contrast to earlier life phases such as midlife or early old age, hedonic wellbeing does, according to our findings, not play a major role as a predictor of time-to-death in very old age. Our findings suggest that there is a “paradoxical” association of autonomy and of within-person variability in autonomy with mortality in advanced old age. Further research is needed to explore the underlying mechanisms linking autonomy and late-life mortality. One potential mechanism which needs to be further investigated is that intraindividual variability in autonomy reflects processes of flexible adjustments, in reaction to changing life circumstances and available resources, which might be adaptive and promote survival among the oldest-old individuals.

## Data Availability Statement

The raw data supporting the conclusions of this article will be made available by the authors, without undue reservation.

## Ethics Statement

The studies involving human participants were reviewed and approved by Institutional Ethics Review Board of Heidelberg University’s Faculty of Behavioral and Cultural Studies. The patients/participants provided their written informed consent to participate in this study.

## Author Contributions

OS computed all statistical analyses, wrote the “Results” section, and was involved in writing all other parts of the manuscript. MW and H-WW conceptualized the study (“Introduction” section), wrote the “Discussion” section, and were involved in writing all other parts of the manuscript.

## Conflict of Interest

The authors declare that the research was conducted in the absence of any commercial or financial relationships that could be construed as a potential conflict of interest.

## Publisher’s Note

All claims expressed in this article are solely those of the authors and do not necessarily represent those of their affiliated organizations, or those of the publisher, the editors and the reviewers. Any product that may be evaluated in this article, or claim that may be made by its manufacturer, is not guaranteed or endorsed by the publisher.

## Footnotes

1 The conceptual distinction between more long-term change trends and more short-term variability around the trend curve is not novel in aging research. Nesselroade (1991) highlighted the distinction between intraindividual change—“more or less enduring changes that are construed as developmental”—and intraindividual variability—“relatively short-term changes that are construed as more or less reversible and that occur more rapidly than the former” (p. 215). We did, however, not use his terminology, as it might be mixed up (change is part of within-person variability), and confuse readers.

2 Precision indicates how close the summary statistic computed in a MCMC chain approximates the respective expected posterior (i.e., the Bayesian estimates), and the ESS is the number of independent draws from the posterior distribution used to compute this MCMC summary statistic. As more elaborate explanations of the statistical terms used in this section would go beyond the scope of this article, we refer to Zitzmann and Hecht (2019) for further details. We also thank an reviewer for pointing to this publication and strategy to enhance estimation accuracy.

3 Notably, at present, statistical software packages enabling the estimation of multilevel location scale models (McNeish, 2020) do not allow for widely used survival analyses, such as Cox proportional hazard models at the second level. To check and compare whether our findings would hold in a more conventional survival analysis, we additionally ran two-step analyses, which are, however, compromised by reliability issues. These are briefly presented in the Supplementary Material.
