# Supplementary material for: Associations of Wellbeing Levels, Changes, and Within-Person Variability With Late-Life All-Cause Mortality Across 12 Years: Contrasting Hedonic vs. Eudaimonic Wellbeing Among Very Old Adults
Source: Front Psychol. 2022 Jan 11;12:750891. doi: 10.3389/fpsyg.2021.750891 (PMC8787264; doi:10.3389/fpsyg.2021.750891)
Supplement: Supplementary file 1 [file Data_Sheet_1.PDF]

Associations of Well-Being Levels, Changes and Within-Person Variability with Late-Life All-Cause Mortality Across 12 Years: Contrasting Hedonic vs. Eudaimonic Well-Being Among Very Old Adults

Oliver Schilling, Markus Wettstein, Hans-Werner-Wahl

Online Supplement: Mplus Outputs

Mplus outputs for all multilevel SEM analyses are printed on the following pages:

p. 2: Single model including within-person model for autonomy
p. 12: Single model including within-person model for environmental mastery
p. 27: Single model including within-person model for purpose in life
p. 53: Single model including within-person model for self acceptance
p. 79: Single model including within-person model for life satisfaction
p. 94: Single model including within-person model for positive affect
p. 109: Single model including within-person model for negative affect
p. 143: Full model including within-person model for all well-being indicators

Outputs have been shortened a bit to reduce length of this supplement – in particular, we deleted „technical 1“ and „technical 16“ outputs.

Mplus Output Single Multilevel SEM – Autonomy:

```
Mplus VERSION 8.5
MUTHEN & MUTHEN
10/12/2021   3:33 PM

INPUT INSTRUCTIONS

  TITLE:

  DATA: FILE = mpltry6.dat;

  VARIABLE:
   names =  pno wave sex yol verst nopart
         basagec90 agec90 tims tsbase
         ttd ttdcens basettd basttdc
         aut pil ema sac dep
         pa na swls adlinv hyp
         visus sfenerg sfsofunc sfpain sfgen sfchang
         anges anget akzet mortinv mortperc
         basadlinv basvisus bashkr bassssc
         bassfen bassfso bassfpain bassfgen bassfcha
         hkr sl sssco;
  usevariables = aut tsbase basttdc bashkr bassssc basagec90 sex yol;
  cluster = PNO;
  missing = .;
  within = tsbase;
  between = basttdc bashkr bassssc basagec90 sex yol;

 DEFINE:
  center sex yol (grandmean);

 ANALYSIS:
  type = twolevel random;
  estimator = BAYES;
  chains = 2;
  biterations=1000000;
  BCONVERGENCE = 0.00125;

 MODEL:

  %WITHIN%

  slopeaut | aut ON tsbase;
  lnvaut | aut;

  %BETWEEN%

  physcon BY bashkr@1;
  physcon BY bassssc*0.36035;

  basttdc ON
   aut*-2.53474
   sex*2.22897
   yol*-0.15840
   basagec90*-0.17061
   physcon*0.64851
   slopeaut*7.00908
   lnvaut*1.81797;

  aut WITH slopeaut*-0.00064;
  aut WITH lnvaut*0.11350;
  slopeaut WITH lnvaut*-0.00053;

  [ basttdc*19.68536 ];
  [ bashkr*18.59694 ];
  [ bassssc*1.62528 ];
  [ aut*4.03331 ] (m1);
  [ slopeaut*-0.00564 ] (ms1);
  [ lnvaut*-2.05118 ] (mv1);

  basttdc*5.33211;
```

```
  bashkr*36.53531;
  bassssc*1.98520;
  aut*0.27312;
  physcon*6.84194;
  slopeaut*0.00042;
  lnvaut*0.33703;

 MODEL PRIORS:
  m1 ~ N(3.98,0.048);
  ms1 ~ N(-0.006,0.000004);
 OUTPUT:
  standardized tech5 tech8 tech16;




*** WARNING in VARIABLE command
  Note that only the first 8 characters of variable names are used in the output.
  Shorten variable names to avoid any confusion.
*** WARNING in MODEL command
  In the MODEL command, the following variable is an x-variable on the BETWEEN
  level and a y-variable on the WITHIN level.  This variable will be treated
  as a y-variable on both levels:  AUT
*** WARNING
  One or more individual-level variables have no variation within a
  cluster for the following clusters.

     Variable   Cluster IDs with no within-cluster variation

      AUT         10130 11297 16142 16798 11258 15175

   3 WARNING(S) FOUND IN THE INPUT INSTRUCTIONS




SUMMARY OF ANALYSIS

Number of groups                                                 1
Number of observations                                         682

Number of dependent variables                                    4
Number of independent variables                                  4
Number of continuous latent variables                            3

Observed dependent variables

  Continuous
   BASTTDC     BASHKR      BASSSSC     AUT

Observed independent variables
   TSBASE      BASAGEC9    SEX         YOL

Continuous latent variables
   PHYSCON     SLOPEAUT    LNVAUT

Variables with special functions

  Cluster variable      PNO

  Within variables
   TSBASE

  Between variables
   BASTTDC     BASHKR      BASSSSC     BASAGEC9    SEX         YOL

  Centering (GRANDMEAN)
   SEX         YOL


Estimator                                                    BAYES
Specifications for Bayesian Estimation
  Point estimate                                            MEDIAN
```

```
  Number of Markov chain Monte Carlo (MCMC) chains              2
  Random seed for the first chain                               0
  Starting value information                          UNPERTURBED
  Algorithm used for Markov chain Monte Carlo           GIBBS(PX1)
  Convergence criterion                                 0.125D-02
  Maximum number of iterations                            1000000
  K-th iteration used for thinning                              1

Input data file(s)
  mpltry6.dat
Input data format  FREE


SUMMARY OF DATA

     Number of clusters                        124

       Size (s)    Cluster ID with Size s

          1        10026 10130 10144 10313 10473 10558 10567 10637 10663
                   10893 10901 10907 10911 10939 10969 11157 11240 11294
                   11297 11741 15018 15177 15241 15319 15564 16142 16423
                   16610 16669 16798 17542 18507 18650
          2        15239 11258 10181 15510 10615 11341 16303 11371 16510
                   11384 11485 11028 16862 10211 17780 18004 18252 15175
                   10228
          3        11573 11295 17130 11100 17745 10340 10460 10461 10210
                   11501
          4        10904 11457 10434 11493 10811 17593
          5        15424 10666 12411 11343 11005
          6        10159 15092 15121 15426 11593 11163 10139 16220 19067
          7        10354 11230 10894 10724 10470
          8        10252 10528 11038 10033 11336 11253
          9        15623 11055
         10        12037 10167 17197 15009 19500
         11        11233 10444 15714 10533
         12        11079 10577 15378 12714 10986 10428
         13        11420 11002
         14        11450
         15        10108 10906 10551 15231 10902
         16        15141 10940 16103 15472 11528 11378



COVARIANCE COVERAGE OF DATA

Minimum covariance coverage value   0.100

     Number of missing data patterns             7


     PROPORTION OF DATA PRESENT
```

Covariance Coverage

	BASTTDC	BASHKR	BASSSSC	AUT	TSBASE
BASTTDC	0.944				
BASHKR	0.912	0.968			
BASSSSC	0.935	0.968	0.991		
AUT	0.921	0.949	0.971	0.977	
TSBASE	0.944	0.968	0.991	0.977	1.000
BASAGEC9	0.944	0.968	0.991	0.977	1.000
SEX	0.944	0.968	0.991	0.977	1.000
YOL	0.944	0.968	0.991	0.977	1.000

Covariance Coverage

	BASAGEC9	SEX	YOL
BASAGEC9	1.000		
SEX	1.000	1.000	
YOL	1.000	1.000	1.000

UNIVARIATE SAMPLE STATISTICS

UNIVARIATE HIGHER-ORDER MOMENT DESCRIPTIVE STATISTICS

Variable/ Sample Size	Mean/ Variance	Skewness/ Kurtosis	Minimum/ Maximum	% with Min/Max	Percentiles 20%/60%	40%/80%	Median
BASTTDC	5.519	0.456	0.250	0.83%	2.250	4.250	5.083
120.000	10.267	-0.614	12.667	1.67%	6.083	8.250	
BASHKR	18.519	1.282	5.330	0.89%	12.330	16.500	17.750
112.000	48.308	2.780	49.000	0.89%	18.670	23.330	
BASSSSC	1.333	0.640	0.000	53.33%	0.000	0.000	0.000
120.000	2.606	-1.294	4.000	17.50%	1.000	3.000	
AUT	3.892	-0.192	2.222	0.30%	3.333	3.778	3.889
666.000	0.344	-0.528	5.000	2.70%	4.111	4.444	
TSBASE	36.711	0.258	0.000	3.08%	13.000	27.000	36.000
682.000	594.554	-0.917	91.000	0.59%	41.000	60.000	
BASAGEC90	0.106	0.407	-3.917	0.81%	-2.833	-1.250	-0.792
124.000	8.313	-1.090	6.417	0.81%	0.500	3.083	
SEX	0.000	-1.426	-0.790	20.97%	-0.790	0.210	0.210
124.000	0.166	0.035	0.210	79.03%	0.210	0.210	
YOL	0.000	0.868	-3.387	20.97%	-3.387	-1.387	-1.387
124.000	8.737	-0.315	5.613	17.74%	0.613	0.613	

THE MODEL ESTIMATION TERMINATED NORMALLY

USE THE FBITERATIONS OPTION TO INCREASE THE NUMBER OF ITERATIONS BY A FACTOR OF AT LEAST TWO TO CHECK CONVERGENCE AND THAT THE PSR VALUE DOES NOT INCREASE.

MODEL FIT INFORMATION

Number of Free Parameters 24

Information Criteria

Deviance (DIC)	2751.296
Estimated Number of Parameters (pD)	233.194

MODEL RESULTS

	Estimate	Posterior S.D.	One-Tailed P-Value	95% C.I. Lower 2.5%	Upper 2.5%	Significance
**Within Level**						
**Between Level**						
PHYSCON BY						
BASHKR	1.000	0.000	0.000	1.000	1.000	
BASSSSC	0.381	0.323	0.000	0.157	1.371	*
BASTTDC ON						
PHYSCON	0.574	0.359	0.000	0.269	1.578	*
SLOPEAUT	-4.847	16.634	0.382	-37.363	27.912	
LNVAUT	2.064	0.955	0.007	0.450	4.199	*
BASTTDC ON						
AUT	-2.178	0.977	0.007	-4.275	-0.449	*
SEX	1.693	0.721	0.009	0.311	3.126	*
YOL	-0.222	0.090	0.008	-0.402	-0.045	*
BASAGEC90	-0.254	0.094	0.004	-0.436	-0.069	*
AUT WITH						
SLOPEAUT	-0.003	0.002	0.034	-0.006	0.000	
LNVAUT	0.118	0.062	0.026	-0.002	0.246	

```
 SLOPEAUT WITH
    LNVAUT            -0.003       0.003      0.133      -0.008       0.002

 Means
    AUT                4.014       0.051      0.000       3.915       4.117      *
    SLOPEAUT          -0.005       0.002      0.004      -0.008      -0.001      *
    LNVAUT            -2.093       0.126      0.000      -2.355      -1.858      *

 Intercepts
    BASTTDC           18.646       5.310      0.000       9.734      30.458      *
    BASHKR            18.466       0.673      0.000      17.143      19.768      *
    BASSSSC            1.329       0.152      0.000       1.028       1.628      *

 Variances
    AUT                0.227       0.047      0.000       0.151       0.334      *
    PHYSCON            8.223       5.911      0.000       0.906      23.453      *
    SLOPEAUT           0.001       0.000      0.000       0.000       0.001      *
    LNVAUT             0.390       0.145      0.000       0.193       0.751      *

 Residual Variances
    BASTTDC            5.170       1.455      0.000       2.663       8.331      *
    BASHKR            41.931       7.484      0.000      28.345      57.932      *
    BASSSSC            1.504       0.579      0.000       0.141       2.483      *


STANDARDIZED MODEL RESULTS


STDYX Standardization

                                Posterior  One-Tailed         95% C.I.
                    Estimate       S.D.      P-Value   Lower 2.5%  Upper 2.5%  Significance

Within-Level Standardized Estimates Averaged Over Clusters

 SLOPEAUT | AUT ON
    TSBASE            -0.063       0.024      0.003      -0.109      -0.014      *

 LNVAUT |
    AUT                0.878       0.015      0.000       0.847       0.905      *

Between Level

 PHYSCON  BY
    BASHKR             0.404       0.130      0.000       0.137       0.653      *
    BASSSSC            0.664       0.152      0.000       0.374       0.974      *

 BASTTDC    ON
    PHYSCON            0.495       0.117      0.000       0.270       0.723      *
    SLOPEAUT          -0.034       0.116      0.382      -0.258       0.194
    LNVAUT             0.399       0.162      0.007       0.081       0.711      *

 BASTTDC    ON
    AUT               -0.315       0.141      0.007      -0.617      -0.064      *
    SEX                0.149       0.061      0.009       0.028       0.267      *
    YOL               -0.143       0.057      0.008      -0.253      -0.029      *
    BASAGEC90         -0.148       0.054      0.004      -0.251      -0.040      *

 AUT      WITH
    SLOPEAUT          -0.244       0.127      0.034      -0.477       0.020
    LNVAUT             0.410       0.192      0.026      -0.005       0.734

 SLOPEAUT WITH
    LNVAUT            -0.183       0.156      0.133      -0.470       0.139

 Means
    AUT                8.415       0.864      0.000       6.950      10.313      *
    SLOPEAUT          -0.200       0.077      0.004      -0.355      -0.051      *
    LNVAUT            -3.357       0.543      0.000      -4.585      -2.461      *

 Intercepts
    BASTTDC            5.654       1.584      0.000       2.962       9.159      *
    BASHKR             2.589       0.200      0.000       2.199       2.985      *
    BASSSSC            0.806       0.106      0.000       0.596       1.016      *
```

```
 Variances
    AUT                1.000       0.000      0.000       1.000       1.000
    PHYSCON            1.000       0.000      0.000       1.000       1.000
    SLOPEAUT           1.000       0.000      0.000       1.000       1.000
    LNVAUT             1.000       0.000      0.000       1.000       1.000

 Residual Variances
    BASTTDC            0.478       0.125      0.000       0.243       0.730      *
    BASHKR             0.837       0.106      0.000       0.574       0.981      *
    BASSSSC            0.559       0.206      0.000       0.051       0.860      *


STDY Standardization

                                Posterior  One-Tailed         95% C.I.
                    Estimate       S.D.      P-Value   Lower 2.5%  Upper 2.5%  Significance

Within-Level Standardized Estimates Averaged Over Clusters

 SLOPEAUT | AUT ON
    TSBASE            -0.006       0.007      0.190      -0.019       0.008

 LNVAUT |
    AUT                0.878       0.015      0.000       0.847       0.905      *

Between Level

 PHYSCON  BY
    BASHKR             0.404       0.130      0.000       0.137       0.653      *
    BASSSSC            0.664       0.152      0.000       0.374       0.974      *

 BASTTDC    ON
    PHYSCON            0.495       0.117      0.000       0.270       0.723      *
    SLOPEAUT          -0.034       0.116      0.382      -0.258       0.194
    LNVAUT             0.399       0.162      0.007       0.081       0.711      *

 BASTTDC    ON
    AUT               -0.315       0.141      0.007      -0.617      -0.064      *
    SEX                0.513       0.211      0.009       0.095       0.916      *
    YOL               -0.067       0.027      0.008      -0.120      -0.014      *
    BASAGEC90         -0.077       0.028      0.004      -0.131      -0.021      *

 AUT      WITH
    SLOPEAUT          -0.244       0.127      0.034      -0.477       0.020
    LNVAUT             0.410       0.192      0.026      -0.005       0.734

 SLOPEAUT WITH
    LNVAUT            -0.183       0.156      0.133      -0.470       0.139

 Means
    AUT                8.415       0.864      0.000       6.950      10.313      *
    SLOPEAUT          -0.200       0.077      0.004      -0.355      -0.051      *
    LNVAUT            -3.357       0.543      0.000      -4.585      -2.461      *

 Intercepts
    BASTTDC            5.654       1.584      0.000       2.962       9.159      *
    BASHKR             2.589       0.200      0.000       2.199       2.985      *
    BASSSSC            0.806       0.106      0.000       0.596       1.016      *

 Variances
    AUT                1.000       0.000      0.000       1.000       1.000
    PHYSCON            1.000       0.000      0.000       1.000       1.000
    SLOPEAUT           1.000       0.000      0.000       1.000       1.000
    LNVAUT             1.000       0.000      0.000       1.000       1.000

 Residual Variances
    BASTTDC            0.478       0.125      0.000       0.243       0.730      *
    BASHKR             0.837       0.106      0.000       0.574       0.981      *
    BASSSSC            0.559       0.206      0.000       0.051       0.860      *


STD Standardization
```

```
                                Posterior  One-Tailed         95% C.I.
                    Estimate       S.D.      P-Value   Lower 2.5%  Upper 2.5%  Significance

Within-Level Standardized Estimates Averaged Over Clusters

 SLOPEAUT | AUT ON
    TSBASE            -0.003       0.002      0.054      -0.007       0.001

 LNVAUT |
    AUT                0.146       0.013      0.000       0.121       0.171      *

Between Level

 PHYSCON  BY
    BASHKR             2.868       0.979      0.000       0.952       4.843      *
    BASSSSC            1.097       0.270      0.000       0.600       1.643      *

 BASTTDC    ON
    PHYSCON            1.635       0.418      0.000       0.864       2.503      *
    SLOPEAUT          -0.112       0.386      0.382      -0.867       0.649
    LNVAUT             1.316       0.543      0.007       0.267       2.386      *

 BASTTDC    ON
    AUT               -2.178       0.977      0.007      -4.275      -0.449      *
    SEX                1.693       0.721      0.009       0.311       3.126      *
    YOL               -0.222       0.090      0.008      -0.402      -0.045      *
    BASAGEC90         -0.254       0.094      0.004      -0.436      -0.069      *

 AUT      WITH
    SLOPEAUT          -0.116       0.066      0.034      -0.251       0.009
    LNVAUT             0.193       0.097      0.026      -0.002       0.377

 SLOPEAUT WITH
    LNVAUT            -0.183       0.156      0.133      -0.470       0.139

 Means
    AUT                4.014       0.051      0.000       3.915       4.117      *
    SLOPEAUT          -0.200       0.077      0.004      -0.355      -0.051      *
    LNVAUT            -3.357       0.543      0.000      -4.585      -2.461      *

 Intercepts
    BASTTDC           18.646       5.310      0.000       9.734      30.458      *
    BASHKR            18.466       0.673      0.000      17.143      19.768      *
    BASSSSC            1.329       0.152      0.000       1.028       1.628      *

 Variances
    AUT                0.227       0.047      0.000       0.151       0.334      *
    PHYSCON            1.000       0.000      0.000       1.000       1.000
    SLOPEAUT           1.000       0.000      0.000       1.000       1.000
    LNVAUT             1.000       0.000      0.000       1.000       1.000

 Residual Variances
    BASTTDC            5.170       1.455      0.000       2.663       8.331      *
    BASHKR            41.931       7.484      0.000      28.345      57.932      *
    BASSSSC            1.504       0.579      0.000       0.141       2.483      *


R-SQUARE

Within-Level R-Square Averaged Across Clusters

                                Posterior  One-Tailed         95% C.I.
    Variable        Estimate       S.D.      P-Value   Lower 2.5%  Upper 2.5%

    AUT                0.122       0.015      0.000       0.095       0.153

Between Level

                                Posterior  One-Tailed         95% C.I.
    Variable        Estimate       S.D.      P-Value   Lower 2.5%  Upper 2.5%

    BASTTDC            0.522       0.125      0.000       0.269       0.756
    BASHKR             0.163       0.106      0.000       0.019       0.426
    BASSSSC            0.441       0.206      0.000       0.140       0.949
```

```
TECHNICAL 8 OUTPUT


   TECHNICAL 8 OUTPUT FOR BAYES ESTIMATION

     CHAIN    BSEED
     1        0
     2        285380

                     POTENTIAL       PARAMETER WITH
     ITERATION    SCALE REDUCTION      HIGHEST PSR
     100              2.381               3
     200              2.424               3
     300              1.666               5
     400              1.306               20
     500              1.388               17
     600              1.707               17
     700              1.565               3
     800              1.610               20
     900              1.526               20
     1000             1.461               7
     1100             1.481               7
     1200             1.348               7
     1300             1.168               10
     1400             1.242               10
     1500             1.191               10
     1600             1.192               10
     1700             1.204               17
     1800             1.247               17
     1900             1.151               17
     2000             1.127               17
     2100             1.209               17
     2200             1.292               17
     2300             1.354               17
     2400             1.399               17
     2500             1.402               17
     2600             1.388               17
     2700             1.177               17
     2800             1.141               10
     2900             1.130               10
     3000             1.111               10
     3100             1.089               10
     3200             1.094               10
     3300             1.091               10
     3400             1.084               10
     3500             1.084               10
     3600             1.078               10
     3700             1.095               10
     3800             1.099               10
     3900             1.099               10
     4000             1.111               10
     4100             1.096               10
     4200             1.072               10
     4300             1.069               10
     4400             1.072               20
     4500             1.099               20
     4600             1.108               20
     4700             1.111               20
     4800             1.103               20
     4900             1.054               10
     5000             1.062               10
     5100             1.038               10
     5200             1.032               20
     5300             1.029               20
     5400             1.029               20
     5500             1.032               20
     5600             1.040               7
     5700             1.051               7
     5800             1.056               20
     5900             1.075               20
     6000             1.086               20
     6100             1.097               20
```

```
6200            1.106            20
6300            1.104            20
6400            1.109            20
6500            1.106            20
6600            1.098            20
6700            1.092            20
6800            1.093            20
6900            1.094            20
7000            1.093            20
7100            1.082            20
7200            1.077            20
7300            1.067            20
7400            1.050            20
7500            1.042            20
7600            1.041            20
7700            1.043            20
7800            1.048            20
7900            1.045            20
8000            1.041            20
8100            1.045            7
8200            1.047            7
8300            1.042            7
8400            1.036            7
8500            1.036            23
8600            1.040            23
8700            1.036            23
8800            1.031            23
8900            1.029            23
9000            1.031            23
9100            1.029            23
9200            1.028            17
9300            1.034            17
9400            1.031            17
9500            1.034            3
9600            1.035            3
9700            1.036            3
9800            1.041            3
9900            1.047            3
10000           1.050            3
10100           1.049            17
10200           1.049            17
10300           1.044            17
10400           1.038            17
10500           1.038            17
10600           1.033            17
10700           1.031            17
10800           1.028            17
10900           1.025            17
11000           1.020            17
11100           1.017            23
11200           1.015            23
11300           1.011            17
11400           1.011            17
11500           1.011            17
11600           1.008            17
11700           1.007            17
11800           1.008            17
11900           1.008            17
12000           1.008            17
12100           1.008            17
12200           1.007            17
12300           1.005            17
12400           1.004            13
12500           1.005            23
12600           1.006            7
12700           1.008            7
12800           1.010            7
12900           1.009            7
13000           1.008            7
13100           1.007            7
13200           1.004            13
13300           1.003            13
13400           1.006            20
13500           1.009            20
```

```
     13600            1.009              20
     13700            1.009              20
     13800            1.010              20
     13900            1.008              20
     14000            1.008              20
     14100            1.006              20
     14200            1.004              20
     14300            1.004              20
     14400            1.005              20
     14500            1.006              20
     14600            1.004              3
     14700            1.008              3
     14800            1.014              3
     14900            1.018              3
     15000            1.021              3
     15100            1.022              3
     15200            1.022              3
     15300            1.024              3
     15400            1.026              3
     15500            1.031              3
     15600            1.031              3
     15700            1.035              3
     15800            1.035              3
     15900            1.039              3
     16000            1.038              3
     16100            1.033              3
     16200            1.025              3
     16300            1.019              3
     16400            1.014              3
     16500            1.013              3
     16600            1.011              3
     16700            1.010              3
     16800            1.010              3
     16900            1.011              3
     17000            1.012              3
     17100            1.011              3
     17200            1.009              3
     17300            1.009              20
     17400            1.009              20
     17500            1.009              20
     17600            1.009              20
     17700            1.010              20
     17800            1.008              20
     17900            1.007              20
     18000            1.004              20
     18100            1.003              20
     18200            1.002              21


TECHNICAL 5 OUTPUT


DIAGRAM INFORMATION

  Mplus diagrams are currently not available for multilevel analysis.
  No diagram output was produced.


     Beginning Time:  15:33:17
        Ending Time:  15:36:23
       Elapsed Time:  00:03:06



MUTHEN & MUTHEN
3463 Stoner Ave.
Los Angeles, CA  90066

Tel: (310) 391-9971
Fax: (310) 391-8971
Web: www.StatModel.com
Support: Support@StatModel.com
```

Copyright (c) 1998-2020 Muthen & Muthen

Mplus Output Single Multilevel SEM – Environmental Mastery:

```
Mplus VERSION 8.5
MUTHEN & MUTHEN
10/15/2021   7:03 PM

INPUT INSTRUCTIONS

  TITLE:

  DATA: FILE = mpltry6.dat;

  VARIABLE:
   names =  pno wave sex yol verst nopart
         basagec90 agec90 tims tsbase
         ttd ttdcens basettd basttdc
         aut pil ema sac dep
         pa na swls adlinv hyp
         visus sfenerg sfsofunc sfpain sfgen sfchang
         anges anget akzet mortinv mortperc
         basadlinv basvisus bashkr bassssc
         bassfen bassfso bassfpain bassfgen bassfcha
         hkr sl sssco;
  usevariables = ema tsbase basttdc bashkr bassssc basagec90 sex yol;
  cluster = PNO;
  missing = .;
  within = tsbase;
  between = basttdc bashkr bassssc basagec90 sex yol;

 DEFINE:
  center sex yol (grandmean);

 ANALYSIS:
  type = twolevel random;
  estimator = BAYES;
  chains = 2;
  biterations=1000000;
  BCONVERGENCE = 0.00125;

 MODEL:

  %WITHIN%

  slopeema | ema ON tsbase;
  lnvema | ema;

  %BETWEEN%

  physcon BY bashkr@1;
             bassssc*0.29379;

  basttdc ON ema*-0.41929
             sex*2.80529
             yol*-0.45843
             basagec90*-0.26795
             physcon*0.44378
             slopeema*5.74904
             lnvema*2.97300;

  ema WITH slopeema*-0.00401;
  ema WITH lnvema*0.14651;
  slopeema WITH lnvema*-0.00271;

  [ basttdc*13.85891 ];
  [ bashkr*18.96634 ];
  [ bassssc*1.25843 ];
  [ ema*4.17714 ] (m3);
  [ slopeema*-0.01565 ] (ms3);
  [ lnvema*-2.05291 ] (mv3);
```

```
  basttdc*7.63556;
  bashkr*28.79375;
  bassssc*1.42364;
  ema*0.31594;
  physcon*17.30515;
  slopeema*0.00072;
  lnvema*0.22910;


 MODEL PRIORS:
  m3 ~ N(4.05,0.078);
  ms3 ~ N(-0.052,0.0001);

 OUTPUT:
  standardized tech5 tech8 tech16;




*** WARNING in VARIABLE command
  Note that only the first 8 characters of variable names are used in the output.
  Shorten variable names to avoid any confusion.
*** WARNING in MODEL command
  In the MODEL command, the following variable is an x-variable on the BETWEEN
  level and a y-variable on the WITHIN level.  This variable will be treated
  as a y-variable on both levels:  EMA
*** WARNING
  One or more individual-level variables have no variation within a
  cluster for the following clusters.

     Variable   Cluster IDs with no within-cluster variation

      EMA         10969 16142 16798 11384

   3 WARNING(S) FOUND IN THE INPUT INSTRUCTIONS




SUMMARY OF ANALYSIS

Number of groups                                                 1
Number of observations                                         682

Number of dependent variables                                    4
Number of independent variables                                  4
Number of continuous latent variables                            3

Observed dependent variables

  Continuous
   BASTTDC     BASHKR      BASSSSC     EMA

Observed independent variables
   TSBASE      BASAGEC9    SEX         YOL

Continuous latent variables
   PHYSCON     SLOPEEMA    LNVEMA

Variables with special functions

  Cluster variable      PNO

  Within variables
   TSBASE

  Between variables
   BASTTDC     BASHKR      BASSSSC     BASAGEC9    SEX         YOL

  Centering (GRANDMEAN)
   SEX         YOL


Estimator                                                    BAYES
```

```
Specifications for Bayesian Estimation
  Point estimate                                            MEDIAN
  Number of Markov chain Monte Carlo (MCMC) chains               2
  Random seed for the first chain                                0
  Starting value information                           UNPERTURBED
  Algorithm used for Markov chain Monte Carlo           GIBBS(PX1)
  Convergence criterion                                  0.125D-02
  Maximum number of iterations                             1000000
  K-th iteration used for thinning                               1

Input data file(s)
  mpltry6.dat
Input data format  FREE


SUMMARY OF DATA

     Number of clusters                       124

       Size (s)    Cluster ID with Size s

          1        10026 10130 10144 10313 10473 10558 10567 10637 10663
                   10893 10901 10907 10911 10939 10969 11157 11240 11294
                   11297 11741 15018 15177 15241 15319 15564 16142 16423
                   16610 16669 16798 17542 18507 18650
          2        15239 11258 10181 15510 10615 11341 16303 11371 16510
                   11384 11485 11028 16862 10211 17780 18004 18252 15175
                   10228
          3        11573 11295 17130 11100 17745 10340 10460 10461 10210
                   11501
          4        10904 11457 10434 11493 10811 17593
          5        15424 10666 12411 11343 11005
          6        10159 15092 15121 15426 11593 11163 10139 16220 19067
          7        10354 11230 10894 10724 10470
          8        10252 10528 11038 10033 11336 11253
          9        15623 11055
         10        12037 10167 17197 15009 19500
         11        11233 10444 15714 10533
         12        11079 10577 15378 12714 10986 10428
         13        11420 11002
         14        11450
         15        10108 10906 10551 15231 10902
         16        15141 10940 16103 15472 11528 11378



COVARIANCE COVERAGE OF DATA

Minimum covariance coverage value   0.100

     Number of missing data patterns            8


     PROPORTION OF DATA PRESENT


           Covariance Coverage
              BASTTDC       BASHKR        BASSSSC       EMA           TSBASE
              ________      ________      ________      ________      ________
 BASTTDC        0.944
 BASHKR         0.912         0.968
 BASSSSC        0.935         0.968         0.991
 EMA            0.921         0.944         0.966         0.974
 TSBASE         0.944         0.968         0.991         0.974         1.000
 BASAGEC9       0.944         0.968         0.991         0.974         1.000
 SEX            0.944         0.968         0.991         0.974         1.000
 YOL            0.944         0.968         0.991         0.974         1.000


           Covariance Coverage
              BASAGEC9      SEX           YOL
              ________      ________      ________
 BASAGEC9       1.000
 SEX            1.000         1.000
```

```
 YOL            1.000         1.000         1.000


UNIVARIATE SAMPLE STATISTICS


     UNIVARIATE HIGHER-ORDER MOMENT DESCRIPTIVE STATISTICS

         Variable/         Mean/     Skewness/   Minimum/ % with                Percentiles
        Sample Size      Variance    Kurtosis    Maximum  Min/Max      20%/60%    40%/80%    Median

     BASTTDC               5.519       0.456       0.250    0.83%       2.250      4.250      5.083
             120.000      10.267      -0.614      12.667    1.67%       6.083      8.250
     BASHKR               18.519       1.282       5.330    0.89%      12.330     16.500     17.750
             112.000      48.308       2.780      49.000    0.89%      18.670     23.330
     BASSSSC               1.333       0.640       0.000   53.33%       0.000      0.000      0.000
             120.000       2.606      -1.294       4.000   17.50%       1.000      3.000
     EMA                   3.936      -0.567       1.222    0.15%       3.444      3.778      4.000
             664.000       0.400       0.104       5.000    2.11%       4.111      4.556
     TSBASE               36.711       0.258       0.000    3.08%      13.000     27.000     36.000
             682.000     594.554      -0.917      91.000    0.59%      41.000     60.000
     BASAGEC90             0.106       0.407      -3.917    0.81%      -2.833     -1.250     -0.792
             124.000       8.313      -1.090       6.417    0.81%       0.500      3.083
     SEX                   0.000      -1.426      -0.790   20.97%      -0.790      0.210      0.210
             124.000       0.166       0.035       0.210   79.03%       0.210      0.210
     YOL                   0.000       0.868      -3.387   20.97%      -3.387     -1.387     -1.387
             124.000       8.737      -0.315       5.613   17.74%       0.613      0.613


THE MODEL ESTIMATION TERMINATED NORMALLY

     USE THE FBITERATIONS OPTION TO INCREASE THE NUMBER OF ITERATIONS BY A FACTOR
     OF AT LEAST TWO TO CHECK CONVERGENCE AND THAT THE PSR VALUE DOES NOT INCREASE.



MODEL FIT INFORMATION

Number of Free Parameters                              24

Information Criteria

          Deviance (DIC)                         2665.294
          Estimated Number of Parameters (pD)     182.895



MODEL RESULTS

                                Posterior  One-Tailed         95% C.I.
                    Estimate       S.D.      P-Value   Lower 2.5%  Upper 2.5%  Significance

Within Level

Between Level

 PHYSCON  BY
    BASHKR             1.000       0.000      0.000       1.000       1.000
    BASSSSC            0.399       0.399      0.000       0.136       1.408      *

 BASTTDC    ON
    PHYSCON            0.563       0.446      0.000       0.223       1.796      *
    SLOPEEMA          10.293      16.820      0.268     -22.607      43.323
    LNVEMA             2.368       1.650      0.044      -0.489       5.748

 BASTTDC    ON
    EMA                0.736       0.828      0.174      -0.868       2.413
    SEX                1.644       0.733      0.010       0.237       3.128      *
    YOL               -0.290       0.092      0.001      -0.470      -0.109      *
    BASAGEC90         -0.224       0.095      0.010      -0.411      -0.037      *

 EMA      WITH
    SLOPEEMA          -0.003       0.002      0.040      -0.007       0.000
```

```
    LNVEMA             0.010       0.057      0.428      -0.099       0.126

 SLOPEEMA WITH
    LNVEMA            -0.001       0.002      0.297      -0.006       0.003

 Means
    EMA                4.128       0.058      0.000       4.013       4.241      *
    SLOPEEMA          -0.011       0.003      0.001      -0.017      -0.004      *
    LNVEMA            -1.968       0.109      0.000      -2.182      -1.758      *

 Intercepts
    BASTTDC            7.187       4.759      0.040      -0.936      17.386
    BASHKR            18.478       0.673      0.000      17.151      19.799      *
    BASSSSC            1.332       0.152      0.000       1.031       1.628      *

 Variances
    EMA                0.278       0.056      0.000       0.187       0.407      *
    PHYSCON            7.177       6.324      0.000       0.767      24.377      *
    SLOPEEMA           0.001       0.000      0.000       0.000       0.001      *
    LNVEMA             0.196       0.102      0.000       0.054       0.445      *

 Residual Variances
    BASTTDC            5.771       1.527      0.000       3.021       8.959      *
    BASHKR            42.068       7.901      0.000      26.858      58.323      *
    BASSSSC            1.556       0.627      0.000       0.187       2.587      *


STANDARDIZED MODEL RESULTS


STDYX Standardization

                                Posterior  One-Tailed         95% C.I.
                    Estimate       S.D.      P-Value   Lower 2.5%  Upper 2.5%  Significance

Within-Level Standardized Estimates Averaged Over Clusters

 SLOPEEMA | EMA ON
    TSBASE            -0.159       0.023      0.000      -0.203      -0.112      *

 LNVEMA |
    EMA                0.843       0.013      0.000       0.816       0.868      *

Between Level

 PHYSCON  BY
    BASHKR             0.379       0.142      0.000       0.124       0.671      *
    BASSSSC            0.649       0.169      0.000       0.328       0.965      *

 BASTTDC    ON
    PHYSCON            0.459       0.129      0.000       0.215       0.718      *
    SLOPEEMA           0.077       0.125      0.268      -0.166       0.323
    LNVEMA             0.324       0.172      0.044      -0.056       0.617

 BASTTDC    ON
    EMA                0.118       0.130      0.174      -0.141       0.372
    SEX                0.146       0.062      0.010       0.021       0.266      *
    YOL               -0.187       0.057      0.001      -0.295      -0.070      *
    BASAGEC90         -0.130       0.055      0.010      -0.236      -0.021      *

 EMA      WITH
    SLOPEEMA          -0.242       0.131      0.040      -0.479       0.030
    LNVEMA             0.044       0.243      0.428      -0.425       0.521

 SLOPEEMA WITH
    LNVEMA            -0.097       0.176      0.297      -0.432       0.253

 Means
    EMA                7.827       0.798      0.000       6.456       9.573      *
    SLOPEEMA          -0.436       0.136      0.001      -0.704      -0.174      *
    LNVEMA            -4.465       1.490      0.000      -8.024      -3.010      *

 Intercepts
    BASTTDC            2.187       1.434      0.040      -0.283       5.252
```

```
    BASHKR             2.611       0.201      0.000       2.222       3.010      *
    BASSSSC            0.809       0.106      0.000       0.599       1.017      *

 Variances
    EMA                1.000       0.000      0.000       1.000       1.000
    PHYSCON            1.000       0.000      0.000       1.000       1.000
    SLOPEEMA           1.000       0.000      0.000       1.000       1.000
    LNVEMA             1.000       0.000      0.000       1.000       1.000

 Residual Variances
    BASTTDC            0.535       0.130      0.000       0.276       0.777      *
    BASHKR             0.857       0.117      0.000       0.549       0.985      *
    BASSSSC            0.579       0.222      0.000       0.069       0.893      *


STDY Standardization

                                Posterior  One-Tailed         95% C.I.
                    Estimate       S.D.      P-Value   Lower 2.5%  Upper 2.5%  Significance

Within-Level Standardized Estimates Averaged Over Clusters

 SLOPEEMA | EMA ON
    TSBASE            -0.019       0.007      0.005      -0.033      -0.006      *

 LNVEMA |
    EMA                0.843       0.013      0.000       0.816       0.868      *

Between Level

 PHYSCON  BY
    BASHKR             0.379       0.142      0.000       0.124       0.671      *
    BASSSSC            0.649       0.169      0.000       0.328       0.965      *

 BASTTDC    ON
    PHYSCON            0.459       0.129      0.000       0.215       0.718      *
    SLOPEEMA           0.077       0.125      0.268      -0.166       0.323
    LNVEMA             0.324       0.172      0.044      -0.056       0.617

 BASTTDC    ON
    EMA                0.118       0.130      0.174      -0.141       0.372
    SEX                0.500       0.214      0.010       0.072       0.913      *
    YOL               -0.088       0.027      0.001      -0.139      -0.033      *
    BASAGEC90         -0.068       0.029      0.010      -0.123      -0.011      *

 EMA      WITH
    SLOPEEMA          -0.242       0.131      0.040      -0.479       0.030
    LNVEMA             0.044       0.243      0.428      -0.425       0.521

 SLOPEEMA WITH
    LNVEMA            -0.097       0.176      0.297      -0.432       0.253

 Means
    EMA                7.827       0.798      0.000       6.456       9.573      *
    SLOPEEMA          -0.436       0.136      0.001      -0.704      -0.174      *
    LNVEMA            -4.465       1.490      0.000      -8.024      -3.010      *

 Intercepts
    BASTTDC            2.187       1.434      0.040      -0.283       5.252
    BASHKR             2.611       0.201      0.000       2.222       3.010      *
    BASSSSC            0.809       0.106      0.000       0.599       1.017      *

 Variances
    EMA                1.000       0.000      0.000       1.000       1.000
    PHYSCON            1.000       0.000      0.000       1.000       1.000
    SLOPEEMA           1.000       0.000      0.000       1.000       1.000
    LNVEMA             1.000       0.000      0.000       1.000       1.000

 Residual Variances
    BASTTDC            0.535       0.130      0.000       0.276       0.777      *
    BASHKR             0.857       0.117      0.000       0.549       0.985      *
    BASSSSC            0.579       0.222      0.000       0.069       0.893      *
```

```
STD Standardization

                                Posterior  One-Tailed         95% C.I.
                    Estimate       S.D.      P-Value   Lower 2.5%  Upper 2.5%  Significance

Within-Level Standardized Estimates Averaged Over Clusters

 SLOPEEMA | EMA ON
    TSBASE            -0.009       0.003      0.000      -0.014      -0.004      *

 LNVEMA |
    EMA                0.153       0.015      0.000       0.133       0.194      *

Between Level

 PHYSCON  BY
    BASHKR             2.679       1.055      0.000       0.876       4.937      *
    BASSSSC            1.073       0.294      0.000       0.530       1.641      *

 BASTTDC    ON
    PHYSCON            1.510       0.451      0.000       0.699       2.458      *
    SLOPEEMA           0.253       0.416      0.268      -0.556       1.080
    LNVEMA             1.065       0.576      0.044      -0.183       2.078

 BASTTDC    ON
    EMA                0.736       0.828      0.174      -0.868       2.413
    SEX                1.644       0.733      0.010       0.237       3.128      *
    YOL               -0.290       0.092      0.001      -0.470      -0.109      *
    BASAGEC90         -0.224       0.095      0.010      -0.411      -0.037      *

 EMA      WITH
    SLOPEEMA          -0.126       0.075      0.040      -0.280       0.015
    LNVEMA             0.023       0.131      0.428      -0.221       0.292

 SLOPEEMA WITH
    LNVEMA            -0.097       0.176      0.297      -0.432       0.253

 Means
    EMA                4.128       0.058      0.000       4.013       4.241      *
    SLOPEEMA          -0.436       0.136      0.001      -0.704      -0.174      *
    LNVEMA            -4.465       1.490      0.000      -8.024      -3.010      *

 Intercepts
    BASTTDC            7.187       4.759      0.040      -0.936      17.386
    BASHKR            18.478       0.673      0.000      17.151      19.799      *
    BASSSSC            1.332       0.152      0.000       1.031       1.628      *

 Variances
    EMA                0.278       0.056      0.000       0.187       0.407      *
    PHYSCON            1.000       0.000      0.000       1.000       1.000
    SLOPEEMA           1.000       0.000      0.000       1.000       1.000
    LNVEMA             1.000       0.000      0.000       1.000       1.000

 Residual Variances
    BASTTDC            5.771       1.527      0.000       3.021       8.959      *
    BASHKR            42.068       7.901      0.000      26.858      58.323      *
    BASSSSC            1.556       0.627      0.000       0.187       2.587      *


R-SQUARE

Within-Level R-Square Averaged Across Clusters

                                Posterior  One-Tailed         95% C.I.
    Variable        Estimate       S.D.      P-Value   Lower 2.5%  Upper 2.5%

    EMA                0.157       0.013      0.000       0.132       0.184

Between Level

                                Posterior  One-Tailed         95% C.I.
    Variable        Estimate       S.D.      P-Value   Lower 2.5%  Upper 2.5%

    BASTTDC            0.465       0.130      0.000       0.223       0.724
```

BASHKR	0.143	0.117	0.000	0.015	0.450
BASSSSC	0.421	0.222	0.000	0.107	0.931

TECHNICAL 8 OUTPUT

TECHNICAL 8 OUTPUT FOR BAYES ESTIMATION

CHAIN	BSEED
1	0
2	285380

ITERATION	POTENTIAL SCALE REDUCTION	PARAMETER WITH HIGHEST PSR
100	3.668	17
200	1.418	20
300	2.224	20
400	1.764	20
500	1.257	23
600	1.132	20
700	1.615	20
800	1.823	20
900	2.270	7
1000	2.335	7
1100	2.233	7
1200	1.968	20
1300	1.905	20
1400	1.838	20
1500	1.849	20
1600	1.849	20
1700	1.959	20
1800	2.035	20
1900	2.090	20
2000	1.921	20
2100	1.841	20
2200	1.708	20
2300	1.500	7
2400	1.430	7
2500	1.249	7
2600	1.255	7
2700	1.147	7
2800	1.090	7
2900	1.067	7
3000	1.056	21
3100	1.037	21
3200	1.034	5
3300	1.048	17
3400	1.064	5
3500	1.080	5
3600	1.085	3
3700	1.082	3
3800	1.058	3
3900	1.045	23
4000	1.031	23
4100	1.022	23
4200	1.024	23
4300	1.016	23
4400	1.018	7
4500	1.027	7
4600	1.036	7
4700	1.042	7
4800	1.038	7
4900	1.046	7
5000	1.050	7
5100	1.043	7
5200	1.047	20
5300	1.053	20
5400	1.064	20
5500	1.057	20
5600	1.041	20
5700	1.049	20

5800	1.062	20
5900	1.067	20
6000	1.067	7
6100	1.068	7
6200	1.065	7
6300	1.063	7
6400	1.050	7
6500	1.038	7
6600	1.031	7
6700	1.031	5
6800	1.032	5
6900	1.032	5
7000	1.028	5
7100	1.023	21
7200	1.021	21
7300	1.017	12
7400	1.019	10
7500	1.023	10
7600	1.024	10
7700	1.027	10
7800	1.030	10
7900	1.033	10
8000	1.032	10
8100	1.031	10
8200	1.034	10
8300	1.037	10
8400	1.035	10
8500	1.035	10
8600	1.035	10
8700	1.036	10
8800	1.039	10
8900	1.038	10
9000	1.039	10
9100	1.047	10
9200	1.053	3
9300	1.058	3
9400	1.059	3
9500	1.061	3
9600	1.064	3
9700	1.062	3
9800	1.060	3
9900	1.062	3
10000	1.063	3
10100	1.057	3
10200	1.055	3
10300	1.053	3
10400	1.049	3
10500	1.048	3
10600	1.049	3
10700	1.048	3
10800	1.049	3
10900	1.050	3
11000	1.049	3
11100	1.047	3
11200	1.049	3
11300	1.049	3
11400	1.049	3
11500	1.048	3
11600	1.047	3
11700	1.048	7
11800	1.052	7
11900	1.055	7
12000	1.063	7
12100	1.066	7
12200	1.072	7
12300	1.076	7
12400	1.081	7
12500	1.077	7
12600	1.074	7
12700	1.078	7
12800	1.085	7
12900	1.089	7
13000	1.098	7
13100	1.104	20

```
13200            1.104            20
13300            1.097            20
13400            1.099            20
13500            1.099            20
13600            1.096            20
13700            1.093            20
13800            1.090            20
13900            1.095            20
14000            1.101            20
14100            1.096            20
14200            1.093            20
14300            1.094            20
14400            1.095            7
14500            1.102            7
14600            1.107            7
14700            1.116            7
14800            1.121            7
14900            1.127            7
15000            1.133            7
15100            1.129            7
15200            1.122            7
15300            1.119            7
15400            1.115            7
15500            1.107            7
15600            1.105            7
15700            1.102            7
15800            1.105            7
15900            1.106            7
16000            1.110            7
16100            1.113            7
16200            1.114            7
16300            1.111            7
16400            1.105            7
16500            1.103            7
16600            1.101            7
16700            1.091            7
16800            1.087            7
16900            1.081            7
17000            1.071            7
17100            1.064            7
17200            1.059            7
17300            1.060            7
17400            1.056            7
17500            1.052            20
17600            1.054            20
17700            1.052            20
17800            1.047            7
17900            1.042            7
18000            1.037            7
18100            1.036            7
18200            1.036            7
18300            1.036            7
18400            1.041            7
18500            1.046            7
18600            1.053            7
18700            1.057            7
18800            1.058            7
18900            1.055            7
19000            1.050            7
19100            1.047            7
19200            1.045            7
19300            1.046            7
19400            1.043            7
19500            1.041            7
19600            1.042            7
19700            1.042            7
19800            1.041            7
19900            1.040            7
20000            1.037            7
20100            1.036            7
20200            1.036            7
20300            1.036            7
20400            1.037            7
20500            1.038            7
```

20600	1.040	7
20700	1.043	7
20800	1.046	7
20900	1.049	7
21000	1.046	7
21100	1.041	7
21200	1.038	7
21300	1.034	7
21400	1.030	7
21500	1.028	7
21600	1.031	17
21700	1.033	17
21800	1.033	17
21900	1.031	17
22000	1.030	17
22100	1.030	17
22200	1.029	17
22300	1.031	17
22400	1.032	17
22500	1.029	17
22600	1.027	17
22700	1.027	17
22800	1.027	17
22900	1.028	17
23000	1.029	17
23100	1.029	17
23200	1.028	17
23300	1.026	17
23400	1.023	17
23500	1.021	17
23600	1.021	17
23700	1.021	17
23800	1.018	17
23900	1.015	17
24000	1.014	17
24100	1.015	17
24200	1.017	17
24300	1.018	17
24400	1.017	17
24500	1.016	17
24600	1.018	17
24700	1.019	17
24800	1.019	17
24900	1.020	17
25000	1.018	17
25100	1.018	17
25200	1.018	17
25300	1.017	17
25400	1.014	17
25500	1.013	17
25600	1.013	17
25700	1.012	17
25800	1.012	17
25900	1.013	17
26000	1.012	17
26100	1.013	17
26200	1.013	17
26300	1.014	17
26400	1.014	17
26500	1.015	17
26600	1.015	17
26700	1.017	17
26800	1.020	17
26900	1.021	17
27000	1.020	17
27100	1.019	17
27200	1.018	17
27300	1.020	17
27400	1.021	17
27500	1.021	17
27600	1.021	17
27700	1.020	17
27800	1.019	17
27900	1.018	17

28000	1.016	17
28100	1.016	17
28200	1.016	17
28300	1.016	17
28400	1.016	17
28500	1.016	17
28600	1.015	17
28700	1.015	17
28800	1.015	17
28900	1.015	17
29000	1.016	17
29100	1.017	17
29200	1.017	17
29300	1.017	17
29400	1.016	17
29500	1.016	17
29600	1.016	17
29700	1.015	20
29800	1.015	17
29900	1.014	17
30000	1.013	17
30100	1.013	17
30200	1.012	17
30300	1.012	17
30400	1.011	17
30500	1.011	17
30600	1.011	17
30700	1.011	17
30800	1.011	17
30900	1.012	17
31000	1.012	17
31100	1.013	17
31200	1.012	5
31300	1.010	5
31400	1.010	5
31500	1.010	5
31600	1.011	5
31700	1.011	5
31800	1.011	5
31900	1.011	5
32000	1.010	5
32100	1.010	5
32200	1.011	5
32300	1.011	5
32400	1.011	5
32500	1.011	5
32600	1.012	5
32700	1.013	5
32800	1.014	5
32900	1.013	5
33000	1.013	17
33100	1.013	17
33200	1.013	17
33300	1.014	17
33400	1.015	17
33500	1.014	17
33600	1.014	17
33700	1.014	17
33800	1.014	17
33900	1.014	17
34000	1.013	17
34100	1.013	17
34200	1.014	17
34300	1.013	17
34400	1.013	17
34500	1.012	17
34600	1.012	17
34700	1.013	17
34800	1.014	17
34900	1.013	17
35000	1.011	17
35100	1.009	17
35200	1.008	17
35300	1.009	17

35400	1.008	17
35500	1.008	17
35600	1.008	17
35700	1.008	17
35800	1.009	17
35900	1.009	17
36000	1.008	17
36100	1.008	17
36200	1.008	17
36300	1.007	3
36400	1.007	3
36500	1.007	3
36600	1.007	17
36700	1.007	17
36800	1.007	17
36900	1.008	17
37000	1.008	17
37100	1.008	17
37200	1.008	17
37300	1.008	17
37400	1.007	3
37500	1.006	3
37600	1.006	3
37700	1.006	3
37800	1.006	3
37900	1.006	3
38000	1.005	3
38100	1.006	5
38200	1.006	5
38300	1.006	3
38400	1.008	3
38500	1.011	3
38600	1.013	3
38700	1.013	3
38800	1.014	3
38900	1.015	3
39000	1.015	3
39100	1.016	3
39200	1.016	3
39300	1.016	3
39400	1.016	3
39500	1.016	3
39600	1.017	3
39700	1.017	3
39800	1.015	3
39900	1.013	3
40000	1.012	3
40100	1.012	3
40200	1.012	3
40300	1.011	3
40400	1.011	3
40500	1.011	3
40600	1.010	3
40700	1.011	3
40800	1.010	3
40900	1.010	3
41000	1.010	3
41100	1.009	3
41200	1.009	7
41300	1.009	7
41400	1.009	3
41500	1.009	3
41600	1.009	3
41700	1.009	3
41800	1.010	3
41900	1.011	3
42000	1.011	3
42100	1.011	3
42200	1.011	3
42300	1.011	3
42400	1.011	3
42500	1.011	3
42600	1.010	3
42700	1.010	3

```
42800           1.010           3
42900           1.009           3
43000           1.009           3
43100           1.009           3
43200           1.008           3
43300           1.008           3
43400           1.009           3
43500           1.009           3
43600           1.009           3
43700           1.009           3
43800           1.009           3
43900           1.009           3
44000           1.010           3
44100           1.009           3
44200           1.009           3
44300           1.009           3
44400           1.010           3
44500           1.009           3
44600           1.009           3
44700           1.009           3
44800           1.009           3
44900           1.010           3
45000           1.010           3
45100           1.010           3
45200           1.009           3
45300           1.009           3
45400           1.008           3
45500           1.007           23
45600           1.008           23
45700           1.008           23
45800           1.008           23
45900           1.008           23
46000           1.008           23
46100           1.008           23
46200           1.008           23
46300           1.008           23
46400           1.008           23
46500           1.008           23
46600           1.008           23
46700           1.008           23
46800           1.008           23
46900           1.008           23
47000           1.008           23
47100           1.009           23
47200           1.008           23
47300           1.008           23
47400           1.008           23
47500           1.008           7
47600           1.008           7
47700           1.009           7
47800           1.008           7
47900           1.008           7
48000           1.007           7
48100           1.007           7
48200           1.007           23
48300           1.007           23
48400           1.006           23
48500           1.007           23
48600           1.007           23
48700           1.007           23
48800           1.007           23
48900           1.007           23
49000           1.007           23
49100           1.007           23
49200           1.007           7
49300           1.008           7
49400           1.009           7
49500           1.009           7
49600           1.009           7
49700           1.010           7
49800           1.010           7
49900           1.010           7
50000           1.011           7
50100           1.011           7
```

```
     50200            1.010              7
     50300            1.010              7
     50400            1.009              7
     50500            1.009              7
     50600            1.008              7
     50700            1.008              7
     50800            1.007              7
     50900            1.007              7
     51000            1.007              7
     51100            1.007              7
     51200            1.007              7
     51300            1.007              7
     51400            1.008              7
     51500            1.008              7
     51600            1.008              7
     51700            1.007              7
     51800            1.006              7
     51900            1.006              7
     52000            1.005              7
     52100            1.005              7
     52200            1.005              7
     52300            1.005              7
     52400            1.005              7
     52500            1.004              7
     52600            1.004              7
     52700            1.003              7
     52800            1.003              7
     52900            1.003              7
     53000            1.002              7
     53100            1.002              23
     53200            1.003              23
     53300            1.003              23
     53400            1.002              23
     53500            1.003              7
     53600            1.002              23
     53700            1.003              23
     53800            1.003              23
     53900            1.003              23
     54000            1.002              7
```

TECHNICAL 5 OUTPUT

DIAGRAM INFORMATION

  Mplus diagrams are currently not available for multilevel analysis.
  No diagram output was produced.

```
     Beginning Time:  19:03:06
        Ending Time:  19:12:16
       Elapsed Time:  00:09:10
```

MUTHEN & MUTHEN
3463 Stoner Ave.
Los Angeles, CA 90066

Tel: (310) 391-9971
Fax: (310) 391-8971
Web: www.StatModel.com
Support: Support@StatModel.com

Copyright (c) 1998-2020 Muthen & Muthen

Mplus Output Single Multilevel SEM – Purpose in Life:

```
Mplus VERSION 8.5
MUTHEN & MUTHEN
10/15/2021   7:52 PM

INPUT INSTRUCTIONS

  TITLE:

  DATA: FILE = mpltry6.dat;

  VARIABLE:
   names =  pno wave sex yol verst nopart
         basagec90 agec90 tims tsbase
         ttd ttdcens basettd basttdc
         aut pil ema sac dep
         pa na swls adlinv hyp
         visus sfenerg sfsofunc sfpain sfgen sfchang
         anges anget akzet mortinv mortperc
         basadlinv basvisus bashkr bassssc
         bassfen bassfso bassfpain bassfgen bassfcha
         hkr sl sssco;
  usevariables = pil tsbase basttdc bashkr bassssc basagec90 sex yol;
  cluster = PNO;
  missing = .;
  within = tsbase;
  between = basttdc bashkr bassssc basagec90 sex yol;

 DEFINE:
  center sex yol (grandmean);

 ANALYSIS:
  type = twolevel random;
  estimator = BAYES;
  chains = 2;
  biterations=1000000;
  BCONVERGENCE = 0.00125;

 MODEL:

  %WITHIN%

  slopepil | pil ON tsbase;
  lnvpil | pil;

  %BETWEEN%
  physcon by bashkr@1
             bassssc*0.4;

  basttdc ON pil
             sex
             yol
             physcon*0.7
             basagec90
             slopepil
             lnvpil;

  [ pil*3.08197 ] (m2);
  [ slopepil*-0.00398 ] (ms2);
  [ lnvpil*-1.93665 ] (mv2);

  basttdc*6.66980;
  pil*0.27574;
  slopepil*0.00045;
  lnvpil*0.38161;

  pil with slopepil lnvpil;
  slopepil with lnvpil;

 MODEL PRIORS:
```

```
  m2 ~ N(3.16,0.109);
  ms2 ~ N(-0.075,0.0001);

 OUTPUT:
  standardized tech5 tech8 tech16;




*** WARNING in VARIABLE command
  Note that only the first 8 characters of variable names are used in the output.
  Shorten variable names to avoid any confusion.
*** WARNING in MODEL command
  In the MODEL command, the following variable is an x-variable on the BETWEEN
  level and a y-variable on the WITHIN level.  This variable will be treated
  as a y-variable on both levels:  PIL
*** WARNING
  One or more individual-level variables have no variation within a
  cluster for the following clusters.

     Variable   Cluster IDs with no within-cluster variation

      PIL         10130 11297 16142 16798 16510 10211

   3 WARNING(S) FOUND IN THE INPUT INSTRUCTIONS




SUMMARY OF ANALYSIS

Number of groups                                                 1
Number of observations                                         682

Number of dependent variables                                    4
Number of independent variables                                  4
Number of continuous latent variables                            3

Observed dependent variables

  Continuous
   BASTTDC     BASHKR      BASSSSC     PIL

Observed independent variables
   TSBASE      BASAGEC9    SEX         YOL

Continuous latent variables
   PHYSCON     SLOPEPIL    LNVPIL

Variables with special functions

  Cluster variable      PNO

  Within variables
   TSBASE

  Between variables
   BASTTDC     BASHKR      BASSSSC     BASAGEC9    SEX         YOL

  Centering (GRANDMEAN)
   SEX         YOL


Estimator                                                    BAYES
Specifications for Bayesian Estimation
  Point estimate                                            MEDIAN
  Number of Markov chain Monte Carlo (MCMC) chains               2
  Random seed for the first chain                                0
  Starting value information                           UNPERTURBED
  Algorithm used for Markov chain Monte Carlo           GIBBS(PX1)
  Convergence criterion                                  0.125D-02
  Maximum number of iterations                             1000000
  K-th iteration used for thinning                               1
```

```
Input data file(s)
  mpltry6.dat
Input data format  FREE


SUMMARY OF DATA

     Number of clusters                        124

       Size (s)    Cluster ID with Size s

          1        10026 10130 10144 10313 10473 10558 10567 10637 10663
                   10893 10901 10907 10911 10939 10969 11157 11240 11294
                   11297 11741 15018 15177 15241 15319 15564 16142 16423
                   16610 16669 16798 17542 18507 18650
          2        15239 11258 10181 15510 10615 11341 16303 11371 16510
                   11384 11485 11028 16862 10211 17780 18004 18252 15175
                   10228
          3        11573 11295 17130 11100 17745 10340 10460 10461 10210
                   11501
          4        10904 11457 10434 11493 10811 17593
          5        15424 10666 12411 11343 11005
          6        10159 15092 15121 15426 11593 11163 10139 16220 19067
          7        10354 11230 10894 10724 10470
          8        10252 10528 11038 10033 11336 11253
          9        15623 11055
         10        12037 10167 17197 15009 19500
         11        11233 10444 15714 10533
         12        11079 10577 15378 12714 10986 10428
         13        11420 11002
         14        11450
         15        10108 10906 10551 15231 10902
         16        15141 10940 16103 15472 11528 11378



COVARIANCE COVERAGE OF DATA

Minimum covariance coverage value   0.100

     Number of missing data patterns             8


     PROPORTION OF DATA PRESENT


           Covariance Coverage
              BASTTDC       BASHKR        BASSSSC       PIL           TSBASE
              ________      ________      ________      ________      ________
 BASTTDC        0.944
 BASHKR         0.912         0.968
 BASSSSC        0.935         0.968         0.991
 PIL            0.909         0.931         0.952         0.957
 TSBASE         0.944         0.968         0.991         0.957         1.000
 BASAGEC9       0.944         0.968         0.991         0.957         1.000
 SEX            0.944         0.968         0.991         0.957         1.000
 YOL            0.944         0.968         0.991         0.957         1.000


           Covariance Coverage
              BASAGEC9      SEX           YOL
              ________      ________      ________
 BASAGEC9       1.000
 SEX            1.000         1.000
 YOL            1.000         1.000         1.000



UNIVARIATE SAMPLE STATISTICS


     UNIVARIATE HIGHER-ORDER MOMENT DESCRIPTIVE STATISTICS
```

```
          Variable/         Mean/     Skewness/   Minimum/ % with                Percentiles
         Sample Size      Variance    Kurtosis    Maximum  Min/Max      20%/60%    40%/80%    Median

     BASTTDC               5.519       0.456       0.250    0.83%       2.250      4.250      5.083
             120.000      10.267      -0.614      12.667    1.67%       6.083      8.250
     BASHKR               18.519       1.282       5.330    0.89%      12.330     16.500     17.750
             112.000      48.308       2.780      49.000    0.89%      18.670     23.330
     BASSSSC               1.333       0.640       0.000   53.33%       0.000      0.000      0.000
             120.000       2.606      -1.294       4.000   17.50%       1.000      3.000
     PIL                   3.027      -0.004       1.250    0.15%       2.500      2.889      3.000
             653.000       0.371       0.003       5.000    0.15%       3.222      3.556
     TSBASE               36.711       0.258       0.000    3.08%      13.000     27.000     36.000
             682.000     594.554      -0.917      91.000    0.59%      41.000     60.000
     BASAGEC90             0.106       0.407      -3.917    0.81%      -2.833     -1.250     -0.792
             124.000       8.313      -1.090       6.417    0.81%       0.500      3.083
     SEX                   0.000      -1.426      -0.790   20.97%      -0.790      0.210      0.210
             124.000       0.166       0.035       0.210   79.03%       0.210      0.210
     YOL                   0.000       0.868      -3.387   20.97%      -3.387     -1.387     -1.387
             124.000       8.737      -0.315       5.613   17.74%       0.613      0.613


THE MODEL ESTIMATION TERMINATED NORMALLY

     USE THE FBITERATIONS OPTION TO INCREASE THE NUMBER OF ITERATIONS BY A FACTOR
     OF AT LEAST TWO TO CHECK CONVERGENCE AND THAT THE PSR VALUE DOES NOT INCREASE.



MODEL FIT INFORMATION

Number of Free Parameters                              24

Information Criteria

          Deviance (DIC)                             2711.800
          Estimated Number of Parameters (pD)         121.730



MODEL RESULTS

                                Posterior  One-Tailed         95% C.I.
                    Estimate       S.D.      P-Value   Lower 2.5%  Upper 2.5%  Significance

Within Level

Between Level

 PHYSCON  BY
    BASHKR             1.000       0.000      0.000       1.000       1.000
    BASSSSC            0.427       0.623      0.000       0.162       2.348      *

 BASTTDC    ON
    PHYSCON            0.690       0.882      0.000       0.289       3.264      *
    SLOPEPIL           0.410      19.204      0.491     -37.719      37.669
    LNVPIL             0.786       0.862      0.177      -0.977       2.423

 BASTTDC    ON
    PIL                0.287       0.673      0.333      -1.039       1.615
    SEX                1.688       0.749      0.010       0.264       3.205      *
    YOL               -0.272       0.094      0.002      -0.455      -0.088      *
    BASAGEC90         -0.230       0.096      0.009      -0.419      -0.042      *

 PIL      WITH
    SLOPEPIL          -0.007       0.003      0.000      -0.013      -0.003      *
    LNVPIL            -0.074       0.076      0.155      -0.229       0.073

 SLOPEPIL WITH
    LNVPIL             0.002       0.003      0.251      -0.004       0.008

 Means
    PIL                3.240       0.067      0.000       3.108       3.373      *
    SLOPEPIL          -0.011       0.003      0.000      -0.018      -0.005      *
    LNVPIL            -1.963       0.127      0.000      -2.216      -1.716      *
```

```
 Intercepts
    BASTTDC            6.250       2.342      0.004       1.716      10.914      *
    BASHKR            18.483       0.672      0.000      17.164      19.803      *
    BASSSSC            1.330       0.151      0.000       1.033       1.624      *

 Variances
    PIL                0.387       0.077      0.000       0.262       0.564      *
    PHYSCON            6.301       5.543      0.000       0.276      21.136      *
    SLOPEPIL           0.001       0.000      0.000       0.000       0.001      *
    LNVPIL             0.420       0.149      0.000       0.205       0.786      *

 Residual Variances
    BASTTDC            6.322       1.792      0.000       2.400       9.610      *
    BASHKR            43.099       7.417      0.000      29.764      59.043      *
    BASSSSC            1.556       0.627      0.000       0.152       2.571      *


STANDARDIZED MODEL RESULTS


STDYX Standardization

                                Posterior  One-Tailed         95% C.I.
                    Estimate       S.D.      P-Value   Lower 2.5%  Upper 2.5%  Significance

Within-Level Standardized Estimates Averaged Over Clusters

 SLOPEPIL | PIL ON
    TSBASE            -0.137       0.025      0.000      -0.185      -0.086      *

 LNVPIL |
    PIL                0.852       0.014      0.000       0.823       0.880      *

Between Level

 PHYSCON  BY
    BASHKR             0.355       0.139      0.000       0.075       0.621      *
    BASSSSC            0.649       0.167      0.000       0.339       0.972      *

 BASTTDC    ON
    PHYSCON            0.516       0.141      0.000       0.256       0.808      *
    SLOPEPIL           0.003       0.139      0.491      -0.270       0.275
    LNVPIL             0.156       0.160      0.177      -0.177       0.445

 BASTTDC    ON
    PIL                0.054       0.125      0.333      -0.189       0.300
    SEX                0.148       0.063      0.010       0.023       0.270      *
    YOL               -0.173       0.058      0.002      -0.284      -0.056      *
    BASAGEC90         -0.133       0.055      0.009      -0.238      -0.024      *

 PIL      WITH
    SLOPEPIL          -0.461       0.117      0.000      -0.660      -0.204      *
    LNVPIL            -0.190       0.179      0.155      -0.518       0.173

 SLOPEPIL WITH
    LNVPIL             0.115       0.166      0.251      -0.219       0.426

 Means
    PIL                5.209       0.520      0.000       4.302       6.344      *
    SLOPEPIL          -0.449       0.135      0.000      -0.717      -0.187      *
    LNVPIL            -3.029       0.514      0.000      -4.237      -2.228      *

 Intercepts
    BASTTDC            1.883       0.694      0.004       0.506       3.228      *
    BASHKR             2.609       0.201      0.000       2.222       3.010      *
    BASSSSC            0.808       0.105      0.000       0.601       1.014      *

 Variances
    PIL                1.000       0.000      0.000       1.000       1.000
    PHYSCON            1.000       0.000      0.000       1.000       1.000
    SLOPEPIL           1.000       0.000      0.000       1.000       1.000
    LNVPIL             1.000       0.000      0.000       1.000       1.000
```

```
 Residual Variances
    BASTTDC            0.577       0.153      0.000       0.213       0.809      *
    BASHKR             0.874       0.101      0.000       0.615       0.994      *
    BASSSSC            0.578       0.222      0.000       0.056       0.885      *


STDY Standardization

                                Posterior  One-Tailed         95% C.I.
                    Estimate       S.D.      P-Value   Lower 2.5%  Upper 2.5%  Significance

Within-Level Standardized Estimates Averaged Over Clusters

 SLOPEPIL | PIL ON
    TSBASE            -0.020       0.008      0.003      -0.036      -0.005      *

 LNVPIL |
    PIL                0.852       0.014      0.000       0.823       0.880      *

Between Level

 PHYSCON  BY
    BASHKR             0.355       0.139      0.000       0.075       0.621      *
    BASSSSC            0.649       0.167      0.000       0.339       0.972      *

 BASTTDC    ON
    PHYSCON            0.516       0.141      0.000       0.256       0.808      *
    SLOPEPIL           0.003       0.139      0.491      -0.270       0.275
    LNVPIL             0.156       0.160      0.177      -0.177       0.445

 BASTTDC    ON
    PIL                0.054       0.125      0.333      -0.189       0.300
    SEX                0.508       0.216      0.010       0.080       0.927      *
    YOL               -0.082       0.028      0.002      -0.134      -0.026      *
    BASAGEC90         -0.069       0.028      0.009      -0.124      -0.012      *

 PIL      WITH
    SLOPEPIL          -0.461       0.117      0.000      -0.660      -0.204      *
    LNVPIL            -0.190       0.179      0.155      -0.518       0.173

 SLOPEPIL WITH
    LNVPIL             0.115       0.166      0.251      -0.219       0.426

 Means
    PIL                5.209       0.520      0.000       4.302       6.344      *
    SLOPEPIL          -0.449       0.135      0.000      -0.717      -0.187      *
    LNVPIL            -3.029       0.514      0.000      -4.237      -2.228      *

 Intercepts
    BASTTDC            1.883       0.694      0.004       0.506       3.228      *
    BASHKR             2.609       0.201      0.000       2.222       3.010      *
    BASSSSC            0.808       0.105      0.000       0.601       1.014      *

 Variances
    PIL                1.000       0.000      0.000       1.000       1.000
    PHYSCON            1.000       0.000      0.000       1.000       1.000
    SLOPEPIL           1.000       0.000      0.000       1.000       1.000
    LNVPIL             1.000       0.000      0.000       1.000       1.000

 Residual Variances
    BASTTDC            0.577       0.153      0.000       0.213       0.809      *
    BASHKR             0.874       0.101      0.000       0.615       0.994      *
    BASSSSC            0.578       0.222      0.000       0.056       0.885      *


STD Standardization

                                Posterior  One-Tailed         95% C.I.
                    Estimate       S.D.      P-Value   Lower 2.5%  Upper 2.5%  Significance

Within-Level Standardized Estimates Averaged Over Clusters

 SLOPEPIL | PIL ON
    TSBASE            -0.008       0.003      0.001      -0.013      -0.003      *
```

```
 LNVPIL |
    PIL                0.171       0.015      0.000       0.141       0.201      *

Between Level

 PHYSCON  BY
    BASHKR             2.510       1.031      0.000       0.526       4.597      *
    BASSSSC            1.072       0.292      0.000       0.545       1.650      *

 BASTTDC    ON
    PHYSCON            1.720       0.503      0.000       0.831       2.787      *
    SLOPEPIL           0.010       0.468      0.491      -0.918       0.919
    LNVPIL             0.518       0.541      0.177      -0.592       1.519

 BASTTDC    ON
    PIL                0.287       0.673      0.333      -1.039       1.615
    SEX                1.688       0.749      0.010       0.264       3.205      *
    YOL               -0.272       0.094      0.002      -0.455      -0.088      *
    BASAGEC90         -0.230       0.096      0.009      -0.419      -0.042      *

 PIL      WITH
    SLOPEPIL          -0.285       0.090      0.000      -0.468      -0.115      *
    LNVPIL            -0.117       0.114      0.155      -0.336       0.109

 SLOPEPIL WITH
    LNVPIL             0.115       0.166      0.251      -0.219       0.426

 Means
    PIL                3.240       0.067      0.000       3.108       3.373      *
    SLOPEPIL          -0.449       0.135      0.000      -0.717      -0.187      *
    LNVPIL            -3.029       0.514      0.000      -4.237      -2.228      *

 Intercepts
    BASTTDC            6.250       2.342      0.004       1.716      10.914      *
    BASHKR            18.483       0.672      0.000      17.164      19.803      *
    BASSSSC            1.330       0.151      0.000       1.033       1.624      *

 Variances
    PIL                0.387       0.077      0.000       0.262       0.564      *
    PHYSCON            1.000       0.000      0.000       1.000       1.000
    SLOPEPIL           1.000       0.000      0.000       1.000       1.000
    LNVPIL             1.000       0.000      0.000       1.000       1.000

 Residual Variances
    BASTTDC            6.322       1.792      0.000       2.400       9.610      *
    BASHKR            43.099       7.417      0.000      29.764      59.043      *
    BASSSSC            1.556       0.627      0.000       0.152       2.571      *


R-SQUARE

Within-Level R-Square Averaged Across Clusters

                                Posterior  One-Tailed         95% C.I.
    Variable        Estimate       S.D.      P-Value   Lower 2.5%  Upper 2.5%

    PIL                0.148       0.014      0.000       0.120       0.177

Between Level

                                Posterior  One-Tailed         95% C.I.
    Variable        Estimate       S.D.      P-Value   Lower 2.5%  Upper 2.5%

    BASTTDC            0.423       0.153      0.000       0.191       0.787
    BASHKR             0.126       0.101      0.000       0.006       0.385
    BASSSSC            0.422       0.222      0.000       0.115       0.944


TECHNICAL 8 OUTPUT


   TECHNICAL 8 OUTPUT FOR BAYES ESTIMATION
```

```
CHAIN    BSEED
1        0
2        285380

                POTENTIAL       PARAMETER WITH
ITERATION    SCALE REDUCTION      HIGHEST PSR
100              2.747               12
200              2.855               20
300              1.967               20
400              2.104               7
500              1.816               7
600              1.547               7
700              1.230               12
800              1.193               12
900              1.271               5
1000             1.122               5
1100             1.064               5
1200             1.053               7
1300             1.063               3
1400             1.122               20
1500             1.118               17
1600             1.162               17
1700             1.141               17
1800             1.048               7
1900             1.049               23
2000             1.051               23
2100             1.100               10
2200             1.156               10
2300             1.191               10
2400             1.252               10
2500             1.241               10
2600             1.291               17
2700             1.302               17
2800             1.313               17
2900             1.336               17
3000             1.397               17
3100             1.534               17
3200             1.601               17
3300             1.703               17
3400             1.718               17
3500             1.739               17
3600             1.722               17
3700             1.636               17
3800             1.619               17
3900             1.629               17
4000             1.672               17
4100             1.864               17
4200             1.906               17
4300             1.917               17
4400             1.883               17
4500             1.870               17
4600             1.838               17
4700             1.831               17
4800             1.773               17
4900             1.756               17
5000             1.782               17
5100             1.772               17
5200             1.658               17
5300             1.665               17
5400             1.698               10
5500             1.736               10
5600             1.758               10
5700             1.801               10
5800             1.845               10
5900             1.885               10
6000             1.861               10
6100             1.853               10
6200             1.867               10
6300             1.879               10
6400             1.867               10
6500             1.874               10
6600             1.881               10
6700             1.902               10
```

```
6800            1.897           10
6900            1.881           10
7000            1.880           10
7100            1.873           10
7200            1.895           10
7300            1.914           10
7400            1.905           10
7500            1.871           10
7600            1.832           10
7700            1.817           10
7800            1.804           10
7900            1.795           10
8000            1.784           10
8100            1.780           10
8200            1.780           10
8300            1.603           10
8400            1.566           17
8500            1.561           17
8600            1.535           17
8700            1.533           17
8800            1.543           17
8900            1.553           17
9000            1.558           17
9100            1.563           17
9200            1.554           17
9300            1.556           17
9400            1.558           17
9500            1.566           17
9600            1.570           17
9700            1.579           17
9800            1.582           17
9900            1.587           17
10000           1.578           17
10100           1.577           17
10200           1.582           17
10300           1.592           17
10400           1.599           17
10500           1.598           17
10600           1.603           17
10700           1.606           17
10800           1.596           17
10900           1.592           17
11000           1.583           17
11100           1.571           17
11200           1.554           17
11300           1.565           17
11400           1.572           17
11500           1.567           17
11600           1.552           17
11700           1.513           17
11800           1.469           17
11900           1.441           17
12000           1.419           17
12100           1.403           17
12200           1.405           17
12300           1.417           17
12400           1.419           17
12500           1.417           17
12600           1.414           17
12700           1.401           17
12800           1.403           17
12900           1.401           17
13000           1.401           17
13100           1.382           17
13200           1.381           17
13300           1.378           17
13400           1.361           17
13500           1.339           17
13600           1.322           17
13700           1.314           17
13800           1.306           17
13900           1.282           17
14000           1.274           17
14100           1.269           17
```

```
     14200             1.262              17
     14300             1.251              17
     14400             1.251              17
     14500             1.252              17
     14600             1.249              17
     14700             1.241              17
     14800             1.237              17
     14900             1.231              17
     15000             1.225              17
     15100             1.222              17
     15200             1.222              17
     15300             1.211              17
     15400             1.194              17
     15500             1.174              17
     15600             1.164              17
     15700             1.152              17
     15800             1.141              17
     15900             1.137              17
     16000             1.136              17
     16100             1.133              17
     16200             1.125              17
     16300             1.119              17
     16400             1.115              17
     16500             1.103              17
     16600             1.087              17
     16700             1.077              17
     16800             1.073              17
     16900             1.072              17
     17000             1.065              17
     17100             1.060              17
     17200             1.056              17
     17300             1.054              17
     17400             1.050              17
     17500             1.046              17
     17600             1.045              17
     17700             1.045              17
     17800             1.039              17
     17900             1.043              10
     18000             1.046              3
     18100             1.046              3
     18200             1.042              3
     18300             1.039              3
     18400             1.035              3
     18500             1.031              3
     18600             1.028              3
     18700             1.025              3
     18800             1.024              3
     18900             1.027              3
     19000             1.025              3
     19100             1.023              3
     19200             1.025              3
     19300             1.027              3
     19400             1.025              3
     19500             1.022              3
     19600             1.020              3
     19700             1.016              3
     19800             1.014              3
     19900             1.011              3
     20000             1.009              3
     20100             1.007              3
     20200             1.007              7
     20300             1.007              7
     20400             1.008              7
     20500             1.008              7
     20600             1.008              7
     20700             1.008              7
     20800             1.009              21
     20900             1.010              21
     21000             1.010              21
     21100             1.010              21
     21200             1.011              21
     21300             1.011              21
     21400             1.011              21
     21500             1.010              21
```

```
    21600             1.010               7
    21700             1.010               7
    21800             1.011               7
    21900             1.012               7
    22000             1.013               7
    22100             1.014               7
    22200             1.015               7
    22300             1.015               7
    22400             1.016               7
    22500             1.017               7
    22600             1.017               7
    22700             1.017               7
    22800             1.016               7
    22900             1.017               7
    23000             1.019               7
    23100             1.018               7
    23200             1.017               7
    23300             1.017               7
    23400             1.019               7
    23500             1.022               7
    23600             1.023               7
    23700             1.027               7
    23800             1.028               7
    23900             1.028               7
    24000             1.029               7
    24100             1.030               7
    24200             1.030               7
    24300             1.028               7
    24400             1.026               7
    24500             1.025               7
    24600             1.026               7
    24700             1.026               7
    24800             1.028               7
    24900             1.030               7
    25000             1.030               7
    25100             1.030               7
    25200             1.030               7
    25300             1.031               7
    25400             1.030               7
    25500             1.031               7
    25600             1.032               7
    25700             1.032               7
    25800             1.030               7
    25900             1.030               7
    26000             1.029               7
    26100             1.028               7
    26200             1.028               7
    26300             1.027               7
    26400             1.026               7
    26500             1.024               7
    26600             1.020               7
    26700             1.017               7
    26800             1.016               7
    26900             1.016               7
    27000             1.016               7
    27100             1.017               7
    27200             1.017               7
    27300             1.017               7
    27400             1.017               7
    27500             1.017               7
    27600             1.015               7
    27700             1.014               7
    27800             1.013               7
    27900             1.013               7
    28000             1.012               7
    28100             1.011               7
    28200             1.011               7
    28300             1.012               7
    28400             1.012               7
    28500             1.012               7
    28600             1.013               7
    28700             1.013               7
    28800             1.013               7
    28900             1.014               7
```

29000	1.015	7
29100	1.014	7
29200	1.013	7
29300	1.013	7
29400	1.012	7
29500	1.013	7
29600	1.013	7
29700	1.015	7
29800	1.016	7
29900	1.018	7
30000	1.019	7
30100	1.018	7
30200	1.016	7
30300	1.014	7
30400	1.013	7
30500	1.012	7
30600	1.011	7
30700	1.010	7
30800	1.009	7
30900	1.009	7
31000	1.008	7
31100	1.009	7
31200	1.010	7
31300	1.011	7
31400	1.011	7
31500	1.010	7
31600	1.009	7
31700	1.009	7
31800	1.009	7
31900	1.009	7
32000	1.009	7
32100	1.009	7
32200	1.009	7
32300	1.009	7
32400	1.009	7
32500	1.009	7
32600	1.009	7
32700	1.009	7
32800	1.008	7
32900	1.007	7
33000	1.006	7
33100	1.006	7
33200	1.007	7
33300	1.009	3
33400	1.011	3
33500	1.013	3
33600	1.015	3
33700	1.018	3
33800	1.020	3
33900	1.022	3
34000	1.024	3
34100	1.026	3
34200	1.029	3
34300	1.030	3
34400	1.031	3
34500	1.031	3
34600	1.031	3
34700	1.031	3
34800	1.031	3
34900	1.031	3
35000	1.031	3
35100	1.031	3
35200	1.031	3
35300	1.031	3
35400	1.031	3
35500	1.031	3
35600	1.031	3
35700	1.031	3
35800	1.030	3
35900	1.030	3
36000	1.030	3
36100	1.030	3
36200	1.030	3
36300	1.030	3

36400	1.030	3
36500	1.030	3
36600	1.030	3
36700	1.029	3
36800	1.029	3
36900	1.028	3
37000	1.028	3
37100	1.027	3
37200	1.027	3
37300	1.027	3
37400	1.027	3
37500	1.027	3
37600	1.027	3
37700	1.027	3
37800	1.027	3
37900	1.026	3
38000	1.027	3
38100	1.026	3
38200	1.026	3
38300	1.026	3
38400	1.026	3
38500	1.026	3
38600	1.026	3
38700	1.026	3
38800	1.026	3
38900	1.026	3
39000	1.026	3
39100	1.026	3
39200	1.025	3
39300	1.025	3
39400	1.025	3
39500	1.025	3
39600	1.025	3
39700	1.025	3
39800	1.025	3
39900	1.025	3
40000	1.026	3
40100	1.025	3
40200	1.025	3
40300	1.025	3
40400	1.025	3
40500	1.025	3
40600	1.025	3
40700	1.025	3
40800	1.025	3
40900	1.025	3
41000	1.025	3
41100	1.025	3
41200	1.025	3
41300	1.025	3
41400	1.025	3
41500	1.024	3
41600	1.024	3
41700	1.024	3
41800	1.024	3
41900	1.024	3
42000	1.024	3
42100	1.024	3
42200	1.024	3
42300	1.023	3
42400	1.023	3
42500	1.024	3
42600	1.024	3
42700	1.023	3
42800	1.023	3
42900	1.023	3
43000	1.024	3
43100	1.024	3
43200	1.023	3
43300	1.023	3
43400	1.023	3
43500	1.023	3
43600	1.022	3
43700	1.022	3

43800	1.022	3
43900	1.022	3
44000	1.022	3
44100	1.022	3
44200	1.022	3
44300	1.022	3
44400	1.022	3
44500	1.022	3
44600	1.022	3
44700	1.022	3
44800	1.022	3
44900	1.022	3
45000	1.022	3
45100	1.022	3
45200	1.022	3
45300	1.022	3
45400	1.021	3
45500	1.021	3
45600	1.021	3
45700	1.021	3
45800	1.021	3
45900	1.021	3
46000	1.021	3
46100	1.021	3
46200	1.022	3
46300	1.022	3
46400	1.022	3
46500	1.022	3
46600	1.022	3
46700	1.021	3
46800	1.021	3
46900	1.021	3
47000	1.021	3
47100	1.021	3
47200	1.021	3
47300	1.021	3
47400	1.021	3
47500	1.021	3
47600	1.021	3
47700	1.021	3
47800	1.020	3
47900	1.020	3
48000	1.020	3
48100	1.020	3
48200	1.020	3
48300	1.020	3
48400	1.020	3
48500	1.020	3
48600	1.020	3
48700	1.020	3
48800	1.020	3
48900	1.020	3
49000	1.020	3
49100	1.020	3
49200	1.020	3
49300	1.020	3
49400	1.020	3
49500	1.020	3
49600	1.020	3
49700	1.019	3
49800	1.019	3
49900	1.019	3
50000	1.019	3
50100	1.020	3
50200	1.020	3
50300	1.020	3
50400	1.019	3
50500	1.019	3
50600	1.019	3
50700	1.019	3
50800	1.019	3
50900	1.019	3
51000	1.019	3
51100	1.019	3

51200	1.019	3
51300	1.019	3
51400	1.019	3
51500	1.019	3
51600	1.019	3
51700	1.019	3
51800	1.020	3
51900	1.020	3
52000	1.020	3
52100	1.020	3
52200	1.020	3
52300	1.020	3
52400	1.020	3
52500	1.020	3
52600	1.021	3
52700	1.021	3
52800	1.021	3
52900	1.021	3
53000	1.021	3
53100	1.021	3
53200	1.021	3
53300	1.021	3
53400	1.021	3
53500	1.021	3
53600	1.021	3
53700	1.021	3
53800	1.021	3
53900	1.021	3
54000	1.021	3
54100	1.021	3
54200	1.021	3
54300	1.021	3
54400	1.021	3
54500	1.021	3
54600	1.021	3
54700	1.021	3
54800	1.021	3
54900	1.021	3
55000	1.021	3
55100	1.021	3
55200	1.021	3
55300	1.021	3
55400	1.021	3
55500	1.021	3
55600	1.021	3
55700	1.021	3
55800	1.021	3
55900	1.021	3
56000	1.021	3
56100	1.020	3
56200	1.020	3
56300	1.020	3
56400	1.020	3
56500	1.020	3
56600	1.020	3
56700	1.020	3
56800	1.020	3
56900	1.020	3
57000	1.020	3
57100	1.020	3
57200	1.020	3
57300	1.020	3
57400	1.020	3
57500	1.019	3
57600	1.019	3
57700	1.019	3
57800	1.019	3
57900	1.019	3
58000	1.019	3
58100	1.019	3
58200	1.019	10
58300	1.018	10
58400	1.018	10
58500	1.018	10

```
58600          1.018          10
58700          1.018          10
58800          1.018          10
58900          1.017          10
59000          1.017          10
59100          1.017          10
59200          1.017          10
59300          1.017          10
59400          1.017          10
59500          1.017          3
59600          1.017          3
59700          1.017          3
59800          1.017          3
59900          1.017          3
60000          1.017          3
60100          1.017          3
60200          1.017          3
60300          1.017          3
60400          1.017          3
60500          1.017          3
60600          1.017          3
60700          1.017          3
60800          1.017          3
60900          1.017          3
61000          1.017          3
61100          1.017          3
61200          1.017          3
61300          1.017          3
61400          1.017          3
61500          1.017          3
61600          1.017          3
61700          1.017          3
61800          1.017          3
61900          1.017          3
62000          1.017          3
62100          1.017          3
62200          1.017          3
62300          1.017          3
62400          1.017          3
62500          1.017          3
62600          1.017          3
62700          1.017          3
62800          1.017          3
62900          1.017          3
63000          1.017          3
63100          1.018          3
63200          1.018          3
63300          1.018          3
63400          1.018          3
63500          1.018          3
63600          1.018          3
63700          1.018          3
63800          1.018          3
63900          1.018          3
64000          1.018          3
64100          1.018          3
64200          1.018          3
64300          1.017          3
64400          1.017          3
64500          1.017          3
64600          1.017          3
64700          1.017          3
64800          1.017          3
64900          1.017          3
65000          1.017          3
65100          1.017          3
65200          1.017          3
65300          1.017          3
65400          1.017          3
65500          1.017          3
65600          1.017          3
65700          1.018          3
65800          1.018          3
65900          1.019          3
```

66000	1.018	3
66100	1.018	10
66200	1.018	10
66300	1.018	10
66400	1.020	10
66500	1.022	10
66600	1.023	3
66700	1.024	3
66800	1.025	3
66900	1.025	3
67000	1.026	3
67100	1.026	3
67200	1.027	3
67300	1.027	3
67400	1.027	3
67500	1.027	3
67600	1.028	3
67700	1.028	3
67800	1.029	3
67900	1.029	3
68000	1.029	3
68100	1.029	3
68200	1.029	3
68300	1.029	3
68400	1.029	3
68500	1.029	3
68600	1.029	3
68700	1.029	3
68800	1.030	3
68900	1.030	3
69000	1.031	3
69100	1.032	3
69200	1.032	3
69300	1.032	3
69400	1.032	3
69500	1.032	3
69600	1.032	3
69700	1.032	3
69800	1.032	3
69900	1.032	3
70000	1.032	3
70100	1.032	3
70200	1.031	3
70300	1.032	3
70400	1.032	3
70500	1.032	3
70600	1.032	3
70700	1.032	3
70800	1.032	3
70900	1.032	3
71000	1.032	3
71100	1.031	3
71200	1.031	3
71300	1.031	3
71400	1.031	3
71500	1.031	3
71600	1.031	3
71700	1.030	3
71800	1.030	3
71900	1.030	3
72000	1.030	3
72100	1.030	3
72200	1.030	3
72300	1.030	3
72400	1.030	3
72500	1.030	3
72600	1.030	3
72700	1.030	3
72800	1.030	3
72900	1.030	3
73000	1.030	3
73100	1.030	3
73200	1.030	3
73300	1.030	3

```
73400           1.030           3
73500           1.030           3
73600           1.030           3
73700           1.030           3
73800           1.030           3
73900           1.030           3
74000           1.030           3
74100           1.030           3
74200           1.030           3
74300           1.030           3
74400           1.030           3
74500           1.030           3
74600           1.030           3
74700           1.030           3
74800           1.030           3
74900           1.030           3
75000           1.030           3
75100           1.029           3
75200           1.029           3
75300           1.029           3
75400           1.029           3
75500           1.029           3
75600           1.029           3
75700           1.029           3
75800           1.029           3
75900           1.029           3
76000           1.029           3
76100           1.029           3
76200           1.029           3
76300           1.029           3
76400           1.029           3
76500           1.029           3
76600           1.029           3
76700           1.029           3
76800           1.029           3
76900           1.029           3
77000           1.028           3
77100           1.028           3
77200           1.028           3
77300           1.028           3
77400           1.028           3
77500           1.028           3
77600           1.028           3
77700           1.028           3
77800           1.028           3
77900           1.028           3
78000           1.028           3
78100           1.028           3
78200           1.028           3
78300           1.028           3
78400           1.028           3
78500           1.028           3
78600           1.028           3
78700           1.028           3
78800           1.028           3
78900           1.028           3
79000           1.028           3
79100           1.028           3
79200           1.028           3
79300           1.027           3
79400           1.027           3
79500           1.027           3
79600           1.026           3
79700           1.026           3
79800           1.026           3
79900           1.026           3
80000           1.026           3
80100           1.026           3
80200           1.026           3
80300           1.026           3
80400           1.026           3
80500           1.026           3
80600           1.026           3
80700           1.026           3
```

80800	1.026	3
80900	1.026	3
81000	1.026	3
81100	1.026	3
81200	1.026	3
81300	1.025	3
81400	1.026	3
81500	1.025	3
81600	1.025	3
81700	1.025	3
81800	1.025	3
81900	1.025	3
82000	1.025	3
82100	1.025	3
82200	1.025	3
82300	1.025	3
82400	1.025	3
82500	1.025	3
82600	1.025	3
82700	1.025	3
82800	1.025	3
82900	1.025	3
83000	1.025	3
83100	1.025	3
83200	1.025	3
83300	1.025	3
83400	1.025	3
83500	1.025	3
83600	1.025	3
83700	1.026	3
83800	1.026	3
83900	1.026	3
84000	1.026	3
84100	1.026	3
84200	1.026	3
84300	1.026	3
84400	1.026	3
84500	1.026	3
84600	1.026	3
84700	1.025	3
84800	1.025	3
84900	1.025	3
85000	1.025	3
85100	1.025	3
85200	1.024	3
85300	1.024	3
85400	1.024	3
85500	1.024	3
85600	1.024	3
85700	1.024	3
85800	1.024	3
85900	1.024	3
86000	1.024	3
86100	1.024	3
86200	1.024	3
86300	1.024	3
86400	1.024	3
86500	1.024	3
86600	1.024	3
86700	1.024	3
86800	1.024	3
86900	1.024	3
87000	1.024	3
87100	1.024	3
87200	1.024	3
87300	1.025	3
87400	1.025	3
87500	1.025	3
87600	1.025	3
87700	1.025	3
87800	1.025	3
87900	1.026	3
88000	1.026	3
88100	1.026	3

```
88200          1.027          3
88300          1.027          3
88400          1.027          3
88500          1.027          3
88600          1.027          3
88700          1.027          3
88800          1.027          3
88900          1.027          3
89000          1.027          3
89100          1.027          3
89200          1.027          3
89300          1.027          3
89400          1.027          3
89500          1.027          3
89600          1.027          3
89700          1.028          3
89800          1.028          3
89900          1.028          3
90000          1.028          3
90100          1.028          3
90200          1.028          3
90300          1.028          3
90400          1.028          3
90500          1.028          3
90600          1.027          3
90700          1.027          3
90800          1.027          3
90900          1.027          3
91000          1.027          3
91100          1.027          3
91200          1.027          3
91300          1.027          3
91400          1.027          3
91500          1.027          3
91600          1.027          3
91700          1.027          3
91800          1.027          3
91900          1.027          3
92000          1.028          3
92100          1.029          3
92200          1.029          3
92300          1.030          3
92400          1.030          3
92500          1.030          3
92600          1.030          3
92700          1.030          3
92800          1.030          3
92900          1.030          3
93000          1.030          3
93100          1.030          3
93200          1.030          3
93300          1.030          3
93400          1.030          3
93500          1.030          3
93600          1.030          3
93700          1.030          3
93800          1.030          3
93900          1.030          3
94000          1.030          3
94100          1.030          3
94200          1.030          3
94300          1.030          3
94400          1.030          3
94500          1.030          3
94600          1.030          3
94700          1.030          3
94800          1.030          3
94900          1.030          3
95000          1.030          3
95100          1.030          3
95200          1.030          3
95300          1.030          3
95400          1.030          3
95500          1.030          3
```

95600	1.031	3
95700	1.031	3
95800	1.031	3
95900	1.031	3
96000	1.031	3
96100	1.031	3
96200	1.031	3
96300	1.031	3
96400	1.031	3
96500	1.031	3
96600	1.031	3
96700	1.031	3
96800	1.031	3
96900	1.031	3
97000	1.031	3
97100	1.031	3
97200	1.031	3
97300	1.031	3
97400	1.031	3
97500	1.031	3
97600	1.030	3
97700	1.030	3
97800	1.030	3
97900	1.030	3
98000	1.030	3
98100	1.030	3
98200	1.030	3
98300	1.030	3
98400	1.030	3
98500	1.030	3
98600	1.030	3
98700	1.030	3
98800	1.030	3
98900	1.030	3
99000	1.030	3
99100	1.030	3
99200	1.030	3
99300	1.030	3
99400	1.029	3
99500	1.029	3
99600	1.029	3
99700	1.029	3
99800	1.029	3
99900	1.029	3
100000	1.029	3
100100	1.029	3
100200	1.029	3
100300	1.029	3
100400	1.029	3
100500	1.029	3
100600	1.029	3
100700	1.029	3
100800	1.029	3
100900	1.029	3
101000	1.029	3
101100	1.029	3
101200	1.029	3
101300	1.029	3
101400	1.029	3
101500	1.029	3
101600	1.029	3
101700	1.029	3
101800	1.029	3
101900	1.029	3
102000	1.029	3
102100	1.029	3
102200	1.029	3
102300	1.028	3
102400	1.028	3
102500	1.028	3
102600	1.028	3
102700	1.028	3
102800	1.028	3
102900	1.028	3

```
     103000           1.028              3
     103100           1.028              3
     103200           1.028              3
     103300           1.028              3
     103400           1.028              3
     103500           1.028              3
     103600           1.027              3
     103700           1.028              3
     103800           1.028              3
     103900           1.028              3
     104000           1.028              3
     104100           1.028              3
     104200           1.028              3
     104300           1.028              3
     104400           1.028              3
     104500           1.028              3
     104600           1.028              3
     104700           1.028              3
     104800           1.028              3
     104900           1.028              3
     105000           1.028              3
     105100           1.027              3
     105200           1.027              3
     105300           1.027              3
     105400           1.027              3
     105500           1.027              3
     105600           1.027              3
     105700           1.027              3
     105800           1.027              3
     105900           1.027              3
     106000           1.026              3
     106100           1.026              3
     106200           1.026              3
     106300           1.026              3
     106400           1.026              3
     106500           1.026              3
     106600           1.026              3
     106700           1.026              3
     106800           1.026              3
     106900           1.026              3
     107000           1.026              3
     107100           1.026              3
     107200           1.026              3
     107300           1.026              3
     107400           1.026              3
     107500           1.026              3
     107600           1.026              3
     107700           1.026              3
     107800           1.026              3
     107900           1.026              3
     108000           1.026              3
     108100           1.025              3
     108200           1.025              3
     108300           1.025              3
     108400           1.025              3
     108500           1.025              3
     108600           1.025              3
     108700           1.025              3
     108800           1.025              3
     108900           1.025              3
     109000           1.025              3
     109100           1.025              3
     109200           1.025              3
     109300           1.025              3
     109400           1.025              3
     109500           1.025              3
     109600           1.025              3
     109700           1.025              3
     109800           1.025              3
     109900           1.025              3
     110000           1.025              3
     110100           1.025              3
     110200           1.024              3
     110300           1.024              3
```

```
110400          1.024          3
110500          1.024          3
110600          1.024          3
110700          1.024          3
110800          1.024          3
110900          1.024          3
111000          1.024          3
111100          1.024          3
111200          1.024          3
111300          1.024          3
111400          1.024          3
111500          1.024          3
111600          1.024          3
111700          1.024          3
111800          1.023          3
111900          1.023          3
112000          1.024          3
112100          1.024          3
112200          1.024          3
112300          1.024          3
112400          1.024          3
112500          1.023          3
112600          1.023          3
112700          1.023          3
112800          1.024          3
112900          1.024          3
113000          1.024          3
113100          1.024          3
113200          1.024          3
113300          1.024          3
113400          1.024          3
113500          1.024          3
113600          1.024          3
113700          1.024          3
113800          1.024          3
113900          1.024          3
114000          1.024          3
114100          1.024          3
114200          1.024          3
114300          1.024          3
114400          1.024          3
114500          1.024          3
114600          1.024          3
114700          1.023          3
114800          1.023          3
114900          1.024          3
115000          1.024          3
115100          1.024          3
115200          1.024          3
115300          1.023          3
115400          1.023          3
115500          1.023          3
115600          1.023          3
115700          1.023          3
115800          1.023          3
115900          1.023          3
116000          1.023          3
116100          1.023          3
116200          1.024          3
116300          1.024          3
116400          1.024          3
116500          1.024          3
116600          1.024          3
116700          1.025          3
116800          1.025          3
116900          1.025          3
117000          1.025          3
117100          1.025          3
117200          1.025          3
117300          1.025          3
117400          1.025          3
117500          1.025          3
117600          1.025          3
117700          1.025          3
```

117800	1.025	3
117900	1.025	3
118000	1.025	3
118100	1.025	3
118200	1.025	3
118300	1.025	3
118400	1.025	3
118500	1.025	3
118600	1.025	3
118700	1.025	3
118800	1.025	3
118900	1.025	3
119000	1.025	3
119100	1.025	3
119200	1.025	3
119300	1.025	3
119400	1.025	3
119500	1.025	3
119600	1.025	3
119700	1.025	3
119800	1.025	3
119900	1.025	3
120000	1.024	3
120100	1.024	3
120200	1.024	3
120300	1.023	3
120400	1.023	3
120500	1.023	3
120600	1.022	3
120700	1.022	3
120800	1.022	3
120900	1.021	3
121000	1.021	3
121100	1.021	3
121200	1.020	3
121300	1.020	3
121400	1.020	3
121500	1.020	3
121600	1.020	3
121700	1.019	3
121800	1.019	3
121900	1.019	3
122000	1.019	3
122100	1.019	3
122200	1.019	3
122300	1.019	3
122400	1.019	3
122500	1.019	3
122600	1.019	3
122700	1.019	3
122800	1.019	3
122900	1.019	3
123000	1.019	3
123100	1.019	3
123200	1.019	3
123300	1.019	3
123400	1.019	3
123500	1.019	3
123600	1.019	3
123700	1.019	3
123800	1.019	3
123900	1.019	3
124000	1.019	3
124100	1.019	3
124200	1.019	3
124300	1.019	3
124400	1.019	3
124500	1.019	3
124600	1.019	3
124700	1.019	3
124800	1.019	3
124900	1.019	3
125000	1.019	3
125100	1.019	3

125200	1.019	3
125300	1.019	3
125400	1.018	3
125500	1.018	3
125600	1.018	3
125700	1.018	3
125800	1.018	3
125900	1.018	3
126000	1.017	3
126100	1.017	3
126200	1.017	3
126300	1.017	3
126400	1.017	3
126500	1.017	3
126600	1.017	3
126700	1.017	3
126800	1.017	3
126900	1.017	3
127000	1.017	3
127100	1.017	3
127200	1.017	3
127300	1.017	3
127400	1.017	3
127500	1.017	3
127600	1.017	3
127700	1.017	3
127800	1.017	3
127900	1.017	3
128000	1.017	3
128100	1.017	3
128200	1.017	3
128300	1.017	3
128400	1.017	3
128500	1.017	3
128600	1.017	3
128700	1.017	3
128800	1.017	3
128900	1.017	3
129000	1.017	3
129100	1.017	3
129200	1.017	3
129300	1.017	3
129400	1.017	3
129500	1.017	3
129600	1.017	3
129700	1.017	3
129800	1.017	3
129900	1.017	3
130000	1.017	3
130100	1.017	3
130200	1.017	3
130300	1.017	3
130400	1.017	3
130500	1.017	3
130600	1.017	3
130700	1.017	3
130800	1.017	3
130900	1.017	3
131000	1.017	3
131100	1.017	3
131200	1.016	3
131300	1.016	3
131400	1.016	3
131500	1.016	3
131600	1.015	3
131700	1.015	3
131800	1.015	3
131900	1.015	3
132000	1.014	3
132100	1.014	3
132200	1.014	3
132300	1.014	3
132400	1.014	3
132500	1.014	3

```
     132600           1.013             3
     132700           1.013             3
     132800           1.012             3
     132900           1.012             3
     133000           1.012             3
     133100           1.012             3
     133200           1.011             3
     133300           1.011             3
     133400           1.011             3
     133500           1.010             3
     133600           1.010             3
     133700           1.010             3
     133800           1.009             3
     133900           1.009             3
     134000           1.009             3
     134100           1.008             3
     134200           1.008             3
     134300           1.008             3
     134400           1.007             3
     134500           1.007             3
     134600           1.007             3
     134700           1.008             3
     134800           1.007             3
     134900           1.007             3
     135000           1.007             3
     135100           1.007             3
     135200           1.006             3
     135300           1.006             3
     135400           1.006             3
     135500           1.005             3
     135600           1.005             3
     135700           1.005             3
     135800           1.004             3
     135900           1.004             3
     136000           1.004             3
     136100           1.003             3
     136200           1.003             3
     136300           1.003             3
     136400           1.003             3
     136500           1.002             20
     136600           1.002             20
     136700           1.002             20
     136800           1.002             20
```

TECHNICAL 5 OUTPUT

DIAGRAM INFORMATION

Mplus diagrams are currently not available for multilevel analysis.
No diagram output was produced.

```
     Beginning Time:  19:52:01
        Ending Time:  20:15:42
       Elapsed Time:  00:23:41
```

MUTHEN & MUTHEN
3463 Stoner Ave.
Los Angeles, CA 90066

Tel: (310) 391-9971
Fax: (310) 391-8971
Web: www.StatModel.com
Support: Support@StatModel.com

Copyright (c) 1998-2020 Muthen & Muthen

Mplus Output Single Multilevel SEM – Self Acceptance:

```
Mplus VERSION 8.5
MUTHEN & MUTHEN
10/12/2021   4:16 PM

INPUT INSTRUCTIONS

  TITLE:

  DATA: FILE = mpltry6.dat;

  VARIABLE:
   names =  pno wave sex yol verst nopart
         basagec90 agec90 tims tsbase
         ttd ttdcens basettd basttdc
         aut pil ema sac dep
         pa na swls adlinv hyp
         visus sfenerg sfsofunc sfpain sfgen sfchang
         anges anget akzet mortinv mortperc
         basadlinv basvisus bashkr bassssc
         bassfen bassfso bassfpain bassfgen bassfcha
         hkr sl sssco;
  usevariables = sac tsbase basttdc bashkr bassssc basagec90 sex yol;
  cluster = PNO;
  missing = .;
  within = tsbase;
  between = basttdc bashkr bassssc basagec90 sex yol;

 DEFINE:
  center sex yol (grandmean);

 ANALYSIS:
  type = twolevel random;
  estimator = BAYES;
  chains = 2;
  biterations=1000000;
  BCONVERGENCE = 0.00125;

MODEL:

  %WITHIN%

  slopesac | sac ON tsbase;
  lnvsac | sac;

  %BETWEEN%
  physcon by bashkr@1
             bassssc*0.376;

  basttdc ON sac*0.604
             sex*1.631
             yol*-0.220
             physcon*0.691
             basagec90
             slopesac*9.769
             lnvsac*0.823;

  [ basttdc*5.038 ];
  [ bashkr*18.476 ];
  [ bassssc*1.331 ];
  [ sac*4.075 ] (m4);
  [ slopesac*-0.004 ] (ms4);
  [ lnvsac*-2.263 ] (mv4);

  basttdc*6.317;
  sac*0.285;
  slopesac*0.001;
  lnvsac*0.360;

  sac with slopesac*-0.004
```

```
           lnvsac*-0.100;
  slopesac with lnvsac*0.000;

 MODEL PRIORS:
  m4 ~ N(4.03,0.109);
  ms4 ~ N(-0.009,0.00026);

 OUTPUT:
  standardized tech5 tech8 tech16;




*** WARNING in VARIABLE command
  Note that only the first 8 characters of variable names are used in the output.
  Shorten variable names to avoid any confusion.
*** WARNING in MODEL command
  In the MODEL command, the following variable is an x-variable on the BETWEEN
  level and a y-variable on the WITHIN level.  This variable will be treated
  as a y-variable on both levels:  SAC
*** WARNING
  One or more individual-level variables have no variation within a
  cluster for the following clusters.

     Variable   Cluster IDs with no within-cluster variation

      SAC         16142 16798 15510

   3 WARNING(S) FOUND IN THE INPUT INSTRUCTIONS




SUMMARY OF ANALYSIS

Number of groups                                                 1
Number of observations                                         682

Number of dependent variables                                    4
Number of independent variables                                  4
Number of continuous latent variables                            3

Observed dependent variables

  Continuous
   BASTTDC     BASHKR      BASSSSC     SAC

Observed independent variables
   TSBASE      BASAGEC9    SEX         YOL

Continuous latent variables
   PHYSCON     SLOPESAC    LNVSAC

Variables with special functions

  Cluster variable      PNO

  Within variables
   TSBASE

  Between variables
   BASTTDC     BASHKR      BASSSSC     BASAGEC9    SEX         YOL

  Centering (GRANDMEAN)
   SEX         YOL


Estimator                                                    BAYES
Specifications for Bayesian Estimation
  Point estimate                                            MEDIAN
  Number of Markov chain Monte Carlo (MCMC) chains               2
  Random seed for the first chain                                0
  Starting value information                           UNPERTURBED
  Algorithm used for Markov chain Monte Carlo           GIBBS(PX1)
```

```
  Convergence criterion                                  0.125D-02
  Maximum number of iterations                             1000000
  K-th iteration used for thinning                               1

Input data file(s)
  mpltry6.dat
Input data format  FREE


SUMMARY OF DATA

     Number of clusters                       124

       Size (s)    Cluster ID with Size s

          1        10026 10130 10144 10313 10473 10558 10567 10637 10663
                   10893 10901 10907 10911 10939 10969 11157 11240 11294
                   11297 11741 15018 15177 15241 15319 15564 16142 16423
                   16610 16669 16798 17542 18507 18650
          2        15239 11258 10181 15510 10615 11341 16303 11371 16510
                   11384 11485 11028 16862 10211 17780 18004 18252 15175
                   10228
          3        11573 11295 17130 11100 17745 10340 10460 10461 10210
                   11501
          4        10904 11457 10434 11493 10811 17593
          5        15424 10666 12411 11343 11005
          6        10159 15092 15121 15426 11593 11163 10139 16220 19067
          7        10354 11230 10894 10724 10470
          8        10252 10528 11038 10033 11336 11253
          9        15623 11055
         10        12037 10167 17197 15009 19500
         11        11233 10444 15714 10533
         12        11079 10577 15378 12714 10986 10428
         13        11420 11002
         14        11450
         15        10108 10906 10551 15231 10902
         16        15141 10940 16103 15472 11528 11378



COVARIANCE COVERAGE OF DATA

Minimum covariance coverage value   0.100

     Number of missing data patterns            8


     PROPORTION OF DATA PRESENT


           Covariance Coverage
              BASTTDC       BASHKR        BASSSSC       SAC           TSBASE
              ________      ________      ________      ________      ________
 BASTTDC        0.944
 BASHKR         0.912         0.968
 BASSSSC        0.935         0.968         0.991
 SAC            0.928         0.952         0.974         0.981
 TSBASE         0.944         0.968         0.991         0.981         1.000
 BASAGEC9       0.944         0.968         0.991         0.981         1.000
 SEX            0.944         0.968         0.991         0.981         1.000
 YOL            0.944         0.968         0.991         0.981         1.000


           Covariance Coverage
              BASAGEC9      SEX           YOL
              ________      ________      ________
 BASAGEC9       1.000
 SEX            1.000         1.000
 YOL            1.000         1.000         1.000


UNIVARIATE SAMPLE STATISTICS
```

```
     UNIVARIATE HIGHER-ORDER MOMENT DESCRIPTIVE STATISTICS

         Variable/         Mean/     Skewness/   Minimum/ % with                Percentiles
        Sample Size      Variance    Kurtosis    Maximum  Min/Max      20%/60%    40%/80%    Median

     BASTTDC               5.519       0.456       0.250    0.83%       2.250      4.250      5.083
             120.000      10.267      -0.614      12.667    1.67%       6.083      8.250
     BASHKR               18.519       1.282       5.330    0.89%      12.330     16.500     17.750
             112.000      48.308       2.780      49.000    0.89%      18.670     23.330
     BASSSSC               1.333       0.640       0.000   53.33%       0.000      0.000      0.000
             120.000       2.606      -1.294       4.000   17.50%       1.000      3.000
     SAC                   3.990      -0.735       1.889    0.30%       3.556      3.875      4.000
             669.000       0.362       0.733       5.000    2.39%       4.222      4.500
     TSBASE               36.711       0.258       0.000    3.08%      13.000     27.000     36.000
             682.000     594.554      -0.917      91.000    0.59%      41.000     60.000
     BASAGEC90             0.106       0.407      -3.917    0.81%      -2.833     -1.250     -0.792
             124.000       8.313      -1.090       6.417    0.81%       0.500      3.083
     SEX                   0.000      -1.426      -0.790   20.97%      -0.790      0.210      0.210
             124.000       0.166       0.035       0.210   79.03%       0.210      0.210
     YOL                   0.000       0.868      -3.387   20.97%      -3.387     -1.387     -1.387
             124.000       8.737      -0.315       5.613   17.74%       0.613      0.613


THE MODEL ESTIMATION TERMINATED NORMALLY

     USE THE FBITERATIONS OPTION TO INCREASE THE NUMBER OF ITERATIONS BY A FACTOR
     OF AT LEAST TWO TO CHECK CONVERGENCE AND THAT THE PSR VALUE DOES NOT INCREASE.



MODEL FIT INFORMATION

Number of Free Parameters                              24

Information Criteria

          Deviance (DIC)                         2598.388
          Estimated Number of Parameters (pD)     259.872



MODEL RESULTS

                                Posterior  One-Tailed         95% C.I.
                    Estimate       S.D.      P-Value   Lower 2.5%  Upper 2.5%  Significance

Within Level

Between Level

 PHYSCON  BY
    BASHKR             1.000       0.000      0.000       1.000       1.000
    BASSSSC            0.426       0.531      0.000       0.160       1.743      *

 BASTTDC    ON
    PHYSCON            0.690       0.742      0.000       0.295       2.622      *
    SLOPESAC           4.965      17.908      0.390     -30.545      39.577
    LNVSAC             0.732       1.022      0.222      -1.253       2.794

 BASTTDC    ON
    SAC                0.473       0.772      0.262      -1.047       2.021
    SEX                1.663       0.747      0.011       0.237       3.176      *
    YOL               -0.256       0.094      0.003      -0.440      -0.072      *
    BASAGEC90         -0.226       0.096      0.010      -0.415      -0.038      *

 SAC      WITH
    SLOPESAC          -0.004       0.002      0.014      -0.008       0.000      *
    LNVSAC            -0.100       0.064      0.051      -0.234       0.021

 SLOPESAC WITH
    LNVSAC             0.000       0.003      0.460      -0.006       0.005

 Means
```

```
    SAC                4.074       0.058      0.000       3.959       4.185      *
    SLOPESAC          -0.004       0.003      0.096      -0.011       0.002
    LNVSAC            -2.259       0.116      0.000      -2.495      -2.036      *

 Intercepts
    BASTTDC            5.425       2.814      0.026      -0.085      11.036
    BASHKR            18.478       0.671      0.000      17.165      19.787      *
    BASSSSC            1.329       0.151      0.000       1.033       1.625      *

 Variances
    SAC                0.286       0.058      0.000       0.191       0.420      *
    PHYSCON            6.321       5.647      0.000       0.466      21.405      *
    SLOPESAC           0.001       0.000      0.000       0.000       0.001      *
    LNVSAC             0.358       0.140      0.000       0.158       0.705      *

 Residual Variances
    BASTTDC            6.361       1.754      0.000       2.600       9.604      *
    BASHKR            42.926       7.456      0.000      29.449      58.760      *
    BASSSSC            1.555       0.611      0.000       0.197       2.548      *


STANDARDIZED MODEL RESULTS


STDYX Standardization

                                Posterior  One-Tailed         95% C.I.
                    Estimate       S.D.      P-Value   Lower 2.5%  Upper 2.5%  Significance

Within-Level Standardized Estimates Averaged Over Clusters

 SLOPESAC | SAC ON
    TSBASE            -0.087       0.025      0.000      -0.137      -0.041      *

 LNVSAC |
    SAC                0.839       0.016      0.000       0.808       0.869      *

Between Level

 PHYSCON  BY
    BASHKR             0.356       0.137      0.000       0.098       0.625      *
    BASSSSC            0.650       0.164      0.000       0.345       0.963      *

 BASTTDC    ON
    PHYSCON            0.522       0.136      0.000       0.269       0.801      *
    SLOPESAC           0.036       0.128      0.390      -0.215       0.286
    LNVSAC             0.133       0.172      0.222      -0.218       0.456

 BASTTDC    ON
    SAC                0.076       0.122      0.262      -0.165       0.318
    SEX                0.146       0.063      0.011       0.021       0.268      *
    YOL               -0.163       0.059      0.003      -0.275      -0.046      *
    BASAGEC90         -0.131       0.055      0.010      -0.236      -0.022      *

 SAC      WITH
    SLOPESAC          -0.301       0.128      0.014      -0.530      -0.032      *
    LNVSAC            -0.323       0.180      0.051      -0.634       0.065

 SLOPESAC WITH
    LNVSAC            -0.018       0.173      0.460      -0.349       0.320

 Means
    SAC                7.613       0.792      0.000       6.252       9.354      *
    SLOPESAC          -0.175       0.135      0.096      -0.442       0.089
    LNVSAC            -3.784       0.770      0.000      -5.661      -2.664      *

 Intercepts
    BASTTDC            1.638       0.843      0.026      -0.025       3.296
    BASHKR             2.610       0.201      0.000       2.225       3.007      *
    BASSSSC            0.807       0.105      0.000       0.602       1.014      *

 Variances
    SAC                1.000       0.000      0.000       1.000       1.000
    PHYSCON            1.000       0.000      0.000       1.000       1.000
```

```
    SLOPESAC           1.000       0.000       0.000       1.000       1.000
    LNVSAC             1.000       0.000       0.000       1.000       1.000

 Residual Variances
    BASTTDC            0.583       0.149       0.000       0.233       0.812      *
    BASHKR             0.873       0.104       0.000       0.609       0.990      *
    BASSSSC            0.578       0.217       0.000       0.072       0.881      *


STDY Standardization

                                Posterior  One-Tailed         95% C.I.
                    Estimate       S.D.      P-Value   Lower 2.5%  Upper 2.5%  Significance

Within-Level Standardized Estimates Averaged Over Clusters

 SLOPESAC | SAC ON
    TSBASE            -0.011       0.008       0.073      -0.028       0.004

 LNVSAC |
    SAC                0.839       0.016       0.000       0.808       0.869      *

Between Level

 PHYSCON  BY
    BASHKR             0.356       0.137       0.000       0.098       0.625      *
    BASSSSC            0.650       0.164       0.000       0.345       0.963      *

 BASTTDC    ON
    PHYSCON            0.522       0.136       0.000       0.269       0.801      *
    SLOPESAC           0.036       0.128       0.390      -0.215       0.286
    LNVSAC             0.133       0.172       0.222      -0.218       0.456

 BASTTDC    ON
    SAC                0.076       0.122       0.262      -0.165       0.318
    SEX                0.501       0.216       0.011       0.072       0.919      *
    YOL               -0.077       0.028       0.003      -0.130      -0.022      *
    BASAGEC90         -0.068       0.029       0.010      -0.123      -0.011      *

 SAC      WITH
    SLOPESAC          -0.301       0.128       0.014      -0.530      -0.032      *
    LNVSAC            -0.323       0.180       0.051      -0.634       0.065

 SLOPESAC WITH
    LNVSAC            -0.018       0.173       0.460      -0.349       0.320

 Means
    SAC                7.613       0.792       0.000       6.252       9.354      *
    SLOPESAC          -0.175       0.135       0.096      -0.442       0.089
    LNVSAC            -3.784       0.770       0.000      -5.661      -2.664      *

 Intercepts
    BASTTDC            1.638       0.843       0.026      -0.025       3.296
    BASHKR             2.610       0.201       0.000       2.225       3.007      *
    BASSSSC            0.807       0.105       0.000       0.602       1.014      *

 Variances
    SAC                1.000       0.000       0.000       1.000       1.000
    PHYSCON            1.000       0.000       0.000       1.000       1.000
    SLOPESAC           1.000       0.000       0.000       1.000       1.000
    LNVSAC             1.000       0.000       0.000       1.000       1.000

 Residual Variances
    BASTTDC            0.583       0.149       0.000       0.233       0.812      *
    BASHKR             0.873       0.104       0.000       0.609       0.990      *
    BASSSSC            0.578       0.217       0.000       0.072       0.881      *


STD Standardization

                                Posterior  One-Tailed         95% C.I.
                    Estimate       S.D.      P-Value   Lower 2.5%  Upper 2.5%  Significance

Within-Level Standardized Estimates Averaged Over Clusters
```

```
 SLOPESAC | SAC ON
    TSBASE            -0.004       0.003      0.043      -0.009       0.001

 LNVSAC |
    SAC                0.118       0.013      0.000       0.097       0.150      *

Between Level

 PHYSCON  BY
    BASHKR             2.514       1.018      0.000       0.683       4.627      *
    BASSSSC            1.073       0.287      0.000       0.552       1.636      *

 BASTTDC    ON
    PHYSCON            1.734       0.489      0.000       0.873       2.770      *
    SLOPESAC           0.119       0.430      0.390      -0.733       0.956
    LNVSAC             0.442       0.578      0.222      -0.725       1.549

 BASTTDC    ON
    SAC                0.473       0.772      0.262      -1.047       2.021
    SEX                1.663       0.747      0.011       0.237       3.176      *
    YOL               -0.256       0.094      0.003      -0.440      -0.072      *
    BASAGEC90         -0.226       0.096      0.010      -0.415      -0.038      *

 SAC      WITH
    SLOPESAC          -0.160       0.077      0.014      -0.317      -0.016      *
    LNVSAC            -0.170       0.099      0.051      -0.352       0.035

 SLOPESAC WITH
    LNVSAC            -0.018       0.173      0.460      -0.349       0.320

 Means
    SAC                4.074       0.058      0.000       3.959       4.185      *
    SLOPESAC          -0.175       0.135      0.096      -0.442       0.089
    LNVSAC            -3.784       0.770      0.000      -5.661      -2.664      *

 Intercepts
    BASTTDC            5.425       2.814      0.026      -0.085      11.036
    BASHKR            18.478       0.671      0.000      17.165      19.787      *
    BASSSSC            1.329       0.151      0.000       1.033       1.625      *

 Variances
    SAC                0.286       0.058      0.000       0.191       0.420      *
    PHYSCON            1.000       0.000      0.000       1.000       1.000
    SLOPESAC           1.000       0.000      0.000       1.000       1.000
    LNVSAC             1.000       0.000      0.000       1.000       1.000

 Residual Variances
    BASTTDC            6.361       1.754      0.000       2.600       9.604      *
    BASHKR            42.926       7.456      0.000      29.449      58.760      *
    BASSSSC            1.555       0.611      0.000       0.197       2.548      *


R-SQUARE

Within-Level R-Square Averaged Across Clusters

                                Posterior  One-Tailed         95% C.I.
    Variable        Estimate       S.D.      P-Value   Lower 2.5%  Upper 2.5%

    SAC                0.161       0.016      0.000       0.131       0.192

Between Level

                                Posterior  One-Tailed         95% C.I.
    Variable        Estimate       S.D.      P-Value   Lower 2.5%  Upper 2.5%

    BASTTDC            0.417       0.149      0.000       0.188       0.767
    BASHKR             0.127       0.104      0.000       0.010       0.391
    BASSSSC            0.422       0.217      0.000       0.119       0.928
```

```
TECHNICAL 8 OUTPUT


   TECHNICAL 8 OUTPUT FOR BAYES ESTIMATION

     CHAIN    BSEED
     1        0
     2        285380

                     POTENTIAL       PARAMETER WITH
     ITERATION    SCALE REDUCTION      HIGHEST PSR
     100              4.390               3
     200              3.596               3
     300              2.652               3
     400              1.600               7
     500              1.370               17
     600              1.214               12
     700              1.063               23
     800              1.030               15
     900              1.022               15
     1000             1.083               17
     1100             1.175               3
     1200             1.114               3
     1300             1.136               7
     1400             1.150               7
     1500             1.151               7
     1600             1.122               7
     1700             1.124               5
     1800             1.144               5
     1900             1.165               7
     2000             1.199               7
     2100             1.196               7
     2200             1.187               7
     2300             1.278               7
     2400             1.318               7
     2500             1.307               7
     2600             1.235               7
     2700             1.163               3
     2800             1.200               3
     2900             1.183               3
     3000             1.160               3
     3100             1.105               17
     3200             1.068               17
     3300             1.043               17
     3400             1.051               17
     3500             1.064               17
     3600             1.084               17
     3700             1.094               17
     3800             1.096               17
     3900             1.105               20
     4000             1.103               20
     4100             1.106               20
     4200             1.097               20
     4300             1.101               20
     4400             1.101               20
     4500             1.135               20
     4600             1.149               20
     4700             1.137               20
     4800             1.129               20
     4900             1.134               20
     5000             1.122               20
     5100             1.106               20
     5200             1.120               20
     5300             1.092               20
     5400             1.078               20
     5500             1.060               20
     5600             1.048               20
     5700             1.041               20
     5800             1.046               20
     5900             1.038               20
     6000             1.019               20
     6100             1.014               3
     6200             1.010               3
     6300             1.007               3
```

6400	1.006	10
6500	1.005	10
6600	1.004	7
6700	1.005	7
6800	1.005	3
6900	1.016	3
7000	1.033	3
7100	1.050	3
7200	1.058	3
7300	1.073	3
7400	1.085	3
7500	1.098	3
7600	1.106	3
7700	1.101	3
7800	1.095	3
7900	1.090	3
8000	1.079	3
8100	1.069	3
8200	1.062	3
8300	1.062	3
8400	1.064	3
8500	1.064	3
8600	1.066	3
8700	1.066	3
8800	1.070	3
8900	1.075	3
9000	1.079	3
9100	1.078	3
9200	1.085	3
9300	1.093	3
9400	1.102	3
9500	1.097	3
9600	1.094	3
9700	1.089	3
9800	1.088	3
9900	1.087	3
10000	1.090	3
10100	1.096	3
10200	1.102	3
10300	1.110	3
10400	1.113	3
10500	1.106	3
10600	1.113	3
10700	1.115	3
10800	1.121	3
10900	1.120	3
11000	1.106	3
11100	1.097	3
11200	1.095	3
11300	1.090	3
11400	1.087	3
11500	1.087	3
11600	1.087	3
11700	1.088	3
11800	1.087	3
11900	1.083	3
12000	1.077	3
12100	1.076	3
12200	1.072	3
12300	1.069	3
12400	1.066	3
12500	1.070	3
12600	1.074	3
12700	1.077	3
12800	1.080	3
12900	1.081	3
13000	1.079	3
13100	1.079	3
13200	1.080	17
13300	1.087	17
13400	1.088	17
13500	1.083	17
13600	1.079	17
13700	1.073	3

13800	1.067	3
13900	1.062	3
14000	1.057	3
14100	1.053	3
14200	1.049	17
14300	1.047	3
14400	1.044	3
14500	1.040	3
14600	1.037	3
14700	1.035	3
14800	1.035	3
14900	1.036	3
15000	1.035	17
15100	1.040	17
15200	1.042	17
15300	1.045	17
15400	1.047	17
15500	1.048	17
15600	1.045	17
15700	1.043	17
15800	1.039	17
15900	1.042	17
16000	1.045	17
16100	1.047	17
16200	1.049	17
16300	1.051	17
16400	1.051	17
16500	1.051	17
16600	1.050	17
16700	1.049	17
16800	1.047	17
16900	1.046	17
17000	1.042	17
17100	1.037	17
17200	1.038	17
17300	1.039	17
17400	1.040	17
17500	1.040	17
17600	1.037	17
17700	1.034	17
17800	1.030	17
17900	1.030	3
18000	1.030	3
18100	1.029	3
18200	1.029	3
18300	1.029	3
18400	1.031	3
18500	1.036	3
18600	1.036	3
18700	1.040	3
18800	1.041	10
18900	1.046	10
19000	1.049	10
19100	1.049	10
19200	1.051	3
19300	1.053	3
19400	1.053	3
19500	1.054	3
19600	1.054	3
19700	1.053	3
19800	1.053	3
19900	1.053	3
20000	1.052	3
20100	1.052	3
20200	1.052	3
20300	1.051	3
20400	1.051	3
20500	1.051	3
20600	1.050	3
20700	1.050	3
20800	1.049	3
20900	1.048	3
21000	1.047	3
21100	1.047	3

```
     21200              1.046              3
     21300              1.045              3
     21400              1.045              3
     21500              1.044              3
     21600              1.044              3
     21700              1.044              3
     21800              1.043              3
     21900              1.043              3
     22000              1.043              3
     22100              1.043              3
     22200              1.045              3
     22300              1.045              3
     22400              1.047              3
     22500              1.049              3
     22600              1.050              3
     22700              1.050              3
     22800              1.049              3
     22900              1.049              3
     23000              1.049              3
     23100              1.048              3
     23200              1.048              3
     23300              1.048              3
     23400              1.048              3
     23500              1.048              3
     23600              1.047              3
     23700              1.046              3
     23800              1.046              3
     23900              1.046              3
     24000              1.046              3
     24100              1.046              3
     24200              1.045              3
     24300              1.045              3
     24400              1.045              3
     24500              1.045              3
     24600              1.045              3
     24700              1.045              3
     24800              1.045              3
     24900              1.044              3
     25000              1.044              3
     25100              1.043              3
     25200              1.043              3
     25300              1.042              3
     25400              1.042              3
     25500              1.041              3
     25600              1.041              3
     25700              1.041              3
     25800              1.040              3
     25900              1.040              3
     26000              1.040              3
     26100              1.040              3
     26200              1.040              3
     26300              1.040              3
     26400              1.039              3
     26500              1.039              3
     26600              1.038              3
     26700              1.038              3
     26800              1.038              3
     26900              1.038              3
     27000              1.037              3
     27100              1.037              3
     27200              1.037              3
     27300              1.037              3
     27400              1.037              3
     27500              1.037              3
     27600              1.036              3
     27700              1.036              3
     27800              1.036              3
     27900              1.036              3
     28000              1.036              3
     28100              1.036              3
     28200              1.036              3
     28300              1.036              3
     28400              1.036              3
     28500              1.036              3
```

```
     28600          1.036          3
     28700          1.036          3
     28800          1.036          3
     28900          1.036          3
     29000          1.036          3
     29100          1.036          3
     29200          1.036          3
     29300          1.035          3
     29400          1.035          3
     29500          1.035          3
     29600          1.035          3
     29700          1.035          3
     29800          1.035          3
     29900          1.035          3
     30000          1.035          3
     30100          1.035          3
     30200          1.034          3
     30300          1.034          3
     30400          1.034          3
     30500          1.034          3
     30600          1.034          3
     30700          1.034          3
     30800          1.034          3
     30900          1.034          3
     31000          1.034          3
     31100          1.034          3
     31200          1.034          3
     31300          1.034          3
     31400          1.034          3
     31500          1.035          3
     31600          1.035          3
     31700          1.035          3
     31800          1.035          3
     31900          1.035          3
     32000          1.035          3
     32100          1.034          3
     32200          1.034          3
     32300          1.033          3
     32400          1.032          3
     32500          1.031          3
     32600          1.030          3
     32700          1.029          3
     32800          1.027          3
     32900          1.026          3
     33000          1.025          3
     33100          1.024          3
     33200          1.023          3
     33300          1.023          3
     33400          1.023          3
     33500          1.022          3
     33600          1.023          3
     33700          1.023          3
     33800          1.023          3
     33900          1.023          3
     34000          1.022          3
     34100          1.022          3
     34200          1.022          3
     34300          1.022          3
     34400          1.022          3
     34500          1.023          3
     34600          1.022          3
     34700          1.022          3
     34800          1.022          3
     34900          1.022          3
     35000          1.022          3
     35100          1.022          3
     35200          1.022          3
     35300          1.021          3
     35400          1.022          3
     35500          1.022          3
     35600          1.021          3
     35700          1.021          3
     35800          1.021          3
     35900          1.021          3
```

36000	1.021	3
36100	1.021	3
36200	1.021	3
36300	1.021	3
36400	1.021	3
36500	1.021	3
36600	1.021	3
36700	1.020	3
36800	1.020	3
36900	1.019	3
37000	1.018	3
37100	1.017	3
37200	1.016	3
37300	1.014	3
37400	1.013	3
37500	1.012	3
37600	1.014	3
37700	1.018	3
37800	1.022	3
37900	1.023	3
38000	1.021	3
38100	1.020	3
38200	1.018	3
38300	1.017	3
38400	1.015	3
38500	1.013	7
38600	1.013	7
38700	1.014	7
38800	1.014	7
38900	1.014	7
39000	1.013	7
39100	1.014	7
39200	1.015	7
39300	1.015	7
39400	1.015	7
39500	1.014	7
39600	1.013	7
39700	1.013	7
39800	1.012	7
39900	1.011	7
40000	1.011	7
40100	1.011	7
40200	1.011	7
40300	1.012	7
40400	1.013	7
40500	1.013	7
40600	1.014	7
40700	1.014	7
40800	1.014	7
40900	1.014	7
41000	1.014	7
41100	1.014	7
41200	1.012	7
41300	1.011	7
41400	1.012	7
41500	1.012	7
41600	1.013	7
41700	1.013	7
41800	1.013	7
41900	1.014	7
42000	1.014	7
42100	1.014	7
42200	1.014	7
42300	1.013	7
42400	1.012	7
42500	1.013	7
42600	1.013	7
42700	1.012	7
42800	1.013	7
42900	1.013	7
43000	1.013	7
43100	1.012	7
43200	1.012	7
43300	1.011	7

43400	1.011	7
43500	1.012	7
43600	1.012	7
43700	1.012	7
43800	1.013	7
43900	1.014	7
44000	1.015	7
44100	1.014	7
44200	1.012	7
44300	1.011	7
44400	1.011	7
44500	1.011	7
44600	1.012	7
44700	1.011	7
44800	1.011	7
44900	1.011	7
45000	1.012	7
45100	1.011	7
45200	1.011	7
45300	1.012	7
45400	1.011	7
45500	1.011	7
45600	1.012	7
45700	1.011	7
45800	1.011	7
45900	1.011	7
46000	1.011	7
46100	1.010	7
46200	1.010	7
46300	1.010	7
46400	1.010	7
46500	1.009	7
46600	1.009	7
46700	1.008	7
46800	1.008	7
46900	1.008	7
47000	1.009	7
47100	1.010	7
47200	1.010	7
47300	1.010	7
47400	1.011	7
47500	1.012	7
47600	1.013	7
47700	1.014	7
47800	1.015	7
47900	1.015	7
48000	1.015	7
48100	1.016	7
48200	1.018	7
48300	1.020	7
48400	1.020	7
48500	1.020	7
48600	1.019	7
48700	1.018	7
48800	1.019	7
48900	1.019	7
49000	1.018	7
49100	1.018	7
49200	1.018	7
49300	1.017	7
49400	1.018	7
49500	1.017	7
49600	1.017	7
49700	1.018	7
49800	1.018	7
49900	1.019	7
50000	1.019	7
50100	1.019	7
50200	1.019	7
50300	1.019	7
50400	1.019	7
50500	1.018	7
50600	1.017	7
50700	1.016	7

```
     50800            1.017            7
     50900            1.017            7
     51000            1.018            7
     51100            1.018            7
     51200            1.018            7
     51300            1.018            7
     51400            1.017            7
     51500            1.018            7
     51600            1.018            7
     51700            1.018            7
     51800            1.018            7
     51900            1.019            7
     52000            1.018            7
     52100            1.018            7
     52200            1.017            7
     52300            1.016            7
     52400            1.015            7
     52500            1.015            7
     52600            1.016            7
     52700            1.016            7
     52800            1.016            7
     52900            1.016            7
     53000            1.016            7
     53100            1.017            7
     53200            1.017            7
     53300            1.017            7
     53400            1.017            7
     53500            1.017            7
     53600            1.017            7
     53700            1.018            7
     53800            1.019            7
     53900            1.019            7
     54000            1.018            7
     54100            1.017            7
     54200            1.017            7
     54300            1.016            7
     54400            1.016            7
     54500            1.017            7
     54600            1.018            7
     54700            1.018            7
     54800            1.017            7
     54900            1.017            7
     55000            1.017            7
     55100            1.017            7
     55200            1.017            7
     55300            1.017            7
     55400            1.018            7
     55500            1.017            7
     55600            1.017            7
     55700            1.017            7
     55800            1.017            7
     55900            1.017            7
     56000            1.017            7
     56100            1.017            7
     56200            1.016            7
     56300            1.016            7
     56400            1.015            7
     56500            1.015            7
     56600            1.015            7
     56700            1.015            7
     56800            1.015            7
     56900            1.014            7
     57000            1.014            7
     57100            1.013            7
     57200            1.013            7
     57300            1.013            7
     57400            1.013            7
     57500            1.014            7
     57600            1.013            7
     57700            1.012            7
     57800            1.012            7
     57900            1.012            7
     58000            1.012            7
     58100            1.012            7
```

```
58200           1.012           7
58300           1.011           7
58400           1.012           7
58500           1.012           7
58600           1.011           7
58700           1.011           7
58800           1.011           7
58900           1.011           7
59000           1.011           7
59100           1.011           7
59200           1.011           7
59300           1.012           7
59400           1.012           7
59500           1.013           7
59600           1.013           7
59700           1.014           7
59800           1.015           7
59900           1.016           7
60000           1.016           7
60100           1.016           7
60200           1.015           7
60300           1.015           7
60400           1.015           7
60500           1.016           7
60600           1.015           7
60700           1.015           7
60800           1.015           7
60900           1.015           7
61000           1.015           7
61100           1.015           7
61200           1.015           7
61300           1.015           7
61400           1.015           7
61500           1.015           7
61600           1.014           7
61700           1.014           7
61800           1.013           7
61900           1.013           7
62000           1.012           7
62100           1.013           7
62200           1.012           7
62300           1.012           7
62400           1.012           7
62500           1.012           7
62600           1.012           7
62700           1.012           7
62800           1.011           7
62900           1.011           7
63000           1.011           7
63100           1.011           7
63200           1.011           7
63300           1.011           7
63400           1.012           7
63500           1.012           7
63600           1.011           7
63700           1.011           7
63800           1.012           7
63900           1.011           7
64000           1.011           7
64100           1.011           7
64200           1.011           7
64300           1.011           7
64400           1.011           7
64500           1.012           7
64600           1.012           7
64700           1.012           7
64800           1.011           7
64900           1.011           7
65000           1.011           7
65100           1.011           7
65200           1.010           7
65300           1.009           7
65400           1.009           7
65500           1.009           7
```

```
65600            1.009            7
65700            1.009            7
65800            1.009            7
65900            1.009            7
66000            1.010            7
66100            1.009            7
66200            1.008            7
66300            1.008            7
66400            1.007            7
66500            1.007            7
66600            1.007            7
66700            1.007            7
66800            1.007            7
66900            1.008            7
67000            1.008            7
67100            1.008            7
67200            1.008            7
67300            1.008            7
67400            1.008            7
67500            1.007            7
67600            1.007            7
67700            1.006            7
67800            1.006            7
67900            1.007            7
68000            1.007            7
68100            1.008            7
68200            1.007            7
68300            1.008            7
68400            1.007            7
68500            1.007            7
68600            1.007            7
68700            1.006            7
68800            1.006            7
68900            1.006            7
69000            1.005            7
69100            1.005            7
69200            1.005            7
69300            1.005            7
69400            1.004            7
69500            1.004            7
69600            1.004            7
69700            1.004            7
69800            1.004            7
69900            1.004            3
70000            1.004            3
70100            1.004            3
70200            1.004            3
70300            1.004            3
70400            1.004            3
70500            1.004            3
70600            1.004            3
70700            1.004            7
70800            1.004            7
70900            1.004            7
71000            1.004            7
71100            1.004            7
71200            1.004            7
71300            1.004            7
71400            1.004            7
71500            1.004            7
71600            1.004            7
71700            1.003            3
71800            1.004            3
71900            1.004            3
72000            1.005            3
72100            1.005            3
72200            1.005            3
72300            1.005            3
72400            1.005            3
72500            1.004            3
72600            1.004            3
72700            1.004            3
72800            1.003            3
72900            1.003            7
```

```
     73000            1.003             7
     73100            1.004             7
     73200            1.004             7
     73300            1.004             7
     73400            1.004             7
     73500            1.004             7
     73600            1.004             7
     73700            1.005             7
     73800            1.005             7
     73900            1.004             7
     74000            1.004             7
     74100            1.004             7
     74200            1.004             7
     74300            1.004             7
     74400            1.003             7
     74500            1.003             7
     74600            1.003             7
     74700            1.003             7
     74800            1.003             7
     74900            1.003             7
     75000            1.003             7
     75100            1.003             7
     75200            1.003             7
     75300            1.003             7
     75400            1.003             7
     75500            1.003             7
     75600            1.003             7
     75700            1.003             7
     75800            1.003             7
     75900            1.003             7
     76000            1.003             7
     76100            1.002             7
     76200            1.002             7
     76300            1.003             3
     76400            1.003             3
     76500            1.004             3
     76600            1.004             3
     76700            1.004             3
     76800            1.004             3
     76900            1.004             3
     77000            1.004             3
     77100            1.004             3
     77200            1.004             3
     77300            1.004             3
     77400            1.005             3
     77500            1.005             3
     77600            1.005             3
     77700            1.006             3
     77800            1.006             3
     77900            1.006             3
     78000            1.006             3
     78100            1.006             3
     78200            1.006             3
     78300            1.006             3
     78400            1.006             3
     78500            1.006             3
     78600            1.006             3
     78700            1.005             3
     78800            1.005             3
     78900            1.005             10
     79000            1.005             10
     79100            1.004             3
     79200            1.004             10
     79300            1.004             10
     79400            1.004             10
     79500            1.004             3
     79600            1.004             3
     79700            1.004             10
     79800            1.004             10
     79900            1.004             3
     80000            1.004             3
     80100            1.004             3
     80200            1.004             3
     80300            1.004             3
```

80400	1.004	3
80500	1.004	3
80600	1.004	10
80700	1.004	10
80800	1.004	10
80900	1.004	10
81000	1.003	10
81100	1.003	3
81200	1.003	3
81300	1.003	10
81400	1.003	10
81500	1.003	10
81600	1.003	10
81700	1.003	10
81800	1.003	10
81900	1.003	10
82000	1.003	10
82100	1.003	10
82200	1.003	10
82300	1.002	10
82400	1.003	3
82500	1.003	3
82600	1.003	3
82700	1.003	3
82800	1.004	3
82900	1.004	3
83000	1.004	3
83100	1.004	3
83200	1.004	3
83300	1.004	3
83400	1.004	3
83500	1.004	3
83600	1.004	3
83700	1.004	3
83800	1.004	10
83900	1.004	10
84000	1.004	10
84100	1.004	10
84200	1.004	10
84300	1.005	10
84400	1.005	10
84500	1.005	3
84600	1.006	3
84700	1.006	3
84800	1.006	3
84900	1.006	3
85000	1.005	3
85100	1.005	3
85200	1.006	3
85300	1.006	3
85400	1.006	3
85500	1.006	3
85600	1.005	3
85700	1.005	3
85800	1.004	3
85900	1.004	3
86000	1.004	10
86100	1.004	10
86200	1.004	10
86300	1.004	10
86400	1.004	3
86500	1.004	3
86600	1.005	3
86700	1.005	3
86800	1.006	10
86900	1.006	3
87000	1.007	3
87100	1.008	10
87200	1.009	10
87300	1.010	10
87400	1.011	10
87500	1.012	10
87600	1.012	3
87700	1.012	3

```
87800             1.013              3
87900             1.013              3
88000             1.013              3
88100             1.013              3
88200             1.013              3
88300             1.013              3
88400             1.013              3
88500             1.013              3
88600             1.013              3
88700             1.013              3
88800             1.013              3
88900             1.013              3
89000             1.013              3
89100             1.014              3
89200             1.014              3
89300             1.014              3
89400             1.014              3
89500             1.014              3
89600             1.014              3
89700             1.014              3
89800             1.014              3
89900             1.014              3
90000             1.015              3
90100             1.016              3
90200             1.016              3
90300             1.017              3
90400             1.017              3
90500             1.016              3
90600             1.016              3
90700             1.016              3
90800             1.016              3
90900             1.016              3
91000             1.016              3
91100             1.016              3
91200             1.016              3
91300             1.016              3
91400             1.016              3
91500             1.016              3
91600             1.016              3
91700             1.017              3
91800             1.017              3
91900             1.017              3
92000             1.017              3
92100             1.017              3
92200             1.017              3
92300             1.017              3
92400             1.017              3
92500             1.017              3
92600             1.017              3
92700             1.017              3
92800             1.017              3
92900             1.017              3
93000             1.017              3
93100             1.017              3
93200             1.017              3
93300             1.017              3
93400             1.017              3
93500             1.016              3
93600             1.016              3
93700             1.016              3
93800             1.016              3
93900             1.016              3
94000             1.016              3
94100             1.016              3
94200             1.016              3
94300             1.016              3
94400             1.016              3
94500             1.016              3
94600             1.016              3
94700             1.015              3
94800             1.015              3
94900             1.015              3
95000             1.015              3
95100             1.015              3
```

95200	1.015	3
95300	1.015	3
95400	1.015	3
95500	1.015	3
95600	1.015	3
95700	1.014	3
95800	1.014	3
95900	1.014	3
96000	1.014	3
96100	1.014	3
96200	1.014	3
96300	1.014	3
96400	1.014	3
96500	1.014	3
96600	1.014	3
96700	1.014	3
96800	1.015	3
96900	1.015	3
97000	1.015	3
97100	1.015	3
97200	1.015	3
97300	1.014	3
97400	1.015	3
97500	1.015	3
97600	1.015	3
97700	1.015	3
97800	1.016	3
97900	1.017	3
98000	1.017	3
98100	1.018	3
98200	1.018	3
98300	1.018	3
98400	1.018	3
98500	1.017	3
98600	1.017	3
98700	1.017	3
98800	1.016	3
98900	1.016	3
99000	1.017	3
99100	1.018	3
99200	1.018	3
99300	1.018	3
99400	1.018	3
99500	1.018	3
99600	1.018	3
99700	1.019	3
99800	1.019	3
99900	1.019	3
100000	1.019	3
100100	1.019	3
100200	1.019	3
100300	1.018	3
100400	1.018	3
100500	1.019	3
100600	1.019	3
100700	1.018	3
100800	1.018	3
100900	1.018	3
101000	1.018	3
101100	1.018	3
101200	1.018	3
101300	1.019	3
101400	1.019	3
101500	1.019	3
101600	1.019	3
101700	1.019	3
101800	1.019	3
101900	1.018	3
102000	1.018	3
102100	1.018	3
102200	1.018	3
102300	1.018	3
102400	1.018	3
102500	1.017	3

```
102600          1.017              3
102700          1.016              3
102800          1.016              3
102900          1.015              3
103000          1.015              3
103100          1.015              3
103200          1.015              3
103300          1.015              3
103400          1.015              3
103500          1.015              3
103600          1.015              3
103700          1.014              3
103800          1.014              3
103900          1.014              3
104000          1.014              3
104100          1.014              3
104200          1.014              3
104300          1.014              3
104400          1.014              3
104500          1.014              3
104600          1.014              3
104700          1.014              3
104800          1.014              3
104900          1.014              3
105000          1.014              3
105100          1.014              3
105200          1.014              3
105300          1.014              3
105400          1.014              3
105500          1.014              3
105600          1.013              3
105700          1.013              3
105800          1.013              3
105900          1.013              3
106000          1.013              3
106100          1.012              3
106200          1.011              3
106300          1.011              3
106400          1.011              3
106500          1.011              3
106600          1.010              3
106700          1.010              3
106800          1.010              3
106900          1.010              3
107000          1.010              3
107100          1.010              3
107200          1.010              3
107300          1.010              3
107400          1.009              3
107500          1.009              3
107600          1.009              3
107700          1.009              3
107800          1.009              3
107900          1.009              3
108000          1.009              3
108100          1.009              3
108200          1.009              3
108300          1.009              3
108400          1.009              3
108500          1.009              3
108600          1.009              3
108700          1.009              3
108800          1.009              3
108900          1.009              3
109000          1.009              3
109100          1.008              3
109200          1.008              3
109300          1.008              3
109400          1.008              3
109500          1.009              3
109600          1.009              3
109700          1.009              3
109800          1.009              3
109900          1.009              3
```

```
110000          1.009          3
110100          1.009          3
110200          1.009          3
110300          1.009          3
110400          1.009          3
110500          1.009          3
110600          1.009          3
110700          1.009          3
110800          1.009          3
110900          1.009          3
111000          1.009          3
111100          1.009          3
111200          1.009          3
111300          1.009          3
111400          1.009          3
111500          1.009          3
111600          1.009          3
111700          1.009          3
111800          1.009          3
111900          1.009          3
112000          1.009          3
112100          1.009          3
112200          1.009          10
112300          1.009          10
112400          1.009          10
112500          1.009          10
112600          1.009          10
112700          1.009          10
112800          1.009          10
112900          1.009          10
113000          1.009          10
113100          1.009          10
113200          1.010          10
113300          1.010          10
113400          1.010          10
113500          1.010          10
113600          1.010          10
113700          1.010          10
113800          1.010          10
113900          1.010          10
114000          1.010          10
114100          1.010          10
114200          1.009          10
114300          1.010          10
114400          1.010          10
114500          1.010          10
114600          1.010          10
114700          1.009          10
114800          1.009          10
114900          1.009          10
115000          1.009          10
115100          1.009          10
115200          1.009          10
115300          1.009          10
115400          1.009          10
115500          1.009          10
115600          1.009          10
115700          1.010          10
115800          1.009          10
115900          1.009          10
116000          1.009          10
116100          1.009          10
116200          1.009          10
116300          1.009          10
116400          1.009          10
116500          1.009          10
116600          1.009          10
116700          1.009          10
116800          1.009          10
116900          1.009          10
117000          1.009          10
117100          1.009          10
117200          1.009          10
117300          1.009          10
```

```
117400          1.009           10
117500          1.009           3
117600          1.009           3
117700          1.009           3
117800          1.008           3
117900          1.008           10
118000          1.007           10
118100          1.007           10
118200          1.007           10
118300          1.007           10
118400          1.007           10
118500          1.007           10
118600          1.007           10
118700          1.007           10
118800          1.007           10
118900          1.007           10
119000          1.007           10
119100          1.007           10
119200          1.007           10
119300          1.007           10
119400          1.007           3
119500          1.007           3
119600          1.007           3
119700          1.006           3
119800          1.006           3
119900          1.006           3
120000          1.006           3
120100          1.006           3
120200          1.006           10
120300          1.006           3
120400          1.006           3
120500          1.006           3
120600          1.006           3
120700          1.006           3
120800          1.006           3
120900          1.006           3
121000          1.005           3
121100          1.005           3
121200          1.004           10
121300          1.004           10
121400          1.004           10
121500          1.004           10
121600          1.004           10
121700          1.004           10
121800          1.004           10
121900          1.004           10
122000          1.004           10
122100          1.004           10
122200          1.003           3
122300          1.004           3
122400          1.004           3
122500          1.004           3
122600          1.004           3
122700          1.004           3
122800          1.004           3
122900          1.004           3
123000          1.004           3
123100          1.004           10
123200          1.004           10
123300          1.004           10
123400          1.004           10
123500          1.004           10
123600          1.004           10
123700          1.004           10
123800          1.004           10
123900          1.004           10
124000          1.004           10
124100          1.004           3
124200          1.004           3
124300          1.004           3
124400          1.004           3
124500          1.004           3
124600          1.004           3
124700          1.004           3
```

```
     124800          1.004               3
     124900          1.004               3
     125000          1.004               3
     125100          1.004               3
     125200          1.004               3
     125300          1.003               3
     125400          1.003               3
     125500          1.003               3
     125600          1.003               3
     125700          1.003               3
     125800          1.003               3
     125900          1.003               3
     126000          1.003               3
     126100          1.003               3
     126200          1.003               3
     126300          1.003               3
     126400          1.003               3
     126500          1.003               3
     126600          1.003               3
     126700          1.003               3
     126800          1.003               3
     126900          1.003               3
     127000          1.003               3
     127100          1.003               3
     127200          1.003               3
     127300          1.003               3
     127400          1.003               3
     127500          1.003               3
     127600          1.003               3
     127700          1.004               3
     127800          1.004               3
     127900          1.003               3
     128000          1.003               3
     128100          1.003               3
     128200          1.003               3
     128300          1.003               3
     128400          1.003               3
     128500          1.003               3
     128600          1.003               3
     128700          1.003               3
     128800          1.003               3
     128900          1.003               3
     129000          1.003               3
     129100          1.003               3
     129200          1.003               10
     129300          1.003               10
     129400          1.003               10
     129500          1.003               10
     129600          1.003               10
     129700          1.003               10
     129800          1.003               10
     129900          1.003               10
     130000          1.003               10
     130100          1.003               10
     130200          1.003               10
     130300          1.003               10
     130400          1.003               10
     130500          1.003               10
     130600          1.003               10
     130700          1.003               10
     130800          1.003               10
     130900          1.002               10
     131000          1.002               10
     131100          1.002               10
     131200          1.002               10
     131300          1.002               10
     131400          1.002               10
     131500          1.002               3
     131600          1.002               3
     131700          1.002               3
     131800          1.002               3

TECHNICAL 5 OUTPUT
```

```
DIAGRAM INFORMATION

  Mplus diagrams are currently not available for multilevel analysis.
  No diagram output was produced.


     Beginning Time:  16:16:45
        Ending Time:  16:39:21
       Elapsed Time:  00:22:36



MUTHEN & MUTHEN
3463 Stoner Ave.
Los Angeles, CA  90066

Tel: (310) 391-9971
Fax: (310) 391-8971
Web: www.StatModel.com
Support: Support@StatModel.com
```

Copyright (c) 1998-2020 Muthen & Muthen

Mplus Output Single Multilevel SEM – Life Satisfaction:

```
Mplus VERSION 8.5
MUTHEN & MUTHEN
10/12/2021   6:25 PM

INPUT INSTRUCTIONS

  TITLE:

  DATA: FILE = mpltry6.dat;

  VARIABLE:
   names =  pno wave sex yol verst nopart
         basagec90 agec90 tims tsbase
         ttd ttdcens basettd basttdc
         aut pil ema sac dep
         pa na swls adlinv hyp
         visus sfenerg sfsofunc sfpain sfgen sfchang
         anges anget akzet mortinv mortperc
         basadlinv basvisus bashkr bassssc
         bassfen bassfso bassfpain bassfgen bassfcha
         hkr sl sssco;
  usevariables = swls tsbase basttdc bashkr bassssc basagec90 sex yol;
  cluster = PNO;
  missing = .;
  within = tsbase;
  between = basttdc bashkr bassssc sex yol basagec90;

 DEFINE:
  center sex yol (grandmean);

 ANALYSIS:
  type = twolevel random;
  estimator = BAYES;
  chains = 2;
  biterations=1000000;
  BCONVERGENCE = 0.00125;

MODEL:

  %WITHIN%

  slopels | swls ON tsbase;
  lnvls | swls;

  %BETWEEN%
  physcon by bashkr@1 bassssc;

  basttdc ON swls
             sex
             yol
             physcon
             basagec90
             slopels
             lnvls;

  [ swls*3.84819 ] (m6);
  [ slopels*-0.00061 ] (ms6);
  [ lnvls*-1.94704 ] (mv6);

  basttdc*6.66980;
  swls*0.44294;
  slopels*0.00041;

  swls with slopels lnvls;
  slopels with lnvls;

 MODEL PRIORS:
  m6 ~ N(3.66,0.372);
  ms6 ~ N(0.045,0.00053);
```

```
 OUTPUT:
  standardized tech5 tech8 tech16;




*** WARNING in MODEL command
  In the MODEL command, the following variable is an x-variable on the BETWEEN
  level and a y-variable on the WITHIN level.  This variable will be treated
  as a y-variable on both levels:  SWLS
*** WARNING
  One or more individual-level variables have no variation within a
  cluster for the following clusters.

     Variable   Cluster IDs with no within-cluster variation

      SWLS        10130 10144 10313 10473 10893 11240 11297 11741 15018 16142 16798 11258 15510
                  18252 10228

   3 WARNING(S) FOUND IN THE INPUT INSTRUCTIONS




SUMMARY OF ANALYSIS

Number of groups                                                 1
Number of observations                                         682

Number of dependent variables                                    4
Number of independent variables                                  4
Number of continuous latent variables                            3

Observed dependent variables

  Continuous
   BASTTDC     BASHKR      BASSSSC     SWLS

Observed independent variables
   TSBASE      BASAGEC9    SEX         YOL

Continuous latent variables
   PHYSCON     SLOPELS     LNVLS

Variables with special functions

  Cluster variable      PNO

  Within variables
   TSBASE

  Between variables
   BASTTDC     BASHKR      BASSSSC     BASAGEC9    SEX         YOL

  Centering (GRANDMEAN)
   SEX         YOL


Estimator                                                    BAYES
Specifications for Bayesian Estimation
  Point estimate                                            MEDIAN
  Number of Markov chain Monte Carlo (MCMC) chains               2
  Random seed for the first chain                                0
  Starting value information                           UNPERTURBED
  Algorithm used for Markov chain Monte Carlo           GIBBS(PX1)
  Convergence criterion                                  0.125D-02
  Maximum number of iterations                             1000000
  K-th iteration used for thinning                               1

Input data file(s)
  mpltry6.dat
Input data format  FREE
```

```
SUMMARY OF DATA

     Number of clusters                        124

       Size (s)    Cluster ID with Size s

          1        10026 10130 10144 10313 10473 10558 10567 10637 10663
                   10893 10901 10907 10911 10939 10969 11157 11240 11294
                   11297 11741 15018 15177 15241 15319 15564 16142 16423
                   16610 16669 16798 17542 18507 18650
          2        15239 11258 10181 15510 10615 11341 16303 11371 16510
                   11384 11485 11028 16862 10211 17780 18004 18252 15175
                   10228
          3        11573 11295 17130 11100 17745 10340 10460 10461 10210
                   11501
          4        10904 11457 10434 11493 10811 17593
          5        15424 10666 12411 11343 11005
          6        10159 15092 15121 15426 11593 11163 10139 16220 19067
          7        10354 11230 10894 10724 10470
          8        10252 10528 11038 10033 11336 11253
          9        15623 11055
         10        12037 10167 17197 15009 19500
         11        11233 10444 15714 10533
         12        11079 10577 15378 12714 10986 10428
         13        11420 11002
         14        11450
         15        10108 10906 10551 15231 10902
         16        15141 10940 16103 15472 11528 11378


COVARIANCE COVERAGE OF DATA

Minimum covariance coverage value   0.100

     Number of missing data patterns             7


     PROPORTION OF DATA PRESENT


           Covariance Coverage
              BASTTDC       BASHKR        BASSSSC       SWLS          TSBASE
              ________      ________      ________      ________      ________
 BASTTDC        0.944
 BASHKR         0.912         0.968
 BASSSSC        0.935         0.968         0.991
 SWLS           0.918         0.956         0.968         0.974
 TSBASE         0.944         0.968         0.991         0.974         1.000
 BASAGEC9       0.944         0.968         0.991         0.974         1.000
 SEX            0.944         0.968         0.991         0.974         1.000
 YOL            0.944         0.968         0.991         0.974         1.000


           Covariance Coverage
              BASAGEC9      SEX           YOL
              ________      ________      ________
 BASAGEC9       1.000
 SEX            1.000         1.000
 YOL            1.000         1.000         1.000


UNIVARIATE SAMPLE STATISTICS


     UNIVARIATE HIGHER-ORDER MOMENT DESCRIPTIVE STATISTICS

         Variable/         Mean/     Skewness/   Minimum/ % with                Percentiles
        Sample Size      Variance    Kurtosis    Maximum  Min/Max      20%/60%    40%/80%    Median

     BASTTDC                5.519       0.456       0.250    0.83%       2.250      4.250      5.083
```

```
        120.000       10.267      -0.614      12.667    1.67%       6.083      8.250
     BASHKR                 18.519       1.282       5.330    0.89%      12.330     16.500     17.750
        112.000       48.308       2.780      49.000    0.89%      18.670     23.330
     BASSSSC                 1.333       0.640       0.000   53.33%       0.000      0.000      0.000
        120.000        2.606      -1.294       4.000   17.50%       1.000      3.000
     SWLS                    3.782      -0.748       1.000    0.30%       3.200      3.750      3.800
        664.000        0.591       0.556       5.000    5.72%       4.000      4.400
     TSBASE                 36.711       0.258       0.000    3.08%      13.000     27.000     36.000
        682.000      594.554      -0.917      91.000    0.59%      41.000     60.000
     BASAGEC90               0.106       0.407      -3.917    0.81%      -2.833     -1.250     -0.792
        124.000        8.313      -1.090       6.417    0.81%       0.500      3.083
     SEX                     0.000      -1.426      -0.790   20.97%      -0.790      0.210      0.210
        124.000        0.166       0.035       0.210   79.03%       0.210      0.210
     YOL                     0.000       0.868      -3.387   20.97%      -3.387     -1.387     -1.387
        124.000        8.737      -0.315       5.613   17.74%       0.613      0.613


THE MODEL ESTIMATION TERMINATED NORMALLY

     USE THE FBITERATIONS OPTION TO INCREASE THE NUMBER OF ITERATIONS BY A FACTOR
     OF AT LEAST TWO TO CHECK CONVERGENCE AND THAT THE PSR VALUE DOES NOT INCREASE.



MODEL FIT INFORMATION

Number of Free Parameters                              24

Information Criteria

          Deviance (DIC)                        2747.537
          Estimated Number of Parameters (pD)    238.686



MODEL RESULTS

                                Posterior  One-Tailed         95% C.I.
                    Estimate       S.D.      P-Value   Lower 2.5%  Upper 2.5%  Significance

Within Level

Between Level

 PHYSCON  BY
    BASHKR             1.000       0.000      0.000       1.000       1.000
    BASSSSC            0.444       0.428      0.000       0.166       1.664      *

 BASTTDC    ON
    PHYSCON            0.708       0.579      0.000       0.295       2.447      *
    SLOPELS           -5.266      18.198      0.388     -40.111      31.313
    LNVLS              0.196       0.965      0.416      -1.772       2.016

 BASTTDC    ON
    SWLS               0.304       0.527      0.277      -0.729       1.356
    SEX                1.646       0.742      0.012       0.221       3.147      *
    YOL               -0.278       0.094      0.002      -0.461      -0.093      *
    BASAGEC90         -0.230       0.095      0.008      -0.418      -0.043      *

 SWLS     WITH
    SLOPELS           -0.005       0.003      0.023      -0.012       0.000      *
    LNVLS             -0.155       0.097      0.048      -0.355       0.027

 SLOPELS  WITH
    LNVLS              0.001       0.003      0.274      -0.003       0.007

 Means
    SWLS               3.683       0.082      0.000       3.520       3.841      *
    SLOPELS            0.002       0.003      0.271      -0.005       0.009
    LNVLS             -1.971       0.113      0.000      -2.196      -1.751      *

 Intercepts
    BASTTDC            4.907       1.986      0.008       1.022       8.864      *
    BASHKR            18.477       0.672      0.000      17.149      19.790      *
```

```
    BASSSSC            1.331       0.151      0.000       1.031       1.626      *

 Variances
    SWLS               0.595       0.111      0.000       0.418       0.851      *
    PHYSCON            5.863       5.303      0.000       0.530      20.214      *
    SLOPELS            0.001       0.000      0.000       0.000       0.001      *
    LNVLS              0.381       0.143      0.000       0.171       0.725      *

 Residual Variances
    BASTTDC            6.517       1.805      0.000       2.492       9.784      *
    BASHKR            43.164       7.396      0.000      29.946      59.046      *
    BASSSSC            1.554       0.630      0.000       0.181       2.570      *


STANDARDIZED MODEL RESULTS


STDYX Standardization

                                Posterior  One-Tailed         95% C.I.
                    Estimate       S.D.      P-Value   Lower 2.5%  Upper 2.5%  Significance

Within-Level Standardized Estimates Averaged Over Clusters

 SLOPELS | SWLS ON
    TSBASE             0.013       0.025      0.294      -0.034       0.062

 LNVLS |
    SWLS               0.865       0.013      0.000       0.836       0.889      *

Between Level

 PHYSCON  BY
    BASHKR             0.343       0.133      0.000       0.104       0.612      *
    BASSSSC            0.649       0.168      0.000       0.341       0.966      *

 BASTTDC    ON
    PHYSCON            0.513       0.143      0.000       0.254       0.806      *
    SLOPELS           -0.038       0.130      0.388      -0.285       0.226
    LNVLS              0.037       0.168      0.416      -0.305       0.351

 BASTTDC    ON
    SWLS               0.071       0.121      0.277      -0.167       0.310
    SEX                0.145       0.063      0.012       0.019       0.265      *
    YOL               -0.177       0.059      0.002      -0.288      -0.059      *
    BASAGEC90         -0.133       0.055      0.008      -0.238      -0.025      *

 SWLS     WITH
    SLOPELS           -0.289       0.135      0.023      -0.532      -0.004      *
    LNVLS             -0.338       0.184      0.048      -0.650       0.058

 SLOPELS  WITH
    LNVLS              0.100       0.161      0.274      -0.224       0.404

 Means
    SWLS               4.776       0.459      0.000       3.949       5.743      *
    SLOPELS            0.086       0.141      0.271      -0.192       0.361
    LNVLS             -3.206       0.663      0.000      -4.744      -2.272      *

 Intercepts
    BASTTDC            1.479       0.597      0.008       0.300       2.651      *
    BASHKR             2.617       0.201      0.000       2.230       3.016      *
    BASSSSC            0.808       0.106      0.000       0.599       1.016      *

 Variances
    SWLS               1.000       0.000      0.000       1.000       1.000
    PHYSCON            1.000       0.000      0.000       1.000       1.000
    SLOPELS            1.000       0.000      0.000       1.000       1.000
    LNVLS              1.000       0.000      0.000       1.000       1.000

 Residual Variances
    BASTTDC            0.597       0.154      0.000       0.224       0.819      *
    BASHKR             0.882       0.098      0.000       0.625       0.989      *
    BASSSSC            0.578       0.223      0.000       0.067       0.884      *
```

```
STDY Standardization

                                Posterior  One-Tailed         95% C.I.
                    Estimate       S.D.      P-Value   Lower 2.5%  Upper 2.5%  Significance

Within-Level Standardized Estimates Averaged Over Clusters

 SLOPELS | SWLS ON
    TSBASE             0.003       0.007      0.334      -0.010       0.017

 LNVLS |
    SWLS               0.865       0.013      0.000       0.836       0.889      *

Between Level

 PHYSCON  BY
    BASHKR             0.343       0.133      0.000       0.104       0.612      *
    BASSSSC            0.649       0.168      0.000       0.341       0.966      *

 BASTTDC    ON
    PHYSCON            0.513       0.143      0.000       0.254       0.806      *
    SLOPELS           -0.038       0.130      0.388      -0.285       0.226
    LNVLS              0.037       0.168      0.416      -0.305       0.351

 BASTTDC    ON
    SWLS               0.071       0.121      0.277      -0.167       0.310
    SEX                0.497       0.215      0.012       0.066       0.910      *
    YOL               -0.084       0.028      0.002      -0.136      -0.028      *
    BASAGEC90         -0.069       0.028      0.008      -0.124      -0.013      *

 SWLS     WITH
    SLOPELS           -0.289       0.135      0.023      -0.532      -0.004      *
    LNVLS             -0.338       0.184      0.048      -0.650       0.058

 SLOPELS  WITH
    LNVLS              0.100       0.161      0.274      -0.224       0.404

 Means
    SWLS               4.776       0.459      0.000       3.949       5.743      *
    SLOPELS            0.086       0.141      0.271      -0.192       0.361
    LNVLS             -3.206       0.663      0.000      -4.744      -2.272      *

 Intercepts
    BASTTDC            1.479       0.597      0.008       0.300       2.651      *
    BASHKR             2.617       0.201      0.000       2.230       3.016      *
    BASSSSC            0.808       0.106      0.000       0.599       1.016      *

 Variances
    SWLS               1.000       0.000      0.000       1.000       1.000
    PHYSCON            1.000       0.000      0.000       1.000       1.000
    SLOPELS            1.000       0.000      0.000       1.000       1.000
    LNVLS              1.000       0.000      0.000       1.000       1.000

 Residual Variances
    BASTTDC            0.597       0.154      0.000       0.224       0.819      *
    BASHKR             0.882       0.098      0.000       0.625       0.989      *
    BASSSSC            0.578       0.223      0.000       0.067       0.884      *


STD Standardization

                                Posterior  One-Tailed         95% C.I.
                    Estimate       S.D.      P-Value   Lower 2.5%  Upper 2.5%  Significance

Within-Level Standardized Estimates Averaged Over Clusters

 SLOPELS | SWLS ON
    TSBASE             0.001       0.003      0.271      -0.003       0.007

 LNVLS |
    SWLS               0.163       0.024      0.000       0.131       0.224      *
```

```
Between Level

 PHYSCON  BY
    BASHKR             2.421       0.982      0.000       0.728       4.496      *
    BASSSSC            1.074       0.294      0.000       0.544       1.645      *

 BASTTDC    ON
    PHYSCON            1.707       0.507      0.000       0.822       2.771      *
    SLOPELS           -0.126       0.438      0.388      -0.966       0.755
    LNVLS              0.121       0.564      0.416      -1.016       1.202

 BASTTDC    ON
    SWLS               0.304       0.527      0.277      -0.729       1.356
    SEX                1.646       0.742      0.012       0.221       3.147      *
    YOL               -0.278       0.094      0.002      -0.461      -0.093      *
    BASAGEC90         -0.230       0.095      0.008      -0.418      -0.043      *

 SWLS     WITH
    SLOPELS           -0.221       0.115      0.023      -0.453      -0.003      *
    LNVLS             -0.258       0.146      0.048      -0.525       0.045

 SLOPELS  WITH
    LNVLS              0.100       0.161      0.274      -0.224       0.404

 Means
    SWLS               3.683       0.082      0.000       3.520       3.841      *
    SLOPELS            0.086       0.141      0.271      -0.192       0.361
    LNVLS             -3.206       0.663      0.000      -4.744      -2.272      *

 Intercepts
    BASTTDC            4.907       1.986      0.008       1.022       8.864      *
    BASHKR            18.477       0.672      0.000      17.149      19.790      *
    BASSSSC            1.331       0.151      0.000       1.031       1.626      *

 Variances
    SWLS               0.595       0.111      0.000       0.418       0.851      *
    PHYSCON            1.000       0.000      0.000       1.000       1.000
    SLOPELS            1.000       0.000      0.000       1.000       1.000
    LNVLS              1.000       0.000      0.000       1.000       1.000

 Residual Variances
    BASTTDC            6.517       1.805      0.000       2.492       9.784      *
    BASHKR            43.164       7.396      0.000      29.946      59.046      *
    BASSSSC            1.554       0.630      0.000       0.181       2.570      *


R-SQUARE

Within-Level R-Square Averaged Across Clusters

                                Posterior  One-Tailed         95% C.I.
    Variable        Estimate       S.D.      P-Value   Lower 2.5%  Upper 2.5%

    SWLS               0.135       0.013      0.000       0.111       0.163

Between Level

                                Posterior  One-Tailed         95% C.I.
    Variable        Estimate       S.D.      P-Value   Lower 2.5%  Upper 2.5%

    BASTTDC            0.403       0.154      0.000       0.181       0.776
    BASHKR             0.118       0.098      0.000       0.011       0.375
    BASSSSC            0.422       0.223      0.000       0.116       0.933



TECHNICAL 8 OUTPUT


   TECHNICAL 8 OUTPUT FOR BAYES ESTIMATION

     CHAIN    BSEED
     1        0
```

```
     2        285380

                POTENTIAL       PARAMETER WITH
     ITERATION    SCALE REDUCTION      HIGHEST PSR
     100              2.576               17
     200              2.150               20
     300              2.646               20
     400              2.959               20
     500              1.482               20
     600              1.252               10
     700              1.322               10
     800              1.633               23
     900              1.948               23
     1000             2.136               23
     1100             1.955               23
     1200             1.644               20
     1300             1.691               20
     1400             1.703               20
     1500             1.608               7
     1600             1.686               7
     1700             1.569               7
     1800             1.602               20
     1900             1.645               20
     2000             1.577               7
     2100             1.543               20
     2200             1.431               7
     2300             1.362               7
     2400             1.292               7
     2500             1.186               7
     2600             1.192               7
     2700             1.106               7
     2800             1.051               7
     2900             1.063               21
     3000             1.069               21
     3100             1.053               21
     3200             1.054               5
     3300             1.066               5
     3400             1.075               5
     3500             1.081               5
     3600             1.074               5
     3700             1.063               5
     3800             1.048               5
     3900             1.031               5
     4000             1.026               7
     4100             1.032               7
     4200             1.048               7
     4300             1.048               7
     4400             1.060               7
     4500             1.081               7
     4600             1.102               7
     4700             1.124               7
     4800             1.117               7
     4900             1.133               7
     5000             1.139               7
     5100             1.113               7
     5200             1.095               7
     5300             1.092               7
     5400             1.099               7
     5500             1.103               7
     5600             1.090               7
     5700             1.099               7
     5800             1.119               7
     5900             1.136               7
     6000             1.134               7
     6100             1.129               7
     6200             1.121               7
     6300             1.103               7
     6400             1.084               7
     6500             1.070               7
     6600             1.062               7
     6700             1.054               7
     6800             1.047               7
     6900             1.044               10
     7000             1.043               7
```

7100	1.043	10
7200	1.043	10
7300	1.039	10
7400	1.041	10
7500	1.046	10
7600	1.049	10
7700	1.053	10
7800	1.056	10
7900	1.059	10
8000	1.059	10
8100	1.059	10
8200	1.069	10
8300	1.080	10
8400	1.071	10
8500	1.075	10
8600	1.080	10
8700	1.080	10
8800	1.087	10
8900	1.086	10
9000	1.086	10
9100	1.094	10
9200	1.097	10
9300	1.096	10
9400	1.097	10
9500	1.093	10
9600	1.091	10
9700	1.086	10
9800	1.081	10
9900	1.085	10
10000	1.082	10
10100	1.076	10
10200	1.069	10
10300	1.067	10
10400	1.062	10
10500	1.061	10
10600	1.062	10
10700	1.057	10
10800	1.056	10
10900	1.055	10
11000	1.053	10
11100	1.052	10
11200	1.052	10
11300	1.052	10
11400	1.050	10
11500	1.049	10
11600	1.045	10
11700	1.044	10
11800	1.044	10
11900	1.042	10
12000	1.042	10
12100	1.042	10
12200	1.039	7
12300	1.043	7
12400	1.045	7
12500	1.044	7
12600	1.043	7
12700	1.045	7
12800	1.048	7
12900	1.049	7
13000	1.055	7
13100	1.053	7
13200	1.050	7
13300	1.047	7
13400	1.046	7
13500	1.046	7
13600	1.042	7
13700	1.040	7
13800	1.038	7
13900	1.036	7
14000	1.038	7
14100	1.040	7
14200	1.041	7
14300	1.047	7
14400	1.052	7

```
     14500           1.054           7
     14600           1.056           7
     14700           1.059           7
     14800           1.063           7
     14900           1.065           7
     15000           1.066           7
     15100           1.061           7
     15200           1.055           7
     15300           1.053           7
     15400           1.051           7
     15500           1.047           7
     15600           1.051           7
     15700           1.051           7
     15800           1.055           7
     15900           1.057           7
     16000           1.063           7
     16100           1.067           7
     16200           1.069           7
     16300           1.063           7
     16400           1.061           7
     16500           1.059           7
     16600           1.058           7
     16700           1.053           7
     16800           1.055           7
     16900           1.053           7
     17000           1.049           7
     17100           1.044           7
     17200           1.042           7
     17300           1.043           7
     17400           1.045           10
     17500           1.045           10
     17600           1.044           10
     17700           1.045           10
     17800           1.044           10
     17900           1.044           10
     18000           1.044           10
     18100           1.043           10
     18200           1.041           10
     18300           1.041           10
     18400           1.040           10
     18500           1.039           7
     18600           1.044           7
     18700           1.047           7
     18800           1.046           7
     18900           1.044           7
     19000           1.041           7
     19100           1.039           7
     19200           1.039           7
     19300           1.040           7
     19400           1.037           7
     19500           1.034           10
     19600           1.035           7
     19700           1.035           10
     19800           1.034           10
     19900           1.035           7
     20000           1.036           7
     20100           1.037           7
     20200           1.037           7
     20300           1.037           7
     20400           1.038           7
     20500           1.039           7
     20600           1.039           7
     20700           1.040           7
     20800           1.042           7
     20900           1.045           7
     21000           1.045           7
     21100           1.040           7
     21200           1.036           7
     21300           1.033           7
     21400           1.032           7
     21500           1.031           7
     21600           1.029           7
     21700           1.028           7
     21800           1.028           7
```

21900	1.025	7
22000	1.022	7
22100	1.022	7
22200	1.021	3
22300	1.020	3
22400	1.020	3
22500	1.022	7
22600	1.023	7
22700	1.022	7
22800	1.022	7
22900	1.021	7
23000	1.019	3
23100	1.018	3
23200	1.018	3
23300	1.018	3
23400	1.018	3
23500	1.018	3
23600	1.018	3
23700	1.018	3
23800	1.021	3
23900	1.023	3
24000	1.024	3
24100	1.025	3
24200	1.027	3
24300	1.029	3
24400	1.031	3
24500	1.033	3
24600	1.032	3
24700	1.032	3
24800	1.032	3
24900	1.031	3
25000	1.031	3
25100	1.031	3
25200	1.031	3
25300	1.032	3
25400	1.034	3
25500	1.034	3
25600	1.035	3
25700	1.035	3
25800	1.035	3
25900	1.034	3
26000	1.034	3
26100	1.033	3
26200	1.032	3
26300	1.031	3
26400	1.031	3
26500	1.031	3
26600	1.030	3
26700	1.030	3
26800	1.029	3
26900	1.029	3
27000	1.029	3
27100	1.029	3
27200	1.029	3
27300	1.029	3
27400	1.029	3
27500	1.029	3
27600	1.029	3
27700	1.029	3
27800	1.028	3
27900	1.028	3
28000	1.029	3
28100	1.029	3
28200	1.029	3
28300	1.029	3
28400	1.029	3
28500	1.029	3
28600	1.030	3
28700	1.029	3
28800	1.029	3
28900	1.029	3
29000	1.028	3
29100	1.027	3
29200	1.027	3

29300	1.027	3
29400	1.027	3
29500	1.027	3
29600	1.026	3
29700	1.026	3
29800	1.026	3
29900	1.026	3
30000	1.026	3
30100	1.026	3
30200	1.026	3
30300	1.027	3
30400	1.027	3
30500	1.027	3
30600	1.027	3
30700	1.028	3
30800	1.028	3
30900	1.027	10
31000	1.026	10
31100	1.026	10
31200	1.026	10
31300	1.026	10
31400	1.026	10
31500	1.026	10
31600	1.026	10
31700	1.026	10
31800	1.026	10
31900	1.026	10
32000	1.026	10
32100	1.025	10
32200	1.026	10
32300	1.025	10
32400	1.025	10
32500	1.024	10
32600	1.022	10
32700	1.020	10
32800	1.019	10
32900	1.019	10
33000	1.018	10
33100	1.017	10
33200	1.016	10
33300	1.015	10
33400	1.013	10
33500	1.013	10
33600	1.012	10
33700	1.011	10
33800	1.010	10
33900	1.009	10
34000	1.008	10
34100	1.007	10
34200	1.007	23
34300	1.007	23
34400	1.007	23
34500	1.007	23
34600	1.008	23
34700	1.010	23
34800	1.010	23
34900	1.010	23
35000	1.010	23
35100	1.010	23
35200	1.010	23
35300	1.010	23
35400	1.010	23
35500	1.010	23
35600	1.010	23
35700	1.010	23
35800	1.010	23
35900	1.010	23
36000	1.010	23
36100	1.010	23
36200	1.010	23
36300	1.011	23
36400	1.011	23
36500	1.010	23
36600	1.010	23

36700	1.009	23
36800	1.009	23
36900	1.009	23
37000	1.009	23
37100	1.008	23
37200	1.008	23
37300	1.007	23
37400	1.006	23
37500	1.006	23
37600	1.005	23
37700	1.005	23
37800	1.005	23
37900	1.005	23
38000	1.005	5
38100	1.006	5
38200	1.006	5
38300	1.006	5
38400	1.005	7
38500	1.006	5
38600	1.007	5
38700	1.007	5
38800	1.008	5
38900	1.008	5
39000	1.007	5
39100	1.008	5
39200	1.008	5
39300	1.008	5
39400	1.008	5
39500	1.009	5
39600	1.010	5
39700	1.010	5
39800	1.009	5
39900	1.008	5
40000	1.008	5
40100	1.008	5
40200	1.008	5
40300	1.007	5
40400	1.007	5
40500	1.006	5
40600	1.007	5
40700	1.007	5
40800	1.007	5
40900	1.006	5
41000	1.006	5
41100	1.006	5
41200	1.007	7
41300	1.007	7
41400	1.007	5
41500	1.007	5
41600	1.007	5
41700	1.007	5
41800	1.007	5
41900	1.008	5
42000	1.008	5
42100	1.008	5
42200	1.007	5
42300	1.008	5
42400	1.008	5
42500	1.009	5
42600	1.009	5
42700	1.009	5
42800	1.009	5
42900	1.009	5
43000	1.008	5
43100	1.008	5
43200	1.008	5
43300	1.008	5
43400	1.008	5
43500	1.008	5
43600	1.008	5
43700	1.008	5
43800	1.008	5
43900	1.008	5
44000	1.009	5

44100	1.009	5
44200	1.008	5
44300	1.008	5
44400	1.009	5
44500	1.009	5
44600	1.008	5
44700	1.009	5
44800	1.009	5
44900	1.010	5
45000	1.010	5
45100	1.009	5
45200	1.008	5
45300	1.007	5
45400	1.007	5
45500	1.006	5
45600	1.006	20
45700	1.006	20
45800	1.006	20
45900	1.006	5
46000	1.006	5
46100	1.006	5
46200	1.006	5
46300	1.006	5
46400	1.006	5
46500	1.006	5
46600	1.005	5
46700	1.004	7
46800	1.004	7
46900	1.005	7
47000	1.005	7
47100	1.006	7
47200	1.006	7
47300	1.006	7
47400	1.006	7
47500	1.006	7
47600	1.005	7
47700	1.005	7
47800	1.005	7
47900	1.005	7
48000	1.005	7
48100	1.004	7
48200	1.005	5
48300	1.005	5
48400	1.006	5
48500	1.007	5
48600	1.007	5
48700	1.007	5
48800	1.008	5
48900	1.009	5
49000	1.008	5
49100	1.008	5
49200	1.006	5
49300	1.006	5
49400	1.005	7
49500	1.006	7
49600	1.007	7
49700	1.007	7
49800	1.007	7
49900	1.007	7
50000	1.007	7
50100	1.008	7
50200	1.007	7
50300	1.007	7
50400	1.006	7
50500	1.006	7
50600	1.006	7
50700	1.006	7
50800	1.006	7
50900	1.005	7
51000	1.004	7
51100	1.004	7
51200	1.004	7
51300	1.005	7
51400	1.005	7

```
     51500           1.006               7
     51600           1.006               7
     51700           1.005               7
     51800           1.005               7
     51900           1.004               7
     52000           1.004               7
     52100           1.004               7
     52200           1.004               20
     52300           1.004               20
     52400           1.004               20
     52500           1.005               20
     52600           1.005               20
     52700           1.004               20
     52800           1.003               20
     52900           1.004               20
     53000           1.003               20
     53100           1.003               20
     53200           1.003               20
     53300           1.003               20
     53400           1.003               20
     53500           1.003               20
     53600           1.004               20
     53700           1.004               20
     53800           1.004               20
     53900           1.003               20
     54000           1.002               20
     54100           1.002               20


TECHNICAL 5 OUTPUT


DIAGRAM INFORMATION

  Mplus diagrams are currently not available for multilevel analysis.
  No diagram output was produced.


     Beginning Time:  18:25:34
        Ending Time:  18:34:45
       Elapsed Time:  00:09:11



MUTHEN & MUTHEN
3463 Stoner Ave.
Los Angeles, CA  90066

Tel: (310) 391-9971
Fax: (310) 391-8971
Web: www.StatModel.com
Support: Support@StatModel.com
```

Copyright (c) 1998-2020 Muthen & Muthen

Mplus Output Single Multilevel SEM – Positive Affect:

```
Mplus VERSION 8.5
MUTHEN & MUTHEN
10/13/2021  12:16 PM

INPUT INSTRUCTIONS

  TITLE:

  DATA: FILE = mpltry6.dat;

  VARIABLE:
   names =  pno wave sex yol verst nopart
         basagec90 agec90 tims tsbase
         ttd ttdcens basettd basttdc
         aut pil ema sac dep
         pa na swls adlinv hyp
         visus sfenerg sfsofunc sfpain sfgen sfchang
         anges anget akzet mortinv mortperc
         basadlinv basvisus bashkr bassssc
         bassfen bassfso bassfpain bassfgen bassfcha
         hkr sl sssco;
  usevariables = pa tsbase basttdc bashkr bassssc basagec90 sex yol;
  missing = .;
  within = tsbase;
  between = basttdc bashkr bassssc sex yol basagec90;

 DEFINE:
  center sex yol (grandmean);

 ANALYSIS:
  type = twolevel random;
  estimator = BAYES;
  chains = 2;
  biterations=1000000;
  BCONVERGENCE = 0.00125;

 MODEL:

  %WITHIN%

  slopepa | pa ON tsbase;
  lnvpa | pa;


  %BETWEEN%

  physcon by bashkr@1 bassssc;

  basttdc ON pa
             sex
             yol
             physcon
             basagec90
             slopepa
             lnvpa;

  [ pa*3.33357 ] (m5);
  [ slopepa*-0.01432 ] (ms5);
  [ lnvpa*-2.25007 ] (mv5);

  basttdc*6.66980;
  pa*0.41394;
  slopepa*0.00049;
  lnvpa*0.55329;

  pa with slopepa lnvpa;
  slopepa with lnvpa;

 MODEL PRIORS:
```

```
  m5 ~ N(3.20,0.116);
  ms5 ~ N(-0.044,0.000036);

 OUTPUT:
  standardized tech5 tech8 tech16;




*** WARNING in VARIABLE command
  Note that only the first 8 characters of variable names are used in the output.
  Shorten variable names to avoid any confusion.
*** WARNING in MODEL command
  In the MODEL command, the following variable is an x-variable on the BETWEEN
  level and a y-variable on the WITHIN level.  This variable will be treated
  as a y-variable on both levels:  PA
*** WARNING
  One or more individual-level variables have no variation within a
  cluster for the following clusters.

     Variable   Cluster IDs with no within-cluster variation

      PA          10130 10144 10313 11240 11741 15018 16142 16798 18650 11028

   3 WARNING(S) FOUND IN THE INPUT INSTRUCTIONS



SUMMARY OF ANALYSIS

Number of groups                                                 1
Number of observations                                         682

Number of dependent variables                                    4
Number of independent variables                                  4
Number of continuous latent variables                            3

Observed dependent variables

  Continuous
   BASTTDC     BASHKR      BASSSSC     PA

Observed independent variables
   TSBASE      BASAGEC9    SEX         YOL

Continuous latent variables
   PHYSCON     SLOPEPA     LNVPA

Variables with special functions

  Cluster variable      PNO

  Within variables
   TSBASE

  Between variables
   BASTTDC     BASHKR      BASSSSC     BASAGEC9    SEX         YOL

  Centering (GRANDMEAN)
   SEX         YOL


Estimator                                                    BAYES
Specifications for Bayesian Estimation
  Point estimate                                            MEDIAN
  Number of Markov chain Monte Carlo (MCMC) chains               2
  Random seed for the first chain                                0
  Starting value information                           UNPERTURBED
  Algorithm used for Markov chain Monte Carlo           GIBBS(PX1)
  Convergence criterion                                  0.125D-02
  Maximum number of iterations                             1000000
```

```
  K-th iteration used for thinning                               1

Input data file(s)
  mpltry6.dat
Input data format  FREE


SUMMARY OF DATA

     Number of clusters                        124

       Size (s)    Cluster ID with Size s

          1        10026 10130 10144 10313 10473 10558 10567 10637 10663
                   10893 10901 10907 10911 10939 10969 11157 11240 11294
                   11297 11741 15018 15177 15241 15319 15564 16142 16423
                   16610 16669 16798 17542 18507 18650
          2        15239 11258 10181 15510 10615 11341 16303 11371 16510
                   11384 11485 11028 16862 10211 17780 18004 18252 15175
                   10228
          3        11573 11295 17130 11100 17745 10340 10460 10461 10210
                   11501
          4        10904 11457 10434 11493 10811 17593
          5        15424 10666 12411 11343 11005
          6        10159 15092 15121 15426 11593 11163 10139 16220 19067
          7        10354 11230 10894 10724 10470
          8        10252 10528 11038 10033 11336 11253
          9        15623 11055
         10        12037 10167 17197 15009 19500
         11        11233 10444 15714 10533
         12        11079 10577 15378 12714 10986 10428
         13        11420 11002
         14        11450
         15        10108 10906 10551 15231 10902
         16        15141 10940 16103 15472 11528 11378



COVARIANCE COVERAGE OF DATA

Minimum covariance coverage value   0.100

     Number of missing data patterns            8


     PROPORTION OF DATA PRESENT


           Covariance Coverage
              BASTTDC       BASHKR        BASSSSC       PA            TSBASE
              ________      ________      ________      ________      ________
 BASTTDC        0.944
 BASHKR         0.912         0.968
 BASSSSC        0.935         0.968         0.991
 PA             0.908         0.943         0.956         0.962
 TSBASE         0.944         0.968         0.991         0.962         1.000
 BASAGEC9       0.944         0.968         0.991         0.962         1.000
 SEX            0.944         0.968         0.991         0.962         1.000
 YOL            0.944         0.968         0.991         0.962         1.000


           Covariance Coverage
              BASAGEC9      SEX           YOL
              ________      ________      ________
 BASAGEC9       1.000
 SEX            1.000         1.000
 YOL            1.000         1.000         1.000



UNIVARIATE SAMPLE STATISTICS


     UNIVARIATE HIGHER-ORDER MOMENT DESCRIPTIVE STATISTICS
```

Variable/ Sample Size	Mean/ Variance	Skewness/ Kurtosis	Minimum/ Maximum	% with Min/Max	Percentiles 20%/60%	40%/80%	Median
BASTTDC	5.519	0.456	0.250	0.83%	2.250	4.250	5.083
120.000	10.267	-0.614	12.667	1.67%	6.083	8.250	
BASHKR	18.519	1.282	5.330	0.89%	12.330	16.500	17.750
112.000	48.308	2.780	49.000	0.89%	18.670	23.330	
BASSSSC	1.333	0.640	0.000	53.33%	0.000	0.000	0.000
120.000	2.606	-1.294	4.000	17.50%	1.000	3.000	
PA	3.172	0.047	1.380	0.15%	2.600	3.000	3.135
656.000	0.406	-0.288	4.800	0.30%	3.300	3.700	
TSBASE	36.711	0.258	0.000	3.08%	13.000	27.000	36.000
682.000	594.554	-0.917	91.000	0.59%	41.000	60.000	
BASAGEC90	0.106	0.407	-3.917	0.81%	-2.833	-1.250	-0.792
124.000	8.313	-1.090	6.417	0.81%	0.500	3.083	
SEX	0.000	-1.426	-0.790	20.97%	-0.790	0.210	0.210
124.000	0.166	0.035	0.210	79.03%	0.210	0.210	
YOL	0.000	0.868	-3.387	20.97%	-3.387	-1.387	-1.387
124.000	8.737	-0.315	5.613	17.74%	0.613	0.613	

THE MODEL ESTIMATION TERMINATED NORMALLY

USE THE FBITERATIONS OPTION TO INCREASE THE NUMBER OF ITERATIONS BY A FACTOR
OF AT LEAST TWO TO CHECK CONVERGENCE AND THAT THE PSR VALUE DOES NOT INCREASE.

MODEL FIT INFORMATION

Number of Free Parameters 24

Information Criteria

Deviance (DIC) 2486.374
Estimated Number of Parameters (pD) 230.312

MODEL RESULTS

	Estimate	Posterior S.D.	One-Tailed P-Value	95% C.I. Lower 2.5%	Upper 2.5%	Significance
**Within Level**						
**Between Level**						
PHYSCON BY						
BASHKR	1.000	0.000	0.000	1.000	1.000	
BASSSSC	0.421	0.643	0.000	0.153	2.126	*
BASTTDC ON						
PHYSCON	0.639	0.717	0.000	0.228	2.835	*
SLOPEPA	13.729	16.935	0.212	-19.928	46.588	
LNVPA	-0.663	1.016	0.241	-2.807	1.249	
BASTTDC ON						
PA	0.431	0.662	0.253	-0.830	1.768	
SEX	1.559	0.740	0.015	0.154	3.050	*
YOL	-0.252	0.092	0.003	-0.432	-0.073	*
BASAGEC90	-0.238	0.095	0.006	-0.425	-0.051	*
PA WITH						
SLOPEPA	-0.001	0.002	0.321	-0.005	0.003	
LNVPA	0.066	0.078	0.177	-0.073	0.237	
SLOPEPA WITH						
LNVPA	-0.002	0.003	0.261	-0.007	0.003	
Means						
PA	3.315	0.063	0.000	3.192	3.438	*
SLOPEPA	-0.016	0.003	0.000	-0.022	-0.010	*

```
    LNVPA             -2.298       0.128      0.000      -2.554      -2.047      *

 Intercepts
    BASTTDC            2.827       3.705      0.215      -4.636      10.039
    BASHKR            18.483       0.668      0.000      17.172      19.799      *
    BASSSSC            1.330       0.151      0.000       1.032       1.625      *

 Variances
    PA                 0.329       0.062      0.000       0.229       0.471      *
    PHYSCON            6.012       5.653      0.000       0.284      21.351      *
    SLOPEPA            0.001       0.000      0.000       0.000       0.001      *
    LNVPA              0.352       0.152      0.000       0.138       0.733      *

 Residual Variances
    BASTTDC            6.455       1.734      0.000       2.704       9.658      *
    BASHKR            43.088       7.511      0.000      29.273      59.083      *
    BASSSSC            1.619       0.621      0.000       0.215       2.629      *


STANDARDIZED MODEL RESULTS


STDYX Standardization

                                Posterior  One-Tailed         95% C.I.
                    Estimate       S.D.      P-Value   Lower 2.5%  Upper 2.5%  Significance

Within-Level Standardized Estimates Averaged Over Clusters

 SLOPEPA | PA ON
    TSBASE            -0.186       0.024      0.000      -0.232      -0.138      *

 LNVPA |
    PA                 0.831       0.015      0.000       0.802       0.858      *

Between Level

 PHYSCON  BY
    BASHKR             0.348       0.140      0.000       0.076       0.626      *
    BASSSSC            0.632       0.171      0.000       0.312       0.960      *

 BASTTDC    ON
    PHYSCON            0.480       0.151      0.000       0.200       0.788      *
    SLOPEPA            0.102       0.126      0.212      -0.145       0.346
    LNVPA             -0.121       0.169      0.241      -0.443       0.218

 BASTTDC    ON
    PA                 0.076       0.115      0.253      -0.142       0.309
    SEX                0.139       0.063      0.015       0.013       0.261      *
    YOL               -0.163       0.058      0.003      -0.274      -0.047      *
    BASAGEC90         -0.140       0.055      0.006      -0.246      -0.029      *

 PA       WITH
    SLOPEPA           -0.068       0.143      0.321      -0.344       0.217
    LNVPA              0.203       0.207      0.177      -0.229       0.576

 SLOPEPA  WITH
    LNVPA             -0.113       0.170      0.261      -0.433       0.230

 Means
    PA                 5.782       0.544      0.000       4.817       6.949      *
    SLOPEPA           -0.645       0.122      0.000      -0.886      -0.407      *
    LNVPA             -3.877       0.920      0.000      -6.105      -2.671      *

 Intercepts
    BASTTDC            0.864       1.117      0.215      -1.388       3.006
    BASHKR             2.612       0.201      0.000       2.224       3.011      *
    BASSSSC            0.807       0.106      0.000       0.600       1.012      *

 Variances
    PA                 1.000       0.000      0.000       1.000       1.000
    PHYSCON            1.000       0.000      0.000       1.000       1.000
    SLOPEPA            1.000       0.000      0.000       1.000       1.000
    LNVPA              1.000       0.000      0.000       1.000       1.000
```

```
 Residual Variances
    BASTTDC            0.609       0.149      0.000       0.248       0.822      *
    BASHKR             0.879       0.104      0.000       0.608       0.994      *
    BASSSSC            0.601       0.219      0.000       0.079       0.903      *


STDY Standardization

                                Posterior  One-Tailed         95% C.I.
                    Estimate       S.D.      P-Value   Lower 2.5%  Upper 2.5%  Significance

Within-Level Standardized Estimates Averaged Over Clusters

 SLOPEPA | PA ON
    TSBASE            -0.031       0.007      0.000      -0.046      -0.018      *

 LNVPA |
    PA                 0.831       0.015      0.000       0.802       0.858      *

Between Level

 PHYSCON  BY
    BASHKR             0.348       0.140      0.000       0.076       0.626      *
    BASSSSC            0.632       0.171      0.000       0.312       0.960      *

 BASTTDC    ON
    PHYSCON            0.480       0.151      0.000       0.200       0.788      *
    SLOPEPA            0.102       0.126      0.212      -0.145       0.346
    LNVPA             -0.121       0.169      0.241      -0.443       0.218

 BASTTDC    ON
    PA                 0.076       0.115      0.253      -0.142       0.309
    SEX                0.476       0.218      0.015       0.046       0.896      *
    YOL               -0.077       0.027      0.003      -0.129      -0.022      *
    BASAGEC90         -0.073       0.029      0.006      -0.128      -0.015      *

 PA       WITH
    SLOPEPA           -0.068       0.143      0.321      -0.344       0.217
    LNVPA              0.203       0.207      0.177      -0.229       0.576

 SLOPEPA  WITH
    LNVPA             -0.113       0.170      0.261      -0.433       0.230

 Means
    PA                 5.782       0.544      0.000       4.817       6.949      *
    SLOPEPA           -0.645       0.122      0.000      -0.886      -0.407      *
    LNVPA             -3.877       0.920      0.000      -6.105      -2.671      *

 Intercepts
    BASTTDC            0.864       1.117      0.215      -1.388       3.006
    BASHKR             2.612       0.201      0.000       2.224       3.011      *
    BASSSSC            0.807       0.106      0.000       0.600       1.012      *

 Variances
    PA                 1.000       0.000      0.000       1.000       1.000
    PHYSCON            1.000       0.000      0.000       1.000       1.000
    SLOPEPA            1.000       0.000      0.000       1.000       1.000
    LNVPA              1.000       0.000      0.000       1.000       1.000

 Residual Variances
    BASTTDC            0.609       0.149      0.000       0.248       0.822      *
    BASHKR             0.879       0.104      0.000       0.608       0.994      *
    BASSSSC            0.601       0.219      0.000       0.079       0.903      *


STD Standardization

                                Posterior  One-Tailed         95% C.I.
                    Estimate       S.D.      P-Value   Lower 2.5%  Upper 2.5%  Significance

Within-Level Standardized Estimates Averaged Over Clusters

 SLOPEPA | PA ON
```

```
    TSBASE            -0.012       0.002      0.000      -0.017      -0.007      *

 LNVPA |
    PA                 0.122       0.015      0.000       0.100       0.161      *

Between Level

 PHYSCON  BY
    BASHKR             2.452       1.041      0.000       0.533       4.621      *
    BASSSSC            1.045       0.298      0.000       0.502       1.628      *

 BASTTDC    ON
    PHYSCON            1.572       0.526      0.000       0.641       2.689      *
    SLOPEPA            0.334       0.417      0.212      -0.486       1.151
    LNVPA             -0.396       0.559      0.241      -1.476       0.727

 BASTTDC    ON
    PA                 0.431       0.662      0.253      -0.830       1.768
    SEX                1.559       0.740      0.015       0.154       3.050      *
    YOL               -0.252       0.092      0.003      -0.432      -0.073      *
    BASAGEC90         -0.238       0.095      0.006      -0.425      -0.051      *

 PA       WITH
    SLOPEPA           -0.039       0.085      0.321      -0.213       0.120
    LNVPA              0.115       0.123      0.177      -0.130       0.352

 SLOPEPA  WITH
    LNVPA             -0.113       0.170      0.261      -0.433       0.230

 Means
    PA                 3.315       0.063      0.000       3.192       3.438      *
    SLOPEPA           -0.645       0.122      0.000      -0.886      -0.407      *
    LNVPA             -3.877       0.920      0.000      -6.105      -2.671      *

 Intercepts
    BASTTDC            2.827       3.705      0.215      -4.636      10.039
    BASHKR            18.483       0.668      0.000      17.172      19.799      *
    BASSSSC            1.330       0.151      0.000       1.032       1.625      *

 Variances
    PA                 0.329       0.062      0.000       0.229       0.471      *
    PHYSCON            1.000       0.000      0.000       1.000       1.000
    SLOPEPA            1.000       0.000      0.000       1.000       1.000
    LNVPA              1.000       0.000      0.000       1.000       1.000

 Residual Variances
    BASTTDC            6.455       1.734      0.000       2.704       9.658      *
    BASHKR            43.088       7.511      0.000      29.273      59.083      *
    BASSSSC            1.619       0.621      0.000       0.215       2.629      *


R-SQUARE

Within-Level R-Square Averaged Across Clusters

                                 Posterior  One-Tailed         95% C.I.
    Variable        Estimate       S.D.      P-Value   Lower 2.5%  Upper 2.5%

    PA                 0.169       0.015      0.000       0.141       0.198

Between Level

                                 Posterior  One-Tailed         95% C.I.
    Variable        Estimate       S.D.      P-Value   Lower 2.5%  Upper 2.5%

    BASTTDC            0.391       0.149      0.000       0.178       0.752
    BASHKR             0.121       0.104      0.000       0.006       0.392
    BASSSSC            0.399       0.219      0.000       0.097       0.921



TECHNICAL 8 OUTPUT
```

```
TECHNICAL 8 OUTPUT FOR BAYES ESTIMATION

  CHAIN    BSEED
  1        0
  2        285380

                POTENTIAL       PARAMETER WITH
  ITERATION    SCALE REDUCTION      HIGHEST PSR
  100              3.674              3
  200              1.843              12
  300              1.269              12
  400              1.447              23
  500              1.692              23
  600              1.571              3
  700              1.579              3
  800              1.375              3
  900              1.131              20
  1000             1.126              17
  1100             1.107              17
  1200             1.048              23
  1300             1.106              23
  1400             1.235              23
  1500             1.305              23
  1600             1.354              23
  1700             1.319              23
  1800             1.235              7
  1900             1.171              23
  2000             1.163              20
  2100             1.212              3
  2200             1.254              3
  2300             1.302              3
  2400             1.391              3
  2500             1.397              3
  2600             1.400              3
  2700             1.388              3
  2800             1.346              3
  2900             1.323              3
  3000             1.314              3
  3100             1.295              3
  3200             1.306              3
  3300             1.294              3
  3400             1.240              3
  3500             1.206              3
  3600             1.178              3
  3700             1.150              3
  3800             1.132              3
  3900             1.084              3
  4000             1.052              3
  4100             1.039              3
  4200             1.036              3
  4300             1.025              3
  4400             1.021              7
  4500             1.032              23
  4600             1.037              23
  4700             1.046              23
  4800             1.055              23
  4900             1.061              23
  5000             1.060              23
  5100             1.054              23
  5200             1.055              23
  5300             1.042              23
  5400             1.037              10
  5500             1.035              10
  5600             1.037              10
  5700             1.033              10
  5800             1.033              10
  5900             1.031              10
  6000             1.030              10
  6100             1.029              10
  6200             1.027              10
  6300             1.027              10
  6400             1.034              3
  6500             1.045              3
```

```
6600            1.059           3
6700            1.077           3
6800            1.078           3
6900            1.079           3
7000            1.079           3
7100            1.077           3
7200            1.073           3
7300            1.075           3
7400            1.076           3
7500            1.074           3
7600            1.074           3
7700            1.086           3
7800            1.101           3
7900            1.050           3
8000            1.051           3
8100            1.063           3
8200            1.073           3
8300            1.075           3
8400            1.077           3
8500            1.082           17
8600            1.089           17
8700            1.104           17
8800            1.115           17
8900            1.126           17
9000            1.141           17
9100            1.158           17
9200            1.157           17
9300            1.165           17
9400            1.159           17
9500            1.123           17
9600            1.114           17
9700            1.117           17
9800            1.121           17
9900            1.107           17
10000           1.087           3
10100           1.084           3
10200           1.082           3
10300           1.080           3
10400           1.079           3
10500           1.080           17
10600           1.081           17
10700           1.079           17
10800           1.081           17
10900           1.082           17
11000           1.083           17
11100           1.085           17
11200           1.078           17
11300           1.073           17
11400           1.076           17
11500           1.071           17
11600           1.069           3
11700           1.068           3
11800           1.067           3
11900           1.066           3
12000           1.066           3
12100           1.066           3
12200           1.065           3
12300           1.065           3
12400           1.065           3
12500           1.065           3
12600           1.064           3
12700           1.062           3
12800           1.061           3
12900           1.058           3
13000           1.053           3
13100           1.051           3
13200           1.049           3
13300           1.048           3
13400           1.045           3
13500           1.044           3
13600           1.043           3
13700           1.042           3
13800           1.042           3
13900           1.042           3
```

```
14000          1.042          10
14100          1.042          10
14200          1.042          10
14300          1.042          3
14400          1.041          10
14500          1.041          10
14600          1.041          10
14700          1.041          10
14800          1.040          10
14900          1.040          10
15000          1.040          10
15100          1.039          10
15200          1.039          10
15300          1.039          10
15400          1.038          10
15500          1.037          10
15600          1.035          10
15700          1.033          10
15800          1.030          10
15900          1.027          3
16000          1.028          3
16100          1.026          3
16200          1.029          3
16300          1.028          3
16400          1.026          3
16500          1.024          3
16600          1.022          3
16700          1.020          3
16800          1.022          20
16900          1.024          20
17000          1.024          20
17100          1.020          20
17200          1.017          20
17300          1.015          20
17400          1.014          20
17500          1.013          20
17600          1.012          20
17700          1.012          20
17800          1.012          20
17900          1.015          20
18000          1.018          20
18100          1.018          20
18200          1.018          20
18300          1.018          20
18400          1.020          20
18500          1.020          20
18600          1.020          20
18700          1.022          20
18800          1.023          20
18900          1.025          20
19000          1.027          20
19100          1.031          20
19200          1.030          20
19300          1.030          20
19400          1.034          20
19500          1.036          20
19600          1.042          20
19700          1.048          20
19800          1.052          20
19900          1.057          20
20000          1.061          20
20100          1.063          20
20200          1.065          20
20300          1.066          20
20400          1.065          20
20500          1.063          20
20600          1.059          20
20700          1.059          20
20800          1.054          20
20900          1.052          20
21000          1.052          20
21100          1.053          20
21200          1.048          20
21300          1.044          20
```

```
21400          1.046          20
21500          1.049          20
21600          1.049          20
21700          1.051          20
21800          1.052          20
21900          1.052          20
22000          1.048          20
22100          1.046          20
22200          1.039          20
22300          1.038          20
22400          1.039          20
22500          1.038          20
22600          1.037          20
22700          1.036          20
22800          1.035          20
22900          1.034          20
23000          1.033          20
23100          1.033          20
23200          1.032          20
23300          1.032          20
23400          1.033          20
23500          1.034          20
23600          1.034          20
23700          1.031          20
23800          1.030          20
23900          1.029          20
24000          1.030          3
24100          1.033          3
24200          1.034          3
24300          1.034          3
24400          1.034          10
24500          1.035          10
24600          1.036          3
24700          1.037          3
24800          1.037          3
24900          1.037          3
25000          1.038          20
25100          1.038          20
25200          1.038          17
25300          1.039          17
25400          1.043          17
25500          1.046          17
25600          1.046          17
25700          1.044          17
25800          1.044          20
25900          1.044          20
26000          1.044          20
26100          1.042          20
26200          1.043          20
26300          1.044          20
26400          1.046          20
26500          1.046          20
26600          1.048          20
26700          1.049          20
26800          1.047          20
26900          1.048          20
27000          1.050          20
27100          1.052          20
27200          1.053          20
27300          1.053          20
27400          1.050          20
27500          1.049          20
27600          1.051          20
27700          1.050          20
27800          1.048          20
27900          1.043          20
28000          1.040          20
28100          1.038          20
28200          1.035          20
28300          1.038          20
28400          1.041          20
28500          1.042          20
28600          1.042          20
28700          1.043          20
```

```
     28800             1.045              20
     28900             1.043              20
     29000             1.041              20
     29100             1.042              20
     29200             1.044              20
     29300             1.042              20
     29400             1.042              20
     29500             1.044              20
     29600             1.045              20
     29700             1.045              20
     29800             1.043              20
     29900             1.044              20
     30000             1.041              20
     30100             1.037              20
     30200             1.036              20
     30300             1.037              20
     30400             1.036              20
     30500             1.037              20
     30600             1.035              20
     30700             1.032              20
     30800             1.031              20
     30900             1.032              20
     31000             1.032              20
     31100             1.032              20
     31200             1.033              20
     31300             1.033              20
     31400             1.032              20
     31500             1.031              20
     31600             1.030              20
     31700             1.028              20
     31800             1.028              20
     31900             1.026              20
     32000             1.022              20
     32100             1.022              10
     32200             1.022              10
     32300             1.023              10
     32400             1.023              10
     32500             1.023              10
     32600             1.023              10
     32700             1.023              10
     32800             1.024              10
     32900             1.024              10
     33000             1.024              10
     33100             1.023              10
     33200             1.022              10
     33300             1.019              10
     33400             1.019              10
     33500             1.020              10
     33600             1.021              10
     33700             1.021              10
     33800             1.022              10
     33900             1.021              10
     34000             1.021              10
     34100             1.020              10
     34200             1.019              10
     34300             1.019              10
     34400             1.019              10
     34500             1.018              10
     34600             1.018              10
     34700             1.018              10
     34800             1.018              10
     34900             1.018              10
     35000             1.019              10
     35100             1.020              10
     35200             1.019              10
     35300             1.020              10
     35400             1.021              10
     35500             1.021              10
     35600             1.021              10
     35700             1.021              10
     35800             1.021              10
     35900             1.020              10
     36000             1.020              10
     36100             1.020              10
```

```
36200          1.020          10
36300          1.020          10
36400          1.020          10
36500          1.019          10
36600          1.019          10
36700          1.018          10
36800          1.017          10
36900          1.017          10
37000          1.017          10
37100          1.017          10
37200          1.017          10
37300          1.016          10
37400          1.016          10
37500          1.016          10
37600          1.016          10
37700          1.016          10
37800          1.016          10
37900          1.015          10
38000          1.015          10
38100          1.015          10
38200          1.016          10
38300          1.016          10
38400          1.018          10
38500          1.018          10
38600          1.017          10
38700          1.017          10
38800          1.018          10
38900          1.019          10
39000          1.019          10
39100          1.019          10
39200          1.019          10
39300          1.019          10
39400          1.019          10
39500          1.018          10
39600          1.018          10
39700          1.018          10
39800          1.018          10
39900          1.018          10
40000          1.019          10
40100          1.020          10
40200          1.020          10
40300          1.020          10
40400          1.020          10
40500          1.020          10
40600          1.020          10
40700          1.020          10
40800          1.019          10
40900          1.019          10
41000          1.019          10
41100          1.019          10
41200          1.019          10
41300          1.019          10
41400          1.019          10
41500          1.019          10
41600          1.019          10
41700          1.017          10
41800          1.014          10
41900          1.012          10
42000          1.010          10
42100          1.009          10
42200          1.007          10
42300          1.006          5
42400          1.006          21
42500          1.006          21
42600          1.005          21
42700          1.006          21
42800          1.006          21
42900          1.005          21
43000          1.005          21
43100          1.005          21
43200          1.006          21
43300          1.005          21
43400          1.005          21
43500          1.007          3
```

```
     43600           1.008           3
     43700           1.010           3
     43800           1.010           3
     43900           1.008           3
     44000           1.007           3
     44100           1.007           3
     44200           1.007           3
     44300           1.007           3
     44400           1.006           3
     44500           1.006           3
     44600           1.006           3
     44700           1.005           3
     44800           1.005           3
     44900           1.005           3
     45000           1.005           3
     45100           1.005           3
     45200           1.005           3
     45300           1.005           3
     45400           1.004           3
     45500           1.005           3
     45600           1.005           3
     45700           1.005           3
     45800           1.005           3
     45900           1.005           3
     46000           1.005           3
     46100           1.005           3
     46200           1.004           3
     46300           1.004           3
     46400           1.004           3
     46500           1.004           3
     46600           1.004           3
     46700           1.004           3
     46800           1.005           3
     46900           1.005           3
     47000           1.005           3
     47100           1.005           3
     47200           1.005           3
     47300           1.005           3
     47400           1.005           3
     47500           1.005           3
     47600           1.005           3
     47700           1.005           3
     47800           1.006           3
     47900           1.006           3
     48000           1.007           3
     48100           1.007           3
     48200           1.008           3
     48300           1.008           3
     48400           1.008           3
     48500           1.008           3
     48600           1.008           3
     48700           1.008           3
     48800           1.008           3
     48900           1.007           3
     49000           1.006           3
     49100           1.006           3
     49200           1.004           3
     49300           1.004           3
     49400           1.004           3
     49500           1.004           3
     49600           1.004           3
     49700           1.004           3
     49800           1.003           3
     49900           1.003           3
     50000           1.003           3
     50100           1.002           7


TECHNICAL 5 OUTPUT


DIAGRAM INFORMATION

  Mplus diagrams are currently not available for multilevel analysis.
```

```
  No diagram output was produced.


     Beginning Time:  12:16:40
        Ending Time:  12:25:09
       Elapsed Time:  00:08:29
```


```
MUTHEN & MUTHEN
3463 Stoner Ave.
Los Angeles, CA  90066

Tel: (310) 391-9971
Fax: (310) 391-8971
Web: www.StatModel.com
Support: Support@StatModel.com
```


Copyright (c) 1998-2020 Muthen & Muthen

Mplus Output Single Multilevel SEM – Negative Affect:

```
Mplus VERSION 8.5
MUTHEN & MUTHEN
10/15/2021   9:59 PM

INPUT INSTRUCTIONS

  TITLE:

  DATA: FILE = mpltry6.dat;

  VARIABLE:
   names =  pno wave sex yol verst nopart
         basagec90 agec90 tims tsbase
         ttd ttdcens basettd basttdc
         aut pil ema sac dep
         pa na swls adlinv hyp
         visus sfenerg sfsofunc sfpain sfgen sfchang
         anges anget akzet mortinv mortperc
         basadlinv basvisus bashkr bassssc
         bassfen bassfso bassfpain bassfgen bassfcha
         hkr sl sssco;
  usevariables = na tsbase basttdc bashkr bassssc basagec90 sex yol;
  cluster = PNO;
  missing = .;
  within = tsbase;
  between = basttdc bashkr bassssc sex yol basagec90;

 DEFINE:

  center sex yol (grandmean);

 ANALYSIS:
  type = twolevel random;
  estimator = BAYES;
  chains = 2;
  biterations=1500000;
  BCONVERGENCE = 0.00125;

 MODEL:

  %WITHIN%

  slopena | na ON tsbase;
  lnvna | na;

  %BETWEEN%

  physcon BY bashkr@1
             bassssc*0.44;

  basttdc ON na*-1.3
            sex*1.6
            yol*-0.27
            basagec90*-0.25
            physcon*0.63
            slopena*2.5
            lnvna*2.2;

  na WITH slopena*-0.004;
  na WITH lnvna*0.166;
  slopena WITH lnvna*-0.0004;

  [ basttdc* ];
  [ bashkr* ];
  [ bassssc* ];
  [ na*2.163 ] (m7);
  [ slopena*-0.009 ] (ms7);
  [ lnvna*-2.403 ] (mv7);
```

```
  basttdc*6.65222;
  bashkr*;
  bassssc*;
  na*0.39314;
  physcon*6.83504;
  slopena*0.00055;
  lnvna*0.22033;


 MODEL PRIORS:
  m7 ~ N(2.12,0.102);
  ms7 ~ N(-0.029,0.00017);

 OUTPUT:
  standardized tech5 tech8 tech16;




*** WARNING in VARIABLE command
  Note that only the first 8 characters of variable names are used in the output.
  Shorten variable names to avoid any confusion.
*** WARNING in MODEL command
  In the MODEL command, the following variable is an x-variable on the BETWEEN
  level and a y-variable on the WITHIN level.  This variable will be treated
  as a y-variable on both levels:  NA
*** WARNING
  One or more individual-level variables have no variation within a
  cluster for the following clusters.

     Variable   Cluster IDs with no within-cluster variation

      NA          10130 10144 10313 11240 11741 15018 16142 16798 11371 11295

   3 WARNING(S) FOUND IN THE INPUT INSTRUCTIONS




SUMMARY OF ANALYSIS

Number of groups                                                 1
Number of observations                                         682

Number of dependent variables                                    4
Number of independent variables                                  4
Number of continuous latent variables                            3

Observed dependent variables

  Continuous
   BASTTDC     BASHKR      BASSSSC     NA

Observed independent variables
   TSBASE      BASAGEC9    SEX         YOL

Continuous latent variables
   PHYSCON     SLOPENA     LNVNA

Variables with special functions

  Cluster variable      PNO

  Within variables
   TSBASE

  Between variables
   BASTTDC     BASHKR      BASSSSC     BASAGEC9    SEX         YOL

  Centering (GRANDMEAN)
   SEX         YOL
```

```
Estimator                                                   BAYES
Specifications for Bayesian Estimation
  Point estimate                                           MEDIAN
  Number of Markov chain Monte Carlo (MCMC) chains              2
  Random seed for the first chain                               0
  Starting value information                          UNPERTURBED
  Algorithm used for Markov chain Monte Carlo          GIBBS(PX1)
  Convergence criterion                                 0.125D-02
  Maximum number of iterations                            1500000
  K-th iteration used for thinning                              1

Input data file(s)
  mpltry6.dat
Input data format  FREE


SUMMARY OF DATA

     Number of clusters                       124

       Size (s)    Cluster ID with Size s

          1        10026 10130 10144 10313 10473 10558 10567 10637 10663
                   10893 10901 10907 10911 10939 10969 11157 11240 11294
                   11297 11741 15018 15177 15241 15319 15564 16142 16423
                   16610 16669 16798 17542 18507 18650
          2        15239 11258 10181 15510 10615 11341 16303 11371 16510
                   11384 11485 11028 16862 10211 17780 18004 18252 15175
                   10228
          3        11573 11295 17130 11100 17745 10340 10460 10461 10210
                   11501
          4        10904 11457 10434 11493 10811 17593
          5        15424 10666 12411 11343 11005
          6        10159 15092 15121 15426 11593 11163 10139 16220 19067
          7        10354 11230 10894 10724 10470
          8        10252 10528 11038 10033 11336 11253
          9        15623 11055
         10        12037 10167 17197 15009 19500
         11        11233 10444 15714 10533
         12        11079 10577 15378 12714 10986 10428
         13        11420 11002
         14        11450
         15        10108 10906 10551 15231 10902
         16        15141 10940 16103 15472 11528 11378



COVARIANCE COVERAGE OF DATA

Minimum covariance coverage value   0.100

     Number of missing data patterns            7


     PROPORTION OF DATA PRESENT


           Covariance Coverage
              BASTTDC       BASHKR        BASSSSC       NA            TSBASE
              ________      ________      ________      ________      ________
 BASTTDC        0.944
 BASHKR         0.912         0.968
 BASSSSC        0.935         0.968         0.991
 NA             0.911         0.947         0.960         0.966
 TSBASE         0.944         0.968         0.991         0.966         1.000
 BASAGEC9       0.944         0.968         0.991         0.966         1.000
 SEX            0.944         0.968         0.991         0.966         1.000
 YOL            0.944         0.968         0.991         0.966         1.000


           Covariance Coverage
              BASAGEC9      SEX           YOL
              ________      ________      ________
 BASAGEC9       1.000
```

```
 SEX            1.000         1.000
 YOL            1.000         1.000         1.000


UNIVARIATE SAMPLE STATISTICS


     UNIVARIATE HIGHER-ORDER MOMENT DESCRIPTIVE STATISTICS

         Variable/         Mean/     Skewness/   Minimum/ % with                Percentiles
        Sample Size      Variance    Kurtosis    Maximum  Min/Max      20%/60%    40%/80%    Median

     BASTTDC                5.519       0.456       0.250    0.83%       2.250      4.250      5.083
             120.000       10.267      -0.614      12.667    1.67%       6.083      8.250
     BASHKR                18.519       1.282       5.330    0.89%      12.330     16.500     17.750
             112.000       48.308       2.780      49.000    0.89%      18.670     23.330
     BASSSSC                1.333       0.640       0.000   53.33%       0.000      0.000      0.000
             120.000        2.606      -1.294       4.000   17.50%       1.000      3.000
     NA                     2.046       0.407       1.000    3.19%       1.500      1.800      2.000
             659.000        0.369      -0.249       4.100    0.15%       2.200      2.600
     TSBASE                36.711       0.258       0.000    3.08%      13.000     27.000     36.000
             682.000      594.554      -0.917      91.000    0.59%      41.000     60.000
     BASAGEC90              0.106       0.407      -3.917    0.81%      -2.833     -1.250     -0.792
             124.000        8.313      -1.090       6.417    0.81%       0.500      3.083
     SEX                    0.000      -1.426      -0.790   20.97%      -0.790      0.210      0.210
             124.000        0.166       0.035       0.210   79.03%       0.210      0.210
     YOL                    0.000       0.868      -3.387   20.97%      -3.387     -1.387     -1.387
             124.000        8.737      -0.315       5.613   17.74%       0.613      0.613


THE MODEL ESTIMATION TERMINATED NORMALLY

     USE THE FBITERATIONS OPTION TO INCREASE THE NUMBER OF ITERATIONS BY A FACTOR
     OF AT LEAST TWO TO CHECK CONVERGENCE AND THAT THE PSR VALUE DOES NOT INCREASE.



MODEL FIT INFORMATION

Number of Free Parameters                              24

Information Criteria

          Deviance (DIC)                             2290.946
          Estimated Number of Parameters (pD)         200.358



MODEL RESULTS

                                Posterior  One-Tailed         95% C.I.
                    Estimate       S.D.      P-Value   Lower 2.5%  Upper 2.5%  Significance

Within Level

Between Level

 PHYSCON  BY
    BASHKR             1.000       0.000      0.000       1.000       1.000
    BASSSSC            0.467       0.616      0.010       0.154       1.567      *

 BASTTDC    ON
    PHYSCON            0.627       0.914      0.010       0.233       1.984      *
    SLOPENA            2.707      18.650      0.442     -33.928      39.217
    LNVNA              2.042       1.348      0.044      -0.319       4.992

 BASTTDC    ON
    NA                -1.330       1.042      0.072      -3.657       0.444
    SEX                1.529       0.736      0.017       0.123       3.026      *
    YOL               -0.272       0.092      0.002      -0.452      -0.092      *
    BASAGEC90         -0.244       0.095      0.005      -0.430      -0.058      *

 NA       WITH
```

```
   SLOPENA           -0.003       0.002      0.044      -0.007       0.000
   LNVNA              0.187       0.074      0.003       0.056       0.348      *

 SLOPENA  WITH
   LNVNA             -0.001       0.002      0.360      -0.005       0.004

 Means
   NA                 2.111       0.061      0.000       1.993       2.233      *
   SLOPENA           -0.002       0.003      0.218      -0.008       0.003
   LNVNA             -2.581       0.117      0.000      -2.819      -2.362      *

 Intercepts
   BASTTDC           13.781       5.337      0.002       4.469      25.463      *
   BASHKR            18.484       0.673      0.000      17.157      19.804      *
   BASSSSC            1.328       0.150      0.000       1.032       1.624      *

 Variances
   NA                 0.349       0.062      0.000       0.250       0.491      *
   PHYSCON            6.008       5.563      0.000       0.537      21.183      *
   SLOPENA            0.000       0.000      0.000       0.000       0.001      *
   LNVNA              0.347       0.144      0.000       0.144       0.706      *

 Residual Variances
   BASTTDC            5.884       1.553      0.000       3.041       9.120      *
   BASHKR            43.421       7.425      0.000      30.021      59.330      *
   BASSSSC            1.366       0.620      0.000       0.112       2.446      *


STANDARDIZED MODEL RESULTS


STDYX Standardization

                                Posterior  One-Tailed         95% C.I.
                    Estimate       S.D.      P-Value   Lower 2.5%  Upper 2.5%  Significance

Within-Level Standardized Estimates Averaged Over Clusters

 SLOPENA | NA ON
   TSBASE            -0.009       0.025      0.350      -0.061       0.039

 LNVNA |
   NA                 0.852       0.014      0.000       0.823       0.879      *

Between Level

 PHYSCON  BY
   BASHKR             0.347       0.135      0.000       0.104       0.620      *
   BASSSSC            0.701       0.205      0.010       0.351       0.979      *

 BASTTDC    ON
   PHYSCON            0.467       0.161      0.010       0.215       0.734      *
   SLOPENA            0.018       0.123      0.442      -0.225       0.257
   LNVNA              0.367       0.219      0.044      -0.054       0.808

 BASTTDC    ON
   NA                -0.238       0.186      0.072      -0.652       0.078
   SEX                0.135       0.063      0.017       0.011       0.258      *
   YOL               -0.175       0.057      0.002      -0.284      -0.059      *
   BASAGEC90         -0.142       0.054      0.005      -0.246      -0.033      *

 NA       WITH
   SLOPENA           -0.239       0.134      0.044      -0.484       0.036
   LNVNA              0.553       0.157      0.003       0.185       0.801      *

 SLOPENA  WITH
   LNVNA             -0.059       0.158      0.360      -0.361       0.255

 Means
   NA                 3.576       0.315      0.000       2.995       4.229      *
   SLOPENA           -0.105       0.134      0.218      -0.367       0.158
   LNVNA             -4.389       0.912      0.000      -6.662      -3.099      *

 Intercepts
```

```
    BASTTDC            4.171       1.615      0.002       1.334       7.687      *
    BASHKR             2.605       0.202      0.000       2.215       3.005      *
    BASSSSC            0.807       0.106      0.000       0.601       1.015      *

 Variances
    NA                 1.000       0.000      0.000       1.000       1.000
    PHYSCON            1.000       0.000      0.000       1.000       1.000
    SLOPENA            1.000       0.000      0.000       1.000       1.000
    LNVNA              1.000       0.000      0.000       1.000       1.000

 Residual Variances
    BASTTDC            0.542       0.130      0.000       0.279       0.780      *
    BASHKR             0.880       0.101      0.000       0.616       0.989      *
    BASSSSC            0.507       0.222      0.000       0.042       0.855      *


STDY Standardization

                                Posterior  One-Tailed         95% C.I.
                    Estimate       S.D.      P-Value   Lower 2.5%  Upper 2.5%  Significance

Within-Level Standardized Estimates Averaged Over Clusters

 SLOPENA | NA ON
    TSBASE            -0.003       0.009      0.359      -0.021       0.014

 LNVNA |
    NA                 0.852       0.014      0.000       0.823       0.879      *

Between Level

 PHYSCON  BY
    BASHKR             0.347       0.135      0.000       0.104       0.620      *
    BASSSSC            0.701       0.205      0.010       0.351       0.979      *

 BASTTDC    ON
    PHYSCON            0.467       0.161      0.010       0.215       0.734      *
    SLOPENA            0.018       0.123      0.442      -0.225       0.257
    LNVNA              0.367       0.219      0.044      -0.054       0.808

 BASTTDC    ON
    NA                -0.238       0.186      0.072      -0.652       0.078
    SEX                0.464       0.216      0.017       0.037       0.885      *
    YOL               -0.082       0.027      0.002      -0.134      -0.028      *
    BASAGEC90         -0.074       0.028      0.005      -0.128      -0.017      *

 NA       WITH
    SLOPENA           -0.239       0.134      0.044      -0.484       0.036
    LNVNA              0.553       0.157      0.003       0.185       0.801      *

 SLOPENA  WITH
    LNVNA             -0.059       0.158      0.360      -0.361       0.255

 Means
    NA                 3.576       0.315      0.000       2.995       4.229      *
    SLOPENA           -0.105       0.134      0.218      -0.367       0.158
    LNVNA             -4.389       0.912      0.000      -6.662      -3.099      *

 Intercepts
    BASTTDC            4.171       1.615      0.002       1.334       7.687      *
    BASHKR             2.605       0.202      0.000       2.215       3.005      *
    BASSSSC            0.807       0.106      0.000       0.601       1.015      *

 Variances
    NA                 1.000       0.000      0.000       1.000       1.000
    PHYSCON            1.000       0.000      0.000       1.000       1.000
    SLOPENA            1.000       0.000      0.000       1.000       1.000
    LNVNA              1.000       0.000      0.000       1.000       1.000

 Residual Variances
    BASTTDC            0.542       0.130      0.000       0.279       0.780      *
    BASHKR             0.880       0.101      0.000       0.616       0.989      *
    BASSSSC            0.507       0.222      0.000       0.042       0.855      *
```

```
STD Standardization

                                Posterior  One-Tailed         95% C.I.
                    Estimate       S.D.      P-Value   Lower 2.5%  Upper 2.5%  Significance

Within-Level Standardized Estimates Averaged Over Clusters

 SLOPENA | NA ON
    TSBASE           -0.001       0.002      0.282      -0.006       0.003

 LNVNA |
    NA                0.086       0.008      0.000       0.074       0.105      *

Between Level

 PHYSCON  BY
    BASHKR            2.451       1.009      0.000       0.733       4.603      *
    BASSSSC           1.156       0.353      0.010       0.562       1.674      *

 BASTTDC    ON
    PHYSCON           1.542       0.556      0.010       0.697       2.526      *
    SLOPENA           0.059       0.412      0.442      -0.750       0.867
    LNVNA             1.212       0.732      0.044      -0.181       2.700

 BASTTDC    ON
    NA               -1.330       1.042      0.072      -3.657       0.444
    SEX               1.529       0.736      0.017       0.123       3.026      *
    YOL              -0.272       0.092      0.002      -0.452      -0.092      *
    BASAGEC90        -0.244       0.095      0.005      -0.430      -0.058      *

 NA       WITH
    SLOPENA          -0.140       0.085      0.044      -0.313       0.020
    LNVNA             0.324       0.100      0.003       0.108       0.504      *

 SLOPENA  WITH
    LNVNA            -0.059       0.158      0.360      -0.361       0.255

 Means
    NA                2.111       0.061      0.000       1.993       2.233      *
    SLOPENA          -0.105       0.134      0.218      -0.367       0.158
    LNVNA            -4.389       0.912      0.000      -6.662      -3.099      *

 Intercepts
    BASTTDC          13.781       5.337      0.002       4.469      25.463      *
    BASHKR           18.484       0.673      0.000      17.157      19.804      *
    BASSSSC           1.328       0.150      0.000       1.032       1.624      *

 Variances
    NA                0.349       0.062      0.000       0.250       0.491      *
    PHYSCON           1.000       0.000      0.000       1.000       1.000
    SLOPENA           1.000       0.000      0.000       1.000       1.000
    LNVNA             1.000       0.000      0.000       1.000       1.000

 Residual Variances
    BASTTDC           5.884       1.553      0.000       3.041       9.120      *
    BASHKR           43.421       7.425      0.000      30.021      59.330      *
    BASSSSC           1.366       0.620      0.000       0.112       2.446      *


R-SQUARE

Within-Level R-Square Averaged Across Clusters

                                Posterior  One-Tailed         95% C.I.
    Variable        Estimate       S.D.      P-Value   Lower 2.5%  Upper 2.5%

    NA                0.148       0.014      0.000       0.121       0.177

Between Level

                                Posterior  One-Tailed         95% C.I.
    Variable        Estimate       S.D.      P-Value   Lower 2.5%  Upper 2.5%
```

BASTTDC	0.458	0.130	0.000	0.220	0.721
BASHKR	0.120	0.101	0.000	0.011	0.384
BASSSSC	0.493	0.222	0.000	0.145	0.958

TECHNICAL 8 OUTPUT

TECHNICAL 8 OUTPUT FOR BAYES ESTIMATION

CHAIN	BSEED
1	0
2	285380

ITERATION	POTENTIAL SCALE REDUCTION	PARAMETER WITH HIGHEST PSR
100	2.794	23
200	1.748	8
300	1.451	20
400	1.407	17
500	1.584	10
600	1.553	10
700	1.512	10
800	1.317	17
900	1.253	17
1000	1.194	7
1100	1.189	7
1200	1.103	7
1300	1.101	7
1400	1.132	3
1500	1.242	5
1600	1.244	5
1700	1.265	5
1800	1.222	5
1900	1.242	5
2000	1.218	5
2100	1.158	5
2200	1.124	5
2300	1.156	5
2400	1.158	5
2500	1.124	5
2600	1.089	12
2700	1.050	5
2800	1.023	5
2900	1.021	10
3000	1.034	20
3100	1.043	20
3200	1.047	7
3300	1.033	7
3400	1.028	7
3500	1.027	7
3600	1.025	7
3700	1.027	7
3800	1.027	7
3900	1.027	7
4000	1.022	7
4100	1.012	10
4200	1.026	10
4300	1.030	3
4400	1.039	3
4500	1.047	3
4600	1.050	3
4700	1.045	3
4800	1.047	3
4900	1.049	3
5000	1.042	3
5100	1.038	3
5200	1.038	3
5300	1.040	10
5400	1.040	10
5500	1.021	10
5600	1.013	7

```
5700            1.013           7
5800            1.021           3
5900            1.038           3
6000            1.048           3
6100            1.056           3
6200            1.057           3
6300            1.058           3
6400            1.059           3
6500            1.060           3
6600            1.060           3
6700            1.058           3
6800            1.059           3
6900            1.059           3
7000            1.061           3
7100            1.052           3
7200            1.037           3
7300            1.027           3
7400            1.030           3
7500            1.033           3
7600            1.038           3
7700            1.038           3
7800            1.035           3
7900            1.035           3
8000            1.041           3
8100            1.053           3
8200            1.064           3
8300            1.066           3
8400            1.068           3
8500            1.065           3
8600            1.068           3
8700            1.068           3
8800            1.067           3
8900            1.067           3
9000            1.069           3
9100            1.068           3
9200            1.067           3
9300            1.065           3
9400            1.065           3
9500            1.066           3
9600            1.064           3
9700            1.063           3
9800            1.055           3
9900            1.046           3
10000           1.039           3
10100           1.037           3
10200           1.035           3
10300           1.034           3
10400           1.035           20
10500           1.038           20
10600           1.038           20
10700           1.033           3
10800           1.036           3
10900           1.034           3
11000           1.032           3
11100           1.029           3
11200           1.022           3
11300           1.015           3
11400           1.013           8
11500           1.015           8
11600           1.019           8
11700           1.020           8
11800           1.017           8
11900           1.016           8
12000           1.019           13
12100           1.016           13
12200           1.018           7
12300           1.019           7
12400           1.022           7
12500           1.025           7
12600           1.024           7
12700           1.023           7
12800           1.023           7
12900           1.021           7
13000           1.019           7
```

13100	1.017	7
13200	1.014	7
13300	1.010	7
13400	1.009	7
13500	1.009	20
13600	1.011	20
13700	1.015	20
13800	1.017	20
13900	1.016	20
14000	1.013	20
14100	1.011	20
14200	1.011	20
14300	1.010	20
14400	1.012	20
14500	1.013	20
14600	1.019	20
14700	1.021	20
14800	1.018	20
14900	1.019	20
15000	1.018	20
15100	1.020	20
15200	1.021	20
15300	1.022	20
15400	1.023	20
15500	1.023	20
15600	1.021	20
15700	1.017	20
15800	1.014	20
15900	1.014	20
16000	1.014	20
16100	1.015	20
16200	1.016	20
16300	1.016	20
16400	1.014	20
16500	1.012	20
16600	1.010	20
16700	1.011	20
16800	1.011	20
16900	1.011	20
17000	1.011	20
17100	1.010	20
17200	1.011	20
17300	1.013	17
17400	1.014	20
17500	1.014	20
17600	1.013	20
17700	1.013	20
17800	1.013	20
17900	1.013	20
18000	1.019	10
18100	1.022	10
18200	1.027	10
18300	1.032	10
18400	1.037	10
18500	1.042	10
18600	1.047	10
18700	1.045	10
18800	1.044	3
18900	1.040	10
19000	1.028	3
19100	1.030	20
19200	1.032	20
19300	1.035	20
19400	1.035	7
19500	1.036	20
19600	1.036	20
19700	1.038	20
19800	1.038	20
19900	1.038	20
20000	1.041	3
20100	1.045	3
20200	1.049	3
20300	1.051	3
20400	1.052	3

20500	1.052	3
20600	1.052	3
20700	1.057	20
20800	1.060	20
20900	1.061	20
21000	1.060	20
21100	1.064	20
21200	1.064	20
21300	1.063	20
21400	1.060	20
21500	1.055	3
21600	1.060	3
21700	1.063	3
21800	1.064	3
21900	1.064	3
22000	1.064	3
22100	1.064	3
22200	1.066	17
22300	1.072	17
22400	1.077	17
22500	1.080	17
22600	1.083	17
22700	1.085	17
22800	1.087	17
22900	1.092	17
23000	1.095	17
23100	1.101	17
23200	1.103	17
23300	1.105	17
23400	1.105	17
23500	1.103	17
23600	1.098	17
23700	1.098	17
23800	1.099	17
23900	1.107	17
24000	1.108	17
24100	1.102	17
24200	1.095	17
24300	1.092	17
24400	1.091	17
24500	1.085	17
24600	1.085	17
24700	1.080	17
24800	1.076	17
24900	1.077	17
25000	1.079	17
25100	1.083	17
25200	1.087	17
25300	1.087	17
25400	1.085	17
25500	1.085	17
25600	1.087	17
25700	1.089	17
25800	1.096	17
25900	1.102	17
26000	1.107	17
26100	1.105	17
26200	1.104	17
26300	1.099	17
26400	1.090	17
26500	1.086	17
26600	1.085	17
26700	1.082	17
26800	1.082	17
26900	1.083	17
27000	1.081	17
27100	1.080	17
27200	1.080	17
27300	1.080	17
27400	1.077	17
27500	1.074	17
27600	1.074	17
27700	1.073	17
27800	1.070	17

27900	1.066	17
28000	1.065	17
28100	1.062	17
28200	1.061	17
28300	1.059	17
28400	1.056	17
28500	1.057	17
28600	1.058	17
28700	1.057	17
28800	1.055	17
28900	1.054	17
29000	1.052	17
29100	1.048	3
29200	1.048	3
29300	1.048	3
29400	1.047	3
29500	1.047	3
29600	1.047	3
29700	1.047	3
29800	1.047	3
29900	1.047	3
30000	1.046	3
30100	1.046	3
30200	1.046	3
30300	1.046	3
30400	1.046	3
30500	1.046	3
30600	1.046	3
30700	1.045	3
30800	1.045	3
30900	1.045	3
31000	1.045	3
31100	1.045	3
31200	1.045	3
31300	1.044	3
31400	1.045	17
31500	1.045	17
31600	1.044	3
31700	1.045	17
31800	1.044	3
31900	1.044	3
32000	1.043	3
32100	1.043	3
32200	1.043	3
32300	1.043	3
32400	1.043	3
32500	1.043	3
32600	1.043	3
32700	1.042	3
32800	1.042	3
32900	1.042	3
33000	1.042	3
33100	1.042	3
33200	1.042	3
33300	1.042	3
33400	1.041	3
33500	1.041	3
33600	1.041	3
33700	1.041	3
33800	1.041	3
33900	1.041	3
34000	1.041	3
34100	1.041	3
34200	1.040	3
34300	1.040	3
34400	1.040	3
34500	1.040	3
34600	1.040	3
34700	1.040	3
34800	1.040	3
34900	1.040	3
35000	1.039	3
35100	1.039	3
35200	1.039	3

```
35300            1.039            3
35400            1.039            3
35500            1.039            3
35600            1.039            3
35700            1.039            3
35800            1.038            3
35900            1.038            3
36000            1.038            3
36100            1.038            3
36200            1.038            3
36300            1.038            3
36400            1.038            3
36500            1.037            3
36600            1.037            3
36700            1.037            3
36800            1.037            3
36900            1.037            3
37000            1.037            3
37100            1.036            3
37200            1.036            3
37300            1.036            3
37400            1.036            3
37500            1.036            3
37600            1.036            3
37700            1.035            3
37800            1.035            3
37900            1.035            3
38000            1.034            3
38100            1.034            3
38200            1.033            3
38300            1.032            3
38400            1.032            3
38500            1.031            3
38600            1.030            3
38700            1.028            3
38800            1.027            3
38900            1.025            3
39000            1.024            3
39100            1.023            3
39200            1.022            3
39300            1.020            3
39400            1.019            3
39500            1.019            3
39600            1.020            3
39700            1.021            3
39800            1.021            3
39900            1.020            3
40000            1.021            10
40100            1.020            10
40200            1.019            3
40300            1.018            10
40400            1.017            10
40500            1.015            10
40600            1.015            10
40700            1.014            10
40800            1.013            3
40900            1.013            3
41000            1.013            3
41100            1.012            3
41200            1.012            3
41300            1.011            3
41400            1.011            3
41500            1.011            3
41600            1.010            3
41700            1.010            3
41800            1.010            3
41900            1.010            3
42000            1.010            3
42100            1.010            3
42200            1.009            3
42300            1.009            3
42400            1.009            3
42500            1.009            3
42600            1.009            3
```

42700	1.009	3
42800	1.008	3
42900	1.008	17
43000	1.008	17
43100	1.008	17
43200	1.008	17
43300	1.008	17
43400	1.009	17
43500	1.009	3
43600	1.009	17
43700	1.010	17
43800	1.011	17
43900	1.011	17
44000	1.011	17
44100	1.010	17
44200	1.009	17
44300	1.009	17
44400	1.009	17
44500	1.009	17
44600	1.008	17
44700	1.007	17
44800	1.007	17
44900	1.007	17
45000	1.007	17
45100	1.007	17
45200	1.008	17
45300	1.009	17
45400	1.009	17
45500	1.014	3
45600	1.017	3
45700	1.019	3
45800	1.021	3
45900	1.022	3
46000	1.023	3
46100	1.024	3
46200	1.024	3
46300	1.025	3
46400	1.025	3
46500	1.025	3
46600	1.026	3
46700	1.026	3
46800	1.026	3
46900	1.026	3
47000	1.026	3
47100	1.026	3
47200	1.026	3
47300	1.026	3
47400	1.026	3
47500	1.026	3
47600	1.026	3
47700	1.026	3
47800	1.026	3
47900	1.026	3
48000	1.026	3
48100	1.026	3
48200	1.026	3
48300	1.026	3
48400	1.026	3
48500	1.026	3
48600	1.025	3
48700	1.025	3
48800	1.025	3
48900	1.025	3
49000	1.025	3
49100	1.025	3
49200	1.025	3
49300	1.026	3
49400	1.026	3
49500	1.027	3
49600	1.028	3
49700	1.028	3
49800	1.028	3
49900	1.028	3
50000	1.028	3

50100	1.028	3
50200	1.028	3
50300	1.028	3
50400	1.029	17
50500	1.030	17
50600	1.031	17
50700	1.033	17
50800	1.033	17
50900	1.033	17
51000	1.033	17
51100	1.033	17
51200	1.033	17
51300	1.033	17
51400	1.032	3
51500	1.032	3
51600	1.032	3
51700	1.032	17
51800	1.034	17
51900	1.036	17
52000	1.037	17
52100	1.038	17
52200	1.036	17
52300	1.035	17
52400	1.035	17
52500	1.036	17
52600	1.034	17
52700	1.032	17
52800	1.032	17
52900	1.032	17
53000	1.031	3
53100	1.031	3
53200	1.030	3
53300	1.030	3
53400	1.030	3
53500	1.030	3
53600	1.030	3
53700	1.030	3
53800	1.030	3
53900	1.029	3
54000	1.029	3
54100	1.029	3
54200	1.028	3
54300	1.028	3
54400	1.028	3
54500	1.027	3
54600	1.028	3
54700	1.028	3
54800	1.028	3
54900	1.028	3
55000	1.028	3
55100	1.027	3
55200	1.027	3
55300	1.027	3
55400	1.026	3
55500	1.025	3
55600	1.025	3
55700	1.025	3
55800	1.024	3
55900	1.024	3
56000	1.024	3
56100	1.024	3
56200	1.024	3
56300	1.024	3
56400	1.023	3
56500	1.023	3
56600	1.023	3
56700	1.023	3
56800	1.023	3
56900	1.023	3
57000	1.022	3
57100	1.022	3
57200	1.022	3
57300	1.022	3
57400	1.021	3

```
57500          1.021          3
57600          1.021          3
57700          1.021          3
57800          1.022          3
57900          1.022          3
58000          1.022          3
58100          1.021          3
58200          1.021          3
58300          1.021          3
58400          1.021          3
58500          1.021          3
58600          1.021          3
58700          1.021          3
58800          1.021          3
58900          1.020          3
59000          1.020          3
59100          1.020          3
59200          1.021          3
59300          1.020          3
59400          1.020          3
59500          1.020          3
59600          1.020          3
59700          1.020          3
59800          1.020          3
59900          1.020          3
60000          1.020          3
60100          1.020          3
60200          1.020          3
60300          1.020          3
60400          1.020          3
60500          1.020          3
60600          1.020          3
60700          1.020          3
60800          1.020          3
60900          1.020          3
61000          1.020          3
61100          1.020          3
61200          1.020          3
61300          1.020          3
61400          1.020          3
61500          1.020          3
61600          1.019          3
61700          1.019          3
61800          1.019          3
61900          1.019          3
62000          1.019          3
62100          1.019          3
62200          1.019          3
62300          1.019          3
62400          1.019          3
62500          1.019          3
62600          1.019          3
62700          1.019          3
62800          1.019          3
62900          1.019          3
63000          1.019          3
63100          1.019          3
63200          1.019          3
63300          1.019          3
63400          1.019          3
63500          1.020          3
63600          1.019          3
63700          1.019          3
63800          1.019          3
63900          1.019          3
64000          1.019          3
64100          1.019          3
64200          1.018          3
64300          1.018          3
64400          1.018          3
64500          1.018          3
64600          1.018          3
64700          1.018          3
64800          1.018          3
```

64900	1.018	3
65000	1.018	3
65100	1.018	3
65200	1.018	3
65300	1.018	3
65400	1.018	3
65500	1.018	3
65600	1.018	3
65700	1.018	3
65800	1.018	3
65900	1.018	3
66000	1.018	3
66100	1.018	3
66200	1.018	3
66300	1.018	3
66400	1.018	3
66500	1.019	3
66600	1.019	3
66700	1.020	3
66800	1.022	3
66900	1.022	3
67000	1.022	3
67100	1.022	3
67200	1.022	3
67300	1.022	3
67400	1.022	3
67500	1.022	3
67600	1.022	3
67700	1.022	3
67800	1.022	3
67900	1.022	3
68000	1.022	3
68100	1.021	3
68200	1.021	3
68300	1.021	3
68400	1.021	3
68500	1.021	3
68600	1.021	3
68700	1.021	3
68800	1.021	3
68900	1.021	3
69000	1.021	3
69100	1.022	3
69200	1.022	3
69300	1.022	3
69400	1.022	3
69500	1.022	3
69600	1.022	3
69700	1.022	3
69800	1.022	3
69900	1.022	3
70000	1.022	3
70100	1.022	3
70200	1.022	3
70300	1.021	3
70400	1.021	3
70500	1.021	3
70600	1.021	3
70700	1.021	3
70800	1.021	3
70900	1.021	3
71000	1.022	3
71100	1.022	3
71200	1.021	3
71300	1.022	3
71400	1.021	3
71500	1.021	3
71600	1.021	10
71700	1.021	10
71800	1.021	10
71900	1.021	10
72000	1.021	10
72100	1.020	10
72200	1.020	10

72300	1.020	10
72400	1.020	10
72500	1.019	10
72600	1.019	10
72700	1.019	10
72800	1.018	10
72900	1.018	10
73000	1.018	10
73100	1.017	10
73200	1.017	10
73300	1.016	10
73400	1.016	10
73500	1.015	10
73600	1.014	10
73700	1.014	10
73800	1.013	10
73900	1.013	10
74000	1.013	10
74100	1.014	10
74200	1.013	10
74300	1.013	10
74400	1.012	10
74500	1.011	10
74600	1.011	10
74700	1.010	10
74800	1.010	10
74900	1.010	10
75000	1.010	10
75100	1.010	10
75200	1.010	10
75300	1.010	10
75400	1.010	10
75500	1.010	10
75600	1.010	10
75700	1.011	3
75800	1.012	3
75900	1.013	3
76000	1.013	3
76100	1.013	3
76200	1.013	3
76300	1.013	3
76400	1.013	3
76500	1.013	3
76600	1.014	3
76700	1.014	3
76800	1.015	3
76900	1.015	3
77000	1.015	3
77100	1.016	3
77200	1.016	3
77300	1.016	3
77400	1.016	3
77500	1.016	3
77600	1.016	3
77700	1.016	3
77800	1.016	3
77900	1.016	3
78000	1.016	3
78100	1.016	3
78200	1.015	3
78300	1.015	3
78400	1.015	3
78500	1.015	3
78600	1.015	3
78700	1.014	3
78800	1.014	3
78900	1.013	3
79000	1.012	10
79100	1.011	10
79200	1.011	10
79300	1.011	10
79400	1.011	10
79500	1.011	10
79600	1.011	10

```
     79700             1.011              10
     79800             1.011              10
     79900             1.011              10
     80000             1.011              10
     80100             1.011              10
     80200             1.011              10
     80300             1.011              10
     80400             1.011              10
     80500             1.011              10
     80600             1.011              10
     80700             1.011              10
     80800             1.011              10
     80900             1.011              10
     81000             1.011              10
     81100             1.011              10
     81200             1.011              10
     81300             1.011              10
     81400             1.011              10
     81500             1.011              10
     81600             1.011              10
     81700             1.011              10
     81800             1.011              10
     81900             1.011              10
     82000             1.011              10
     82100             1.012              10
     82200             1.012              10
     82300             1.011              10
     82400             1.011              10
     82500             1.011              10
     82600             1.011              10
     82700             1.011              10
     82800             1.011              10
     82900             1.011              10
     83000             1.011              10
     83100             1.011              10
     83200             1.011              10
     83300             1.011              10
     83400             1.011              10
     83500             1.011              10
     83600             1.011              10
     83700             1.011              10
     83800             1.011              10
     83900             1.011              10
     84000             1.011              10
     84100             1.010              10
     84200             1.010              10
     84300             1.010              10
     84400             1.010              10
     84500             1.010              10
     84600             1.010              10
     84700             1.010              10
     84800             1.010              10
     84900             1.010              10
     85000             1.010              10
     85100             1.010              10
     85200             1.010              10
     85300             1.010              10
     85400             1.010              10
     85500             1.010              10
     85600             1.010              10
     85700             1.010              10
     85800             1.010              10
     85900             1.010              10
     86000             1.010              10
     86100             1.010              10
     86200             1.010              10
     86300             1.010              10
     86400             1.010              10
     86500             1.010              10
     86600             1.010              10
     86700             1.010              10
     86800             1.009              10
     86900             1.009              10
     87000             1.009              10
```

87100	1.009	10
87200	1.009	10
87300	1.009	10
87400	1.009	10
87500	1.009	10
87600	1.009	10
87700	1.009	10
87800	1.009	10
87900	1.009	10
88000	1.008	10
88100	1.008	10
88200	1.008	10
88300	1.008	10
88400	1.008	10
88500	1.008	10
88600	1.008	10
88700	1.008	10
88800	1.008	10
88900	1.008	10
89000	1.008	10
89100	1.009	10
89200	1.009	10
89300	1.009	10
89400	1.009	10
89500	1.009	10
89600	1.009	10
89700	1.009	10
89800	1.008	10
89900	1.008	10
90000	1.009	10
90100	1.008	10
90200	1.008	10
90300	1.008	10
90400	1.008	10
90500	1.008	10
90600	1.008	10
90700	1.008	10
90800	1.008	10
90900	1.008	10
91000	1.008	10
91100	1.007	10
91200	1.008	10
91300	1.008	10
91400	1.008	10
91500	1.007	10
91600	1.007	10
91700	1.007	10
91800	1.007	10
91900	1.007	10
92000	1.007	10
92100	1.007	10
92200	1.007	10
92300	1.007	10
92400	1.007	10
92500	1.007	10
92600	1.007	10
92700	1.007	10
92800	1.007	10
92900	1.007	10
93000	1.007	10
93100	1.007	10
93200	1.007	10
93300	1.007	10
93400	1.006	10
93500	1.006	10
93600	1.006	10
93700	1.006	10
93800	1.006	10
93900	1.007	10
94000	1.007	10
94100	1.007	10
94200	1.007	10
94300	1.007	10
94400	1.006	10

94500	1.006	10
94600	1.007	10
94700	1.007	10
94800	1.007	10
94900	1.007	10
95000	1.007	10
95100	1.006	10
95200	1.006	10
95300	1.006	10
95400	1.006	10
95500	1.006	10
95600	1.006	10
95700	1.006	10
95800	1.006	10
95900	1.006	10
96000	1.006	10
96100	1.006	10
96200	1.006	10
96300	1.006	10
96400	1.005	10
96500	1.005	10
96600	1.005	10
96700	1.005	10
96800	1.005	10
96900	1.005	10
97000	1.005	10
97100	1.005	10
97200	1.005	10
97300	1.005	10
97400	1.005	10
97500	1.005	10
97600	1.005	10
97700	1.005	10
97800	1.005	10
97900	1.005	10
98000	1.005	10
98100	1.005	10
98200	1.005	10
98300	1.005	10
98400	1.005	10
98500	1.005	10
98600	1.005	10
98700	1.005	10
98800	1.004	10
98900	1.004	10
99000	1.004	5
99100	1.004	5
99200	1.004	5
99300	1.004	5
99400	1.004	5
99500	1.004	5
99600	1.004	5
99700	1.004	5
99800	1.004	5
99900	1.005	5
100000	1.005	5
100100	1.005	5
100200	1.005	5
100300	1.005	5
100400	1.005	5
100500	1.005	5
100600	1.005	5
100700	1.005	5
100800	1.005	5
100900	1.005	5
101000	1.005	5
101100	1.005	5
101200	1.006	5
101300	1.006	5
101400	1.006	5
101500	1.006	5
101600	1.006	5
101700	1.006	3
101800	1.006	3

101900	1.006	3
102000	1.007	3
102100	1.007	3
102200	1.007	3
102300	1.007	3
102400	1.007	3
102500	1.007	3
102600	1.007	3
102700	1.007	3
102800	1.006	3
102900	1.006	3
103000	1.006	3
103100	1.006	3
103200	1.006	3
103300	1.006	3
103400	1.006	3
103500	1.006	3
103600	1.006	3
103700	1.006	3
103800	1.006	3
103900	1.006	3
104000	1.006	3
104100	1.006	3
104200	1.006	3
104300	1.006	3
104400	1.006	3
104500	1.006	3
104600	1.006	3
104700	1.006	3
104800	1.006	3
104900	1.007	3
105000	1.008	3
105100	1.008	3
105200	1.008	3
105300	1.008	3
105400	1.008	3
105500	1.008	3
105600	1.008	3
105700	1.008	3
105800	1.008	3
105900	1.008	3
106000	1.008	3
106100	1.008	3
106200	1.008	3
106300	1.008	3
106400	1.008	3
106500	1.008	3
106600	1.008	3
106700	1.008	3
106800	1.008	3
106900	1.008	3
107000	1.009	3
107100	1.009	3
107200	1.009	3
107300	1.008	3
107400	1.008	3
107500	1.008	3
107600	1.008	3
107700	1.008	3
107800	1.008	3
107900	1.008	3
108000	1.008	3
108100	1.008	3
108200	1.007	3
108300	1.007	3
108400	1.007	3
108500	1.007	3
108600	1.007	3
108700	1.007	3
108800	1.006	3
108900	1.006	3
109000	1.006	3
109100	1.006	3
109200	1.007	3

```
109300           1.007              3
109400           1.007              3
109500           1.007              3
109600           1.007              3
109700           1.007              3
109800           1.007              3
109900           1.007              3
110000           1.007              3
110100           1.007              3
110200           1.007              3
110300           1.007              3
110400           1.007              3
110500           1.007              3
110600           1.007              3
110700           1.007              3
110800           1.006              3
110900           1.006              3
111000           1.007              3
111100           1.007              3
111200           1.007              3
111300           1.007              3
111400           1.007              3
111500           1.007              3
111600           1.007              3
111700           1.007              3
111800           1.007              3
111900           1.007              3
112000           1.007              3
112100           1.007              3
112200           1.007              3
112300           1.007              3
112400           1.007              3
112500           1.007              3
112600           1.007              3
112700           1.007              3
112800           1.007              3
112900           1.007              3
113000           1.007              3
113100           1.007              3
113200           1.007              3
113300           1.007              3
113400           1.007              3
113500           1.007              3
113600           1.007              3
113700           1.007              3
113800           1.007              3
113900           1.007              3
114000           1.007              3
114100           1.007              3
114200           1.007              3
114300           1.007              3
114400           1.007              3
114500           1.007              3
114600           1.007              3
114700           1.006              3
114800           1.006              3
114900           1.006              3
115000           1.006              3
115100           1.006              3
115200           1.007              3
115300           1.007              3
115400           1.007              3
115500           1.007              3
115600           1.007              3
115700           1.007              3
115800           1.007              3
115900           1.007              3
116000           1.007              3
116100           1.007              3
116200           1.007              3
116300           1.007              3
116400           1.007              3
116500           1.007              3
116600           1.007              3
```

```
     116700          1.007             3
     116800          1.007             3
     116900          1.007             3
     117000          1.007             3
     117100          1.007             3
     117200          1.007             3
     117300          1.007             3
     117400          1.007             3
     117500          1.007             3
     117600          1.007             3
     117700          1.007             3
     117800          1.007             3
     117900          1.007             3
     118000          1.007             3
     118100          1.007             3
     118200          1.007             3
     118300          1.007             3
     118400          1.007             3
     118500          1.007             3
     118600          1.007             3
     118700          1.007             3
     118800          1.007             3
     118900          1.007             3
     119000          1.007             3
     119100          1.007             3
     119200          1.007             3
     119300          1.007             3
     119400          1.007             3
     119500          1.007             3
     119600          1.007             3
     119700          1.007             3
     119800          1.007             3
     119900          1.007             3
     120000          1.007             3
     120100          1.007             3
     120200          1.007             3
     120300          1.007             3
     120400          1.007             3
     120500          1.006             3
     120600          1.006             3
     120700          1.006             3
     120800          1.006             3
     120900          1.006             3
     121000          1.006             3
     121100          1.006             3
     121200          1.006             3
     121300          1.006             3
     121400          1.006             3
     121500          1.006             3
     121600          1.006             3
     121700          1.006             3
     121800          1.006             3
     121900          1.006             3
     122000          1.006             3
     122100          1.006             3
     122200          1.006             3
     122300          1.006             3
     122400          1.006             3
     122500          1.006             3
     122600          1.006             3
     122700          1.006             3
     122800          1.006             3
     122900          1.006             3
     123000          1.006             3
     123100          1.006             3
     123200          1.006             3
     123300          1.006             3
     123400          1.006             3
     123500          1.006             3
     123600          1.006             3
     123700          1.006             3
     123800          1.006             3
     123900          1.006             3
     124000          1.006             3
```

```
124100          1.006           3
124200          1.005           3
124300          1.005           3
124400          1.005           3
124500          1.005           3
124600          1.005           3
124700          1.005           3
124800          1.005           3
124900          1.005           3
125000          1.005           3
125100          1.005           3
125200          1.005           3
125300          1.005           3
125400          1.005           3
125500          1.005           3
125600          1.005           3
125700          1.005           3
125800          1.005           3
125900          1.005           3
126000          1.005           3
126100          1.005           3
126200          1.005           3
126300          1.005           3
126400          1.005           3
126500          1.005           3
126600          1.005           3
126700          1.005           3
126800          1.005           3
126900          1.005           3
127000          1.005           3
127100          1.005           3
127200          1.005           3
127300          1.005           3
127400          1.005           3
127500          1.006           3
127600          1.006           3
127700          1.006           3
127800          1.006           3
127900          1.006           3
128000          1.006           3
128100          1.005           3
128200          1.005           3
128300          1.005           3
128400          1.005           3
128500          1.005           3
128600          1.005           3
128700          1.005           3
128800          1.005           3
128900          1.005           3
129000          1.005           3
129100          1.005           3
129200          1.005           3
129300          1.005           3
129400          1.006           3
129500          1.006           3
129600          1.006           3
129700          1.005           3
129800          1.005           3
129900          1.005           3
130000          1.005           3
130100          1.005           3
130200          1.005           3
130300          1.005           3
130400          1.005           3
130500          1.005           3
130600          1.005           3
130700          1.006           3
130800          1.006           3
130900          1.006           3
131000          1.006           3
131100          1.006           3
131200          1.006           3
131300          1.006           3
131400          1.006           3
```

```
     131500          1.006             3
     131600          1.006             3
     131700          1.006             3
     131800          1.006             3
     131900          1.006             3
     132000          1.006             3
     132100          1.006             3
     132200          1.006             3
     132300          1.006             3
     132400          1.006             3
     132500          1.006             3
     132600          1.006             3
     132700          1.006             3
     132800          1.006             3
     132900          1.006             3
     133000          1.006             3
     133100          1.007             3
     133200          1.007             3
     133300          1.008             3
     133400          1.008             3
     133500          1.009             3
     133600          1.010             3
     133700          1.011             3
     133800          1.011             3
     133900          1.012             3
     134000          1.012             3
     134100          1.012             3
     134200          1.012             3
     134300          1.012             3
     134400          1.012             3
     134500          1.012             3
     134600          1.012             3
     134700          1.012             3
     134800          1.012             3
     134900          1.012             3
     135000          1.012             3
     135100          1.012             3
     135200          1.012             3
     135300          1.012             3
     135400          1.012             3
     135500          1.012             3
     135600          1.011             3
     135700          1.011             3
     135800          1.011             3
     135900          1.011             3
     136000          1.011             3
     136100          1.011             3
     136200          1.011             3
     136300          1.011             3
     136400          1.011             3
     136500          1.011             3
     136600          1.011             3
     136700          1.011             3
     136800          1.011             3
     136900          1.011             3
     137000          1.011             3
     137100          1.011             3
     137200          1.011             3
     137300          1.011             3
     137400          1.011             3
     137500          1.011             3
     137600          1.011             3
     137700          1.010             3
     137800          1.010             3
     137900          1.010             3
     138000          1.010             3
     138100          1.010             3
     138200          1.010             3
     138300          1.010             3
     138400          1.010             3
     138500          1.010             3
     138600          1.010             10
     138700          1.009             10
     138800          1.009             10
```

138900	1.009	10
139000	1.009	10
139100	1.009	10
139200	1.009	10
139300	1.009	10
139400	1.009	10
139500	1.009	10
139600	1.009	10
139700	1.009	10
139800	1.009	10
139900	1.009	10
140000	1.009	10
140100	1.009	10
140200	1.009	10
140300	1.009	10
140400	1.009	10
140500	1.009	10
140600	1.009	10
140700	1.009	10
140800	1.009	10
140900	1.009	10
141000	1.009	10
141100	1.009	10
141200	1.009	10
141300	1.008	10
141400	1.008	10
141500	1.008	10
141600	1.008	10
141700	1.008	10
141800	1.008	10
141900	1.008	10
142000	1.009	10
142100	1.009	10
142200	1.009	10
142300	1.009	10
142400	1.009	10
142500	1.009	10
142600	1.009	10
142700	1.009	10
142800	1.009	10
142900	1.009	10
143000	1.009	10
143100	1.009	10
143200	1.009	10
143300	1.009	10
143400	1.009	10
143500	1.009	10
143600	1.009	10
143700	1.009	10
143800	1.009	10
143900	1.009	10
144000	1.009	10
144100	1.009	10
144200	1.009	10
144300	1.009	10
144400	1.009	10
144500	1.009	10
144600	1.009	10
144700	1.009	10
144800	1.009	10
144900	1.009	10
145000	1.009	10
145100	1.009	10
145200	1.009	10
145300	1.009	10
145400	1.009	10
145500	1.008	10
145600	1.008	10
145700	1.008	10
145800	1.008	10
145900	1.008	10
146000	1.008	10
146100	1.008	10
146200	1.008	10

```
146300          1.008          10
146400          1.008          10
146500          1.008          10
146600          1.008          10
146700          1.008          10
146800          1.008          10
146900          1.008          10
147000          1.008          10
147100          1.008          10
147200          1.008          10
147300          1.008          10
147400          1.008          10
147500          1.008          10
147600          1.008          10
147700          1.008          10
147800          1.008          10
147900          1.008          10
148000          1.008          10
148100          1.008          10
148200          1.008          10
148300          1.008          10
148400          1.008          10
148500          1.008          10
148600          1.008          10
148700          1.008          10
148800          1.008          10
148900          1.008          10
149000          1.008          10
149100          1.008          10
149200          1.008          10
149300          1.008          10
149400          1.008          10
149500          1.008          10
149600          1.008          10
149700          1.008          10
149800          1.008          10
149900          1.008          10
150000          1.008          10
150100          1.008          10
150200          1.008          10
150300          1.008          10
150400          1.008          10
150500          1.008          10
150600          1.008          10
150700          1.008          10
150800          1.008          10
150900          1.008          10
151000          1.008          10
151100          1.008          10
151200          1.008          10
151300          1.008          10
151400          1.008          10
151500          1.009          10
151600          1.009          10
151700          1.010          10
151800          1.010          10
151900          1.010          10
152000          1.010          10
152100          1.010          10
152200          1.010          3
152300          1.010          3
152400          1.010          3
152500          1.010          3
152600          1.010          3
152700          1.010          3
152800          1.010          3
152900          1.010          3
153000          1.010          3
153100          1.011          3
153200          1.011          3
153300          1.011          3
153400          1.011          3
153500          1.011          3
153600          1.011          3
```

```
153700           1.011            3
153800           1.012            3
153900           1.012            3
154000           1.012            3
154100           1.012            3
154200           1.012            3
154300           1.012            3
154400           1.012            3
154500           1.012            3
154600           1.012            3
154700           1.012            3
154800           1.012            3
154900           1.012            3
155000           1.012            3
155100           1.012            3
155200           1.012            3
155300           1.012            3
155400           1.012            3
155500           1.012            3
155600           1.012            3
155700           1.012            3
155800           1.012            3
155900           1.012            3
156000           1.012            3
156100           1.012            3
156200           1.012            3
156300           1.011            3
156400           1.011            3
156500           1.011            3
156600           1.011            3
156700           1.011            3
156800           1.011            3
156900           1.011            3
157000           1.011            3
157100           1.011            3
157200           1.011            3
157300           1.011            3
157400           1.011            3
157500           1.011            3
157600           1.011            3
157700           1.011            3
157800           1.011            3
157900           1.011            3
158000           1.010            3
158100           1.010            3
158200           1.010            3
158300           1.010            3
158400           1.010            10
158500           1.010            10
158600           1.009            10
158700           1.009            10
158800           1.010            10
158900           1.009            10
159000           1.010            10
159100           1.010            10
159200           1.010            10
159300           1.010            10
159400           1.010            10
159500           1.010            10
159600           1.010            3
159700           1.010            10
159800           1.010            3
159900           1.010            3
160000           1.010            3
160100           1.010            3
160200           1.010            3
160300           1.010            10
160400           1.010            3
160500           1.010            3
160600           1.010            3
160700           1.010            3
160800           1.010            3
160900           1.010            3
161000           1.009            10
```

161100	1.009	10
161200	1.009	10
161300	1.009	10
161400	1.009	10
161500	1.009	10
161600	1.009	10
161700	1.009	10
161800	1.009	10
161900	1.009	10
162000	1.009	10
162100	1.009	3
162200	1.010	3
162300	1.010	3
162400	1.010	3
162500	1.010	3
162600	1.010	3
162700	1.010	3
162800	1.010	3
162900	1.010	3
163000	1.010	3
163100	1.010	3
163200	1.010	3
163300	1.010	3
163400	1.010	3
163500	1.011	3
163600	1.011	3
163700	1.011	3
163800	1.011	3
163900	1.011	3
164000	1.011	3
164100	1.012	3
164200	1.012	3
164300	1.012	3
164400	1.012	3
164500	1.012	3
164600	1.012	3
164700	1.012	3
164800	1.012	3
164900	1.012	3
165000	1.012	3
165100	1.012	3
165200	1.012	3
165300	1.011	3
165400	1.011	3
165500	1.011	3
165600	1.011	3
165700	1.011	3
165800	1.011	3
165900	1.011	3
166000	1.011	3
166100	1.011	3
166200	1.011	3
166300	1.011	3
166400	1.012	3
166500	1.012	3
166600	1.012	3
166700	1.012	3
166800	1.012	3
166900	1.012	3
167000	1.011	3
167100	1.011	3
167200	1.011	3
167300	1.011	3
167400	1.011	3
167500	1.010	3
167600	1.010	3
167700	1.010	3
167800	1.010	3
167900	1.010	3
168000	1.010	3
168100	1.010	3
168200	1.010	3
168300	1.010	3
168400	1.010	3

```
168500          1.010          3
168600          1.010          3
168700          1.010          3
168800          1.010          3
168900          1.010          3
169000          1.010          3
169100          1.010          3
169200          1.010          3
169300          1.009          3
169400          1.009          3
169500          1.009          10
169600          1.009          10
169700          1.009          10
169800          1.009          10
169900          1.009          10
170000          1.009          3
170100          1.009          3
170200          1.009          10
170300          1.009          10
170400          1.009          10
170500          1.009          3
170600          1.009          3
170700          1.009          3
170800          1.009          3
170900          1.009          3
171000          1.009          3
171100          1.009          3
171200          1.009          3
171300          1.009          3
171400          1.009          3
171500          1.009          3
171600          1.009          3
171700          1.009          3
171800          1.009          3
171900          1.009          3
172000          1.009          3
172100          1.008          3
172200          1.008          3
172300          1.008          10
172400          1.007          10
172500          1.007          10
172600          1.007          10
172700          1.007          10
172800          1.007          3
172900          1.008          3
173000          1.008          3
173100          1.008          3
173200          1.008          3
173300          1.008          3
173400          1.007          3
173500          1.007          3
173600          1.007          3
173700          1.007          3
173800          1.007          3
173900          1.007          3
174000          1.007          3
174100          1.007          3
174200          1.007          3
174300          1.007          3
174400          1.007          3
174500          1.007          3
174600          1.007          3
174700          1.007          10
174800          1.007          10
174900          1.007          10
175000          1.007          10
175100          1.007          10
175200          1.007          10
175300          1.007          10
175400          1.007          10
175500          1.007          10
175600          1.007          10
175700          1.007          10
175800          1.007          10
```

```
     175900            1.007            10
     176000            1.007            10
     176100            1.007            10
     176200            1.007            10
     176300            1.007            10
     176400            1.007            10
     176500            1.007            10
     176600            1.007            10
     176700            1.007            10
     176800            1.007            10
     176900            1.007            10
     177000            1.007            10
     177100            1.007            10
     177200            1.007            10
     177300            1.007            10
     177400            1.007            10
     177500            1.007            10
     177600            1.007            10
     177700            1.007            10
     177800            1.007            10
     177900            1.007            10
     178000            1.007            10
     178100            1.007            10
     178200            1.007            10
     178300            1.007            10
     178400            1.007            10
     178500            1.007            10
     178600            1.007            10
     178700            1.007            10
     178800            1.007            10
     178900            1.007            10
     179000            1.007            10
     179100            1.007            10
     179200            1.007            10
     179300            1.006            10
     179400            1.006            10
     179500            1.006            10
     179600            1.006            10
     179700            1.006            10
     179800            1.006            10
     179900            1.007            10
     180000            1.007            10
     180100            1.007            10
     180200            1.007            10
     180300            1.006            10
     180400            1.006            10
     180500            1.006            10
     180600            1.006            10
     180700            1.006            10
     180800            1.006            10
     180900            1.006            10
     181000            1.006            10
     181100            1.006            10
     181200            1.006            10
     181300            1.006            10
     181400            1.006            10
     181500            1.006            10
     181600            1.006            10
     181700            1.006            10
     181800            1.006            10
     181900            1.006            10
     182000            1.006            10
     182100            1.006            10
     182200            1.006            10
     182300            1.006            10
     182400            1.006            10
     182500            1.006            10
     182600            1.006            10
     182700            1.006            10
     182800            1.006            10
     182900            1.006            10
     183000            1.006            10
     183100            1.006            10
     183200            1.006            10
```

183300	1.006	10
183400	1.006	10
183500	1.006	10
183600	1.006	10
183700	1.006	10
183800	1.006	10
183900	1.006	10
184000	1.005	10
184100	1.005	10
184200	1.005	10
184300	1.005	10
184400	1.005	10
184500	1.005	10
184600	1.005	10
184700	1.004	10
184800	1.003	10
184900	1.002	10
185000	1.002	10
185100	1.002	10
185200	1.002	10
185300	1.002	10
185400	1.002	10
185500	1.002	10
185600	1.002	10
185700	1.002	10
185800	1.002	10
185900	1.002	10
186000	1.002	10
186100	1.003	20
186200	1.003	20
186300	1.003	20
186400	1.003	20
186500	1.003	20
186600	1.003	20
186700	1.003	20
186800	1.003	20
186900	1.003	20
187000	1.003	20
187100	1.002	20
187200	1.003	20
187300	1.003	20
187400	1.002	10
187500	1.002	10
187600	1.002	10
187700	1.002	20
187800	1.002	20
187900	1.003	20
188000	1.003	20
188100	1.003	20
188200	1.003	20
188300	1.003	20
188400	1.003	20
188500	1.003	20
188600	1.003	20
188700	1.003	20
188800	1.003	20
188900	1.003	20
189000	1.003	20
189100	1.002	20
189200	1.002	10
189300	1.002	10
189400	1.002	10
189500	1.002	10
189600	1.002	10
189700	1.002	10
189800	1.002	10
189900	1.002	10
190000	1.002	10
190100	1.002	10
190200	1.002	20
190300	1.002	20
190400	1.002	20
190500	1.002	20
190600	1.002	20

```
     190700           1.002              20
     190800           1.002              20
     190900           1.002              20
     191000           1.002              20
     191100           1.002              20
     191200           1.002              20
     191300           1.002              20
     191400           1.002              20
     191500           1.002              20
     191600           1.002              20


TECHNICAL 5 OUTPUT


DIAGRAM INFORMATION

  Mplus diagrams are currently not available for multilevel analysis.
  No diagram output was produced.


     Beginning Time:  21:59:59
        Ending Time:  22:33:26
       Elapsed Time:  00:33:27



MUTHEN & MUTHEN
3463 Stoner Ave.
Los Angeles, CA  90066

Tel: (310) 391-9971
Fax: (310) 391-8971
Web: www.StatModel.com
Support: Support@StatModel.com

Copyright (c) 1998-2020 Muthen & Muthen
```

Mplus Output Full Multilevel SEM:

```
Mplus VERSION 8.4
MUTHEN & MUTHEN
10/14/2021  12:11 PM

INPUT INSTRUCTIONS

  TITLE:

  DATA: FILE = mpltry6.dat;

  VARIABLE:
   names =  pno wave sex yol verst nopart
         basagec90 agec90 tims tsbase
         ttd ttdcens basettd basttdc
         aut pil ema sac dep
         pa na swls adlinv hyp
         visus sfenerg sfsofunc sfpain sfgen sfchang
         anges anget akzet mortinv mortperc
         basadlinv basvisus bashkr bassssc
         bassfen bassfso bassfpain bassfgen bassfcha
         hkr sl sssco;
  usevariables = aut ema pil sac pa na swls tsbase basttdc
                 bashkr bassssc basagec90 sex yol;
  cluster = PNO;
  missing = .;
  within = tsbase;
  between = basttdc bashkr bassssc basagec90 sex yol;

 DEFINE:
  center sex yol (grandmean);

 ANALYSIS:
  type = twolevel random;
  estimator = BAYES;
  chains = 2;
  biterations=1000000;
  BCONVERGENCE = 0.00125;

 MODEL:

  %WITHIN%

  slopeaut | aut ON tsbase;
  lnvaut | aut;
  slopeema | ema ON tsbase;
  lnvema | ema;
  slopepil | pil ON tsbase;
  lnvpil | pil;
  slopesac | sac ON tsbase;
  lnvsac | sac;
  slopels | swls ON tsbase;
  lnvls | swls;
  slopena | na ON tsbase;
  lnvna | na;
  slopepa | pa ON tsbase;
  lnvpa | pa;

  %BETWEEN%

  physcon by bashkr@1 bassssc;

  BASTTDC ON
   PHYSCON*0.413
   SLOPEAUT*-7.254
   LNVAUT*1.433
   SLOPEEMA*10.407
   LNVEMA*1.122
   SLOPEPIL*-1.873
   LNVPIL*0.399
```

```
 SLOPESAC*-0.043
 LNVSAC*0.190
 SLOPELS*-1.418
 LNVLS*0.030
 SLOPENA*1.873
 LNVNA*0.967
 SLOPEPA*8.963
 LNVPA*-0.699
 AUT*-2.160
 EMA*0.883
 PIL*-0.099
 SAC*-0.252
 SWLS*0.127
 NA*-0.827
 PA*0.498
 SEX*1.427
 YOL*-0.248
 BASAGEC90*-0.255
 PHYSCON*8.685;

[ aut*4.014 ] (m1);
[ pil*3.242 ] (m2);
[ ema*4.130 ] (m3);
[ sac*4.076] (m4);
[ pa*3.314 ] (m5);
[ swls*3.688 ] (m6);
[ na*2.110 ] (m7);
[ slopeaut*-0.005 ] (ms1);
[ lnvaut*-2.018 ] (mv1);
[ slopeema*-0.011 ] (ms3);
[ lnvema*-1.911 ] (mv3);
[ slopepil*-0.011 ] (ms2);
[ lnvpil*-1.935 ] (mv2);
[ slopesac*-0.004 ] (ms4);
[ lnvsac*-2.234 ] (mv4);
[ slopels*-0.002 ] (ms6);
[ lnvls*-1.963 ] (mv6);
[ slopena*-0.002 ] (ms7);
[ lnvna*-2.531 ] (mv7);
[ slopepa*-0.015 ] (ms5);
[ lnvpa*-2.300 ] (mv5);

[ BASTTDC*19.379];
[ BASHKR*18.495];
[ BASSSSC*1.334];

aut*0.224;
pil*0.383;
ema*0.275;
sac*0.283;
pa*0.329;
swls*0.595;
na*0.344;
slopeaut*0.001
lnvaut*0.354;
slopeema*0.001;
lnvema*0.174;
slopepil*0.001;
lnvpil*0.423;
slopesac*0.001;
lnvsac*0.348;
slopels*0.001;
lnvls*0.392;
slopena*0.000;
lnvna*0.328;
slopepa*0.001;
lnvpa*0.340;

aut with slopeaut*-0.003
         lnvaut*0.123;
slopeaut with lnvaut*-0.002;
pil with slopepil*-0.007
         lnvpil*-0.076;
slopepil with lnvpil*0.002;
```

```
  ema with slopeema*-0.003
           lnvema*0.007;
  slopeema with lnvema*-0.001;
  sac with slopesac*-0.004
           lnvsac*-0.010;
  slopesac with lnvsac*0.000;
  swls with slopels*-0.005
            lnvls*-0.153;
  slopels with lnvls*0.001;
  pa with slopepa*-0.001
          lnvpa*0.065;
  slopepa with lnvpa*-0.001;
  na with slopena*-0.003
          lnvna*0.180;
  slopena with lnvna*-0.001;

 MODEL PRIORS:
  m1 ~ N(3.98,0.048);
  m3 ~ N(4.05,0.078);
  m2 ~ N(3.16,0.109);
  m4 ~ N(4.03,0.109);
  m7 ~ N(2.12,0.102);
  m5 ~ N(3.20,0.116);
  m6 ~ N(3.66,0.372);
  ms1 ~ N(-0.006,0.000004);
  ms3 ~ N(-0.052,0.0001);
  ms2 ~ N(-0.075,0.0001);
  ms4 ~ N(-0.009,0.00026);
  ms6 ~ N(0.045,0.00053);
  ms7 ~ N(-0.029,0.00017);
  ms5 ~ N(-0.044,0.000036);

 OUTPUT:
  standardized
  tech5 tech8 tech16;




*** WARNING in VARIABLE command
  Note that only the first 8 characters of variable names are used in the output.
  Shorten variable names to avoid any confusion.
*** WARNING in MODEL command
  In the MODEL command, the following variable is an x-variable on the BETWEEN
  level and a y-variable on the WITHIN level.  This variable will be treated
  as a y-variable on both levels:  AUT
*** WARNING in MODEL command
  In the MODEL command, the following variable is an x-variable on the BETWEEN
  level and a y-variable on the WITHIN level.  This variable will be treated
  as a y-variable on both levels:  EMA
*** WARNING in MODEL command
  In the MODEL command, the following variable is an x-variable on the BETWEEN
  level and a y-variable on the WITHIN level.  This variable will be treated
  as a y-variable on both levels:  PIL
*** WARNING in MODEL command
  In the MODEL command, the following variable is an x-variable on the BETWEEN
  level and a y-variable on the WITHIN level.  This variable will be treated
  as a y-variable on both levels:  SAC
*** WARNING in MODEL command
  In the MODEL command, the following variable is an x-variable on the BETWEEN
  level and a y-variable on the WITHIN level.  This variable will be treated
  as a y-variable on both levels:  PA
*** WARNING in MODEL command
  In the MODEL command, the following variable is an x-variable on the BETWEEN
  level and a y-variable on the WITHIN level.  This variable will be treated
  as a y-variable on both levels:  NA
*** WARNING in MODEL command
  In the MODEL command, the following variable is an x-variable on the BETWEEN
  level and a y-variable on the WITHIN level.  This variable will be treated
  as a y-variable on both levels:  SWLS
*** WARNING
  One or more individual-level variables have no variation within a
  cluster for the following clusters.
```

```
     Variable   Cluster IDs with no within-cluster variation

      AUT         10130 11297 16142 16798 11258 15175
      EMA         10969 16142 16798 11384
      PIL         10130 11297 16142 16798 16510 10211
      SAC         16142 16798 15510
      PA          10130 10144 10313 11240 11741 15018 16142 16798 18650 11028
      NA          10130 10144 10313 11240 11741 15018 16142 16798 11371 11295
      SWLS        10130 10144 10313 10473 10893 11240 11297 11741 15018 16142 16798 11258 15510
                  18252 10228

   9 WARNING(S) FOUND IN THE INPUT INSTRUCTIONS



SUMMARY OF ANALYSIS

Number of groups                                                 1
Number of observations                                         682

Number of dependent variables                                   10
Number of independent variables                                  4
Number of continuous latent variables                           15

Observed dependent variables

  Continuous
   BASTTDC     BASHKR      BASSSSC     AUT         EMA         PIL
   SAC         PA          NA          SWLS

Observed independent variables
   TSBASE      BASAGEC9    SEX         YOL

Continuous latent variables
   PHYSCON     SLOPEAUT    LNVAUT      SLOPEEMA    LNVEMA      SLOPEPIL
   LNVPIL      SLOPESAC    LNVSAC      SLOPELS     LNVLS       SLOPENA
   LNVNA       SLOPEPA     LNVPA

Variables with special functions

  Cluster variable      PNO

  Within variables
   TSBASE

  Between variables
   BASTTDC     BASHKR      BASSSSC     BASAGEC9    SEX         YOL

  Centering (GRANDMEAN)
   SEX         YOL


Estimator                                                    BAYES
Specifications for Bayesian Estimation
  Point estimate                                            MEDIAN
  Number of Markov chain Monte Carlo (MCMC) chains               2
  Random seed for the first chain                                0
  Starting value information                           UNPERTURBED
  Algorithm used for Markov chain Monte Carlo           GIBBS(PX1)
  Convergence criterion                                 0.125D-02
  Maximum number of iterations                             1000000
  K-th iteration used for thinning                               1

Input data file(s)
  mpltry6.dat
Input data format  FREE


SUMMARY OF DATA

     Number of clusters                       124

       Size (s)    Cluster ID with Size s
```

1	10026 10130 10144 10313 10473 10558 10567 10637 10663 10893 10901 10907 10911 10939 10969 11157 11240 11294 11297 11741 15018 15177 15241 15319 15564 16142 16423 16610 16669 16798 17542 18507 18650
2	15239 11258 10181 15510 10615 11341 16303 11371 16510 11384 11485 11028 16862 10211 17780 18004 18252 15175 10228
3	11573 11295 17130 11100 17745 10340 10460 10461 10210 11501
4	10904 11457 10434 11493 10811 17593
5	15424 10666 12411 11343 11005
6	10159 15092 15121 15426 11593 11163 10139 16220 19067
7	10354 11230 10894 10724 10470
8	10252 10528 11038 10033 11336 11253
9	15623 11055
10	12037 10167 17197 15009 19500
11	11233 10444 15714 10533
12	11079 10577 15378 12714 10986 10428
13	11420 11002
14	11450
15	10108 10906 10551 15231 10902
16	15141 10940 16103 15472 11528 11378

COVARIANCE COVERAGE OF DATA

Minimum covariance coverage value 0.100

Number of missing data patterns 31

PROPORTION OF DATA PRESENT

Covariance Coverage

	BASTTDC	BASHKR	BASSSSC	AUT	EMA
BASTTDC	0.944				
BASHKR	0.912	0.968			
BASSSSC	0.935	0.968	0.991		
AUT	0.921	0.949	0.971	0.977	
EMA	0.921	0.944	0.966	0.962	0.974
PIL	0.909	0.931	0.952	0.957	0.947
SAC	0.928	0.952	0.974	0.969	0.969
PA	0.908	0.943	0.956	0.950	0.943
NA	0.911	0.947	0.960	0.955	0.947
SWLS	0.918	0.956	0.968	0.962	0.957
TSBASE	0.944	0.968	0.991	0.977	0.974
BASAGEC9	0.944	0.968	0.991	0.977	0.974
SEX	0.944	0.968	0.991	0.977	0.974
YOL	0.944	0.968	0.991	0.977	0.974

Covariance Coverage

	PIL	SAC	PA	NA	SWLS
PIL	0.957				
SAC	0.955	0.981			
PA	0.931	0.950	0.962		
NA	0.935	0.955	0.962	0.966	
SWLS	0.943	0.965	0.952	0.956	0.974
TSBASE	0.957	0.981	0.962	0.966	0.974
BASAGEC9	0.957	0.981	0.962	0.966	0.974
SEX	0.957	0.981	0.962	0.966	0.974
YOL	0.957	0.981	0.962	0.966	0.974

Covariance Coverage

	TSBASE	BASAGEC9	SEX	YOL
TSBASE	1.000			
BASAGEC9	1.000	1.000		

```
 SEX            1.000         1.000         1.000
 YOL            1.000         1.000         1.000         1.000


UNIVARIATE SAMPLE STATISTICS


     UNIVARIATE HIGHER-ORDER MOMENT DESCRIPTIVE STATISTICS

         Variable/         Mean/     Skewness/   Minimum/ % with                Percentiles
        Sample Size      Variance    Kurtosis    Maximum  Min/Max      20%/60%    40%/80%    Median

     BASTTDC               5.519       0.456       0.250    0.83%       2.250      4.250      5.083
             120.000      10.267      -0.614      12.667    1.67%       6.083      8.250
     BASHKR               18.519       1.282       5.330    0.89%      12.330     16.500     17.750
             112.000      48.308       2.780      49.000    0.89%      18.670     23.330
     BASSSSC               1.333       0.640       0.000   53.33%       0.000      0.000      0.000
             120.000       2.606      -1.294       4.000   17.50%       1.000      3.000
     AUT                   3.892      -0.192       2.222    0.30%       3.333      3.778      3.889
             666.000       0.344      -0.528       5.000    2.70%       4.111      4.444
     EMA                   3.936      -0.567       1.222    0.15%       3.444      3.778      4.000
             664.000       0.400       0.104       5.000    2.11%       4.111      4.556
     PIL                   3.027      -0.004       1.250    0.15%       2.500      2.889      3.000
             653.000       0.371       0.003       5.000    0.15%       3.222      3.556
     SAC                   3.990      -0.735       1.889    0.30%       3.556      3.875      4.000
             669.000       0.362       0.733       5.000    2.39%       4.222      4.500
     PA                    3.172       0.047       1.380    0.15%       2.600      3.000      3.135
             656.000       0.406      -0.288       4.800    0.30%       3.300      3.700
     NA                    2.046       0.407       1.000    3.19%       1.500      1.800      2.000
             659.000       0.369      -0.249       4.100    0.15%       2.200      2.600
     SWLS                  3.782      -0.748       1.000    0.30%       3.200      3.750      3.800
             664.000       0.591       0.556       5.000    5.72%       4.000      4.400
     TSBASE               36.711       0.258       0.000    3.08%      13.000     27.000     36.000
             682.000     594.554      -0.917      91.000    0.59%      41.000     60.000
     BASAGEC90             0.106       0.407      -3.917    0.81%      -2.833     -1.250     -0.792
             124.000       8.313      -1.090       6.417    0.81%       0.500      3.083
     SEX                   0.000      -1.426      -0.790   20.97%      -0.790      0.210      0.210
             124.000       0.166       0.035       0.210   79.03%       0.210      0.210
     YOL                   0.000       0.868      -3.387   20.97%      -3.387     -1.387     -1.387
             124.000       8.737      -0.315       5.613   17.74%       0.613      0.613


THE MODEL ESTIMATION TERMINATED NORMALLY

     USE THE FBITERATIONS OPTION TO INCREASE THE NUMBER OF ITERATIONS BY A FACTOR
     OF AT LEAST TWO TO CHECK CONVERGENCE AND THAT THE PSR VALUE DOES NOT INCREASE.



MODEL FIT INFORMATION

Number of Free Parameters                              96

Information Criteria

          Deviance (DIC)                         7545.832
          Estimated Number of Parameters (pD)    1464.805



MODEL RESULTS

                                Posterior  One-Tailed         95% C.I.
                    Estimate       S.D.      P-Value   Lower 2.5%  Upper 2.5%  Significance

Within Level

Between Level

 PHYSCON  BY
    BASHKR             1.000       0.000      0.000       1.000       1.000
    BASSSSC            0.364       0.415      0.000       0.086       1.409      *
```

```
 BASTTDC    ON
    PHYSCON            0.412       0.390      0.006       0.076       1.313      *
    SLOPEAUT          -7.199      17.458      0.337     -41.746      26.814
    LNVAUT             1.425       1.090      0.075      -0.583       3.733
    SLOPEEMA          10.418      18.387      0.284     -25.817      46.391
    LNVEMA             1.138       1.625      0.211      -2.069       4.395
    SLOPEPIL          -1.854      19.188      0.461     -39.792      35.556
    LNVPIL             0.401       0.844      0.312      -1.297       2.037
    SLOPESAC          -0.070      19.287      0.499     -38.238      37.594
    LNVSAC             0.184       1.006      0.423      -1.818       2.173
    SLOPELS           -1.397      18.526      0.470     -37.659      35.131
    LNVLS              0.026       0.912      0.488      -1.812       1.800
    SLOPENA            1.903      18.670      0.459     -34.848      38.471
    LNVNA              0.958       1.257      0.195      -1.292       3.659
    SLOPEPA            8.976      17.352      0.301     -25.181      43.072
    LNVPA             -0.691       0.943      0.217      -2.611       1.126

 BASTTDC    ON
    AUT               -2.157       1.136      0.023      -4.522      -0.042      *
    EMA                0.881       1.121      0.213      -1.321       3.092
    PIL               -0.097       0.878      0.456      -1.826       1.626
    SAC               -0.257       1.102      0.406      -2.437       1.899
    SWLS               0.126       0.796      0.437      -1.408       1.717
    NA                -0.818       1.020      0.192      -2.983       1.040
    PA                 0.494       0.897      0.288      -1.231       2.290
    SEX                1.432       0.826      0.037      -0.144       3.109
    YOL               -0.248       0.108      0.011      -0.461      -0.036      *
    BASAGEC90         -0.255       0.108      0.009      -0.468      -0.043      *

 AUT      WITH
    SLOPEAUT          -0.003       0.002      0.038      -0.006       0.000
    LNVAUT             0.123       0.064      0.021       0.004       0.258      *

 SLOPEAUT WITH
    LNVAUT            -0.002       0.002      0.124      -0.008       0.002

 PIL      WITH
    SLOPEPIL          -0.007       0.003      0.000      -0.012      -0.003      *
    LNVPIL            -0.076       0.077      0.151      -0.234       0.073

 SLOPEPIL WITH
    LNVPIL             0.002       0.003      0.253      -0.004       0.008

 EMA      WITH
    SLOPEEMA          -0.003       0.002      0.040      -0.007       0.000
    LNVEMA             0.008       0.057      0.443      -0.102       0.123

 SLOPEEMA WITH
    LNVEMA            -0.001       0.002      0.290      -0.005       0.003

 SAC      WITH
    SLOPESAC          -0.004       0.002      0.015      -0.008       0.000      *
    LNVSAC            -0.101       0.063      0.045      -0.234       0.017

 SLOPESAC WITH
    LNVSAC             0.000       0.003      0.468      -0.005       0.005

 SWLS     WITH
    SLOPELS           -0.005       0.003      0.022      -0.012       0.000      *
    LNVLS             -0.153       0.099      0.054      -0.356       0.036

 SLOPELS  WITH
    LNVLS              0.001       0.003      0.283      -0.004       0.007

 PA       WITH
    SLOPEPA           -0.001       0.002      0.318      -0.005       0.003
    LNVPA              0.065       0.077      0.180      -0.073       0.230

 SLOPEPA  WITH
    LNVPA             -0.001       0.003      0.273      -0.007       0.003

 NA       WITH
    SLOPENA           -0.003       0.002      0.048      -0.007       0.001
    LNVNA              0.179       0.072      0.003       0.052       0.337      *
```

```
 SLOPENA  WITH
    LNVNA             -0.001       0.002      0.359      -0.005       0.003

 Means
    AUT                4.014       0.051      0.000       3.914       4.116      *
    EMA                4.129       0.058      0.000       4.015       4.244      *
    PIL                3.242       0.067      0.000       3.111       3.375      *
    SAC                4.076       0.058      0.000       3.960       4.187      *
    PA                 3.314       0.063      0.000       3.191       3.438      *
    NA                 2.110       0.061      0.000       1.992       2.232      *
    SWLS               3.688       0.082      0.000       3.525       3.848      *
    SLOPEAUT          -0.005       0.002      0.004      -0.008      -0.001      *
    LNVAUT            -2.019       0.118      0.000      -2.266      -1.803      *
    SLOPEEMA          -0.011       0.003      0.000      -0.017      -0.004      *
    LNVEMA            -1.913       0.106      0.000      -2.131      -1.717      *
    SLOPEPIL          -0.011       0.003      0.000      -0.017      -0.005      *
    LNVPIL            -1.935       0.122      0.000      -2.186      -1.704      *
    SLOPESAC          -0.004       0.003      0.094      -0.010       0.002
    LNVSAC            -2.234       0.110      0.000      -2.452      -2.017      *
    SLOPELS            0.002       0.003      0.300      -0.005       0.008
    LNVLS             -1.968       0.112      0.000      -2.191      -1.751      *
    SLOPENA           -0.002       0.003      0.230      -0.008       0.004
    LNVNA             -2.529       0.110      0.000      -2.757      -2.324      *
    SLOPEPA           -0.015       0.003      0.000      -0.022      -0.010      *
    LNVPA             -2.302       0.118      0.000      -2.539      -2.074      *

 Intercepts
    BASTTDC           19.378       8.657      0.011       2.922      37.202      *
    BASHKR            18.495       0.677      0.000      17.164      19.825      *
    BASSSSC            1.334       0.151      0.000       1.037       1.630      *

 Variances
    AUT                0.224       0.047      0.000       0.147       0.332      *
    EMA                0.275       0.056      0.000       0.183       0.403      *
    PIL                0.383       0.077      0.000       0.259       0.559      *
    SAC                0.283       0.058      0.000       0.189       0.416      *
    PA                 0.329       0.062      0.000       0.230       0.474      *
    NA                 0.344       0.062      0.000       0.246       0.486      *
    SWLS               0.595       0.111      0.000       0.417       0.852      *
    PHYSCON            8.734       8.707      0.000       0.912      34.504      *
    SLOPEAUT           0.001       0.000      0.000       0.000       0.001      *
    LNVAUT             0.357       0.137      0.000       0.164       0.696      *
    SLOPEEMA           0.001       0.000      0.000       0.000       0.001      *
    LNVEMA             0.174       0.101      0.000       0.042       0.430      *
    SLOPEPIL           0.001       0.000      0.000       0.000       0.001      *
    LNVPIL             0.425       0.157      0.000       0.206       0.818      *
    SLOPESAC           0.001       0.000      0.000       0.000       0.001      *
    LNVSAC             0.348       0.140      0.000       0.152       0.696      *
    SLOPELS            0.001       0.000      0.000       0.000       0.001      *
    LNVLS              0.390       0.144      0.000       0.181       0.744      *
    SLOPENA            0.000       0.000      0.000       0.000       0.001      *
    LNVNA              0.326       0.142      0.000       0.131       0.683      *
    SLOPEPA            0.001       0.000      0.000       0.000       0.001      *
    LNVPA              0.340       0.142      0.000       0.134       0.687      *

 Residual Variances
    BASTTDC            5.455       1.468      0.000       3.077       8.820      *
    BASHKR            41.136       9.642      0.000      17.697      57.874      *
    BASSSSC            1.554       0.669      0.000       0.153       2.713      *


STANDARDIZED MODEL RESULTS


STDYX Standardization

                                Posterior  One-Tailed         95% C.I.
                    Estimate       S.D.      P-Value   Lower 2.5%  Upper 2.5%  Significance

Within-Level Standardized Estimates Averaged Over Clusters

 SLOPEAUT | AUT ON
    TSBASE            -0.060       0.024      0.005      -0.108      -0.013      *
```

```
 SLOPEEMA | EMA ON
    TSBASE            -0.155       0.024      0.000      -0.203      -0.108      *

 SLOPEPIL | PIL ON
    TSBASE            -0.136       0.024      0.000      -0.183      -0.086      *

 SLOPESAC | SAC ON
    TSBASE            -0.085       0.024      0.001      -0.134      -0.037      *

 SLOPELS | SWLS ON
    TSBASE             0.014       0.024      0.287      -0.033       0.062

 SLOPENA | NA ON
    TSBASE            -0.014       0.026      0.299      -0.061       0.036

 SLOPEPA | PA ON
    TSBASE            -0.189       0.024      0.000      -0.233      -0.142      *

 LNVAUT |
    AUT                0.885       0.013      0.000       0.859       0.910      *

 LNVEMA |
    EMA                0.845       0.014      0.000       0.816       0.872      *

 LNVPIL |
    PIL                0.857       0.015      0.000       0.826       0.884      *

 LNVSAC |
    SAC                0.845       0.016      0.000       0.814       0.876      *

 LNVLS |
    SWLS               0.867       0.013      0.000       0.842       0.892      *

 LNVNA |
    NA                 0.855       0.014      0.000       0.823       0.880      *

 LNVPA |
    PA                 0.826       0.015      0.000       0.798       0.855      *

Between Level

 PHYSCON  BY
    BASHKR             0.414       0.167      0.000       0.137       0.805      *
    BASSSSC            0.650       0.188      0.000       0.263       0.971      *

 BASTTDC    ON
    PHYSCON            0.316       0.118      0.006       0.076       0.538      *
    SLOPEAUT          -0.044       0.105      0.337      -0.250       0.162
    LNVAUT             0.227       0.159      0.075      -0.086       0.538
    SLOPEEMA           0.067       0.117      0.284      -0.163       0.295
    LNVEMA             0.127       0.153      0.211      -0.183       0.411
    SLOPEPIL          -0.012       0.121      0.461      -0.248       0.225
    LNVPIL             0.069       0.139      0.312      -0.206       0.336
    SLOPESAC           0.000       0.119      0.499      -0.234       0.234
    LNVSAC             0.029       0.147      0.423      -0.262       0.315
    SLOPELS           -0.009       0.115      0.470      -0.234       0.217
    LNVLS              0.004       0.142      0.488      -0.276       0.280
    SLOPENA            0.011       0.107      0.459      -0.198       0.219
    LNVNA              0.145       0.175      0.195      -0.180       0.505
    SLOPEPA            0.058       0.110      0.301      -0.157       0.275
    LNVPA             -0.106       0.134      0.217      -0.363       0.160

 BASTTDC    ON
    AUT               -0.268       0.140      0.023      -0.556      -0.005      *
    EMA                0.122       0.150      0.213      -0.179       0.404
    PIL               -0.016       0.140      0.456      -0.285       0.261
    SAC               -0.036       0.150      0.406      -0.326       0.260
    SWLS               0.026       0.157      0.437      -0.279       0.334
    NA                -0.126       0.156      0.192      -0.457       0.156
    PA                 0.075       0.133      0.288      -0.180       0.337
    SEX                0.110       0.062      0.037      -0.011       0.231
    YOL               -0.138       0.059      0.011      -0.252      -0.019      *
    BASAGEC90         -0.129       0.054      0.009      -0.232      -0.022      *
```

```
 AUT      WITH
    SLOPEAUT          -0.240       0.128      0.038     -0.474      0.026
    LNVAUT             0.451       0.202      0.021      0.015      0.798      *

 SLOPEAUT WITH
    LNVAUT            -0.187       0.155      0.124     -0.470      0.132

 PIL      WITH
    SLOPEPIL          -0.458       0.117      0.000     -0.657     -0.202      *
    LNVPIL            -0.194       0.181      0.151     -0.528      0.175

 SLOPEPIL WITH
    LNVPIL             0.114       0.167      0.253     -0.222      0.428

 EMA      WITH
    SLOPEEMA          -0.243       0.131      0.040     -0.479      0.029
    LNVEMA             0.038       0.256      0.443     -0.465      0.532

 SLOPEEMA WITH
    LNVEMA            -0.103       0.181      0.290     -0.441      0.263

 SAC      WITH
    SLOPESAC          -0.299       0.128      0.015     -0.527     -0.030      *
    LNVSAC            -0.333       0.180      0.045     -0.642      0.056

 SLOPESAC WITH
    LNVSAC            -0.014       0.170      0.468     -0.344      0.317

 SWLS     WITH
    SLOPELS           -0.290       0.135      0.022     -0.534     -0.007      *
    LNVLS             -0.330       0.185      0.054     -0.645      0.075

 SLOPELS  WITH
    LNVLS              0.097       0.164      0.283     -0.232      0.406

 PA       WITH
    SLOPEPA           -0.069       0.143      0.318     -0.345      0.213
    LNVPA              0.203       0.209      0.180     -0.230      0.577

 SLOPEPA  WITH
    LNVPA             -0.104       0.167      0.273     -0.418      0.231

 NA       WITH
    SLOPENA           -0.234       0.134      0.048     -0.479      0.042
    LNVNA              0.550       0.161      0.003      0.178      0.806      *

 SLOPENA  WITH
    LNVNA             -0.058       0.157      0.359     -0.359      0.254

 Means
    AUT                8.483       0.887      0.000      6.978     10.450      *
    EMA                7.875       0.813      0.000      6.485      9.682      *
    PIL                5.240       0.525      0.000      4.327      6.383      *
    SAC                7.654       0.797      0.000      6.288      9.412      *
    PA                 5.775       0.545      0.000      4.800      6.934      *
    NA                 3.597       0.319      0.000      3.009      4.260      *
    SWLS               4.782       0.459      0.000      3.955      5.755      *
    SLOPEAUT          -0.199       0.077      0.004     -0.355     -0.052      *
    LNVAUT            -3.387       0.604      0.000     -4.835     -2.473      *
    SLOPEEMA          -0.438       0.133      0.000     -0.703     -0.180      *
    LNVEMA            -4.595       1.594      0.000     -8.981     -2.993      *
    SLOPEPIL          -0.451       0.131      0.000     -0.711     -0.196      *
    LNVPIL            -2.972       0.525      0.000     -4.199     -2.149      *
    SLOPESAC          -0.174       0.132      0.094     -0.434      0.085
    LNVSAC            -3.791       0.792      0.000     -5.720     -2.639      *
    SLOPELS            0.072       0.137      0.300     -0.197      0.339
    LNVLS             -3.161       0.601      0.000     -4.600     -2.245      *
    SLOPENA           -0.097       0.131      0.230     -0.354      0.160
    LNVNA             -4.434       0.969      0.000     -6.884     -3.092      *
    SLOPEPA           -0.632       0.120      0.000     -0.871     -0.399      *
    LNVPA             -3.959       0.896      0.000     -6.219     -2.748      *

 Intercepts
```

```
    BASTTDC            5.093       2.255      0.011       0.757       9.648      *
    BASHKR             2.594       0.202      0.000       2.206       2.996      *
    BASSSSC            0.810       0.105      0.000       0.603       1.017      *

 Variances
    AUT                1.000       0.000      0.000       1.000       1.000
    EMA                1.000       0.000      0.000       1.000       1.000
    PIL                1.000       0.000      0.000       1.000       1.000
    SAC                1.000       0.000      0.000       1.000       1.000
    PA                 1.000       0.000      0.000       1.000       1.000
    NA                 1.000       0.000      0.000       1.000       1.000
    SWLS               1.000       0.000      0.000       1.000       1.000
    PHYSCON            1.000       0.000      0.000       1.000       1.000
    SLOPEAUT           1.000       0.000      0.000       1.000       1.000
    LNVAUT             1.000       0.000      0.000       1.000       1.000
    SLOPEEMA           1.000       0.000      0.000       1.000       1.000
    LNVEMA             1.000       0.000      0.000       1.000       1.000
    SLOPEPIL           1.000       0.000      0.000       1.000       1.000
    LNVPIL             1.000       0.000      0.000       1.000       1.000
    SLOPESAC           1.000       0.000      0.000       1.000       1.000
    LNVSAC             1.000       0.000      0.000       1.000       1.000
    SLOPELS            1.000       0.000      0.000       1.000       1.000
    LNVLS              1.000       0.000      0.000       1.000       1.000
    SLOPENA            1.000       0.000      0.000       1.000       1.000
    LNVNA              1.000       0.000      0.000       1.000       1.000
    SLOPEPA            1.000       0.000      0.000       1.000       1.000
    LNVPA              1.000       0.000      0.000       1.000       1.000

 Residual Variances
    BASTTDC            0.377       0.089      0.000       0.216       0.563      *
    BASHKR             0.828       0.161      0.000       0.351       0.981      *
    BASSSSC            0.577       0.236      0.000       0.057       0.931      *


STDY Standardization

                                Posterior  One-Tailed         95% C.I.
                    Estimate       S.D.      P-Value   Lower 2.5%  Upper 2.5%  Significance

Within-Level Standardized Estimates Averaged Over Clusters

 SLOPEAUT | AUT ON
    TSBASE            -0.004       0.006      0.214      -0.016       0.006

 SLOPEEMA | EMA ON
    TSBASE            -0.018       0.006      0.003      -0.031      -0.006      *

 SLOPEPIL | PIL ON
    TSBASE            -0.019       0.006      0.002      -0.033      -0.007      *

 SLOPESAC | SAC ON
    TSBASE            -0.011       0.007      0.047      -0.025       0.002

 SLOPELS | SWLS ON
    TSBASE             0.002       0.006      0.347      -0.010       0.015

 SLOPENA | NA ON
    TSBASE            -0.005       0.008      0.292      -0.020       0.013

 SLOPEPA | PA ON
    TSBASE            -0.031       0.007      0.000      -0.045      -0.018      *

 LNVAUT |
    AUT                0.885       0.013      0.000       0.859       0.910      *

 LNVEMA |
    EMA                0.845       0.014      0.000       0.816       0.872      *

 LNVPIL |
    PIL                0.857       0.015      0.000       0.826       0.884      *

 LNVSAC |
    SAC                0.845       0.016      0.000       0.814       0.876      *
```

```
 LNVLS |
    SWLS               0.867       0.013      0.000       0.842       0.892      *

 LNVNA |
    NA                 0.855       0.014      0.000       0.823       0.880      *

 LNVPA |
    PA                 0.826       0.015      0.000       0.798       0.855      *

Between Level

 PHYSCON  BY
    BASHKR             0.414       0.167      0.000       0.137       0.805      *
    BASSSSC            0.650       0.188      0.000       0.263       0.971      *

 BASTTDC    ON
    PHYSCON            0.316       0.118      0.006       0.076       0.538      *
    SLOPEAUT          -0.044       0.105      0.337      -0.250       0.162
    LNVAUT             0.227       0.159      0.075      -0.086       0.538
    SLOPEEMA           0.067       0.117      0.284      -0.163       0.295
    LNVEMA             0.127       0.153      0.211      -0.183       0.411
    SLOPEPIL          -0.012       0.121      0.461      -0.248       0.225
    LNVPIL             0.069       0.139      0.312      -0.206       0.336
    SLOPESAC           0.000       0.119      0.499      -0.234       0.234
    LNVSAC             0.029       0.147      0.423      -0.262       0.315
    SLOPELS           -0.009       0.115      0.470      -0.234       0.217
    LNVLS              0.004       0.142      0.488      -0.276       0.280
    SLOPENA            0.011       0.107      0.459      -0.198       0.219
    LNVNA              0.145       0.175      0.195      -0.180       0.505
    SLOPEPA            0.058       0.110      0.301      -0.157       0.275
    LNVPA             -0.106       0.134      0.217      -0.363       0.160

 BASTTDC    ON
    AUT               -0.268       0.140      0.023      -0.556      -0.005      *
    EMA                0.122       0.150      0.213      -0.179       0.404
    PIL               -0.016       0.140      0.456      -0.285       0.261
    SAC               -0.036       0.150      0.406      -0.326       0.260
    SWLS               0.026       0.157      0.437      -0.279       0.334
    NA                -0.126       0.156      0.192      -0.457       0.156
    PA                 0.075       0.133      0.288      -0.180       0.337
    SEX                0.377       0.212      0.037      -0.037       0.794
    YOL               -0.065       0.028      0.011      -0.119      -0.009      *
    BASAGEC90         -0.067       0.028      0.009      -0.121      -0.011      *

 AUT      WITH
    SLOPEAUT          -0.240       0.128      0.038      -0.474       0.026
    LNVAUT             0.451       0.202      0.021       0.015       0.798      *

 SLOPEAUT WITH
    LNVAUT            -0.187       0.155      0.124      -0.470       0.132

 PIL      WITH
    SLOPEPIL          -0.458       0.117      0.000      -0.657      -0.202      *
    LNVPIL            -0.194       0.181      0.151      -0.528       0.175

 SLOPEPIL WITH
    LNVPIL             0.114       0.167      0.253      -0.222       0.428

 EMA      WITH
    SLOPEEMA          -0.243       0.131      0.040      -0.479       0.029
    LNVEMA             0.038       0.256      0.443      -0.465       0.532

 SLOPEEMA WITH
    LNVEMA            -0.103       0.181      0.290      -0.441       0.263

 SAC      WITH
    SLOPESAC          -0.299       0.128      0.015      -0.527      -0.030      *
    LNVSAC            -0.333       0.180      0.045      -0.642       0.056

 SLOPESAC WITH
    LNVSAC            -0.014       0.170      0.468      -0.344       0.317

 SWLS     WITH
    SLOPELS           -0.290       0.135      0.022      -0.534      -0.007      *
```

```
   LNVLS             -0.330       0.185      0.054      -0.645       0.075

SLOPELS  WITH
   LNVLS              0.097       0.164      0.283      -0.232       0.406

PA       WITH
   SLOPEPA           -0.069       0.143      0.318      -0.345       0.213
   LNVPA              0.203       0.209      0.180      -0.230       0.577

SLOPEPA  WITH
   LNVPA             -0.104       0.167      0.273      -0.418       0.231

NA       WITH
   SLOPENA           -0.234       0.134      0.048      -0.479       0.042
   LNVNA              0.550       0.161      0.003       0.178       0.806      *

SLOPENA  WITH
   LNVNA             -0.058       0.157      0.359      -0.359       0.254

Means
   AUT                8.483       0.887      0.000       6.978      10.450      *
   EMA                7.875       0.813      0.000       6.485       9.682      *
   PIL                5.240       0.525      0.000       4.327       6.383      *
   SAC                7.654       0.797      0.000       6.288       9.412      *
   PA                 5.775       0.545      0.000       4.800       6.934      *
   NA                 3.597       0.319      0.000       3.009       4.260      *
   SWLS               4.782       0.459      0.000       3.955       5.755      *
   SLOPEAUT          -0.199       0.077      0.004      -0.355      -0.052      *
   LNVAUT            -3.387       0.604      0.000      -4.835      -2.473      *
   SLOPEEMA          -0.438       0.133      0.000      -0.703      -0.180      *
   LNVEMA            -4.595       1.594      0.000      -8.981      -2.993      *
   SLOPEPIL          -0.451       0.131      0.000      -0.711      -0.196      *
   LNVPIL            -2.972       0.525      0.000      -4.199      -2.149      *
   SLOPESAC          -0.174       0.132      0.094      -0.434       0.085
   LNVSAC            -3.791       0.792      0.000      -5.720      -2.639      *
   SLOPELS            0.072       0.137      0.300      -0.197       0.339
   LNVLS             -3.161       0.601      0.000      -4.600      -2.245      *
   SLOPENA           -0.097       0.131      0.230      -0.354       0.160
   LNVNA             -4.434       0.969      0.000      -6.884      -3.092      *
   SLOPEPA           -0.632       0.120      0.000      -0.871      -0.399      *
   LNVPA             -3.959       0.896      0.000      -6.219      -2.748      *

Intercepts
   BASTTDC            5.093       2.255      0.011       0.757       9.648      *
   BASHKR             2.594       0.202      0.000       2.206       2.996      *
   BASSSSC            0.810       0.105      0.000       0.603       1.017      *

Variances
   AUT                1.000       0.000      0.000       1.000       1.000
   EMA                1.000       0.000      0.000       1.000       1.000
   PIL                1.000       0.000      0.000       1.000       1.000
   SAC                1.000       0.000      0.000       1.000       1.000
   PA                 1.000       0.000      0.000       1.000       1.000
   NA                 1.000       0.000      0.000       1.000       1.000
   SWLS               1.000       0.000      0.000       1.000       1.000
   PHYSCON            1.000       0.000      0.000       1.000       1.000
   SLOPEAUT           1.000       0.000      0.000       1.000       1.000
   LNVAUT             1.000       0.000      0.000       1.000       1.000
   SLOPEEMA           1.000       0.000      0.000       1.000       1.000
   LNVEMA             1.000       0.000      0.000       1.000       1.000
   SLOPEPIL           1.000       0.000      0.000       1.000       1.000
   LNVPIL             1.000       0.000      0.000       1.000       1.000
   SLOPESAC           1.000       0.000      0.000       1.000       1.000
   LNVSAC             1.000       0.000      0.000       1.000       1.000
   SLOPELS            1.000       0.000      0.000       1.000       1.000
   LNVLS              1.000       0.000      0.000       1.000       1.000
   SLOPENA            1.000       0.000      0.000       1.000       1.000
   LNVNA              1.000       0.000      0.000       1.000       1.000
   SLOPEPA            1.000       0.000      0.000       1.000       1.000
   LNVPA              1.000       0.000      0.000       1.000       1.000

Residual Variances
   BASTTDC            0.377       0.089      0.000       0.216       0.563      *
   BASHKR             0.828       0.161      0.000       0.351       0.981      *
```

```
    BASSSSC            0.577       0.236      0.000       0.057       0.931      *


STD Standardization

                                Posterior  One-Tailed         95% C.I.
                    Estimate       S.D.      P-Value   Lower 2.5%  Upper 2.5%  Significance

Within-Level Standardized Estimates Averaged Over Clusters

 SLOPEAUT | AUT ON
    TSBASE            -0.003       0.002      0.070      -0.007       0.001

 SLOPEEMA | EMA ON
    TSBASE            -0.009       0.002      0.001      -0.013      -0.004      *

 SLOPEPIL | PIL ON
    TSBASE            -0.008       0.002      0.001      -0.013      -0.003      *

 SLOPESAC | SAC ON
    TSBASE            -0.004       0.002      0.042      -0.009       0.000

 SLOPELS | SWLS ON
    TSBASE             0.001       0.003      0.276      -0.004       0.006

 SLOPENA | NA ON
    TSBASE            -0.002       0.002      0.226      -0.006       0.003

 SLOPEPA | PA ON
    TSBASE            -0.012       0.002      0.000      -0.016      -0.007      *

 LNVAUT |
    AUT                0.159       0.013      0.000       0.136       0.184      *

 LNVEMA |
    EMA                0.157       0.011      0.000       0.138       0.180      *

 LNVPIL |
    PIL                0.185       0.022      0.000       0.150       0.230      *

 LNVSAC |
    SAC                0.129       0.016      0.000       0.100       0.167      *

 LNVLS |
    SWLS               0.172       0.019      0.000       0.137       0.214      *

 LNVNA |
    NA                 0.093       0.010      0.000       0.080       0.119      *

 LNVPA |
    PA                 0.114       0.010      0.000       0.097       0.135      *

Between Level

 PHYSCON  BY
    BASHKR             2.955       1.238      0.000       0.955       5.874      *
    BASSSSC            1.072       0.323      0.000       0.426       1.651      *

 BASTTDC    ON
    PHYSCON            1.200       0.461      0.006       0.292       2.110      *
    SLOPEAUT          -0.167       0.406      0.337      -0.973       0.623
    LNVAUT             0.862       0.610      0.075      -0.333       2.067
    SLOPEEMA           0.255       0.454      0.284      -0.633       1.151
    LNVEMA             0.482       0.587      0.211      -0.707       1.586
    SLOPEPIL          -0.045       0.466      0.461      -0.970       0.866
    LNVPIL             0.263       0.536      0.312      -0.796       1.303
    SLOPESAC          -0.002       0.462      0.499      -0.916       0.905
    LNVSAC             0.109       0.566      0.423      -1.013       1.216
    SLOPELS           -0.033       0.445      0.470      -0.906       0.847
    LNVLS              0.016       0.547      0.488      -1.061       1.088
    SLOPENA            0.042       0.411      0.459      -0.765       0.849
    LNVNA              0.552       0.671      0.195      -0.699       1.934
    SLOPEPA            0.219       0.425      0.301      -0.613       1.060
    LNVPA             -0.405       0.516      0.217      -1.400       0.620
```

```
 BASTTDC    ON
    AUT               -2.157       1.136      0.023      -4.522      -0.042      *
    EMA                0.881       1.121      0.213      -1.321       3.092
    PIL               -0.097       0.878      0.456      -1.826       1.626
    SAC               -0.257       1.102      0.406      -2.437       1.899
    SWLS               0.126       0.796      0.437      -1.408       1.717
    NA                -0.818       1.020      0.192      -2.983       1.040
    PA                 0.494       0.897      0.288      -1.231       2.290
    SEX                1.432       0.826      0.037      -0.144       3.109
    YOL               -0.248       0.108      0.011      -0.461      -0.036      *
    BASAGEC90         -0.255       0.108      0.009      -0.468      -0.043      *

 AUT      WITH
    SLOPEAUT          -0.112       0.067      0.038      -0.249       0.011
    LNVAUT             0.211       0.103      0.021       0.007       0.407      *

 SLOPEAUT WITH
    LNVAUT            -0.187       0.155      0.124      -0.470       0.132

 PIL      WITH
    SLOPEPIL          -0.282       0.089      0.000      -0.463      -0.113      *
    LNVPIL            -0.119       0.115      0.151      -0.341       0.109

 SLOPEPIL WITH
    LNVPIL             0.114       0.167      0.253      -0.222       0.428

 EMA      WITH
    SLOPEEMA          -0.126       0.075      0.040      -0.279       0.014
    LNVEMA             0.020       0.136      0.443      -0.241       0.294

 SLOPEEMA WITH
    LNVEMA            -0.103       0.181      0.290      -0.441       0.263

 SAC      WITH
    SLOPESAC          -0.158       0.076      0.015      -0.314      -0.015      *
    LNVSAC            -0.175       0.099      0.045      -0.357       0.030

 SLOPESAC WITH
    LNVSAC            -0.014       0.170      0.468      -0.344       0.317

 SWLS     WITH
    SLOPELS           -0.222       0.115      0.022      -0.457      -0.005      *
    LNVLS             -0.252       0.147      0.054      -0.519       0.059

 SLOPELS  WITH
    LNVLS              0.097       0.164      0.283      -0.232       0.406

 PA       WITH
    SLOPEPA           -0.039       0.085      0.318      -0.215       0.119
    LNVPA              0.116       0.124      0.180      -0.132       0.352

 SLOPEPA  WITH
    LNVPA             -0.104       0.167      0.273      -0.418       0.231

 NA       WITH
    SLOPENA           -0.136       0.084      0.048      -0.306       0.024
    LNVNA              0.320       0.102      0.003       0.103       0.503      *

 SLOPENA  WITH
    LNVNA             -0.058       0.157      0.359      -0.359       0.254

 Means
    AUT                4.014       0.051      0.000       3.914       4.116      *
    EMA                4.129       0.058      0.000       4.015       4.244      *
    PIL                3.242       0.067      0.000       3.111       3.375      *
    SAC                4.076       0.058      0.000       3.960       4.187      *
    PA                 3.314       0.063      0.000       3.191       3.438      *
    NA                 2.110       0.061      0.000       1.992       2.232      *
    SWLS               3.688       0.082      0.000       3.525       3.848      *
    SLOPEAUT          -0.199       0.077      0.004      -0.355      -0.052      *
    LNVAUT            -3.387       0.604      0.000      -4.835      -2.473      *
    SLOPEEMA          -0.438       0.133      0.000      -0.703      -0.180      *
    LNVEMA            -4.595       1.594      0.000      -8.981      -2.993      *
```

```
    SLOPEPIL          -0.451       0.131      0.000      -0.711      -0.196      *
    LNVPIL            -2.972       0.525      0.000      -4.199      -2.149      *
    SLOPESAC          -0.174       0.132      0.094      -0.434       0.085
    LNVSAC            -3.791       0.792      0.000      -5.720      -2.639      *
    SLOPELS            0.072       0.137      0.300      -0.197       0.339
    LNVLS             -3.161       0.601      0.000      -4.600      -2.245      *
    SLOPENA           -0.097       0.131      0.230      -0.354       0.160
    LNVNA             -4.434       0.969      0.000      -6.884      -3.092      *
    SLOPEPA           -0.632       0.120      0.000      -0.871      -0.399      *
    LNVPA             -3.959       0.896      0.000      -6.219      -2.748      *

 Intercepts
    BASTTDC           19.378       8.657      0.011       2.922      37.202      *
    BASHKR            18.495       0.677      0.000      17.164      19.825      *
    BASSSSC            1.334       0.151      0.000       1.037       1.630      *

 Variances
    AUT                0.224       0.047      0.000       0.147       0.332      *
    EMA                0.275       0.056      0.000       0.183       0.403      *
    PIL                0.383       0.077      0.000       0.259       0.559      *
    SAC                0.283       0.058      0.000       0.189       0.416      *
    PA                 0.329       0.062      0.000       0.230       0.474      *
    NA                 0.344       0.062      0.000       0.246       0.486      *
    SWLS               0.595       0.111      0.000       0.417       0.852      *
    PHYSCON            1.000       0.000      0.000       1.000       1.000
    SLOPEAUT           1.000       0.000      0.000       1.000       1.000
    LNVAUT             1.000       0.000      0.000       1.000       1.000
    SLOPEEMA           1.000       0.000      0.000       1.000       1.000
    LNVEMA             1.000       0.000      0.000       1.000       1.000
    SLOPEPIL           1.000       0.000      0.000       1.000       1.000
    LNVPIL             1.000       0.000      0.000       1.000       1.000
    SLOPESAC           1.000       0.000      0.000       1.000       1.000
    LNVSAC             1.000       0.000      0.000       1.000       1.000
    SLOPELS            1.000       0.000      0.000       1.000       1.000
    LNVLS              1.000       0.000      0.000       1.000       1.000
    SLOPENA            1.000       0.000      0.000       1.000       1.000
    LNVNA              1.000       0.000      0.000       1.000       1.000
    SLOPEPA            1.000       0.000      0.000       1.000       1.000
    LNVPA              1.000       0.000      0.000       1.000       1.000

 Residual Variances
    BASTTDC            5.455       1.468      0.000       3.077       8.820      *
    BASHKR            41.136       9.642      0.000      17.697      57.874      *
    BASSSSC            1.554       0.669      0.000       0.153       2.713      *


R-SQUARE

Within-Level R-Square Averaged Across Clusters

                                Posterior  One-Tailed         95% C.I.
    Variable        Estimate       S.D.      P-Value   Lower 2.5%  Upper 2.5%

    AUT                0.115       0.013      0.000       0.090       0.141
    EMA                0.155       0.014      0.000       0.128       0.184
    PIL                0.143       0.015      0.000       0.116       0.174
    SAC                0.155       0.016      0.000       0.124       0.186
    PA                 0.174       0.015      0.000       0.145       0.202
    NA                 0.145       0.014      0.000       0.120       0.177
    SWLS               0.133       0.013      0.000       0.107       0.158

Between Level

                                Posterior  One-Tailed         95% C.I.
    Variable        Estimate       S.D.      P-Value   Lower 2.5%  Upper 2.5%

    BASTTDC            0.623       0.089      0.000       0.437       0.784
    BASHKR             0.172       0.161      0.000       0.019       0.649
    BASSSSC            0.423       0.236      0.000       0.069       0.943



TECHNICAL 8 OUTPUT
```

```
TECHNICAL 8 OUTPUT FOR BAYES ESTIMATION

  CHAIN    BSEED
  1        0
  2        285380

                     POTENTIAL       PARAMETER WITH
  ITERATION    SCALE REDUCTION      HIGHEST PSR
  100              3.841              19
  200              3.391              19
  300              2.801              3
  400              3.044              7
  500              3.793              3
  600              2.677              3
  700              1.901              7
  800              1.648              7
  900              1.526              28
  1000             1.529              53
  1100             1.584              65
  1200             1.444              65
  1300             1.304              65
  1400             1.390              95
  1500             1.517              68
  1600             1.507              71
  1700             1.588              71
  1800             1.634              71
  1900             1.636              71
  2000             1.638              71
  2100             1.383              71
  2200             1.299              11
  2300             1.232              17
  2400             1.238              17
  2500             1.251              17
  2600             1.204              19
  2700             1.193              19
  2800             1.220              19
  2900             1.237              17
  3000             1.245              17
  3100             1.233              19
  3200             1.188              19
  3300             1.142              19
  3400             1.126              19
  3500             1.112              19
  3600             1.108              89
  3700             1.091              19
  3800             1.097              19
  3900             1.107              19
  4000             1.115              19
  4100             1.104              19
  4200             1.097              19
  4300             1.121              9
  4400             1.159              59
  4500             1.184              59
  4600             1.147              59
  4700             1.121              59
  4800             1.095              59
  4900             1.096              9
  5000             1.104              9
  5100             1.107              9
  5200             1.129              9
  5300             1.152              9
  5400             1.166              9
  5500             1.172              9
  5600             1.197              9
  5700             1.193              9
  5800             1.200              9
  5900             1.200              9
  6000             1.199              9
  6100             1.204              9
  6200             1.196              9
  6300             1.194              9
  6400             1.185              9
```

6500	1.162	9
6600	1.149	9
6700	1.152	9
6800	1.124	9
6900	1.101	9
7000	1.077	9
7100	1.054	9
7200	1.051	9
7300	1.055	9
7400	1.051	13
7500	1.054	13
7600	1.044	13
7700	1.042	9
7800	1.046	9
7900	1.040	9
8000	1.040	9
8100	1.039	13
8200	1.052	13
8300	1.062	13
8400	1.067	13
8500	1.064	13
8600	1.068	13
8700	1.064	13
8800	1.067	13
8900	1.071	13
9000	1.070	13
9100	1.054	13
9200	1.055	13
9300	1.062	13
9400	1.060	13
9500	1.050	13
9600	1.044	13
9700	1.043	13
9800	1.046	13
9900	1.056	13
10000	1.068	13
10100	1.077	13
10200	1.084	13
10300	1.077	13
10400	1.077	13
10500	1.079	13
10600	1.071	13
10700	1.063	13
10800	1.051	13
10900	1.046	83
11000	1.038	59
11100	1.040	7
11200	1.044	7
11300	1.045	7
11400	1.036	7
11500	1.036	74
11600	1.035	74
11700	1.035	74
11800	1.035	5
11900	1.038	5
12000	1.041	5
12100	1.043	5
12200	1.046	5
12300	1.047	5
12400	1.044	5
12500	1.048	5
12600	1.047	5
12700	1.049	5
12800	1.050	5
12900	1.053	5
13000	1.059	5
13100	1.067	5
13200	1.074	5
13300	1.077	5
13400	1.077	5
13500	1.072	5
13600	1.074	5
13700	1.071	5
13800	1.072	5

13900	1.070	5
14000	1.067	5
14100	1.069	5
14200	1.072	5
14300	1.070	5
14400	1.073	5
14500	1.078	5
14600	1.075	5
14700	1.066	5
14800	1.064	5
14900	1.062	5
15000	1.062	5
15100	1.061	5
15200	1.059	5
15300	1.056	5
15400	1.058	5
15500	1.061	5
15600	1.059	5
15700	1.061	5
15800	1.061	5
15900	1.057	5
16000	1.056	5
16100	1.055	5
16200	1.052	5
16300	1.049	5
16400	1.045	5
16500	1.045	5
16600	1.049	5
16700	1.053	5
16800	1.053	5
16900	1.050	5
17000	1.051	5
17100	1.046	5
17200	1.045	3
17300	1.042	3
17400	1.044	3
17500	1.045	3
17600	1.046	3
17700	1.047	3
17800	1.047	3
17900	1.045	3
18000	1.042	3
18100	1.041	3
18200	1.039	3
18300	1.037	3
18400	1.036	3
18500	1.035	3
18600	1.036	3
18700	1.037	3
18800	1.039	3
18900	1.038	3
19000	1.035	3
19100	1.034	3
19200	1.036	5
19300	1.036	5
19400	1.033	5
19500	1.031	5
19600	1.031	5
19700	1.033	3
19800	1.033	3
19900	1.035	3
20000	1.034	3
20100	1.034	3
20200	1.030	3
20300	1.027	3
20400	1.027	77
20500	1.028	77
20600	1.029	77
20700	1.029	77
20800	1.030	77
20900	1.030	77
21000	1.031	77
21100	1.035	3
21200	1.041	3

```
     21300            1.047             3
     21400            1.050             3
     21500            1.051             3
     21600            1.054             3
     21700            1.053             3
     21800            1.049             3
     21900            1.043             3
     22000            1.041             3
     22100            1.039             3
     22200            1.038             3
     22300            1.037             3
     22400            1.032             3
     22500            1.028             3
     22600            1.028             3
     22700            1.029             3
     22800            1.030             3
     22900            1.029             3
     23000            1.028             3
     23100            1.027             3
     23200            1.029             3
     23300            1.027             3
     23400            1.030             3
     23500            1.033             3
     23600            1.037             3
     23700            1.041             3
     23800            1.045             3
     23900            1.047             3
     24000            1.050             3
     24100            1.052             3
     24200            1.055             3
     24300            1.058             3
     24400            1.058             3
     24500            1.059             3
     24600            1.055             3
     24700            1.056             3
     24800            1.057             3
     24900            1.057             3
     25000            1.057             3
     25100            1.056             3
     25200            1.058             3
     25300            1.059             3
     25400            1.059             3
     25500            1.059             3
     25600            1.058             3
     25700            1.058             3
     25800            1.058             3
     25900            1.057             3
     26000            1.051             3
     26100            1.044             3
     26200            1.041             3
     26300            1.041             3
     26400            1.039             3
     26500            1.038             3
     26600            1.038             3
     26700            1.037             3
     26800            1.033             3
     26900            1.030             3
     27000            1.028             3
     27100            1.026             3
     27200            1.025             3
     27300            1.024             3
     27400            1.023             3
     27500            1.022             3
     27600            1.020             3
     27700            1.019             3
     27800            1.020             3
     27900            1.021             3
     28000            1.021             3
     28100            1.022             3
     28200            1.022             3
     28300            1.023             3
     28400            1.023             3
     28500            1.022             3
     28600            1.023             3
```

28700	1.022	3
28800	1.019	3
28900	1.017	3
29000	1.016	3
29100	1.015	71
29200	1.015	71
29300	1.015	71
29400	1.013	71
29500	1.013	71
29600	1.013	71
29700	1.015	71
29800	1.015	71
29900	1.015	71
30000	1.015	71
30100	1.016	71
30200	1.018	71
30300	1.018	71
30400	1.017	71
30500	1.017	71
30600	1.018	71
30700	1.017	71
30800	1.017	17
30900	1.018	17
31000	1.018	17
31100	1.020	17
31200	1.019	17
31300	1.019	17
31400	1.017	17
31500	1.016	17
31600	1.016	17
31700	1.017	92
31800	1.019	92
31900	1.021	92
32000	1.021	92
32100	1.020	92
32200	1.021	92
32300	1.022	92
32400	1.022	92
32500	1.022	92
32600	1.021	92
32700	1.020	92
32800	1.019	92
32900	1.019	92
33000	1.019	92
33100	1.018	92
33200	1.016	92
33300	1.015	3
33400	1.014	3
33500	1.015	3
33600	1.015	3
33700	1.016	3
33800	1.016	3
33900	1.016	3
34000	1.016	3
34100	1.015	3
34200	1.014	3
34300	1.014	3
34400	1.014	3
34500	1.015	3
34600	1.014	3
34700	1.013	3
34800	1.012	3
34900	1.012	3
35000	1.012	3
35100	1.012	3
35200	1.012	3
35300	1.012	3
35400	1.012	3
35500	1.011	3
35600	1.010	3
35700	1.010	3
35800	1.009	3
35900	1.009	3
36000	1.009	3

```
36100          1.009          95
36200          1.009          95
36300          1.009          95
36400          1.009          95
36500          1.009          95
36600          1.009          3
36700          1.010          3
36800          1.010          3
36900          1.009          3
37000          1.009          3
37100          1.009          3
37200          1.008          95
37300          1.009          95
37400          1.008          95
37500          1.008          95
37600          1.008          95
37700          1.007          95
37800          1.007          17
37900          1.007          17
38000          1.007          17
38100          1.008          17
38200          1.008          17
38300          1.009          17
38400          1.010          17
38500          1.010          17
38600          1.009          17
38700          1.009          17
38800          1.009          17
38900          1.009          17
39000          1.008          17
39100          1.008          17
39200          1.008          17
39300          1.009          17
39400          1.008          17
39500          1.009          74
39600          1.010          74
39700          1.009          74
39800          1.010          74
39900          1.010          74
40000          1.011          74
40100          1.012          74
40200          1.012          74
40300          1.012          74
40400          1.012          74
40500          1.013          74
40600          1.013          74
40700          1.013          74
40800          1.013          74
40900          1.014          74
41000          1.014          74
41100          1.015          74
41200          1.015          74
41300          1.016          74
41400          1.016          74
41500          1.016          74
41600          1.016          74
41700          1.017          74
41800          1.017          74
41900          1.017          74
42000          1.016          74
42100          1.016          74
42200          1.016          74
42300          1.017          74
42400          1.018          74
42500          1.018          74
42600          1.018          74
42700          1.018          74
42800          1.018          74
42900          1.019          74
43000          1.020          74
43100          1.021          74
43200          1.023          74
43300          1.024          74
43400          1.026          74
```

43500	1.027	74
43600	1.029	74
43700	1.029	74
43800	1.028	74
43900	1.028	74
44000	1.027	74
44100	1.025	74
44200	1.024	74
44300	1.023	74
44400	1.021	74
44500	1.020	74
44600	1.020	74
44700	1.018	74
44800	1.017	74
44900	1.018	74
45000	1.018	74
45100	1.019	74
45200	1.019	74
45300	1.019	74
45400	1.020	74
45500	1.020	74
45600	1.019	74
45700	1.018	74
45800	1.018	74
45900	1.017	74
46000	1.016	74
46100	1.016	74
46200	1.016	74
46300	1.016	74
46400	1.016	74
46500	1.015	74
46600	1.014	74
46700	1.014	74
46800	1.014	74
46900	1.013	74
47000	1.012	74
47100	1.012	74
47200	1.013	74
47300	1.014	74
47400	1.013	74
47500	1.012	74
47600	1.011	74
47700	1.011	74
47800	1.011	74
47900	1.012	74
48000	1.012	74
48100	1.013	74
48200	1.013	74
48300	1.012	74
48400	1.011	74
48500	1.011	74
48600	1.011	3
48700	1.012	3
48800	1.012	3
48900	1.012	3
49000	1.013	3
49100	1.012	3
49200	1.012	3
49300	1.011	3
49400	1.011	3
49500	1.012	3
49600	1.012	3
49700	1.012	3
49800	1.012	3
49900	1.012	3
50000	1.013	3
50100	1.013	3
50200	1.014	3
50300	1.015	3
50400	1.016	3
50500	1.016	3
50600	1.016	3
50700	1.016	3
50800	1.016	3

```
50900            1.017            3
51000            1.017            3
51100            1.017            3
51200            1.017            3
51300            1.017            3
51400            1.018            3
51500            1.017            3
51600            1.017            3
51700            1.017            3
51800            1.017            3
51900            1.017            3
52000            1.016            3
52100            1.016            3
52200            1.016            3
52300            1.016            3
52400            1.016            3
52500            1.016            3
52600            1.017            3
52700            1.017            3
52800            1.017            3
52900            1.017            3
53000            1.017            3
53100            1.018            3
53200            1.019            3
53300            1.020            3
53400            1.020            3
53500            1.019            3
53600            1.019            3
53700            1.018            3
53800            1.017            3
53900            1.016            3
54000            1.016            3
54100            1.015            3
54200            1.016            3
54300            1.017            3
54400            1.018            3
54500            1.018            3
54600            1.018            3
54700            1.018            3
54800            1.017            3
54900            1.016            3
55000            1.015            3
55100            1.015            3
55200            1.015            3
55300            1.015            3
55400            1.015            3
55500            1.016            3
55600            1.017            3
55700            1.018            3
55800            1.018            3
55900            1.018            3
56000            1.018            56
56100            1.019            56
56200            1.019            56
56300            1.018            56
56400            1.018            3
56500            1.019            3
56600            1.019            3
56700            1.020            3
56800            1.021            3
56900            1.022            3
57000            1.021            3
57100            1.021            3
57200            1.021            3
57300            1.021            3
57400            1.019            3
57500            1.018            56
57600            1.018            56
57700            1.018            3
57800            1.019            3
57900            1.021            3
58000            1.022            3
58100            1.021            3
58200            1.022            3
```

```
58300          1.024          3
58400          1.025          3
58500          1.026          3
58600          1.027          3
58700          1.026          3
58800          1.027          3
58900          1.028          3
59000          1.028          3
59100          1.029          3
59200          1.029          3
59300          1.028          3
59400          1.027          3
59500          1.027          3
59600          1.026          3
59700          1.026          3
59800          1.027          3
59900          1.028          3
60000          1.029          3
60100          1.031          3
60200          1.032          3
60300          1.031          3
60400          1.031          3
60500          1.031          3
60600          1.031          3
60700          1.031          3
60800          1.029          3
60900          1.028          3
61000          1.028          3
61100          1.028          3
61200          1.028          3
61300          1.028          3
61400          1.029          3
61500          1.028          3
61600          1.029          3
61700          1.030          3
61800          1.030          3
61900          1.031          3
62000          1.031          3
62100          1.030          3
62200          1.029          3
62300          1.030          3
62400          1.029          3
62500          1.029          3
62600          1.028          3
62700          1.027          3
62800          1.028          3
62900          1.027          3
63000          1.027          3
63100          1.027          3
63200          1.028          3
63300          1.028          3
63400          1.028          3
63500          1.028          3
63600          1.027          3
63700          1.026          3
63800          1.026          3
63900          1.026          3
64000          1.028          3
64100          1.029          3
64200          1.031          3
64300          1.032          3
64400          1.032          3
64500          1.032          3
64600          1.032          3
64700          1.032          3
64800          1.032          3
64900          1.032          3
65000          1.032          3
65100          1.032          3
65200          1.032          3
65300          1.032          3
65400          1.033          3
65500          1.033          3
65600          1.033          3
```

65700	1.033	3
65800	1.034	3
65900	1.037	3
66000	1.039	3
66100	1.040	3
66200	1.040	3
66300	1.041	3
66400	1.041	3
66500	1.041	3
66600	1.041	3
66700	1.041	3
66800	1.040	3
66900	1.038	3
67000	1.037	3
67100	1.036	3
67200	1.037	3
67300	1.037	3
67400	1.037	3
67500	1.037	3
67600	1.037	3
67700	1.036	3
67800	1.035	3
67900	1.036	3
68000	1.036	3
68100	1.037	3
68200	1.037	3
68300	1.037	3
68400	1.037	3
68500	1.037	3
68600	1.038	3
68700	1.038	3
68800	1.037	3
68900	1.038	3
69000	1.038	3
69100	1.037	3
69200	1.037	3
69300	1.037	3
69400	1.037	3
69500	1.037	3
69600	1.037	3
69700	1.037	3
69800	1.038	3
69900	1.038	3
70000	1.038	3
70100	1.039	3
70200	1.039	3
70300	1.039	3
70400	1.039	3
70500	1.039	3
70600	1.037	3
70700	1.037	3
70800	1.036	3
70900	1.036	3
71000	1.035	3
71100	1.035	3
71200	1.033	3
71300	1.032	3
71400	1.031	3
71500	1.031	3
71600	1.031	3
71700	1.031	3
71800	1.030	3
71900	1.030	3
72000	1.030	3
72100	1.031	3
72200	1.031	3
72300	1.032	3
72400	1.034	3
72500	1.035	3
72600	1.036	3
72700	1.038	3
72800	1.038	3
72900	1.038	3
73000	1.039	3

73100	1.039	3
73200	1.040	3
73300	1.040	3
73400	1.040	3
73500	1.040	3
73600	1.039	3
73700	1.038	3
73800	1.037	3
73900	1.036	3
74000	1.036	3
74100	1.036	3
74200	1.035	3
74300	1.035	3
74400	1.035	3
74500	1.035	3
74600	1.036	3
74700	1.036	3
74800	1.036	3
74900	1.036	3
75000	1.035	3
75100	1.035	3
75200	1.036	3
75300	1.036	3
75400	1.035	3
75500	1.035	3
75600	1.034	3
75700	1.034	3
75800	1.033	3
75900	1.033	3
76000	1.032	3
76100	1.032	3
76200	1.031	3
76300	1.032	3
76400	1.032	3
76500	1.032	3
76600	1.031	3
76700	1.031	3
76800	1.030	3
76900	1.029	3
77000	1.026	3
77100	1.023	3
77200	1.022	3
77300	1.022	3
77400	1.021	3
77500	1.020	3
77600	1.019	3
77700	1.019	3
77800	1.019	3
77900	1.019	3
78000	1.019	3
78100	1.019	3
78200	1.019	3
78300	1.019	3
78400	1.019	3
78500	1.020	3
78600	1.021	3
78700	1.021	3
78800	1.022	3
78900	1.022	3
79000	1.022	3
79100	1.022	3
79200	1.022	3
79300	1.022	3
79400	1.021	3
79500	1.021	3
79600	1.021	3
79700	1.020	3
79800	1.020	3
79900	1.020	3
80000	1.020	3
80100	1.020	3
80200	1.020	3
80300	1.019	3
80400	1.020	3

80500	1.020	3
80600	1.020	3
80700	1.020	3
80800	1.020	3
80900	1.020	3
81000	1.020	3
81100	1.020	3
81200	1.020	3
81300	1.020	3
81400	1.020	3
81500	1.021	3
81600	1.021	3
81700	1.021	3
81800	1.022	3
81900	1.023	3
82000	1.024	3
82100	1.025	3
82200	1.027	3
82300	1.028	3
82400	1.029	3
82500	1.030	3
82600	1.031	3
82700	1.031	3
82800	1.030	3
82900	1.030	3
83000	1.030	3
83100	1.030	3
83200	1.030	3
83300	1.030	3
83400	1.031	3
83500	1.031	3
83600	1.031	3
83700	1.031	3
83800	1.030	3
83900	1.029	3
84000	1.029	3
84100	1.029	3
84200	1.029	3
84300	1.030	3
84400	1.030	3
84500	1.030	3
84600	1.031	3
84700	1.031	3
84800	1.031	3
84900	1.030	3
85000	1.030	3
85100	1.029	3
85200	1.029	3
85300	1.028	3
85400	1.028	3
85500	1.027	3
85600	1.027	3
85700	1.026	3
85800	1.026	3
85900	1.026	3
86000	1.025	3
86100	1.025	3
86200	1.024	3
86300	1.024	3
86400	1.025	3
86500	1.025	3
86600	1.025	3
86700	1.025	3
86800	1.025	3
86900	1.024	3
87000	1.024	3
87100	1.024	3
87200	1.023	3
87300	1.023	3
87400	1.021	3
87500	1.021	3
87600	1.020	3
87700	1.020	3
87800	1.020	3

```
87900            1.021            3
88000            1.021            3
88100            1.021            3
88200            1.021            3
88300            1.021            3
88400            1.021            3
88500            1.020            3
88600            1.020            3
88700            1.020            3
88800            1.019            3
88900            1.019            3
89000            1.019            3
89100            1.019            3
89200            1.018            3
89300            1.019            3
89400            1.019            3
89500            1.018            3
89600            1.018            3
89700            1.018            3
89800            1.017            3
89900            1.016            3
90000            1.015            3
90100            1.015            3
90200            1.015            3
90300            1.015            3
90400            1.015            3
90500            1.015            3
90600            1.015            3
90700            1.015            3
90800            1.016            3
90900            1.015            3
91000            1.015            3
91100            1.016            3
91200            1.016            3
91300            1.016            3
91400            1.016            3
91500            1.015            3
91600            1.015            3
91700            1.015            3
91800            1.015            3
91900            1.016            3
92000            1.016            3
92100            1.016            3
92200            1.016            3
92300            1.016            3
92400            1.016            3
92500            1.016            3
92600            1.016            3
92700            1.016            3
92800            1.016            3
92900            1.015            3
93000            1.015            3
93100            1.015            3
93200            1.015            3
93300            1.015            3
93400            1.015            3
93500            1.015            3
93600            1.015            3
93700            1.015            3
93800            1.016            3
93900            1.016            3
94000            1.017            3
94100            1.017            3
94200            1.017            3
94300            1.017            3
94400            1.017            3
94500            1.017            3
94600            1.018            3
94700            1.018            3
94800            1.019            3
94900            1.020            3
95000            1.020            3
95100            1.020            3
95200            1.020            3
```

```
95300           1.019           3
95400           1.019           3
95500           1.019           3
95600           1.019           3
95700           1.019           3
95800           1.019           3
95900           1.019           3
96000           1.018           3
96100           1.018           3
96200           1.018           3
96300           1.018           3
96400           1.019           3
96500           1.020           3
96600           1.021           3
96700           1.021           3
96800           1.022           3
96900           1.022           3
97000           1.022           3
97100           1.022           3
97200           1.022           3
97300           1.021           3
97400           1.021           3
97500           1.021           3
97600           1.021           3
97700           1.021           3
97800           1.021           3
97900           1.021           3
98000           1.022           3
98100           1.021           3
98200           1.021           3
98300           1.021           3
98400           1.022           3
98500           1.022           3
98600           1.022           3
98700           1.022           3
98800           1.022           3
98900           1.022           3
99000           1.022           3
99100           1.022           3
99200           1.022           3
99300           1.022           3
99400           1.022           3
99500           1.021           3
99600           1.021           3
99700           1.021           3
99800           1.021           3
99900           1.020           3
100000          1.020           3
100100          1.020           3
100200          1.020           3
100300          1.020           3
100400          1.020           3
100500          1.020           3
100600          1.019           3
100700          1.019           3
100800          1.019           3
100900          1.019           3
101000          1.018           3
101100          1.018           3
101200          1.018           3
101300          1.018           3
101400          1.019           3
101500          1.018           3
101600          1.018           3
101700          1.018           3
101800          1.018           3
101900          1.018           3
102000          1.017           3
102100          1.017           3
102200          1.017           3
102300          1.016           3
102400          1.016           3
102500          1.016           3
102600          1.016           3
```

```
102700           1.016           3
102800           1.016           3
102900           1.016           3
103000           1.016           3
103100           1.017           3
103200           1.018           3
103300           1.019           3
103400           1.020           3
103500           1.020           3
103600           1.020           3
103700           1.020           3
103800           1.020           3
103900           1.020           3
104000           1.020           3
104100           1.019           3
104200           1.019           3
104300           1.019           3
104400           1.020           3
104500           1.020           3
104600           1.020           3
104700           1.020           3
104800           1.020           3
104900           1.020           3
105000           1.020           3
105100           1.020           3
105200           1.020           3
105300           1.020           3
105400           1.020           3
105500           1.020           3
105600           1.020           3
105700           1.020           3
105800           1.020           3
105900           1.020           3
106000           1.020           3
106100           1.019           3
106200           1.019           3
106300           1.019           3
106400           1.019           3
106500           1.019           3
106600           1.019           3
106700           1.018           3
106800           1.018           3
106900           1.018           3
107000           1.018           3
107100           1.018           3
107200           1.018           3
107300           1.018           3
107400           1.018           3
107500           1.018           3
107600           1.018           3
107700           1.018           3
107800           1.018           3
107900           1.018           3
108000           1.018           3
108100           1.017           3
108200           1.017           3
108300           1.017           3
108400           1.017           3
108500           1.017           3
108600           1.016           3
108700           1.015           3
108800           1.015           3
108900           1.015           3
109000           1.015           3
109100           1.015           3
109200           1.015           3
109300           1.015           3
109400           1.015           3
109500           1.015           3
109600           1.015           3
109700           1.015           3
109800           1.015           3
109900           1.015           3
110000           1.015           3
```

```
110100          1.015             3
110200          1.016             3
110300          1.016             3
110400          1.015             3
110500          1.015             3
110600          1.015             3
110700          1.015             3
110800          1.015             3
110900          1.015             3
111000          1.015             3
111100          1.015             3
111200          1.015             3
111300          1.015             3
111400          1.015             3
111500          1.015             3
111600          1.015             3
111700          1.015             3
111800          1.012             3
111900          1.011             3
112000          1.011             3
112100          1.011             3
112200          1.011             3
112300          1.011             3
112400          1.011             3
112500          1.011             3
112600          1.011             3
112700          1.011             3
112800          1.011             3
112900          1.011             3
113000          1.011             3
113100          1.011             3
113200          1.011             3
113300          1.011             3
113400          1.011             3
113500          1.011             3
113600          1.011             3
113700          1.010             3
113800          1.010             3
113900          1.010             3
114000          1.010             3
114100          1.011             3
114200          1.011             3
114300          1.011             3
114400          1.011             3
114500          1.011             3
114600          1.011             3
114700          1.011             3
114800          1.011             3
114900          1.011             3
115000          1.011             3
115100          1.011             3
115200          1.011             3
115300          1.011             3
115400          1.011             3
115500          1.010             3
115600          1.010             3
115700          1.009             3
115800          1.009             3
115900          1.008             3
116000          1.008             3
116100          1.007             3
116200          1.007             3
116300          1.007             3
116400          1.006             3
116500          1.005             68
116600          1.006             68
116700          1.006             68
116800          1.005             68
116900          1.005             68
117000          1.005             68
117100          1.005             68
117200          1.005             68
117300          1.005             68
117400          1.005             68
```

117500	1.006	68
117600	1.006	68
117700	1.006	68
117800	1.006	68
117900	1.006	68
118000	1.006	68
118100	1.006	68
118200	1.006	68
118300	1.006	68
118400	1.006	68
118500	1.006	68
118600	1.006	68
118700	1.006	68
118800	1.006	68
118900	1.006	68
119000	1.006	68
119100	1.006	68
119200	1.006	68
119300	1.006	68
119400	1.006	68
119500	1.006	68
119600	1.006	68
119700	1.006	68
119800	1.006	68
119900	1.006	68
120000	1.006	68
120100	1.006	68
120200	1.006	68
120300	1.006	68
120400	1.006	68
120500	1.006	68
120600	1.007	68
120700	1.007	68
120800	1.007	68
120900	1.007	68
121000	1.007	68
121100	1.007	68
121200	1.007	68
121300	1.007	68
121400	1.007	68
121500	1.008	68
121600	1.008	68
121700	1.008	68
121800	1.007	68
121900	1.007	68
122000	1.008	68
122100	1.008	68
122200	1.008	68
122300	1.008	68
122400	1.007	68
122500	1.007	68
122600	1.007	68
122700	1.007	68
122800	1.007	68
122900	1.007	68
123000	1.007	68
123100	1.007	68
123200	1.007	68
123300	1.007	68
123400	1.007	68
123500	1.007	68
123600	1.007	68
123700	1.007	68
123800	1.007	68
123900	1.007	68
124000	1.007	68
124100	1.007	68
124200	1.007	68
124300	1.007	68
124400	1.007	68
124500	1.007	68
124600	1.007	68
124700	1.007	68
124800	1.007	68

```
     124900              1.008              68
     125000              1.008              68
     125100              1.008              68
     125200              1.009              68
     125300              1.009              68
     125400              1.009              68
     125500              1.008              68
     125600              1.008              68
     125700              1.008              68
     125800              1.008              68
     125900              1.007              68
     126000              1.007              68
     126100              1.008              68
     126200              1.008              68
     126300              1.008              68
     126400              1.008              68
     126500              1.008              68
     126600              1.008              68
     126700              1.008              68
     126800              1.008              68
     126900              1.008              68
     127000              1.009              68
     127100              1.009              68
     127200              1.009              68
     127300              1.010              68
     127400              1.010              68
     127500              1.009              68
     127600              1.010              68
     127700              1.010              68
     127800              1.010              68
     127900              1.010              68
     128000              1.010              68
     128100              1.010              68
     128200              1.010              68
     128300              1.010              68
     128400              1.010              68
     128500              1.010              68
     128600              1.010              68
     128700              1.009              68
     128800              1.009              68
     128900              1.009              68
     129000              1.009              68
     129100              1.010              68
     129200              1.010              68
     129300              1.009              68
     129400              1.009              68
     129500              1.008              68
     129600              1.008              68
     129700              1.008              68
     129800              1.008              68
     129900              1.008              68
     130000              1.009              68
     130100              1.008              68
     130200              1.008              68
     130300              1.008              68
     130400              1.008              68
     130500              1.008              68
     130600              1.008              68
     130700              1.008              68
     130800              1.008              68
     130900              1.008              68
     131000              1.008              68
     131100              1.008              68
     131200              1.008              68
     131300              1.008              68
     131400              1.008              68
     131500              1.008              68
     131600              1.008              68
     131700              1.008              68
     131800              1.008              68
     131900              1.008              68
     132000              1.008              68
     132100              1.008              68
     132200              1.008              68
```

132300	1.008	68
132400	1.008	68
132500	1.008	68
132600	1.008	68
132700	1.008	68
132800	1.008	68
132900	1.008	68
133000	1.008	68
133100	1.008	68
133200	1.008	68
133300	1.009	68
133400	1.009	68
133500	1.008	68
133600	1.008	68
133700	1.008	68
133800	1.008	68
133900	1.008	68
134000	1.008	68
134100	1.008	68
134200	1.008	68
134300	1.008	68
134400	1.008	68
134500	1.008	68
134600	1.008	68
134700	1.009	68
134800	1.009	68
134900	1.009	68
135000	1.009	68
135100	1.009	68
135200	1.009	68
135300	1.009	68
135400	1.008	68
135500	1.009	68
135600	1.009	68
135700	1.009	68
135800	1.009	68
135900	1.009	68
136000	1.009	68
136100	1.009	68
136200	1.009	68
136300	1.009	68
136400	1.009	68
136500	1.009	68
136600	1.008	68
136700	1.008	68
136800	1.008	68
136900	1.008	68
137000	1.008	68
137100	1.008	68
137200	1.008	68
137300	1.008	68
137400	1.008	68
137500	1.008	68
137600	1.008	68
137700	1.008	68
137800	1.008	68
137900	1.008	68
138000	1.008	68
138100	1.008	68
138200	1.008	74
138300	1.008	74
138400	1.008	74
138500	1.008	74
138600	1.008	68
138700	1.008	74
138800	1.008	74
138900	1.008	74
139000	1.008	74
139100	1.007	74
139200	1.007	74
139300	1.007	74
139400	1.007	74
139500	1.007	74
139600	1.007	74

```
     139700           1.007             68
     139800           1.007             68
     139900           1.007             68
     140000           1.008             68
     140100           1.008             68
     140200           1.008             68
     140300           1.007             68
     140400           1.008             68
     140500           1.008             74
     140600           1.008             74
     140700           1.008             74
     140800           1.008             74
     140900           1.008             74
     141000           1.008             74
     141100           1.008             74
     141200           1.008             74
     141300           1.008             74
     141400           1.008             74
     141500           1.008             74
     141600           1.008             68
     141700           1.008             68
     141800           1.008             68
     141900           1.008             68
     142000           1.008             68
     142100           1.008             68
     142200           1.008             68
     142300           1.008             68
     142400           1.007             68
     142500           1.007             68
     142600           1.007             68
     142700           1.007             74
     142800           1.007             74
     142900           1.007             74
     143000           1.008             74
     143100           1.008             74
     143200           1.008             74
     143300           1.008             74
     143400           1.008             74
     143500           1.008             74
     143600           1.008             74
     143700           1.009             74
     143800           1.009             74
     143900           1.009             74
     144000           1.009             74
     144100           1.009             74
     144200           1.009             74
     144300           1.010             74
     144400           1.009             74
     144500           1.009             74
     144600           1.009             74
     144700           1.009             74
     144800           1.009             74
     144900           1.009             74
     145000           1.009             74
     145100           1.009             74
     145200           1.009             74
     145300           1.008             74
     145400           1.008             74
     145500           1.008             74
     145600           1.008             74
     145700           1.008             74
     145800           1.007             74
     145900           1.007             74
     146000           1.007             74
     146100           1.007             74
     146200           1.007             74
     146300           1.007             74
     146400           1.007             74
     146500           1.007             74
     146600           1.007             74
     146700           1.007             74
     146800           1.008             74
     146900           1.008             74
     147000           1.007             74
```

147100	1.007	74
147200	1.008	74
147300	1.008	74
147400	1.008	74
147500	1.008	74
147600	1.008	74
147700	1.008	74
147800	1.008	74
147900	1.007	74
148000	1.007	74
148100	1.007	74
148200	1.007	74
148300	1.007	74
148400	1.007	74
148500	1.007	74
148600	1.007	74
148700	1.007	74
148800	1.007	74
148900	1.007	74
149000	1.007	74
149100	1.007	74
149200	1.007	74
149300	1.007	74
149400	1.007	74
149500	1.007	74
149600	1.007	74
149700	1.007	74
149800	1.007	74
149900	1.007	74
150000	1.007	74
150100	1.007	74
150200	1.006	74
150300	1.007	19
150400	1.007	19
150500	1.007	19
150600	1.007	19
150700	1.007	19
150800	1.007	19
150900	1.007	74
151000	1.007	74
151100	1.007	74
151200	1.007	74
151300	1.007	19
151400	1.007	19
151500	1.007	74
151600	1.007	74
151700	1.007	74
151800	1.007	74
151900	1.007	74
152000	1.007	19
152100	1.007	19
152200	1.007	19
152300	1.007	19
152400	1.007	19
152500	1.007	19
152600	1.007	19
152700	1.007	19
152800	1.007	19
152900	1.007	19
153000	1.007	19
153100	1.007	19
153200	1.007	19
153300	1.006	19
153400	1.007	19
153500	1.007	19
153600	1.007	19
153700	1.007	74
153800	1.007	74
153900	1.007	74
154000	1.007	74
154100	1.007	74
154200	1.007	74
154300	1.007	74
154400	1.007	74

154500	1.007	74
154600	1.007	74
154700	1.007	74
154800	1.007	74
154900	1.007	74
155000	1.007	74
155100	1.007	74
155200	1.007	74
155300	1.007	74
155400	1.007	74
155500	1.008	74
155600	1.008	74
155700	1.008	74
155800	1.008	74
155900	1.008	74
156000	1.008	74
156100	1.007	74
156200	1.008	74
156300	1.008	74
156400	1.008	74
156500	1.008	74
156600	1.007	74
156700	1.007	74
156800	1.007	74
156900	1.007	74
157000	1.007	74
157100	1.007	74
157200	1.007	74
157300	1.007	74
157400	1.007	74
157500	1.007	74
157600	1.007	74
157700	1.008	74
157800	1.008	74
157900	1.008	74
158000	1.008	74
158100	1.008	74
158200	1.008	74
158300	1.008	74
158400	1.008	74
158500	1.008	74
158600	1.008	74
158700	1.008	74
158800	1.008	74
158900	1.008	74
159000	1.008	74
159100	1.008	74
159200	1.008	74
159300	1.008	74
159400	1.008	74
159500	1.008	74
159600	1.008	74
159700	1.008	74
159800	1.008	74
159900	1.008	74
160000	1.008	74
160100	1.008	74
160200	1.008	74
160300	1.007	74
160400	1.007	74
160500	1.007	74
160600	1.007	74
160700	1.007	74
160800	1.007	74
160900	1.007	74
161000	1.007	74
161100	1.007	74
161200	1.007	74
161300	1.007	74
161400	1.007	74
161500	1.007	74
161600	1.007	74
161700	1.007	74
161800	1.007	74

161900	1.007	74
162000	1.007	74
162100	1.006	74
162200	1.007	74
162300	1.006	74
162400	1.006	74
162500	1.006	74
162600	1.006	74
162700	1.006	74
162800	1.006	74
162900	1.006	74
163000	1.006	74
163100	1.006	74
163200	1.006	74
163300	1.006	74
163400	1.006	74
163500	1.006	74
163600	1.006	74
163700	1.006	74
163800	1.006	74
163900	1.006	74
164000	1.006	74
164100	1.006	74
164200	1.006	74
164300	1.006	74
164400	1.006	74
164500	1.006	74
164600	1.007	74
164700	1.006	74
164800	1.006	74
164900	1.006	74
165000	1.006	74
165100	1.006	74
165200	1.006	74
165300	1.006	74
165400	1.006	74
165500	1.006	74
165600	1.006	74
165700	1.006	74
165800	1.006	74
165900	1.006	74
166000	1.006	74
166100	1.006	74
166200	1.006	74
166300	1.006	74
166400	1.007	74
166500	1.007	74
166600	1.007	74
166700	1.007	74
166800	1.007	74
166900	1.007	74
167000	1.007	74
167100	1.007	74
167200	1.007	74
167300	1.007	74
167400	1.007	74
167500	1.007	74
167600	1.007	74
167700	1.007	74
167800	1.007	74
167900	1.007	74
168000	1.007	74
168100	1.007	74
168200	1.007	74
168300	1.007	74
168400	1.007	74
168500	1.007	74
168600	1.006	74
168700	1.006	74
168800	1.006	74
168900	1.006	74
169000	1.007	74
169100	1.006	74
169200	1.006	74

```
169300          1.006          74
169400          1.006          74
169500          1.006          74
169600          1.006          74
169700          1.006          74
169800          1.006          74
169900          1.005          74
170000          1.006          74
170100          1.006          74
170200          1.006          74
170300          1.006          74
170400          1.006          74
170500          1.006          74
170600          1.006          74
170700          1.006          74
170800          1.006          74
170900          1.006          74
171000          1.006          74
171100          1.006          74
171200          1.006          74
171300          1.006          74
171400          1.007          74
171500          1.007          74
171600          1.007          74
171700          1.007          74
171800          1.007          74
171900          1.008          74
172000          1.008          74
172100          1.008          74
172200          1.009          74
172300          1.008          74
172400          1.008          74
172500          1.008          74
172600          1.008          74
172700          1.008          74
172800          1.008          74
172900          1.008          74
173000          1.008          74
173100          1.008          74
173200          1.008          74
173300          1.008          74
173400          1.008          74
173500          1.008          74
173600          1.008          74
173700          1.009          74
173800          1.008          74
173900          1.008          74
174000          1.008          74
174100          1.008          74
174200          1.009          74
174300          1.009          74
174400          1.009          74
174500          1.009          74
174600          1.009          74
174700          1.009          74
174800          1.009          74
174900          1.009          74
175000          1.009          74
175100          1.009          74
175200          1.009          74
175300          1.009          74
175400          1.010          74
175500          1.010          74
175600          1.010          74
175700          1.010          74
175800          1.010          74
175900          1.010          74
176000          1.010          74
176100          1.010          74
176200          1.010          74
176300          1.010          74
176400          1.010          74
176500          1.010          74
176600          1.009          74
```

```
     176700          1.009              74
     176800          1.010              74
     176900          1.010              74
     177000          1.010              74
     177100          1.010              74
     177200          1.010              74
     177300          1.010              74
     177400          1.010              74
     177500          1.010              74
     177600          1.010              74
     177700          1.010              74
     177800          1.010              74
     177900          1.010              74
     178000          1.010              74
     178100          1.010              74
     178200          1.010              74
     178300          1.009              74
     178400          1.009              74
     178500          1.009              74
     178600          1.009              74
     178700          1.008              74
     178800          1.008              74
     178900          1.008              74
     179000          1.008              74
     179100          1.008              74
     179200          1.008              74
     179300          1.008              74
     179400          1.008              74
     179500          1.008              74
     179600          1.008              74
     179700          1.007              74
     179800          1.007              74
     179900          1.007              74
     180000          1.007              74
     180100          1.007              74
     180200          1.007              74
     180300          1.007              74
     180400          1.007              74
     180500          1.007              74
     180600          1.008              74
     180700          1.008              74
     180800          1.007              74
     180900          1.007              74
     181000          1.008              74
     181100          1.008              74
     181200          1.008              74
     181300          1.008              74
     181400          1.008              74
     181500          1.008              74
     181600          1.008              74
     181700          1.008              74
     181800          1.009              74
     181900          1.009              74
     182000          1.009              74
     182100          1.009              74
     182200          1.009              74
     182300          1.009              74
     182400          1.009              74
     182500          1.009              74
     182600          1.009              74
     182700          1.009              74
     182800          1.009              74
     182900          1.009              74
     183000          1.009              74
     183100          1.009              74
     183200          1.009              74
     183300          1.009              74
     183400          1.009              74
     183500          1.009              74
     183600          1.009              74
     183700          1.009              74
     183800          1.010              74
     183900          1.010              74
     184000          1.010              74
```

184100	1.010	74
184200	1.010	74
184300	1.009	74
184400	1.009	74
184500	1.009	74
184600	1.009	74
184700	1.009	74
184800	1.009	74
184900	1.009	74
185000	1.009	74
185100	1.009	74
185200	1.009	74
185300	1.009	74
185400	1.009	74
185500	1.009	74
185600	1.009	74
185700	1.009	74
185800	1.009	74
185900	1.009	74
186000	1.009	74
186100	1.009	74
186200	1.009	74
186300	1.009	74
186400	1.009	74
186500	1.009	74
186600	1.009	74
186700	1.009	74
186800	1.009	74
186900	1.009	74
187000	1.009	74
187100	1.009	74
187200	1.009	74
187300	1.009	74
187400	1.009	74
187500	1.009	74
187600	1.009	74
187700	1.009	74
187800	1.009	74
187900	1.009	74
188000	1.009	74
188100	1.009	74
188200	1.009	74
188300	1.009	74
188400	1.009	74
188500	1.009	74
188600	1.009	74
188700	1.009	74
188800	1.009	74
188900	1.009	74
189000	1.009	74
189100	1.009	74
189200	1.009	74
189300	1.009	74
189400	1.009	74
189500	1.009	74
189600	1.009	74
189700	1.009	74
189800	1.009	74
189900	1.009	74
190000	1.009	74
190100	1.009	74
190200	1.009	74
190300	1.009	74
190400	1.009	74
190500	1.009	74
190600	1.009	74
190700	1.009	74
190800	1.009	74
190900	1.009	74
191000	1.009	74
191100	1.009	74
191200	1.009	74
191300	1.009	74
191400	1.009	74

```
191500           1.009            74
191600           1.009            74
191700           1.009            74
191800           1.009            74
191900           1.009            74
192000           1.009            74
192100           1.009            74
192200           1.009            74
192300           1.009            74
192400           1.009            74
192500           1.009            74
192600           1.009            74
192700           1.008            74
192800           1.008            74
192900           1.008            74
193000           1.008            74
193100           1.008            74
193200           1.008            74
193300           1.007            74
193400           1.007            74
193500           1.008            74
193600           1.007            74
193700           1.007            74
193800           1.007            74
193900           1.007            74
194000           1.007            74
194100           1.007            74
194200           1.007            74
194300           1.007            74
194400           1.007            74
194500           1.007            74
194600           1.007            74
194700           1.006            74
194800           1.006            74
194900           1.006            74
195000           1.007            74
195100           1.007            74
195200           1.006            74
195300           1.006            74
195400           1.006            74
195500           1.006            74
195600           1.006            74
195700           1.006            74
195800           1.006            74
195900           1.006            74
196000           1.006            74
196100           1.006            74
196200           1.006            74
196300           1.006            74
196400           1.006            74
196500           1.006            74
196600           1.006            74
196700           1.006            74
196800           1.006            74
196900           1.005            74
197000           1.006            74
197100           1.006            74
197200           1.006            74
197300           1.006            74
197400           1.006            74
197500           1.006            74
197600           1.006            74
197700           1.006            74
197800           1.006            74
197900           1.006            74
198000           1.006            74
198100           1.006            74
198200           1.006            74
198300           1.006            74
198400           1.006            74
198500           1.006            74
198600           1.006            74
198700           1.006            74
198800           1.006            74
```

```
198900          1.006           74
199000          1.006           74
199100          1.006           74
199200          1.006           74
199300          1.006           74
199400          1.006           74
199500          1.006           74
199600          1.006           74
199700          1.006           74
199800          1.006           74
199900          1.006           74
200000          1.006           74
200100          1.006           74
200200          1.006           74
200300          1.006           74
200400          1.006           74
200500          1.006           74
200600          1.006           74
200700          1.006           74
200800          1.006           74
200900          1.006           74
201000          1.006           74
201100          1.006           74
201200          1.006           74
201300          1.006           74
201400          1.006           74
201500          1.006           74
201600          1.006           74
201700          1.006           74
201800          1.006           74
201900          1.006           74
202000          1.006           74
202100          1.006           74
202200          1.006           74
202300          1.006           74
202400          1.006           74
202500          1.006           74
202600          1.007           74
202700          1.007           74
202800          1.007           74
202900          1.007           74
203000          1.007           74
203100          1.007           74
203200          1.006           74
203300          1.006           74
203400          1.007           74
203500          1.006           74
203600          1.007           74
203700          1.007           74
203800          1.007           74
203900          1.007           74
204000          1.007           74
204100          1.007           74
204200          1.007           74
204300          1.006           74
204400          1.007           74
204500          1.006           74
204600          1.006           74
204700          1.006           74
204800          1.006           74
204900          1.006           74
205000          1.006           74
205100          1.006           74
205200          1.006           74
205300          1.006           74
205400          1.006           74
205500          1.006           74
205600          1.006           74
205700          1.006           74
205800          1.006           74
205900          1.006           74
206000          1.006           74
206100          1.006           74
206200          1.006           74
```

206300	1.006	74
206400	1.006	74
206500	1.006	74
206600	1.006	74
206700	1.006	74
206800	1.006	74
206900	1.006	74
207000	1.006	74
207100	1.006	74
207200	1.006	74
207300	1.006	74
207400	1.006	74
207500	1.005	74
207600	1.005	74
207700	1.006	74
207800	1.005	74
207900	1.005	74
208000	1.005	74
208100	1.005	74
208200	1.005	74
208300	1.005	74
208400	1.005	74
208500	1.005	74
208600	1.005	74
208700	1.005	74
208800	1.005	74
208900	1.005	74
209000	1.005	74
209100	1.005	74
209200	1.005	74
209300	1.006	74
209400	1.006	74
209500	1.006	74
209600	1.006	74
209700	1.006	74
209800	1.006	74
209900	1.006	74
210000	1.006	74
210100	1.006	74
210200	1.005	74
210300	1.005	74
210400	1.005	74
210500	1.005	74
210600	1.005	74
210700	1.005	74
210800	1.006	74
210900	1.006	74
211000	1.006	74
211100	1.006	74
211200	1.006	74
211300	1.006	74
211400	1.006	74
211500	1.006	74
211600	1.006	74
211700	1.006	74
211800	1.006	74
211900	1.006	74
212000	1.006	74
212100	1.006	74
212200	1.006	74
212300	1.006	74
212400	1.007	74
212500	1.007	74
212600	1.006	74
212700	1.006	74
212800	1.006	74
212900	1.006	74
213000	1.006	74
213100	1.006	74
213200	1.006	74
213300	1.006	74
213400	1.006	74
213500	1.006	74
213600	1.006	74

213700	1.006	74
213800	1.006	74
213900	1.006	74
214000	1.006	74
214100	1.006	74
214200	1.006	74
214300	1.006	74
214400	1.006	74
214500	1.005	74
214600	1.005	74
214700	1.005	74
214800	1.005	74
214900	1.005	74
215000	1.005	74
215100	1.005	74
215200	1.005	74
215300	1.005	74
215400	1.005	74
215500	1.005	74
215600	1.005	74
215700	1.005	74
215800	1.005	74
215900	1.005	74
216000	1.005	74
216100	1.005	74
216200	1.005	74
216300	1.005	74
216400	1.005	74
216500	1.005	74
216600	1.005	74
216700	1.005	74
216800	1.005	74
216900	1.005	74
217000	1.005	74
217100	1.005	74
217200	1.005	74
217300	1.005	74
217400	1.005	74
217500	1.005	74
217600	1.005	74
217700	1.005	74
217800	1.005	74
217900	1.005	74
218000	1.005	74
218100	1.005	74
218200	1.005	74
218300	1.005	74
218400	1.005	74
218500	1.005	74
218600	1.005	74
218700	1.005	74
218800	1.005	74
218900	1.005	74
219000	1.005	74
219100	1.005	74
219200	1.005	74
219300	1.005	74
219400	1.005	74
219500	1.006	74
219600	1.006	74
219700	1.006	74
219800	1.006	74
219900	1.006	74
220000	1.006	74
220100	1.006	74
220200	1.006	74
220300	1.006	74
220400	1.006	74
220500	1.006	74
220600	1.006	74
220700	1.006	74
220800	1.007	74
220900	1.007	74
221000	1.007	74

221100	1.007	74
221200	1.007	74
221300	1.007	74
221400	1.007	74
221500	1.006	74
221600	1.006	74
221700	1.006	74
221800	1.006	74
221900	1.006	74
222000	1.006	74
222100	1.006	74
222200	1.006	74
222300	1.006	74
222400	1.006	74
222500	1.006	74
222600	1.006	74
222700	1.006	74
222800	1.006	74
222900	1.006	74
223000	1.006	74
223100	1.006	74
223200	1.006	74
223300	1.006	74
223400	1.006	74
223500	1.006	74
223600	1.006	74
223700	1.006	74
223800	1.006	74
223900	1.006	74
224000	1.006	74
224100	1.006	74
224200	1.006	74
224300	1.006	74
224400	1.006	74
224500	1.006	74
224600	1.006	74
224700	1.006	74
224800	1.006	74
224900	1.006	74
225000	1.006	74
225100	1.006	74
225200	1.006	74
225300	1.006	74
225400	1.006	74
225500	1.006	74
225600	1.006	74
225700	1.006	74
225800	1.006	74
225900	1.006	74
226000	1.005	74
226100	1.005	74
226200	1.005	74
226300	1.005	74
226400	1.005	74
226500	1.005	74
226600	1.005	74
226700	1.005	74
226800	1.005	74
226900	1.005	74
227000	1.005	74
227100	1.005	74
227200	1.005	74
227300	1.005	74
227400	1.005	74
227500	1.005	74
227600	1.005	74
227700	1.005	74
227800	1.005	74
227900	1.005	74
228000	1.005	74
228100	1.005	74
228200	1.005	74
228300	1.005	74
228400	1.005	74

228500	1.005	74
228600	1.005	74
228700	1.005	74
228800	1.005	74
228900	1.005	74
229000	1.005	74
229100	1.005	74
229200	1.005	74
229300	1.005	74
229400	1.005	74
229500	1.005	74
229600	1.005	74
229700	1.005	74
229800	1.005	74
229900	1.005	74
230000	1.005	74
230100	1.005	74
230200	1.005	74
230300	1.005	74
230400	1.005	74
230500	1.005	74
230600	1.005	74
230700	1.005	74
230800	1.005	74
230900	1.005	74
231000	1.005	74
231100	1.005	74
231200	1.005	74
231300	1.005	74
231400	1.005	74
231500	1.005	74
231600	1.005	74
231700	1.005	74
231800	1.005	74
231900	1.005	74
232000	1.005	74
232100	1.005	74
232200	1.005	74
232300	1.005	74
232400	1.005	74
232500	1.005	74
232600	1.005	74
232700	1.005	74
232800	1.005	74
232900	1.005	74
233000	1.005	74
233100	1.005	74
233200	1.005	74
233300	1.005	74
233400	1.005	74
233500	1.005	74
233600	1.005	74
233700	1.005	74
233800	1.005	74
233900	1.005	74
234000	1.005	74
234100	1.005	74
234200	1.005	74
234300	1.004	74
234400	1.004	74
234500	1.005	74
234600	1.005	74
234700	1.005	74
234800	1.005	74
234900	1.005	74
235000	1.005	74
235100	1.005	74
235200	1.005	74
235300	1.005	74
235400	1.005	74
235500	1.005	74
235600	1.005	74
235700	1.005	74
235800	1.005	74

```
235900           1.005            74
236000           1.005            74
236100           1.004            74
236200           1.004            74
236300           1.004            74
236400           1.004            74
236500           1.004            74
236600           1.004            74
236700           1.004            74
236800           1.004            74
236900           1.004            74
237000           1.004            74
237100           1.004            74
237200           1.004            74
237300           1.004            74
237400           1.004            74
237500           1.004            74
237600           1.004            74
237700           1.004            74
237800           1.004            74
237900           1.004            74
238000           1.004            74
238100           1.004            74
238200           1.004            74
238300           1.004            74
238400           1.004            74
238500           1.004            74
238600           1.004            74
238700           1.004            74
238800           1.004            74
238900           1.004            74
239000           1.004            74
239100           1.004            74
239200           1.004            74
239300           1.004            74
239400           1.004            74
239500           1.004            74
239600           1.004            74
239700           1.004            74
239800           1.004            3
239900           1.005            3
240000           1.005            3
240100           1.005            3
240200           1.005            3
240300           1.006            3
240400           1.006            3
240500           1.006            3
240600           1.006            3
240700           1.005            3
240800           1.006            3
240900           1.006            3
241000           1.006            3
241100           1.006            3
241200           1.006            3
241300           1.006            3
241400           1.005            3
241500           1.005            3
241600           1.005            3
241700           1.005            3
241800           1.005            3
241900           1.005            3
242000           1.005            3
242100           1.005            3
242200           1.005            3
242300           1.005            3
242400           1.005            3
242500           1.005            3
242600           1.005            3
242700           1.005            3
242800           1.005            3
242900           1.005            3
243000           1.005            3
243100           1.005            3
243200           1.005            3
```

```
243300          1.005          3
243400          1.005          3
243500          1.005          3
243600          1.005          3
243700          1.005          3
243800          1.005          3
243900          1.005          3
244000          1.005          3
244100          1.005          3
244200          1.005          3
244300          1.005          3
244400          1.005          3
244500          1.005          3
244600          1.005          3
244700          1.005          3
244800          1.005          3
244900          1.005          3
245000          1.005          3
245100          1.005          3
245200          1.005          3
245300          1.005          3
245400          1.005          3
245500          1.005          3
245600          1.005          3
245700          1.005          3
245800          1.005          3
245900          1.005          3
246000          1.005          3
246100          1.005          3
246200          1.005          3
246300          1.005          3
246400          1.005          3
246500          1.004          3
246600          1.004          3
246700          1.004          3
246800          1.004          3
246900          1.004          3
247000          1.004          3
247100          1.004          3
247200          1.005          3
247300          1.005          3
247400          1.005          3
247500          1.005          3
247600          1.005          3
247700          1.005          3
247800          1.005          3
247900          1.005          3
248000          1.005          3
248100          1.005          3
248200          1.005          3
248300          1.005          3
248400          1.005          3
248500          1.005          3
248600          1.005          3
248700          1.005          3
248800          1.006          3
248900          1.006          3
249000          1.006          3
249100          1.006          3
249200          1.006          3
249300          1.006          3
249400          1.006          3
249500          1.006          3
249600          1.006          3
249700          1.006          3
249800          1.006          3
249900          1.006          3
250000          1.006          3
250100          1.007          3
250200          1.007          3
250300          1.007          3
250400          1.007          3
250500          1.007          3
250600          1.007          3
```

250700	1.007	3
250800	1.007	3
250900	1.007	3
251000	1.007	3
251100	1.006	3
251200	1.006	3
251300	1.006	3
251400	1.006	3
251500	1.006	3
251600	1.007	3
251700	1.007	3
251800	1.007	3
251900	1.007	3
252000	1.007	3
252100	1.007	3
252200	1.007	3
252300	1.007	3
252400	1.007	3
252500	1.007	3
252600	1.007	3
252700	1.007	3
252800	1.007	3
252900	1.007	3
253000	1.007	3
253100	1.006	3
253200	1.006	3
253300	1.006	3
253400	1.006	3
253500	1.006	3
253600	1.006	3
253700	1.006	3
253800	1.006	3
253900	1.005	3
254000	1.005	3
254100	1.005	3
254200	1.005	3
254300	1.005	3
254400	1.005	3
254500	1.005	3
254600	1.005	3
254700	1.005	3
254800	1.005	3
254900	1.005	3
255000	1.005	3
255100	1.005	3
255200	1.005	3
255300	1.005	3
255400	1.005	3
255500	1.005	3
255600	1.005	3
255700	1.005	3
255800	1.006	3
255900	1.006	3
256000	1.006	3
256100	1.005	3
256200	1.005	3
256300	1.005	3
256400	1.005	3
256500	1.005	3
256600	1.005	3
256700	1.005	3
256800	1.005	3
256900	1.006	3
257000	1.005	3
257100	1.006	3
257200	1.006	3
257300	1.006	3
257400	1.006	3
257500	1.006	3
257600	1.006	3
257700	1.006	3
257800	1.006	3
257900	1.006	3
258000	1.006	3

258100	1.006	3
258200	1.006	3
258300	1.006	3
258400	1.006	3
258500	1.006	3
258600	1.007	3
258700	1.007	3
258800	1.007	3
258900	1.008	3
259000	1.008	3
259100	1.008	3
259200	1.008	3
259300	1.008	3
259400	1.008	3
259500	1.008	3
259600	1.008	3
259700	1.008	3
259800	1.008	3
259900	1.008	3
260000	1.008	3
260100	1.008	3
260200	1.008	3
260300	1.008	3
260400	1.008	3
260500	1.008	3
260600	1.008	3
260700	1.008	3
260800	1.007	3
260900	1.007	3
261000	1.007	3
261100	1.007	3
261200	1.007	3
261300	1.007	3
261400	1.007	3
261500	1.007	3
261600	1.007	3
261700	1.007	3
261800	1.007	3
261900	1.007	3
262000	1.007	3
262100	1.007	3
262200	1.007	3
262300	1.007	3
262400	1.007	3
262500	1.007	3
262600	1.007	3
262700	1.007	3
262800	1.007	3
262900	1.007	3
263000	1.007	3
263100	1.007	3
263200	1.007	3
263300	1.007	3
263400	1.007	3
263500	1.007	3
263600	1.007	3
263700	1.007	3
263800	1.007	3
263900	1.007	3
264000	1.007	3
264100	1.007	3
264200	1.007	3
264300	1.007	3
264400	1.007	3
264500	1.007	3
264600	1.007	3
264700	1.007	3
264800	1.007	3
264900	1.007	3
265000	1.007	3
265100	1.007	3
265200	1.007	3
265300	1.007	3
265400	1.007	3

265500	1.007	3
265600	1.007	3
265700	1.007	3
265800	1.007	3
265900	1.007	3
266000	1.007	3
266100	1.007	3
266200	1.007	3
266300	1.007	3
266400	1.007	3
266500	1.007	3
266600	1.007	3
266700	1.008	3
266800	1.008	3
266900	1.008	3
267000	1.009	3
267100	1.009	3
267200	1.009	3
267300	1.009	3
267400	1.009	3
267500	1.009	3
267600	1.009	3
267700	1.009	3
267800	1.009	3
267900	1.009	3
268000	1.009	3
268100	1.009	3
268200	1.008	3
268300	1.008	3
268400	1.008	3
268500	1.008	3
268600	1.008	3
268700	1.008	3
268800	1.008	3
268900	1.008	3
269000	1.008	3
269100	1.008	3
269200	1.008	3
269300	1.008	3
269400	1.008	3
269500	1.008	3
269600	1.008	3
269700	1.008	3
269800	1.008	3
269900	1.008	3
270000	1.008	3
270100	1.008	3
270200	1.008	3
270300	1.008	3
270400	1.008	3
270500	1.008	3
270600	1.008	3
270700	1.008	3
270800	1.008	3
270900	1.008	3
271000	1.008	3
271100	1.008	3
271200	1.008	3
271300	1.008	3
271400	1.008	3
271500	1.009	3
271600	1.009	3
271700	1.009	3
271800	1.009	3
271900	1.009	3
272000	1.009	3
272100	1.009	3
272200	1.009	3
272300	1.009	3
272400	1.009	3
272500	1.009	3
272600	1.009	3
272700	1.009	3
272800	1.009	3

```
272900           1.009            3
273000           1.008            3
273100           1.008            3
273200           1.008            3
273300           1.008            3
273400           1.009            3
273500           1.009            3
273600           1.009            3
273700           1.009            3
273800           1.009            3
273900           1.009            3
274000           1.009            3
274100           1.009            3
274200           1.009            3
274300           1.009            3
274400           1.009            3
274500           1.009            3
274600           1.009            3
274700           1.009            3
274800           1.009            3
274900           1.009            3
275000           1.009            3
275100           1.009            3
275200           1.009            3
275300           1.009            3
275400           1.009            3
275500           1.009            3
275600           1.009            3
275700           1.009            3
275800           1.010            3
275900           1.010            3
276000           1.010            3
276100           1.010            3
276200           1.010            3
276300           1.010            3
276400           1.010            3
276500           1.010            3
276600           1.010            3
276700           1.010            3
276800           1.010            3
276900           1.010            3
277000           1.010            3
277100           1.010            3
277200           1.010            3
277300           1.010            3
277400           1.010            3
277500           1.010            3
277600           1.010            3
277700           1.010            3
277800           1.010            3
277900           1.010            3
278000           1.010            3
278100           1.010            3
278200           1.010            3
278300           1.010            3
278400           1.009            3
278500           1.009            3
278600           1.009            3
278700           1.009            3
278800           1.009            3
278900           1.009            3
279000           1.009            3
279100           1.009            3
279200           1.009            3
279300           1.009            3
279400           1.009            3
279500           1.009            3
279600           1.009            3
279700           1.009            3
279800           1.009            3
279900           1.009            3
280000           1.009            3
280100           1.009            3
280200           1.009            3
```

```
280300          1.009          3
280400          1.009          3
280500          1.009          3
280600          1.009          3
280700          1.009          3
280800          1.009          3
280900          1.009          3
281000          1.009          3
281100          1.009          3
281200          1.009          3
281300          1.009          3
281400          1.009          3
281500          1.009          3
281600          1.009          3
281700          1.009          3
281800          1.009          3
281900          1.009          3
282000          1.009          3
282100          1.009          3
282200          1.009          3
282300          1.009          3
282400          1.009          3
282500          1.009          3
282600          1.009          3
282700          1.009          3
282800          1.009          3
282900          1.009          3
283000          1.009          3
283100          1.009          3
283200          1.009          3
283300          1.009          3
283400          1.009          3
283500          1.009          3
283600          1.009          3
283700          1.009          3
283800          1.009          3
283900          1.009          3
284000          1.009          3
284100          1.009          3
284200          1.009          3
284300          1.009          3
284400          1.009          3
284500          1.009          3
284600          1.009          3
284700          1.009          3
284800          1.009          3
284900          1.009          3
285000          1.009          3
285100          1.009          3
285200          1.009          3
285300          1.009          3
285400          1.009          3
285500          1.009          3
285600          1.009          3
285700          1.009          3
285800          1.009          3
285900          1.009          3
286000          1.009          3
286100          1.009          3
286200          1.009          3
286300          1.009          3
286400          1.009          3
286500          1.009          3
286600          1.009          3
286700          1.008          3
286800          1.008          3
286900          1.008          3
287000          1.008          3
287100          1.008          3
287200          1.008          3
287300          1.008          3
287400          1.008          3
287500          1.008          3
287600          1.008          3
```

```
     287700              1.008              3
     287800              1.007              3
     287900              1.007              3
     288000              1.007              3
     288100              1.007              3
     288200              1.007              3
     288300              1.007              3
     288400              1.007              3
     288500              1.007              3
     288600              1.007              3
     288700              1.007              3
     288800              1.007              3
     288900              1.007              3
     289000              1.007              3
     289100              1.007              3
     289200              1.007              3
     289300              1.007              3
     289400              1.007              3
     289500              1.007              3
     289600              1.007              3
     289700              1.007              3
     289800              1.007              3
     289900              1.008              3
     290000              1.008              3
     290100              1.008              3
     290200              1.007              3
     290300              1.007              3
     290400              1.007              3
     290500              1.007              3
     290600              1.007              3
     290700              1.007              3
     290800              1.007              3
     290900              1.007              3
     291000              1.008              3
     291100              1.008              3
     291200              1.008              3
     291300              1.008              3
     291400              1.007              3
     291500              1.007              3
     291600              1.007              3
     291700              1.007              3
     291800              1.007              3
     291900              1.007              3
     292000              1.007              3
     292100              1.007              3
     292200              1.007              3
     292300              1.007              3
     292400              1.007              3
     292500              1.007              3
     292600              1.007              3
     292700              1.007              3
     292800              1.007              3
     292900              1.007              3
     293000              1.007              3
     293100              1.007              3
     293200              1.007              3
     293300              1.007              3
     293400              1.007              3
     293500              1.007              3
     293600              1.007              3
     293700              1.007              3
     293800              1.007              3
     293900              1.007              3
     294000              1.007              3
     294100              1.007              3
     294200              1.007              3
     294300              1.007              3
     294400              1.007              3
     294500              1.007              3
     294600              1.007              3
     294700              1.007              3
     294800              1.007              3
     294900              1.007              3
     295000              1.007              3
```

295100	1.007	3
295200	1.007	3
295300	1.007	3
295400	1.007	3
295500	1.007	3
295600	1.007	3
295700	1.007	3
295800	1.007	3
295900	1.007	3
296000	1.007	3
296100	1.007	3
296200	1.007	3
296300	1.007	3
296400	1.007	3
296500	1.007	3
296600	1.007	3
296700	1.007	3
296800	1.007	3
296900	1.007	3
297000	1.007	3
297100	1.007	3
297200	1.007	3
297300	1.007	3
297400	1.007	3
297500	1.007	3
297600	1.007	3
297700	1.007	3
297800	1.007	3
297900	1.007	3
298000	1.007	3
298100	1.007	3
298200	1.007	3
298300	1.007	3
298400	1.007	3
298500	1.007	3
298600	1.007	3
298700	1.007	3
298800	1.007	3
298900	1.007	3
299000	1.007	3
299100	1.007	3
299200	1.007	3
299300	1.007	3
299400	1.007	3
299500	1.007	3
299600	1.008	3
299700	1.008	3
299800	1.008	3
299900	1.008	3
300000	1.008	3
300100	1.008	3
300200	1.008	3
300300	1.008	3
300400	1.008	3
300500	1.008	3
300600	1.008	3
300700	1.008	3
300800	1.008	3
300900	1.008	3
301000	1.008	3
301100	1.008	3
301200	1.007	3
301300	1.007	3
301400	1.007	3
301500	1.007	3
301600	1.007	3
301700	1.007	3
301800	1.007	3
301900	1.007	3
302000	1.007	3
302100	1.007	3
302200	1.007	3
302300	1.007	3
302400	1.007	3

```
     302500          1.008              3
     302600          1.008              3
     302700          1.008              3
     302800          1.008              3
     302900          1.008              3
     303000          1.008              3
     303100          1.008              3
     303200          1.008              3
     303300          1.008              3
     303400          1.008              3
     303500          1.008              3
     303600          1.008              3
     303700          1.009              3
     303800          1.009              3
     303900          1.009              3
     304000          1.009              3
     304100          1.009              3
     304200          1.009              3
     304300          1.009              3
     304400          1.009              3
     304500          1.009              3
     304600          1.009              3
     304700          1.009              3
     304800          1.009              3
     304900          1.009              3
     305000          1.009              3
     305100          1.009              3
     305200          1.009              3
     305300          1.009              3
     305400          1.009              3
     305500          1.009              3
     305600          1.009              3
     305700          1.009              3
     305800          1.009              3
     305900          1.009              3
     306000          1.009              3
     306100          1.009              3
     306200          1.009              3
     306300          1.009              3
     306400          1.009              3
     306500          1.009              3
     306600          1.009              3
     306700          1.009              3
     306800          1.009              3
     306900          1.009              3
     307000          1.009              3
     307100          1.009              3
     307200          1.009              3
     307300          1.009              3
     307400          1.009              3
     307500          1.009              3
     307600          1.009              3
     307700          1.009              3
     307800          1.009              3
     307900          1.009              3
     308000          1.009              3
     308100          1.009              3
     308200          1.009              3
     308300          1.009              3
     308400          1.009              3
     308500          1.009              3
     308600          1.009              3
     308700          1.009              3
     308800          1.009              3
     308900          1.009              3
     309000          1.009              3
     309100          1.009              3
     309200          1.009              3
     309300          1.010              3
     309400          1.010              3
     309500          1.010              3
     309600          1.010              3
     309700          1.010              3
     309800          1.010              3
```

309900	1.010	3
310000	1.010	3
310100	1.010	3
310200	1.010	3
310300	1.010	3
310400	1.010	3
310500	1.010	3
310600	1.010	3
310700	1.010	3
310800	1.010	3
310900	1.010	3
311000	1.010	3
311100	1.010	3
311200	1.010	3
311300	1.010	3
311400	1.010	3
311500	1.010	3
311600	1.010	3
311700	1.010	3
311800	1.010	3
311900	1.010	3
312000	1.010	3
312100	1.010	3
312200	1.011	3
312300	1.011	3
312400	1.011	3
312500	1.011	3
312600	1.011	3
312700	1.011	3
312800	1.011	3
312900	1.010	3
313000	1.010	3
313100	1.010	3
313200	1.010	3
313300	1.010	3
313400	1.010	3
313500	1.010	3
313600	1.010	3
313700	1.010	3
313800	1.010	3
313900	1.010	3
314000	1.010	3
314100	1.010	3
314200	1.010	3
314300	1.010	3
314400	1.010	3
314500	1.010	3
314600	1.010	3
314700	1.010	3
314800	1.010	3
314900	1.010	3
315000	1.010	3
315100	1.010	3
315200	1.010	3
315300	1.010	3
315400	1.010	3
315500	1.010	3
315600	1.010	3
315700	1.010	3
315800	1.010	3
315900	1.011	3
316000	1.011	3
316100	1.011	3
316200	1.011	3
316300	1.011	3
316400	1.011	3
316500	1.011	3
316600	1.010	3
316700	1.010	3
316800	1.010	3
316900	1.010	3
317000	1.010	3
317100	1.010	3
317200	1.010	3

```
317300          1.010           3
317400          1.010           3
317500          1.010           3
317600          1.010           3
317700          1.010           3
317800          1.010           3
317900          1.010           3
318000          1.010           3
318100          1.010           3
318200          1.010           3
318300          1.010           3
318400          1.010           3
318500          1.010           3
318600          1.010           3
318700          1.010           3
318800          1.010           3
318900          1.010           3
319000          1.009           3
319100          1.009           3
319200          1.009           3
319300          1.009           3
319400          1.009           3
319500          1.009           3
319600          1.009           3
319700          1.009           3
319800          1.009           3
319900          1.009           3
320000          1.010           3
320100          1.010           3
320200          1.010           3
320300          1.010           3
320400          1.010           3
320500          1.010           3
320600          1.010           3
320700          1.010           3
320800          1.010           3
320900          1.010           3
321000          1.010           3
321100          1.010           3
321200          1.010           3
321300          1.010           3
321400          1.010           3
321500          1.010           3
321600          1.010           3
321700          1.010           3
321800          1.010           3
321900          1.010           3
322000          1.010           3
322100          1.010           3
322200          1.010           3
322300          1.010           3
322400          1.010           3
322500          1.010           3
322600          1.010           3
322700          1.010           3
322800          1.010           3
322900          1.010           3
323000          1.010           3
323100          1.010           3
323200          1.010           3
323300          1.010           3
323400          1.010           3
323500          1.010           3
323600          1.010           3
323700          1.010           3
323800          1.010           3
323900          1.010           3
324000          1.010           3
324100          1.010           3
324200          1.010           3
324300          1.010           3
324400          1.010           3
324500          1.010           3
324600          1.010           3
```

324700	1.010	3
324800	1.010	3
324900	1.010	3
325000	1.010	3
325100	1.011	3
325200	1.011	3
325300	1.011	3
325400	1.011	3
325500	1.011	3
325600	1.011	3
325700	1.010	3
325800	1.010	3
325900	1.010	3
326000	1.010	3
326100	1.010	3
326200	1.010	3
326300	1.010	3
326400	1.010	3
326500	1.010	3
326600	1.010	3
326700	1.010	3
326800	1.010	3
326900	1.010	3
327000	1.010	3
327100	1.010	3
327200	1.010	3
327300	1.010	3
327400	1.010	3
327500	1.009	3
327600	1.009	3
327700	1.009	3
327800	1.009	3
327900	1.009	3
328000	1.009	3
328100	1.009	3
328200	1.009	3
328300	1.009	3
328400	1.009	3
328500	1.009	3
328600	1.009	3
328700	1.009	3
328800	1.009	3
328900	1.009	3
329000	1.009	3
329100	1.009	3
329200	1.009	3
329300	1.009	3
329400	1.009	3
329500	1.008	3
329600	1.008	3
329700	1.008	3
329800	1.008	3
329900	1.008	3
330000	1.008	3
330100	1.008	3
330200	1.008	3
330300	1.008	3
330400	1.008	3
330500	1.008	3
330600	1.008	3
330700	1.008	3
330800	1.008	3
330900	1.008	3
331000	1.008	3
331100	1.008	3
331200	1.008	3
331300	1.008	3
331400	1.008	3
331500	1.007	3
331600	1.007	3
331700	1.007	3
331800	1.007	3
331900	1.007	3
332000	1.007	3

332100	1.007	3
332200	1.007	3
332300	1.008	3
332400	1.007	3
332500	1.008	3
332600	1.008	3
332700	1.008	3
332800	1.008	3
332900	1.008	3
333000	1.008	3
333100	1.008	3
333200	1.008	3
333300	1.008	3
333400	1.008	3
333500	1.008	3
333600	1.008	3
333700	1.008	3
333800	1.007	3
333900	1.007	3
334000	1.007	3
334100	1.007	3
334200	1.007	3
334300	1.007	3
334400	1.007	3
334500	1.008	3
334600	1.008	3
334700	1.008	3
334800	1.007	3
334900	1.007	3
335000	1.007	3
335100	1.007	3
335200	1.007	3
335300	1.007	3
335400	1.007	3
335500	1.007	3
335600	1.007	3
335700	1.007	3
335800	1.007	3
335900	1.007	3
336000	1.008	3
336100	1.008	3
336200	1.008	3
336300	1.008	3
336400	1.008	3
336500	1.008	3
336600	1.008	3
336700	1.008	3
336800	1.008	3
336900	1.008	3
337000	1.008	3
337100	1.008	3
337200	1.008	3
337300	1.008	3
337400	1.008	3
337500	1.008	3
337600	1.008	3
337700	1.007	3
337800	1.007	3
337900	1.007	3
338000	1.007	3
338100	1.007	3
338200	1.007	3
338300	1.007	3
338400	1.007	3
338500	1.007	3
338600	1.007	3
338700	1.007	3
338800	1.007	3
338900	1.006	3
339000	1.006	3
339100	1.006	3
339200	1.006	3
339300	1.006	3
339400	1.006	3

339500	1.006	3
339600	1.006	3
339700	1.006	3
339800	1.006	3
339900	1.006	3
340000	1.006	3
340100	1.006	3
340200	1.006	3
340300	1.006	3
340400	1.006	3
340500	1.006	3
340600	1.006	3
340700	1.006	3
340800	1.006	3
340900	1.006	3
341000	1.006	3
341100	1.006	3
341200	1.006	3
341300	1.006	3
341400	1.006	3
341500	1.006	3
341600	1.006	3
341700	1.006	3
341800	1.006	3
341900	1.006	3
342000	1.006	3
342100	1.006	3
342200	1.006	3
342300	1.006	3
342400	1.006	3
342500	1.006	3
342600	1.006	3
342700	1.006	3
342800	1.006	3
342900	1.006	3
343000	1.006	3
343100	1.006	3
343200	1.006	3
343300	1.006	3
343400	1.006	3
343500	1.006	3
343600	1.006	3
343700	1.006	3
343800	1.006	3
343900	1.006	3
344000	1.006	3
344100	1.006	3
344200	1.006	3
344300	1.006	3
344400	1.006	3
344500	1.006	3
344600	1.006	3
344700	1.006	3
344800	1.006	3
344900	1.006	3
345000	1.006	3
345100	1.006	3
345200	1.006	3
345300	1.006	3
345400	1.006	3
345500	1.006	3
345600	1.006	3
345700	1.006	3
345800	1.006	3
345900	1.006	3
346000	1.006	3
346100	1.006	3
346200	1.006	3
346300	1.006	3
346400	1.006	3
346500	1.006	3
346600	1.006	3
346700	1.006	3
346800	1.006	3

346900	1.006	3
347000	1.006	3
347100	1.006	3
347200	1.006	3
347300	1.006	3
347400	1.006	3
347500	1.006	3
347600	1.006	3
347700	1.006	3
347800	1.006	3
347900	1.006	3
348000	1.006	3
348100	1.006	3
348200	1.006	3
348300	1.006	3
348400	1.006	3
348500	1.006	3
348600	1.006	3
348700	1.006	3
348800	1.006	3
348900	1.006	3
349000	1.006	3
349100	1.006	3
349200	1.006	3
349300	1.006	3
349400	1.006	3
349500	1.006	3
349600	1.006	3
349700	1.006	3
349800	1.005	3
349900	1.005	3
350000	1.005	3
350100	1.005	3
350200	1.005	3
350300	1.005	3
350400	1.005	3
350500	1.005	3
350600	1.004	3
350700	1.004	3
350800	1.004	3
350900	1.004	3
351000	1.004	3
351100	1.004	3
351200	1.004	3
351300	1.004	3
351400	1.004	3
351500	1.004	3
351600	1.004	3
351700	1.004	3
351800	1.004	3
351900	1.004	3
352000	1.004	3
352100	1.004	3
352200	1.004	3
352300	1.004	3
352400	1.003	3
352500	1.003	3
352600	1.003	3
352700	1.003	3
352800	1.003	3
352900	1.003	3
353000	1.003	3
353100	1.003	3
353200	1.003	3
353300	1.003	3
353400	1.003	3
353500	1.003	3
353600	1.003	3
353700	1.003	3
353800	1.003	3
353900	1.003	3
354000	1.003	3
354100	1.003	3
354200	1.003	3

```
     354300           1.003              3
     354400           1.003              3
     354500           1.003              3
     354600           1.003              3
     354700           1.002              3


TECHNICAL 5 OUTPUT


DIAGRAM INFORMATION

  Mplus diagrams are currently not available for multilevel analysis.
  No diagram output was produced.


     Beginning Time:  12:11:02
        Ending Time:  19:27:50
       Elapsed Time:  07:16:48
```


```
MUTHEN & MUTHEN
3463 Stoner Ave.
Los Angeles, CA  90066

Tel: (310) 391-9971
Fax: (310) 391-8971
Web: www.StatModel.com
Support: Support@StatModel.com
```


Copyright (c) 1998-2019 Muthen & Muthen
