# Supplementary material for: Associations of Wellbeing Levels, Changes, and Within-Person Variability With Late-Life All-Cause Mortality Across 12 Years: Contrasting Hedonic vs. Eudaimonic Wellbeing Among Very Old Adults
Source: Front Psychol. 2022 Jan 11;12:750891. doi: 10.3389/fpsyg.2021.750891 (PMC8787264; doi:10.3389/fpsyg.2021.750891)
Supplement: Supplementary file 2 [file Data_Sheet_2.PDF]

Associations of Well-Being Levels, Changes and Within-Person Variability with Late-Life All-Cause Mortality Across 12 Years: Contrasting Hedonic vs. Eudaimonic Well-Being Among Very Old Adults

Oliver Schilling, Markus Wettstein, Hans-Werner-Wahl

Online Supplement: Additional Results From Two-Step Cox Regression Analyses

To check whether the findings from the multilevel SEM analyses would hold in a more conventional survival analysis, we additionally ran two-step analyses as follows: First, we estimated the within-person location scale model on the well-being indicators and retained the level, slope, and detrendend variance random effects (i.e., the individual estimates of these parameters) in the database. Second, we ran Cox proportional hazard models with these estimates as predictors of survival. This analytical approach, however, also has a crucial disadvantage, in that it does not account for differential reliability of the estimates obtained from the first step. Specifically, reliability of the location scale estimates may depend on the numbers of measurement occasions the respective individual participated in. In particular, for those who dropped out of the study early, hence providing, say, less than four self-reports on their well-being, there is a lack of information needed to reliably decompose these self-reports into a level, slope, and intra-individual residual variance component. This means a missing data problem, which is dealt by the missing-at-random treatment implied in the Bayes estimation. In the one-step multilevel SEM approach, this might result in less biased estimation of the survival equation parameters, because it accounts for the degree of uncertainty of parameter estimates due to missingness (Graham, 2009). Thus, we mainly focus on the multilevel SEM results and report the two-step-findings for comparison reasons only.

Results of the second step – i.e. Cox regression coefficient estimates and confidence intervals – are shown in Tables S1-S3. Note that the coefficients indicate effects on the hazards of dying, hence a negative sign indicates lowered risk of dying earlier associated with higher scores on the respective predictor.

Table S1: Continuous Time Survival Predicted by Pre-Estimated Scores of Level, Slope, and Detrended Variance of Autonomy, Environmental Mastery, Purpose In Life, and Self-Acceptance

		Estimate	*SE*	95% CI	Standard. Estimate
**Autonomy**	Level	0.381*	0.175	0.038 - 0.724	0.211
	Slope	11.079	8.373	−5.331 - 27.489	0.116
	Variance	−0.641*	0.286	−1.202 - −0.080	−0.197
Age		0.112*	0.030	0.053 - 0.172	0.323
Sex		−0.374	0.232	−0.829 - 0.081	−0.150
Education		0.070*	0.031	0.010 - 0.130	0.203
Physical Condition		−0.410*	0.061	−0.530 - −0.290	−0.525
**Environ. Mastery**	Level	−0.129	0.211	−0.542 - 0.285	−0.078
	Slope	−1.273	7.189	−15.362 - 12.817	−0.016
	Variance	−1.546	0.852	−3.216 - 0.124	−0.162
Age		0.098*	0.030	0.039 - 0.158	0.283
Sex		−0.578	0.521	−1.599 - 0.443	−0.237
Education		0.103*	0.038	0.029 - 0.177	0.302
Physical Condition		−0.230*	0.082	−0.392 - −0.069	−0.537
**Purpose in Life**	Level	0.006	0.167	−0.321 - 0.332	0.004
	Slope	5.158	9.531	−13.523 - 23.839	0.051
	Variance	−0.295	0.267	−0.818 - 0.228	−0.094
Age		0.102*	0.032	0.039 - 0.166	0.294
Sex		−0.527	0.414	−1.339 - 0.284	−0.212
Education		0.091*	0.037	0.017 - 0.164	0.259
Physical Condition		−0.237*	0.082	−0.398 - −0.076	−0.573
**Self Acceptance**	Level	0.041	0.160	−0.272 - 0.354	0.026
	Slope	−3.485	7.654	−18.486 - 11.517	−0.042
	Variance	−0.139	0.238	−0.607 - 0.328	−0.055
Age		0.090*	0.030	0.032 - 0.149	0.259
Sex		−0.494	0.426	−1.330 - 0.342	−0.202
Education		0.084*	0.037	0.011 - 0.156	0.245
Physical Condition		−0.246*	0.090	−0.421 - −0.070	−0.549

Note. Results from Cox regression model (run with Mplus; Muthén & Muthén (1998-2017)).
* $p < 0.05$.

Table S2: Continuous Time Survival Predicted by Pre-Estimated Scores of Level, Slope, and Variance of Life Satisfaction, Positive Affect, and Negative Affect

		Estimate	*SE*	95% CI	Standard. Estimate
**Life Satisfaction**	Level	−0.013	0.157	−0.320 - 0.294	-0.011
	Slope	3.942	7.717	−11.183 - 19.067	0.044
	Variance	−0.235	0.224	−0.674 - 0.203	-0.094
Age		0.109*	0.031	0.048 - 0.169	0.316
Sex		−0.426	0.356	−1.124 - 0.272	-0.172
Education		0.096*	0.031	0.036 - 0.157	0.273
Physical Condition		−0.233*	0.080	−0.390 - −0.075	−0.510
**Positive Affect**	Level	−0.091	0.149	−0.383 - 0.202	−0.060
	Slope	−7.234	8.101	−23.111 - 8.644	−0.097
	Variance	−0.139	0.292	−0.711 - 0.433	−0.042
Age		0.096*	0.030	0.036 - 0.155	0.276
Sex		−0.347	0.290	−0.915 - 0.220	−0.142
Education		0.099*	0.030	0.039 - 0.158	0.285
Physical Condition		−0.227*	0.079	−0.381 - −0.073	−0.457
**Negative Affect**	Level	0.077	0.197	−0.310 - 0.464	0.047
	Slope	1.706	13.969	25.674 - 29.086	0.017
	Variance	−0.592	0.376	−1.330 - 0.145	−0.151
Age		0.104*	0.032	0.040 - 0.167	0.300
Sex		−0.399	0.331	−1.049 - 0.250	−0.162
Education		0.095*	0.035	0.027 - 0.163	0.275
Physical Condition		−0.261*	0.095	−0.448 - −0.074	−0.535

Note. Results from Cox regression model (run with Mplus; Muthén & Muthén (1998-2017)).
* $p < 0.05$.

Table S3: Continuous Time Survival Predicted by Pre-Estimated Scores of Level, Slope, and Variance of Well-Being, Full Model Including All Predictors

		Estimate	SE	95% CI	Standard. Estimate
Autonomy	Level	0.588*	0.250	0.099 - 1.077	0.332
	Slope	15.189	12.480	−9.271 - 39.650	0.162
	Variance	−0.898*	0.357	−1.598 - −0.198	−0.284
Environ. Mastery	Level	−0.373	0.378	−1.113 - 0.368	−0.231
	Slope	−3.750	10.171	−23.685 - 16.185	−0.048
	Variance	−0.707	1.021	−2.708 - 1.295	−0.077
Purpose in Life	Level	0.156	0.208	−0.251 - 0.564	0.109
	Slope	14.996	12.969	−10.423 - 40.414	0.151
	Variance	−0.043	0.364	−0.756 - 0.671	−0.014
Self Acceptance	Level	0.713*	0.316	0.093 - 1.332	0.444
	Slope	4.291	13.279	−21.737 - 30.318	0.052
	Variance	0.342	0.275	−0.197 - 0.880	0.139
Life Satisfaction	Level	−0.061	0.184	−0.421 - 0.299	−0.053
	Slope	−12.781	17.738	−47.547 - 21.985	−0.133
	Variance	0.127	0.303	−0.466 - 0.720	0.051
Positive Affect	Level	−0.546*	0.223	−0.983 - −0.109	−0.360
	Slope	−0.990	9.601	−19.808 - 17.829	−0.013
	Variance	0.014	0.300	−0.574 - 0.602	0.004
Negative Affect	Level	0.429	0.281	−0.122 - 0.980	0.261
	Slope	4.160	12.159	−19.672 - 27.993	0.042
	Variance	−0.735	0.485	−1.684 - 0.215	−0.191
Age		0.161*	0.039	0.084 - 0.238	0.469
Sex		−0.347	0.306	−0.947 - 0.252	−0.142
Education		0.061	0.046	−0.029 - 0.152	0.171
Physical Condition		−0.202*	0.102	−0.401 - −0.002	−0.432

Note. Results from Cox regression model (run with Mplus, Muthén & Muthén (1998-2017)). * $p < 0.05$
